# Supplementary material for: Amyotrophic lateral sclerosis and parkinsonism in Papua, Indonesia: 2001–2012 survey results
Source: BMJ Open. 2014 Apr 16;4(4):e004353. doi: 10.1136/bmjopen-2013-004353 (PMC3996815; doi:10.1136/bmjopen-2013-004353)
Supplement: Author's manuscript [file bmjopen-2013-004353.draft_revisions.pdf]

Amyotrophic lateral sclerosis and parkinsonism in Papua, Indonesia: 2001–2012 survey results

Journal:	*BMJ Open*
Manuscript ID:	bmjopen-2013-004353
Article Type:	Research
Date Submitted by the Author:	29-Oct-2013
Complete List of Authors:	Okumiya, Kiyohito; Research Institute for Humanity and Nature,<br>Wada, Taizo; Kyoto University, Center for Southeast Asian Studies<br>Fujisawa, Michiko; Center for Southeast Asian Studies,<br>Ishine, Masayuki; Yasugi Clinic,<br>Garcia del Saz, Eva; Kochi University, Center for Regional & International Collaboration<br>Hirata, Yutaka; Kitaakita Municipal Hospital,<br>Kuzuhara, Shigeki; Suzuka University of Medical Science, Faculty of Health Science<br>Kokubo, Yasumasa; Mie University, Graduate School of Regional Innovation Studies<br>Seguchi, Harumichi; Kochi University, Faculty of Medicine<br>Sakamoto, Ryota; Kyoto University, Hakubi Center for Advanced Research<br>Manuaba, Indrajaya; Cenderawasih University, Faculty of Medicine<br>Watofa, Paulina; Cenderawasih University, Faculty of Medicine<br>Rantetampang, Andreas; Cenderawasih University, Faculty of Public Health<br>Matsubayashi, Kozo; Kyoto University, Center for Southeast Asian Studies
**Primary Subject Heading**:	Epidemiology
Secondary Subject Heading:	Neurology
Keywords:	Motor neurone disease < NEUROLOGY, Parkinson-s disease < NEUROLOGY, Dementia < NEUROLOGY

SCHOLARONE™ Manuscripts

Amyotrophic lateral sclerosis and parkinsonism in Papua, Indonesia: 2001–2012 survey results

Kiyohito Okumiya[1], Taizo Wada[2], Michiko Fujisawa[2], Masayuki Ishine[3], Eva Garcia del Saz[4], Yutaka Hirata[5], Shigeki Kuzuhara[6], Yasumasa Kokubo[7], Harumichi Seguchi[8], Ryota Sakamoto[9], Indrajaya Manuaba[10], Paulina Watofa[10], Andreas L. Rantetampang[11], Kozo Matsubayashi[2]

[1]Research Institute for Humanity and Nature, Kyoto, Japan
[2]Center for Southeast Asian Studies, Kyoto University, Kyoto, Japan
[3]Yasugi Clinic, Shimane, Japan
[4]Center for Regional & International Collaboration, Kochi University, Kochi, Japan
[5]Kitaakita Municipal Hospital, Akita, Japan
[6]Faculty of Health Science, Suzuka University of Medical Science, Mie, Japan
[7]Graduate School of Regional Innovation Studies, Mie University, Mie, Japan
[8]Faculty of Medicine, Kochi University, Kochi, Japan
[9]Hakubi Center for Advanced Research, Kyoto University, Kyoto, Japan
[10]Faculty of Medicine, Cenderawasih University, Papua, Indonesia
[11]Faculty of Public Health, Cenderawasih University, Papua, Indonesia

Correspondence to: Kiyohito Okumiya,
Research Institute for Humanity and Nature
Motoyama 457-4, Kamigamo, Kita-ku, Kyoto 603-8047, Japan
Tel: +81-75-707-2411, Fax: +81-75-707-2509
E-mail: okumiyak@chikyu.ac.jp

Abstract:286 words
Text:3532 words
4 tables and 3 figures

For peer review only

ABSTRACT

Objective: Few follow-up studies have been carried out on the incidence of amyotrophic lateral sclerosis (ALS) and parkinsonism in Papua, Indonesia since a survey undertaken from 1962 to 1980 by Gajdusek and colleagues. To clarify the clinical epidemiology of ALS and parkinsonism in the south coastal region of Papua, clinical characteristics and the prevalence of the diseases in this region were examined and assessed.

Methods: Cases of ALS and parkinsonism were clinically examined during a 2001–2012 survey in Bade and other villages along the Ia, Edera, Dumut, and Obaa rivers in Papua, Indonesia. Possible, probable, and definite ALS were diagnosed clinically by certified neurologists based on El Escorial criteria. The criteria for diagnosis of parkinsonism were the presence of at least two of the four signs of tremor, rigidity, bradykinesia, and postural impairment with a progressive course.

Results: During the survey, 46 cases of ALS and/or parkinsonism were diagnosed within a population range of 7000 (2001–2002) to 13,900 (2007–2012). The 46 cases consisted of 17 probable-definite cases of ALS, including three with cognitive impairment (CI), 13 cases of overlap of possible, probable, or definite ALS and parkinsonism, including five cases with CI, and 16 cases of parkinsonism, including one case with CI. Crude point prevalence rate of pure ALS was estimated at least 73-133/100,000 people and that of overlap of ALS and parkinsonism was at least 53-98/100,000 in 2007 or 2010 in some regions.

Conclusions: Though lower than the prevalence previously reported, the recent prevalence of ALS in this region of Papua is still higher than the global average. In particular, there was a high prevalence of overlap of ALS and parkinsonism, such as has

For peer review only - http://bmjopen.bmj.com/site/about/guidelines.xhtml

been previously reported in Guam and Kii.

Strengths and limitations of this study

・This study is unique in the epidemiological survey of neurodegenerative diseases during the period between 2001 and 2012 in Papua, Indonesia, one of the foci with the highest incidence of ALS in the world.

・As this was not a complete inventory survey, the actual prevalence rate may be a little higher than our estimation. In spite of its limitation the recent prevalence of ALS and overlap of ALS and parkinsonism in this region of Papua was still higher than the global average.

・This study was based only on clinical and not on neuropathological findings. In spite of its limitation the overlap of ALS and/or parkinsonism with cognitive impairment suggests that it may be the same disease entity as the ALS/PDC diagnosed in Guam and Kii.

For peer review only

For peer review only - http://bmjopen.bmj.com/site/about/guidelines.xhtml

INTRODUCTION

Between 1962 and 1980, Gajdusek et al. reported 97 cases of amyotrophic lateral sclerosis (ALS), 18 of parkinsonism, and 18 of poliomyeloradiculitis (PMR) in the south coastal area of Papua (former West New Guinea) within a population of 7,000.[1,2] Along with Guam and Kii in Japan, Papua (Indonesia) has been considered one of the foci with the highest incidence of ALS in the world.[1-7] The high incidence of ALS and parkinsonism-dementia complex (PDC) in Guam and Kii reportedly no longer exists.[8,9] In 1987, Spencer et al. reported only two cases of ALS and three of parkinsonism in the villages along the Ia river, in 1990, no cases of ALS and three of parkinsonism along the same river; thus decline or disappearance of ALS was suspected to have also occurred in Papua.[10] However, there have been few follow-up surveys on ALS and parkinsonism in Papua since 1991.

To clarify the clinical epidemiology of ALS and parkinsonism in the south coastal region of Papua, we carried out a survey of neurodegenerative diseases from 2001 to 2012.

METHODS

The survey sites between 2001 and 2002 (total population 7000) were the villages of Kepi (population 800), Emete (800), Ogoto (400), Senggo (2000), and Bade (3000).[11,12] From 2007 to 2012, the survey sites were the villages along four rivers (total population 13,900), namely Bade along the Digul river (3800 people), Mur along the Mappi river (1500), the eight villages along the Ia river (4500), and the 12 villages along the Edera and Dumut rivers (4100 people). The eight villages along the Ia river

For peer review only - http://bmjopen.bmj.com/site/about/guidelines.xhtml

were Gimikya (800), Geturki (500), Homlikya (700), Kobeta (600), Asset (600), Osso (300), Bosma (500), and Ogorito (500). The 12 villages along the Edera and Dumut rivers were Benggo (200), Kokoya (500), Sahapikya (400), Harapan (400), Yibin (300), Mopio (300), Pies (300), Memes (300), Habeske (300), Muya (300), Getio (200), and Sien (600) (Figure 1). At least two of six Japanese certified neurologists (KO, MF, YH, SK, YK, and KM) visited each village with a local neurologist (IM).

Among the many types of neurological and non-neurological cases encountered, diagnoses of ALS, parkinsonism, and cognitive impairment (CI) were based on clinical examinations and discussions among the participating neurologists. ALS was classified as “definite”, “probable”, “possible”, and “suspected” on the basis of combinations of upper motor neuron (UMN) and lower motor neuron (LMN) signs according to the El Escorial criteria of the World Federation of Neurology.[13] Probable and definite ALS are defined as pure ALS in this report. A diagnosis of parkinsonism required the presence of at least two of the four signs of tremor, rigidity, bradykinesia, and postural impairment with a progressive course without evidence/history of vascular accidents or history of taking drugs known to induce parkinsonism. The degree of disability of patients with parkinsonism was classified according to the scale of Hoehn and Yahr.[14] Overlap of possible, probable, or definite ALS with parkinsonism was defined as ALS–parkinsonism. Cognitive impairment was diagnosed when loss of memory or impairments of language usage, praxis, or executive functions were recognized during clinical examination and described by family members. Neurological cases with overlapping cerebellar signs and poliomyeloradiculitis (PMR)[1,2] were excluded from this study. In Bade and the villages along the Ia, Edera, and Dumut rivers, not only new

For peer review only

cases were identified but previously diagnosed cases were also followed up and assessed neurologically and documented in 2007, 2008, 2010, 2011, and 2012. Death years were confirmed, and the durations between subjective onset of illness and the last survey or death were recorded as durations of subjective illness.

The village leaders and medical staff members in each village's primary healthcare center agreed to take part in the survey, which was conducted with the permission of the Department of Health of the Papua Provincial Government in cooperation with Cenderawasih University. The purpose of the neurological examinations was explained to all of the villagers. All subjects volunteered to participate following the announcement of availability of neurological check-ups, which were performed in community health centers or homes in each field site. This survey was approved by the Ethics Committees of the Research Institute for Humanity and Nature in Japan and Cenderawasih University in Papua, Indonesia. Written informed consent was obtained from all study participants. The populations of Bade and the villages along the Ia, Edera, and Dumut rivers were obtained from a report by the public office of Edera district in 2006.[15] We were informed by village leaders of the populations of other villages in the survey during 2001–2002.

RESULTS

Clinical types of ALS and/or parkinsonism

We identified 46 cases with signs of ALS and/or parkinsonism including 17 cases with pure ALS (Table 1), 13 with ALS–parkinsonism (Table 2), and 16 with parkinsonism (Table 3). Relevant information about the cases including neurological signs, age at last

For peer review only - http://bmjopen.bmj.com/site/about/guidelines.xhtml

survey, age and year of onset, duration of disease, certified death year, and ethnicity are shown in Tables 1–3. The sex distribution was male/female: 10/7 (male 59%) for ALS, 11/2 (85%) for ALS–parkinsonism, and 8/8 (50%) for parkinsonism. The average age at last survey was 51.3 years for ALS, 52.9 years for ALS–parkinsonism, and 51.6 years for parkinsonism. The mean age of onset was 46.2 (11–68) years for pure ALS, 49.5 (11–70) years for ALS–parkinsonism, and 45.8 (27–64) years for parkinsonism. Mean duration of subjective illness was 7.0 (2–20) years for pure ALS, 6.3 (2–17) years for ALS–parkinsonism, and 6.3 (2–14) years for parkinsonism. We observed overlapping cognitive impairment (CI) in three of the 17 cases (18%) of pure ALS, five of the 13 cases (38%) of ALS–parkinsonism, and one of the 16 cases (6%) of parkinsonism.

During the follow-up surveys of the cases in the Bade, Ia, and Edera rivers, we observed one change from possible ALS to probable ALS (Case ALS10), and two from parkinsonism to ALS–parkinsonism (Cases ALS-P 2 and ALS-P 3). We also identified one instance of a shared family history of parkinsonism (P 6) and ALS–parkinsonism (ALS-P 1).

There were family histories of neurological disorders in seven of 17 cases (41%) of pure ALS, two of 13 cases (15%) of ALS–parkinsonism, and six of 16 cases (38%) of parkinsonism (Tables 1–3).

Cases of ALS and parkinsonism in Bade and villages along the Ia, Edera, and Dumut rivers between 2007 and 2012

Between 2007 and 2012, we identified four cases of pure ALS, two of ALS–parkinsonism, and one of parkinsonism in Bade (population 3800 people) and seven cases of pure ALS, four of ALS–parkinsonism, and three of parkinsonism in the villages

For peer review only

(population 4500 people) along the Ia river. Between 2008 and 2012, we identified three cases of pure ALS, five of ALS–parkinsonism, and six of parkinsonism in the villages (population 4100 people) along the Edera and Dumut rivers (Table 4).

In the 2007 survey, there were four cases of pure ALS suffered and alive (crude point prevalence rate 105/100,000 people), two of ALS–parkinsonism (53/100,000), and one (26/100,000) of parkinsonism in Bade. In 2010, there were six cases of pure ALS suffered and alive (133/100,000 people), four of ALS–parkinsonism (89/100,000), and three of parkinsonism (67/100,000) in the villages along the Ia river. In 2010, there were three cases of pure ALS suffered and alive (73/100,000 people), four of ALS–parkinsonism (98/100,000), and five of parkinsonism (122/100,000) in the villages along the Edera and Dumut rivers (Table 4).

Case reports

Case ALS 12

A 64-year-old (in 2012) man of Auyu ethnicity, a resident of Bosma along the Ia river, had a history of progressive gait disturbance and motor weakness for 9 years in 2010; these symptoms had progressed in the follow-ups of 2011 and 2012. Neurological examination revealed UMN and LMN signs including a positive Babinski sign, hyperreflexia with ankle clonus, muscle atrophy and weakness in the tongue and upper and lower limbs bilaterally, and fasciculation in the upper limbs and tongue. He had no cognitive impairment or sensory disturbance. We diagnosed him as having definite ALS (Figure 2-A,B,C,D). His father allegedly had a history of parkinsonism.

For peer review only - http://bmjopen.bmj.com/site/about/guidelines.xhtml

Case ALS-17

A 70-year-old (in 2012) man of Auyu ethnicity, a resident of Ogorito along the Ia river, had a history of progressive memory loss for 4 years and slow gait for 2 years. Neurological examination revealed UMN and LMN signs including a positive Babinski sign, hyperreflexia in the jaw jerk, bilateral upper and lower limbs, and muscle atrophy, weakness, and fasciculation in the distal upper and lower limbs bilaterally. He had fasciculation in the tongue and respiratory muscle weakness with a low oxygen saturation (SpO_2 86%). He also had memory disturbances accompanied by positive snout and palmomental reflexes and difficulty with mental activities of daily living. He had no sensory disturbance. We diagnosed him as having definite ALS with CI (Figure 3-A).

Case ALS-P 1

A 65-year-old (in 2008) woman of Auyu ethnicity, a resident of Pies along the Edera river, had a history of progressive motor weakness and gait disturbance for 5 years. She could not walk without support. Neurological examination revealed a resting tremor, cogwheel rigidity in the upper and lower limbs, bradykinesia throughout, postural impairment and UMN and LMN signs with a positive Babinski sign, hyperreflexia in the upper and lower limbs bilaterally, and thenar and hypothenar muscle atrophy. She had no cognitive impairment or sensory disturbance. We diagnosed her as having ALS–parkinsonism; more specifically, with severe parkinsonism (Hoen and Yahr IV) simultaneously complicated by motor neuron signs indicative of possible ALS. She died in 2009. Her younger sister was Case P 6 and her cousin allegedly had parkinsonism (Figure 3-B).

For peer review only - http://bmjopen.bmj.com/site/about/guidelines.xhtml

Case ALS-P 2

A 76-year-old (in 2011) man of Auyu ethnicity, a resident of Ogorito, had a history of tremor in both hands for 5 years in 2010. Neurological examination revealed cogwheel rigidity in his upper and lower limbs, bradykinesia, and UMN signs with hyperreflexia in bilateral upper and lower limbs. We diagnosed him as having parkinsonism (Hoen and Yahr II) in 2010. He was examined again in 2011, by which time his parkinsonism was complicated by not only postural impairment but also muscle weakness and atrophy, and spasticity of the upper and lower limbs with Babinski signs. He had no cognitive impairment or sensory disturbance. We diagnosed him as having parkinsonism (Hoen and Yahr III) plus possible ALS (parkinsonism was accompanied by ALS later). He died in 2011 of unknown causes.

We also diagnosed another case of ALS-P3 parkinsonism (Hoen and Yahr III) plus possible ALS (parkinsonism was accompanied by ALS later).

Case ALS-P6

A 50-year-old (in 2012) man of Auyu ethnicity, a resident of Benggo along the Edera river, had a history of dysarthria for 2 years. Neurological examination revealed UMN and LMN signs with a positive Babinski sign, muscle atrophy and weakness in the tongue, proximal upper limb, shoulder, and tibialis anterior muscles. He had cogwheel rigidity in the wrist and elbow, bradykinesia throughout, a positive Myerson sign and impairment of coordination and postural reflex. He had memory loss, bradyphrenia, slow speech, and positive snout and palmomental reflexes with difficulty with activities

For peer review only - http://bmjopen.bmj.com/site/about/guidelines.xhtml

For peer review only

of daily living. We diagnosed him as having definite ALS and parkinsonism (Hoen and Yahr III) with cognitive impairment (Figure 3-C).

Case P 7

A 52-year-old (in 2010) woman of Auyu ethnicity living in Mopio, along the Edera river, had a history of progressive gait disturbance with parkinsonian signs including resting tremor, cogwheel rigidity, anterior bending, and bradykinesia for 3 years in 2008. In 2008, we diagnosed her as having parkinsonism (Hoen and Yahr II). By the time of the follow-up survey in 2010, she could not stand by herself and had postural impairment with parkinsonian hands bilaterally. She had no UMN or LMN signs, no cognitive impairment, and no sensory disturbance. We diagnosed her as having Parkinsonism (Hoen and Yahr IV) (Figure 3-D).

DISCUSSION

In the present survey, we performed full neurological examinations on 46 subjects with ALS and/or parkinsonism from 2001 to 2012. Among them there were 17 probable-definite cases of pure ALS, 13 with overlapping ALS (possible, probable, or definite) and parkinsonism, and 16 of parkinsonism. Nine cases of ALS and/or parkinsonism had the complication of cognitive impairment. During the study period, some patients' diagnoses changed from pure parkinsonism to ALS–parkinsonism and their disease progressed in severity. Some subjects had both ALS and parkinsonism on their first examination. It is thus evident that overlapping ALS and parkinsonism and/or cognitive impairment still exist in the specified region of Papua.

For peer review only - http://bmjopen.bmj.com/site/about/guidelines.xhtml

Gajdusek et al. reported diagnosing 97 cases of ALS during the six surveys they conducted from 1962 to 1980. [1] However, they reported the neurological findings of only 32 of these cases, in only 16 of which the reported findings of UMN, LMN, and parkinsonian signs justify a diagnosis of probable or definite ALS. These 16 comprise 12 cases of pure ALS and four of overlapping ALS–parkinsonism. Among the 32 ALS cases they diagnosed, three had a positive family history (9%): two had sisters with ALS and one subject with ALS–parkinsonism had a family history of ALS. None of the ALS cases had a family history of parkinsonism. These authors also reported their neurological findings in 13 subjects in whom they diagnosed parkinsonism. They reported their findings on full neurological examinations of 10 of these parkinsonism cases: according to their reported neurological signs, these comprised eight cases of parkinsonism, including five with dementia, and two of overlapping ALS–parkinsonism with dementia.[1] Among the 13 parkinsonism cases, three had a positive family history (23%). In summary, we believe that Gajdusek et al.'s 26 cases whose full neurological examinations they reported comprised 12 of pure ALS (probable or definite), six of overlapping ALS–parkinsonism, and eight of parkinsonism with/without dementia. We have here reported 17 cases of pure ALS (probable or definite), 13 of overlapping ALS–parkinsonism, and 16 of parkinsonism with/without dementia in the 46 cases we examined fully neurologically. Thus, we identified many cases of overlapping ALS and parkinsonism (n = 13, 28%) as well as the previous report (n = 6, 23%).[1]

In 1975, Gajdusek et al. reported 13 cases of ALS (crude point prevalence rate, 650/100,000 people) and three of parkinsonism (150/100,000) in the villages along the Ia river (population 2000). [1] We here compare the prevalence of probable-definite

For peer review only - http://bmjopen.bmj.com/site/about/guidelines.xhtml

ALS and parkinsonism according to Gajdusek's report and our more current findings. In our survey of 2010 (population: 4500), we identified six probable-definite cases of ALS in this region (ALS 8, 10–13, 17) (133/100,000) and seven of parkinsonism (possible ALS-P 2, 3, 9, and 13 and P 10, 15, and 16) (156/100,000). In 1975, Gajdusek et al. reported eight cases of ALS (410/100,000) and six of parkinsonism (308/100,000) in the villages along the Edera and Dumut rivers (population 1950). In our survey of this region in 2010 (population 4100), we identified five probable-definite cases of ALS (ALS 9, 14, and 16 and ALS-P 5, 6) (122/100,000 people) and seven of parkinsonism (possible ALS-P 10, and 11 and P 7, 8, and 11–13) (171/100,000) (Table 4). As this was not a complete inventory survey, the actual prevalence rate may be a little higher than our estimation. Thus our findings suggest that the prevalence of ALS may have decreased during the 35 years since Gajdusek et al.'s report. However, it is still much higher than the global average (incidence: approximately 2/100,000; prevalence: about 6/100,000)[16-21] and, in particular, the prevalence of overlapping ALS and parkinsonism is especially high in Papua.

Overlap of ALS–parkinsonism and dementia has also been reported in patients with ALS/PDC in Guam and Kii, Japan.[4,22-28] Hirano et al. provided clinicopathological evidence of 47 cases of PDC in Guam in 1961 comprising the following three groups: (1) 30 cases of PDC; (2) eight of parkinsonism with dementia with clinical evidence of UMN impairment; and (3) nine of parkinsonism with dementia with clinical evidence of UMN and LMN impairment. At least 15 of these cases (31.9%) had a family history of ALS, parkinsonism, or both.[4] Kuzuhara and Kokubo et al. surveyed neurological diseases in Kii, Japan in the 1990s and found not only a continuously high incidence of

For peer review only - http://bmjopen.bmj.com/site/about/guidelines.xhtml
For peer review only

ALS, but also neuropathologically verified cases of PDC. ALS and PDC frequently affected the same individuals simultaneously, as well as affecting members of the same family. These authors reported 37 cases of ALS/PDC from 1996 to 2006, a high proportion of whom (78%) had a positive family history, as well as clinical examples of pure ALS, PDC, and overlapping ALS and PDC. They also reported neuropathological changes common to cases of ALS and PDC in 12 cases of ALS/PDC, resembling those found in patients with ALS/PDC on Guam.[23-28] In our more recent study in Papua, like the previous studies in Kii, Japan, and Guam, we identified many cases of pure ALS and ALS overlapping with parkinsonism with/without cognitive impairment. The prevalence of cognitive impairment was lower in our study than in previous studies.[1,26] Cognitive impairment may have been underestimated by our neurological examinations in the field: our study was based only on clinical and not on neuropathological findings. However, we did identify many patients with overlapping ALS–parkinsonism in this study. The overlap of ALS and/or parkinsonism with CI suggests that it may be the same disease entity as the ALS/PDC diagnosed in Guam and Kii.

Various possible causal genetic and environmental factors for these conditions have been reported. The environmental factors of low concentrations of calcium and magnesium in drinking water and cycad neurotoxins have been suggested to be underlying causes of these diseases, but these links are so far unproven.[1,2,10,15,29-34]

Genetic abnormalities that had been previously established associated with familial or

For peer review only - http://bmjopen.bmj.com/site/about/guidelines.xhtml

sporadic ALS, dementia, familial Parkinson's disease and parkinsonism were looked for in the cases in Kii, but none of them were identified.[23,35] Hermousura et al. have discovered a nuclear mutation in the calcium/magnesium membrane ion channel transient receptor potential melastatin 7 (TRPM7) gene in a subset of patients with ALS/PDC in Guam.[36] They reported that a TRPM7 variant that is associated with altered sensitivity to magnesium may have contributed to the pathogenesis of ALS and PDC in two Guamanians. TRPM7 was not found to be associated with ALS/PDC in the Kii peninsula of Japan.[37] Ishiura et al. have recently discovered *C9ORF72* repeat expansion in some cases of ALS in the Kii peninsula of Japan: this repeat expansion partly accounts for the high prevalence of ALS in this region.[38] However, the responsible gene(s) remain unproved in almost all cases of ALS/PDC in both Guam and Kii. Other possible causal factors of ALS and ALS–parkinsonism in Papua, Guam, or Kii include associations with prion disease or epigenetics.

In Guam, although the incidence of pure ALS has declined markedly, the prevalence of PDC is still high.[39] The prevalence of dementia among elderly Chamorros (earliest known inhabitants of the Mariana Islands, which include Guam) is also reportedly relatively high.[40] In addition, in Kii the incidence of pure ALS has declined markedly, and the prevalence of PDC and ALS/PDC or ALS with dementia recently increased.[25,28] These changing patterns of ALS/PDC incidence in Guam and Kii might be caused by changes in environmental and socioeconomic factors as well as aging.[40,41]

In our recent survey in Papua, the prevalence of pure ALS in the south coastal area in

For peer review only

For peer review only - http://bmjopen.bmj.com/site/about/guidelines.xhtml

Papua appeared to be lower than in a previous study [1]; however, we still identified many cases of overlap of ALS and parkinsonism. The mean age of onset in our cases was 46 years for ALS, 53 years for ALS–parkinsonism, and 46 years for parkinsonism. Thus, they were older than the previously reported cases of ALS (33 years) and parkinsonism (43 years).[1] In our cases, the mean duration of subjective illness was 7.0 years for ALS, 6.3 years for ALS–parkinsonism, and 6.3 years for parkinsonism; these are longer than the previously reported 3.5 years for ALS and 6.0 years for parkinsonism.[1] The mean age of onset may recently have increased and the mean disease duration become longer in subjects with ALS.

Indigenous people of Papua still retain aspects of their traditional lifestyles, such as hunting, gathering, fishing, and eating sago. They also drink water from shallow wells. However, with the spread of a market economy, recent increases in numbers of rubber plantations and progress in transportation have been causing lifestyle changes in the villages in the south coastal area of Papua. Ongoing follow-up surveys should be performed to evaluate change in clinical types of ALS and/or parkinsonism with or without cognitive impairment accompanying changes in environmental and socioeconomic factors like, as have occurred in Guam and Kii.

In conclusion, the crude prevalence of ALS in the south coastal area of Papua may be lower than previously reported, but there are still many cases of pure ALS and overlapping ALS–parkinsonism and cognitive impairment as has previously been reported in Guam and Kii. Further ongoing follow-up surveys focusing on the clinical epidemiology and etiology of these neuro-degenerative diseases in association with

For peer review only - http://bmjopen.bmj.com/site/about/guidelines.xhtml

ecological and environmental factors in Papua are needed. Future studies, including neuropathological examinations, should be performed when the local villagers in this region of Papua, Indonesia are willing to consent to autopsies.

ACKNOWLEDGMENTS

The authors wish to thank all the participants in the villages who volunteered for the survey, the village leaders, and Mappi Regency officials. We are deeply indebted to doctors Imelda Mandagi, Reginald Nangoy, and Christha Z. Tamburian and their staff at Puskesmas Bade, and to Arifin Wasaraka, Lazarus Revasy, Yosefina Griapon, Arius Togodly, Kerry Yarangga, and Dolfinus Y. Bouway at Cenderawasih University for their invaluable contributions to the study. Andre Liem and Herry Wondiwoy at Papua Tour Guides Community and S.E. Amos at Bade High School provided logistic and language assistance. On the Japanese side, we acknowledge the technical support and advice of Katsuyuki Eguchi (Nagasaki University), Yasushi Osaki (Kochi University), Hidekazu Tomimoto (Mie University), Taro Yamauchi (The University of Tokyo), Hideyuki Onishi (Doshisha Women's College), Tetsuya Inamura (Aichi Prefectural University), Wenling Chen, Yumi Kimura, Yasuko Ishimoto, and Hissei Imai (Kyoto University), and Yasuyuki Kosaka, Yukiko Kita, and Chizu Wada (Research Institute for Humanity and Nature).

AUTHOR CONTRIBUTIONS

For peer review only

Okumiya had full access to all the data in the study and takes responsibility for the integrity of the data and the accuracy of the data analysis.

Study concept and design: Okumiya, Matsubayashi.

Acquisition of data: Okumiya, Wada, Fujisawa, Ishine, Garcia del Saz Hirata, Kuzuhara, Kokubo, Seguchi, Sakamoto, Manuaba, Matsubayashi.

Analysis and interpretation of data: Okumiya,Wada, Sakamoto, Fujisawa, Matusbayashi.

Drafting of the manuscript: Okumiya,Wada, Sakamoto, Fujisawa, Matusbayashi.

Critical revision of the manuscript for important intellectual content: All authors.

Statistical analysis: Okumiya.

Obtained funding: Okumiya, Wada, Kuzuhara, Kokubo, Matsubayashi

Administrative, technical, or material support: Okumiya, Watofa, Rantetampang, Matsubayashi.

Study supervision: Okumiya, Matsubayashi.

COMPETING INTERESTS : None

FUNDING

K. Okumiya receives research support from Grant-in-Aid for Scientific Research (A) from the Japan Society for the Promotion of Science (JSPS). K. Matsubayashi receives research support from Grant-in-Aid for Scientific Research (A) from the JSPS and is an editor of the journal *Geriatrics & Gerontology International*. T. Wada receives research support from Grant-in-Aid for Scientific Research (C) from the JSPS. Y. Kokubo and S.

For peer review only - http://bmjopen.bmj.com/site/about/guidelines.xhtml

Kuzuhara receive support from Grants-in-Aid from the Research Committee of CNS Degenerative Diseases and the Research Committee on the Muro disease (Kii amyotrophic lateral sclerosis/Parkinsonism-dementia), the Ministry of Health, Labour and Welfare of Japan.

For peer review only

REFERENCES

1. Gajdusek DC, Salazar AM. Amyotrophic lateral sclerosis and parkinsonian syndromes in high incidence among the Auyu and Jakai people of West New Guinea. Neurology 1982;**32**:107–26.
2. Gajdusek DC. Foci of amyotrophic lateral sclerosis in high incidence in isolated populations of East Asia and the Western Pacific. In: Lewis P Rowland, ed. Human Amyotrophic Lateral Sclerosis. New York: Raven Press 1982:363–93.
3. Kurland LT, Mulder DW. Epidemiologic investigations of amyotrophic lateral sclerosis. 1. Preliminary report on geographic distribution and special reference to the Mariana Islands, including clinical and pathologic observations. Neurology 1954;**4**:438–48.
4. Hirano A, Kurland LT, Krooth RS, et al. Parkinsonism-dementia complex, an endemic disease on the island of Guam. I. Clinical features. Brain 1961;**84**:642–61.
5. Hirano A, Malamud N, Kurland LT. Parkinsonism-dementia complex, an endemic disease on the island of Guam. II. Pathological features. Brain 1961;**84**:662–79.
6. Kimura K, Yase Y, Higashi Y, et al. Epidemiological and geomedical studies on ALS and allied diseases in Kii peninsula (Japan): preliminary report. Proc Jpn Acad 1961;**37**:417–20.
7. Shiraki H, Yase Y. Amyotrophic lateral sclerosis in Japan. In: Vinken PJ, Bruyn GW, eds. Handbook of clinical neurology Vol. 22, System disorders and atrophies, Part II. Amsterdam: Elsevier Science 1975:353–419.
8. Garruto RM, Yanagihara R, Gajdusek DC. Disappearance of high incidence ALS and PDC on Guam. Neurology 1985;**35**:193–8.
9. Yoshida S. ALS tahatsuchi niokeru kankyoyoin to aluminium no neuron hensei eno

For peer review only

For peer review only - http://bmjopen.bmj.com/site/about/guidelines.xhtml

kanyo nitsuite.[Environmental factors in western Pacific foci of ALS and a possible pathogenetic role of aluminum (Al) in motor neuron degeneration] (in Japanese with English abstract). Rinsho Shinkeigaku [Clin Neurol] 1991;**31**:1310–2.

10. Spencer P, Palmer VS, Ludolph AC. On the decline and etiology of high-incidence motor system disease in West Papua (Southwest New Guinea). Mov Disord 2005;**20(**Suppl 12):S119–126.
11. Wada T, Matsubayashi K, Okumiya K, et al. Health status and subjective economic satisfaction in West Papua. Lancet 2002;**360**:951.
12. Okumiya K, Fujisawa M, Ishine M, et al. Nishi New Guinea chiiki (Indonesia/ Papua shu) no shinkeihensei shikkan no jittai. 2001-2002 nen, 2006-2007 nen no fieldwork yori. [Fieldwork survey of neurodegenerative diseases in West New-Guinea in 2001-02 and 2006-07] (in Japanese). Rinsho Shinkeigaku [Clin Neurol] 2007;**47**:977–8.
13. Brooks BR. El Escorial World Federation of Neurology criteria for the diagnosis of amyotrophic lateral sclerosis: subcommittee on Motor Neuron Diseases/Amyotrophic Lateral Sclerosis of the World Federation of Neurology Research Group on Neuromuscular Diseases and the El Escorial "Clinical Limits of ALS" Workshop Contributors. J Neurol Sci 1994;**124**:96–107.
14. Hoehn MM, Yahr MD. Parkinsonism: onset, progression and mortality. Neurology. 1967;**17**:427–42.
15. Kontor Statistics Distrik Edera. Laporan Kependudukan, Kabupaten Mappi Distrik Edera 2006 (December).
16. Armon C. Amyotrophic lateral sclerosis. In: Neuroepidemiology from principles to practice. USA: Oxford University Press 2004:162–87.

For peer review only - http://bmjopen.bmj.com/site/about/guidelines.xhtml

For peer review only

17. McGuire V, Longstreth WT Jr, Koepsell TD, et al. Incidence of amyotrophic lateral sclerosis in three counties in western Washington state. Neurology 1996 ;**47**:571–3.

18. Sorenson EJ, Stalker AP, Kurland LT, et al. Amyotrophic lateral sclerosis in Olmsted County, Minnesota, 1925 to 1998. Neurology 2002; **23**:280–2.

19. O'Toole O, Traynor BJ, Brennan P, et al. Epidemiology and clinical features of amyotrophic lateral sclerosis in Ireland between 1995 and 2004. J Neurol Neurosurg Psychiatry 2008;**79**:30–2.

20. Chiò A, Mora G, Calvo A, et al. Epidemiology of ALS in Italy: a 10-year prospective population-based study. Neurology 2009;**72**:725–31.

21. Factor-Litvak P, Al-Chalabi A, Ascherio A, et al. Current pathways for epidemiological research in amyotrophic lateral sclerosis. Amyotroph Lateral Scler Frontotemporal Degener 2013;**14**(Suppl 1):33–43.

22. Rodgers-Johnson P, Garruto RM, Yanigahara R, et al. Amyotrophic lateral sclerosis and parkinsonism-dementia on Guam: a 30-year evaluation of clinical and neurological trends. *Neurology.* 1986;36:7-13.

23. Kuzuhara S, Kokubo Y, Sasaki R, et al. Familial amyotrophic lateral sclerosis and parkinsonism-dementia complex of the Kii peninsula of Japan: clinical and neuropathological study and tau analysis. Ann Neurol 2001;**49**:501–11.

24. Kuzuhara S, Kokubo Y. Atypical parkinsonism of Japan: amyotrophic lateral sclerosis-parkinsonism-dementia complex of the Kii peninsula of Japan (Muro disease): an update. Mov Disord 2005;**20**(Suppl 12):S108–13.

25. Kuzuhara S. Kii ALS/PDC (Murou byou) no rekishi, gainen, ekigaku. [ALS-parkinsonism-dementia complex of the Kii peninsula of Japan (Muro disease). Historical review, epidemiology and concept] (in Japanese with English abstract).

For peer review only - http://bmjopen.bmj.com/site/about/guidelines.xhtml

Rinsho Shinkeigaku [Clin Neurol] 2007;**47**:962–5.

26. Kokubo Y. Kiihanto no kinishukuseisokusakukokasho/ parkinson chihou hukugo no rinshou, shinkei byourigakuteki kenkyu. [Clinical aspects, imaging and neuropathology of Kii ALS/PDC] (in Japanese with English abstract). Rinsho Shinkeigaku [Clin Neurol] 2007;**47**:966–9.
27. Kuzuhara S. Kii hanto no Murobyo (Kinishukuseisokusakukokasho/ parkinson/ ninchisho hukugo) [Muro disease or ALS–parkinsonism–dementia complex of the Kii peninsula of Japan] (in Japanese with English abstract). Rinsho Shinkeigaku [Clin Neurol] 2007;**47**:695–702.
28. Kuzuhara S, KokuboY. Amyotrophic lateral sclerosis-parkinsonism-dementia complex in the Kii peninsula of Japan (Muro disease): a review on recent research and new concept. In: Michael J. Strong, ed. Amyotrophic lateral sclerosis and the frontotemporal dementias. Oxford: Oxford University Press 2012:39–54.
29. Yase Y. The pathogenesis of amyotrophic lateral sclerosis. Lancet 1972;**2**(7772):292–6.
30. Garruto RM, Yanagihara R. Contributions of isolated Pacific populations to understanding neurodegenerative diseases. Folia Neuropathol 2009;**47**(2):149–70.
31. Spencer PS, Nunn PB, Hugon J, et al. Guam amyotrophic lateral sclerosis-parkinsonism-dementia linked to a plant excitant neurotoxin. Science 1987;**237**(4814):517–22.
32. Spencer PS, Ohta M, Palmer VS. Cycad use and motor neuron disease in Kii peninsula of Japan. Lancet 1987;**2**(8573):1462–3.
33. Cox PA, Sacks OW. Cycad neurotoxins, consumption of flying foxes, and ALS-PDC disease in Guam. Neurology 2002;**58**(6):956–9.

For peer review only - http://bmjopen.bmj.com/site/about/guidelines.xhtml

34. Banack SA, Cox PA. Biomagnification of cycad neurotoxins in flying foxes: implications for ALS-PDC in Guam. Neurology 2003;**61**:387–9.

35. Tomiyama H, Kokubo Y, Sasaki R, et al. Mutation analyses in amyotrophic lateral sclerosis/parkinsonism-dementia complex of the Kii peninsula, Japan. Mov Disord 2008;**23**(16):2344–8.

36. Hermosura MC, Nayakanti H, Dorovkov MV, et al. A TRPM7 variant shows altered sensitivity to magnesium that may contribute to the pathogenesis of two Guamanian neurodegenerative disorders. Proc Natl Acad Sci 2005;**102**(32):11510–5.

37. Hara K, Kokubo Y, Ishiura H, et al. TRPM7 is not associated with amyotrophic lateral sclerosis-parkinsonism dementia complex in the Kii peninsula of Japan. Am J Med Genet B Neuropsychiatr Genet 2010;**153B**(1):310–3.

38. Ishiura H, Takahashi Y, Mitsui J, et al. *C9ORF72* Repeat expansion in amyotrophic lateral sclerosis in the Kii peninsula of Japan. Arch Neurol 2012;**69**(9):1154–8.

39. Plato CC, Garruto RM, Galasko D, et al. Amyotrophic lateral sclerosis and parkinsonism-dementia complex of Guam: changing incidence rates during the past 60 years. Am J Epidemiol 2003;**157**:149–57.

40. Galasko D, Salmon D, Gamst A, et al. Prevalence of dementia in Chamorros on Guam: relationship to age, gender, education, and APOE. Neurology 2007;**68**:1772–81.

41. Plato CC, Galasko D, Garruto RM, et al. ALS and PDC of Guam: forty-year follow-up. Neurology 2002;**58**:765–73.

For peer review only

For peer review only - http://bmjopen.bmj.com/site/about/guidelines.xhtml

Figure and table legends

Table 1. Relevant characteristics and neurological signs of cases with ALS with/without cognitive impairment in Papua, 2001–2012.

Abbreviations: ALS = amyotrophic lateral sclerosis; BK = bradykinesia; BS = Babinski sign; DTR = deep tendon reflex; Dys = dysphagia/dysarthria; F= female; Fasc = fasciculation; LMN = lower motor neuron signs; M = male; MA = muscle atrophy with weakness; NC = not confirmed by examination; P = parkinsonism; PI = postural impairment; R = rigidity; T = tremor; TA = tongue atrophy; U/L: upper limbs/lower limbs; UMN = upper motor neuron signs.

Age: at the time of final survey; Duration: between subjective onset of illness and last survey or death; Alive: alive in 2012; Onset: subjective onset of the illness; Deep tendon reflexes, -: absent, 1: hypoactive, 2: normal, 3: hyperactive, 4: markedly hyperactive with ankle clonus.

Other signs are classified as: -: absent; ±: questionably present; +: present; ++: markedly present.

Family history 1: cousin (possible ALS); 2: grandfather (NC); 3: sister (ALS15); 4: cousin's daughter (P16); 5: cousin (NC); 6: father (NC); 7: sister (ALS 7); 8: sister (P 6) and cousin (NC); 9: father (NC); 10: son: (NC); 11: sister (ALS-P 1) and cousin (NC); 12: mother and father (NC); 13: grandmother (NC); 14: mother (NC); 15: ALS 10 (mother's cousin).

Table 2. Relevant characteristics and neurological signs in cases with overlap of

For peer review only - http://bmjopen.bmj.com/site/about/guidelines.xhtml

ALS and parkinsonism with/without cognitive impairment in Papua, 2001–2012.

Table 3. Relevant characteristics and neurological signs in cases with parkinsonism with/without cognitive impairment in Papua, 2001–2012.

Table 4. Cases of ALS and parkinsonism according to field site

*; Cases suffered and alive in 2007 in Bade

#; Cases suffered and alive in 2010 in the villages along the Ia, Edera, and Dumut rivers

Figure 1. Map of the field sites

A. Large-scale map of the southern coastal plain in Papua, Indonesia.

B. Smaller scale map of the southern coastal plain in Papua, Indonesia showing rivers.

C. Detailed map showing villages in the southern coastal area in Papua in which all confirmed cases of ALS and/or parkinsonism with/without cognitive impairment were seen from 2001 to 2012.

Figure 2. Photographs of patient with ALS (Case ALS 12**)**

A: The patient needs assistance from family members to stand.

B: Advanced atrophy of the tongue.

C: There is upper limb-girdle and truncal muscle atrophy with a positive Babinski sign.

D: Advanced thenar muscle atrophy.

Figure 3. Photographs of patients with ALS, ALS-parkinsonism, and parkinsonism

A: Patient with ALS (Case ALS-17) showing muscle atrophy in the distal upper and

For peer review only - http://bmjopen.bmj.com/site/about/guidelines.xhtml

lower limbs.

B: Patient with ALS-parkinsonism (Case ALS-P 1) showing muscle atrophy in distal upper limbs.

C: Patient with ALS-parkinsonism (Case ALS-P 7) showing atrophy in muscles in the proximal upper limb, shoulder, and tibialis anterior.

D: Patient with parkinsonism (Case P 7) showing typical parkinsonian hands and anterior bending posture.

For peer review only

Okumiya

Table 1. Relevant characteristics and neurological signs in cases with pure ALS with/without cognitive impairment in Papua, 2001–2012.

Case number	ALS criteria Yahr stage	Survey year	Village	Age	Sex	Onset (age/year)	Duration (years)	Death (year)	Tribe (origin)	UMN signs		LMN signs		Bulbar signs		Parkinsonian sign				Cognitive impairment	Family history
										DTR U/L	BS	MA U/L	Fasc U/L	TA	Dys	T U/L	R U/L	BK	PI		
Pure ALS (n=17)																					
ALS 1	Definite	2001	Bade	35	M	NC	NC	NC	Auyu	3/3	+	++/++	+/+	++	-	-	-	-	-	-	-
ALS 2	Definite	2001	Ogoto	43	F	NC	NC	2002	Auyu	3/3	+	++/++	+/+	++	++	-	-	-	-	-	-
ALS 3	Definite	2002	Senggo	50	M	48/'00	2	NC	Citak	3/3	+	++/++	±/±	±	+	-	-	-	-	-	1
ALS 4	Definite	2007	Homilikia	53	M	51/'05	2	2007	Auyu	3/2	+	++/++	+/+	-	-	-	-	-	-	-	-
ALS 5	Definite	2007/'08	Bade	51	M	49/'06	3	2009	Auyu	3/4	+	+/-	-/-	-	+	-	-	-	-	-	-
ALS 6	Definite	2008	Bade	21	M	11/'98	11	2009	Auyu	4/4	+	++/++	-/-	-	-	-	-	-	-	-	2
ALS 7	Definite	2007/'08/'10	Bade	70	F	57/'97	13	2010	Maluku	3/3	+	++/++	+/-	-	+	-	-	-	-	-	3
ALS 8	Probable	2011	Geturki	53	M	48/'06	5	Alive	Auyu	3/3	-	+/+	-/+	-	-	-	-	-	-	-	-
ALS 9	Definite	2011	Muya	41	F	36/'06	6	2012	Auyu	3/3	+	+/+	-/-	-	-	-	-	-	-	-	-
ALS 10	Probable	2007/'10/'11/'12	Osso	65	F	59/'06	6	Alive	Auyu	3/4	+	+/-	-/-	+	-	-	-	-	-	-	4
ALS 11	Probable	2007/'08/'10/'12	Gimikya	32	M	12/'92	20	Alive	Auyu	4/4	+	++/+	++/-	±	-	-	+/-	-	-	-	5
ALS 12	Definite	2010/'11/'12	Bosma	64	M	53/'01	11	Alive	Auyu	3/4	+	++/++	+/-	++	-	-	-	-	-	-	6
ALS 13	Definite	2012	Asset	70	F	68/'10	2	Alive	Auyu	3/3	-	+/+	-/-	-	-	-	-	-	-	-	-
ALS 14	Probable	2012	Pies	65	F	60/'07	5	Alive	Auyu	3/4	-	+/+	-/-	-	-	-	-	-	-	-	-
ALS 15	Definite	2007	Bade	64	F	62/'05	2	2007	Maluku	3/3	+	++/++	-/-	-	+	-	-	-	-	+	7
ALS 16	Definite	2008/'10/'11	Yibin	25	M	13/'99	13	2012	Auyu	3/4	+	++/+	++/++	-	-	-	-	-	-	+	-
ALS 17	Definite	2012	Ogorito	70	M	66/'08	4	Alive	Auyu	3/3	+	+/+	+/+	-	-	-	-	-	-	+	-

Abbreviations: ALS = amyotrophic lateral sclerosis; BK = bradykinesia; BS = Babinski sign; DTR = deep tendon reflex; Dys = dysphagia/dysarthria; F = female; Fasc = fasciculation; LMN = lower motor neuron signs; M = male; MA = muscle atrophy with weakness; NC = not confirmed by examination; P = parkinsonism; PI = postural impairment; R = rigidity; T = tremor; TA = tongue atrophy; U/L: upper limbs/lower limbs; UMN = upper motor neuron signs.
Age: at the time of final survey; Duration: between subjective onset of illness and last survey or death; Alive: alive in 2012; Onset: subjective onset of illness; Deep tendon reflexes, -: absent, 1: hypoactive, 2: normal, 3: hyperactive, 4: markedly hyperactive with ankle clonus.
Other signs are classified as: -: absent; ±: questionably present; +: present; ++: markedly present.
Family history 1: cousin (possible ALS); 2: grandfather (NC); 3: sister (ALS15); 4: cousin's daughter (P16); 5: cousin (NC); 6: father (NC); 7: sister (ALS 7); 8: sister (P 6) and cousin (NC); 9: father (NC); 10: son: (NC); 11: sister (ALS-P 1) and cousin (NC); 12: mother and father (NC); 13: grandmother (NC); 14: mother (NC); 15: ALS 10 (mother's cousin).

For peer review only

Okumiya

Table 2. Relevant characteristics and neurological signs in cases with overlap of ALS and parkinsonism with/without cognitive impairment in Papua, 2001–2012.

Case number	ALS criteria Yahr stage	Survey year	Village	Age	Sex	Onset (age/year)	Duration (year)	Death (year)	Tribe (origin)	UMN signs		LMN signs		Bulbar signs		Parkinsonian sign				Cognitive impairment	Family history
										DTR	BS	MA	Fasc	TA	Dys	T	R	BK	PI		
										U/L		U/L	U/L			U/L	U/L				
ALS-parkinsonism (n = 13)																					
ALS-P 1	Possible-IV	2008	Pies	65	F	60/'03	6	2009	Auyu	3/3	+	+/-	-/-	-	-	+/+	++/++	++	+	-	8
ALS-P 2	Possible-III	2010/'11	Ogorito	76	M	70/'05	6	2011	Auyu	3/3	+	+/±	-/-	-	-	+/+	+/+	+	+	-	-
ALS-P 3	Possible-III	2010/'11/'12	Homilikia	69	M	65/'08	4	Alive	Auyu	3/2	+	+/-	-/-	-	-	+/+	+/+	+	+	-	-
ALS-P 4	Definite-III	2008	Bade	34	M	18/'92	17	2009	Muyu	3/3	+	+/-	-/-	++	++	-	+/+	+	+	+	9
ALS-P 5	Definite-III	2012	Sien	60	M	58/'10	2	Alive	Auyu	3/3	+	+/+	-/-	-	-	-	+/+	+	+	+	-
ALS-P 6	Definite-III	2012	Benggo	50	M	48/'10	2	Alive	Auyu	2/2	+	+/+	-/-	+	+	-	+/-	+	+	+	-
ALS-P 7	Possible-II	2001	Bade	36	M	NC	NC	NC	Auyu	3/3	+	-/-	-/-	-	-	+	+	+	-	-	-
ALS-P 8	Possible-II	2008/'10	Bade	23	M	11/'98	12	Alive	Auyu	3/4	+	-/-	-/-	-	-	+/+	+/±	+	-	-	-
ALS-P 9	Possible-III	2010/'11	Ogorito	76	M	70/'05	6	2011	Auyu	3/3	+	-/-	-/-	-	-	+/-	+/+	+	+	-	-
ALS-P 10	Possible-III	2008/'10/'11	Yibin	52	M	44/'03	8	Alive	Auyu	3/3	+	-/-	-/-	-	-	+/-	+/+	+	+	-	-
ALS-P 11	Possible-III	2011	Getio	55	F	53/09	2	2011	Auyu	3/3	+	±/-	-/-	-	-	+/+	+/+	+	+	-	-
ALS-P 12	Possible-IV	2001	Bade	40	M	NC	NC	NC	Auyu	3/3	+	-/-	-/-	-	+	+	+	+	+	+	-
ALS-P 13	Possible-III	2012	Bosma	52	M	48/'08	4	Alive	Auyu	3/4	+	-/-	-/-	-	-	-	++/+	+	+	+	-

Abbreviations: ALS = amyotrophic lateral sclerosis; BK = bradykinesia; BS = Babinski sign; DTR = deep tendon reflex; Dys = dysphagia/dysarthria; F = female; Fasc = fasciculation; LMN = lower motor neuron signs; M = male; MA = muscle atrophy with weakness; NC = not confirmed by examination; P = parkinsonism; PI = postural impairment; R = rigidity; T = tremor; TA = tongue atrophy; U/L: upper limbs/lower limbs; UMN = upper motor neuron signs.
Age: at the time of final survey; Duration: between subjective onset of illness and last survey or death; Alive: alive in 2012; Onset: subjective onset of illness; Deep tendon reflexes, -: absent, 1: hypoactive, 2: normal, 3: hyperactive, 4: markedly hyperactive with ankle clonus.
Other signs are classified as: -: absent; ±: questionably present; +: present; ++: markedly present.
Family history 1: cousin (possible ALS); 2: grandfather (NC); 3: sister (ALS15); 4: cousin's daughter (P16); 5: cousin (NC); 6: father (NC); 7: sister (ALS 7); 8: sister (P 6) and cousin (NC); 9: father (NC); 10: son: (NC); 11: sister (ALS-P 1) and cousin (NC); 12: mother and father (NC); 13: grandmother (NC); 14: mother (NC); 15: ALS 10 (mother's cousin).

For peer review only - http://bmjopen.bmj.com/site/about/guidelines.xhtml

Okumiya

Table 3. Relevant characteristics and neurological signs in cases with parkinsonism with/without cognitive impairment in Papua, 2001–2012.

Case number	ALS criteria Yahr stage	Survey year	Village	Age	Sex	Onset (age/year)	Duration (years.)	Death (year)	Ethnicity	UMN signs		LMN signs		Bulbar signs		Parkinsonian sign				Cognitive impairment	Family history
										DTR U/L	BS	MA U/L	Fasc U/L	TA	Dys	T U/L	R U/L	BK	PI		
Parkinsonism (n = 16)																					
P 1	Yahr IV	2001	Kepi	50	M	NC	NC	NC	Jakai	2/2	-	-/-	-/-	-	-	+/+	+/+	++	+	-	-
P 2	Yahr II	2001	Kepi	50	M	NC	NC	NC	Jakai	2/2	-	-/-	-/-	-	-	+	+	+	-	-	-
P 3	Yahr IV	2002	Emete	52	M	49/'99	3	NC	Jakai	2/2	-	-/-	-/-	-	-	+	+	+	+	-	-
P 4	Yahr II	2002	Senggo	41	M	36/'97	5	NC	Citak	2/2	-	-/-	-/-	-	-	+	+	-	-	-	-
P 5	Yahr I	2007	Mur	65	F	61/'03	4	NC	Jakai	2/3	-	-/-	-/-	-	-	+/-	+/-	-	-	-	10
P 6	Yahr IV	2008	Pies	60	F	55/'03	6	2009	Auyu	2/2	-	-/-	-/-	-	-	+/+	+/+	++	+	-	11
P 7	Yahr IV	2008/'10	Mopio	52	F	47/'05	5	Alive	Auyu	3/3	-	-/-	-/-	-	-	++/+	+/+	++	+	-	-
P 8	Yahr II	2011	Sien	57	F	49/03	9	2012	Auyu	3/3	-	-/-	-/-	-	-	+/+	+/+	+	-	-	12
P 9	Yahr IV	2007/'08/'10/'[illegible]	Bade	46	F	40/'06	6	Alive	Auyu	3/3	-	-/-	-/-	-	-	+/+	++/+	+	+	-	13
P 10	Yahr II	2011/'12	Asset	38	M	27/'01	11	Alive	Auyu	3/3	-	-/-	-/-	-	-	+/-	+/-	+	-	-	-
P 11	Yahr III	2011/'12	Sien	54	F	40/'98	14	Alive	Auyu	3/3	-	-/-	-/-	-	-	++/+	++/+	+	+	-	-
P 12	Yahr II	2011/'12	Memes	66	F	64/10	2	Alive	Auyu	3/3	-	-/-	-/-	-	-	+/+	+/+	+	-	-	14
P 13	Yahr II	2012	Harapan	60	M	57/09	3	Alive	Auyu	2/2	-	-/-	-/-	-	-	-	+/+	-	-	-	-
P 14	Yahr II	2007	Mur	50	M	38/'95	12	NC	Auyu	2/4	+	-/-	-/-	-	-	+	+	+	-	-	-
P 15	Yahr I	2007	Homilikia	50	M	NC	NC	Alive	Auyu	2/3	+	-/-	-/-	-	-	+/-	+/±	-	-	-	-
P 16	Yahr III	2011	Osso	35	F	33/'09	2	2011	Auyu	3/2	-	-/-	-/-	-	-	+/+	+/+	+	+	+	15

Abbreviations: ALS = amyotrophic lateral sclerosis; BK = bradykinesia; BS = Babinski sign; DTR = deep tendon reflex; Dys = dysphagia/dysarthria; F = female; Fasc = fasciculation; LMN = lower motor neuron signs; M = male; MA = muscle atrophy with weakness; NC = not confirmed by examination; P = parkinsonism; PI = postural impairment; R = rigidity; T = tremor; TA = tongue atrophy; U/L: upper limbs/lower limbs; UMN = upper motor neuron signs.

Age: at the time of final survey; Duration: between subjective onset of illness and last survey or death; Alive: alive in 2012; Onset: subjective onset of illness; Deep tendon reflexes, -: absent, 1: hypoactive, 2: normal, 3: hyperactive, 4: markedly hyperactive with ankle clonus.

Other signs are classified as: -: absent; ±: questionably present; +: present; ++: markedly present.

Family history 1: cousin (possible ALS); 2: grandfather (NC); 3: sister (ALS15); 4: cousin's daughter (P16); 5: cousin (NC); 6: father (NC); 7: sister (ALS 7); 8: sister (P 6) and cousin (NC); 9: father (NC); 10: son: (NC); 11: sister (ALS-P 1) and cousin (NC); 12: mother and father (NC); 13: grandmother (NC); 14: mother (NC); 15: ALS 10 (mother’s cousin).

For peer review only

Table 4. Cases of ALS and parkinsonism in the field sites

	ALS criteria	Case No.	Crude point prevalence rate among ALS/ ALS-P/ Parkinsonism (2007* or 2010#)
Bade 2007–2012 (population 3800)			
ALS	Definite	ALS 5*	105 /100,000 in 2007*
(n=4)	Definite	ALS 6*	
	Definite	ALS 7*	
	Definite	ALS 15*	
ALS-parkinsonism	Definite	ALS-P 4*	53 /100,000 in 2007*
(n=2)	Possible	ALS-P 8*	
Parkinsonism (n=1)		P 9*	26 /100,000 in 2007*
Ia river 2007–2012 (population 4500)			
ALS	Definite	ALS 4	133 /100,000 in 2010#
(n=7)	Probable	ALS 8#	
	Probable	ALS 10#	
	Probable	ALS 11#	
	Definite	ALS 12#	
	Definite	ALS 13#	
	Definite	ALS 17#	
ALS-parkinsonism	Possible	ALS-P 2#	89 /100,000 in 2010#
(n=4)	Possible	ALS-P 3#	
	Possible	ALS-P 9#	
	Possible	ALS-P 13#	
Parkinsonism		P 10#	67 /100,000 in 2010#
(n=3)		P 15#	
		P 16#	
Edera and Dumut rivers 2008–2012 (population: 4100)			
ALS	Definite	ALS 9#	73 /100,000 in 2010#
(n=3)	Probable	ALS 14#	
	Definite	ALS 16#	
ALS-parkinsonism	Possible	ALS-P 1	98 /100,000 in 2010#
(n=5)	Definite	ALS-P 5#	
	Definite	ALS-P 6#	
	Possible	ALS-P 10#	
	Possible	ALS-P 11#	
Parkinsonism		P 6	122 /100,000 in 2010
(n=6)		P 7#	
		P 8#	
		P 11#	
		P 12#	
		P 13#	

*:Cases suffered and alive in 2007 in Bade

#:Cases suffered and alive in 2010 in the villages along Ia, Edera and Dumut rivers

For peer review only

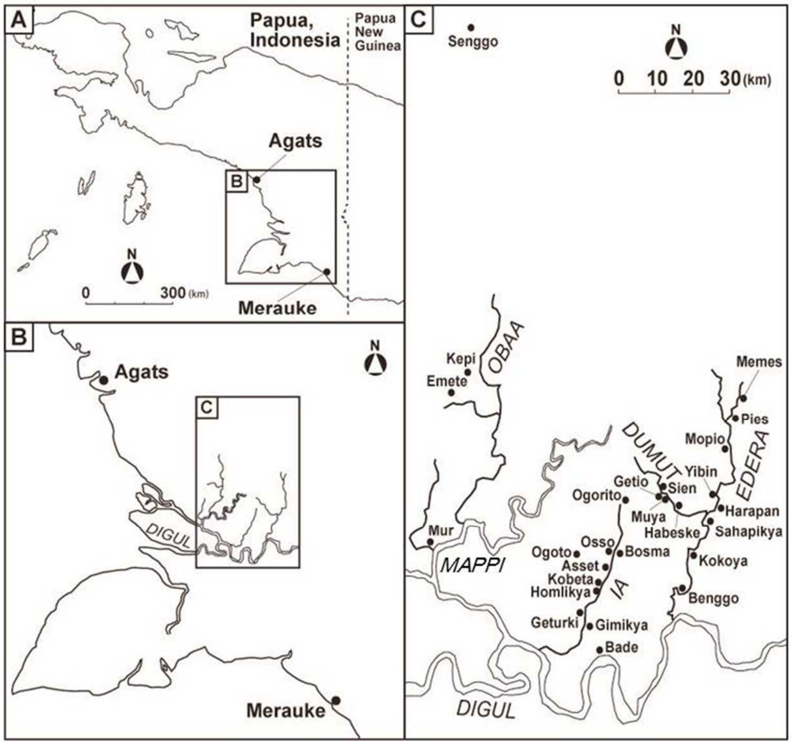

254x190mm (96 x 96 DPI)

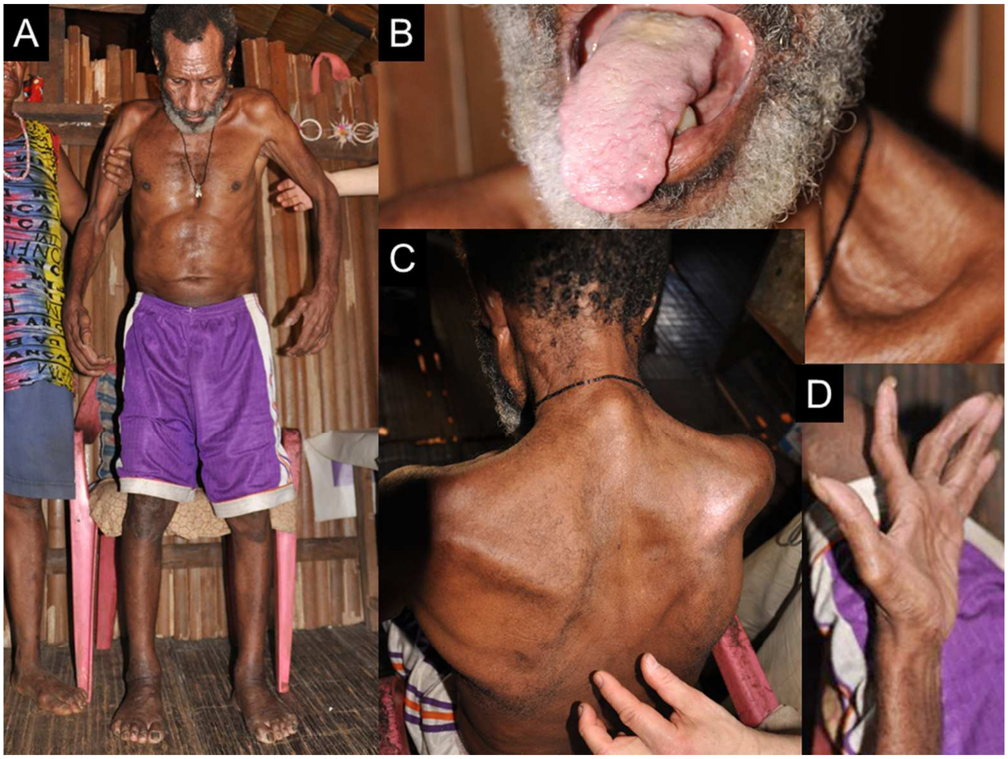

119x90mm (300 x 300 DPI)

For peer review only

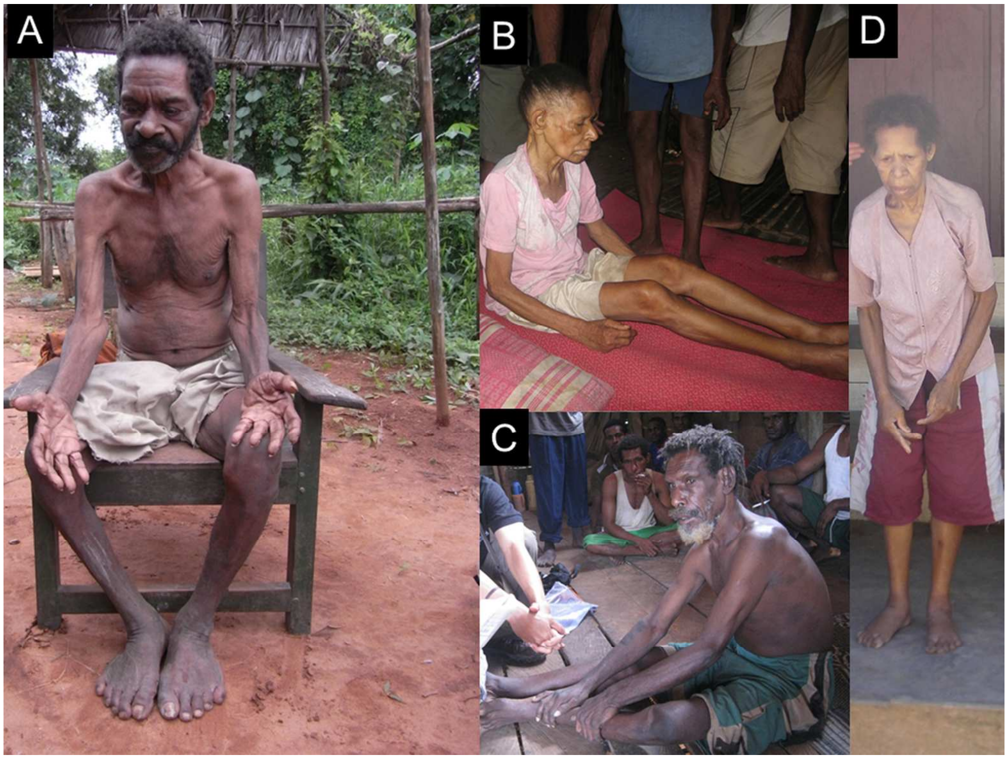

119x90mm (300 x 300 DPI)

For peer review only

Amyotrophic lateral sclerosis and parkinsonism in Papua, Indonesia: 2001–2012 survey results

Journal:	*BMJ Open*
Manuscript ID:	bmjopen-2013-004353.R1
Article Type:	Research
Date Submitted by the Author:	07-Feb-2014
Complete List of Authors:	Okumiya, Kiyohito; Research Institute for Humanity and Nature,<br>Wada, Taizo; Kyoto University, Center for Southeast Asian Studies<br>Fujisawa, Michiko; Center for Southeast Asian Studies,<br>Ishine, Masayuki; Yasugi Clinic,<br>Garcia del Saz, Eva; Kochi University, Center for Regional & International Collaboration<br>Hirata, Yutaka; Kitaakita Municipal Hospital,<br>Kuzuhara, Shigeki; Suzuka University of Medical Science, Faculty of Health Science<br>Kokubo, Yasumasa; Mie University, Graduate School of Regional Innovation Studies<br>Seguchi, Harumichi; Kochi University, Faculty of Medicine<br>Sakamoto, Ryota; Kyoto University, Hakubi Center for Advanced Research<br>Manuaba, Indrajaya; Cenderawasih University, Faculty of Medicine<br>Watofa, Paulina; Cenderawasih University, Faculty of Medicine<br>Rantetampang, Andreas; Cenderawasih University, Faculty of Public Health<br>Matsubayashi, Kozo; Kyoto University, Center for Southeast Asian Studies
**Primary Subject Heading**:	Epidemiology
Secondary Subject Heading:	Neurology
Keywords:	Motor neurone disease < NEUROLOGY, Parkinson-s disease < NEUROLOGY, Dementia < NEUROLOGY

SCHOLARONE™ Manuscripts

Amyotrophic lateral sclerosis and parkinsonism in Papua, Indonesia: 2001–2012 survey results

Kiyohito Okumiya[1], Taizo Wada[2], Michiko Fujisawa[2], Masayuki Ishine[3], Eva Garcia del Saz[4], Yutaka Hirata[5], Shigeki Kuzuhara[6], Yasumasa Kokubo[7], Harumichi Seguchi[8], Ryota Sakamoto[9], Indrajaya Manuaba[10], Paulina Watofa[10], Andreas L. Rantetampang[11], Kozo Matsubayashi[2]

[1]Research Institute for Humanity and Nature, Kyoto, Japan
[2]Center for Southeast Asian Studies, Kyoto University, Kyoto, Japan
[3]Yasugi Clinic, Shimane, Japan
[4]Center for Regional & International Collaboration, Kochi University, Kochi, Japan
[5]Kitaakita Municipal Hospital, Akita, Japan
[6]Faculty of Health Science, Suzuka University of Medical Science, Mie, Japan
[7]Graduate School of Regional Innovation Studies, Mie University, Mie, Japan
[8]Faculty of Medicine, Kochi University, Kochi, Japan
[9]Hakubi Center for Advanced Research, Kyoto University, Kyoto, Japan
[10]Faculty of Medicine, Cenderawasih University, Papua, Indonesia
[11]Faculty of Public Health, Cenderawasih University, Papua, Indonesia

Correspondence to: Kiyohito Okumiya,
Research Institute for Humanity and Nature
Motoyama 457-4, Kamigamo, Kita-ku, Kyoto 603-8047, Japan
Tel: +81-75-707-2411, Fax: +81-75-707-2509
E-mail: okumiyak@chikyu.ac.jp

Abstract:300 words
Text:5238 words
4 tables and 3 figures

For peer review only

ABSTRACT

Objective: Only one prior follow-up study of amyotrophic lateral sclerosis (ALS) and parkinsonism in Papua, Indonesia has been carried out since a survey undertaken from 1962 to 1980 by Gajdusek and colleagues. Therefore, to clarify the clinical epidemiology of ALS and parkinsonism in the south coastal region of Papua, the clinical characteristics and prevalence of the diseases in this region were examined and assessed.

Methods: Cases of ALS and parkinsonism were clinically examined during a 2001–2012 survey in Bade and other villages along the Ia, Edera, Dumut, and Obaa rivers in Papua, Indonesia. Possible, probable, and definite ALS were diagnosed clinically by certified neurologists on the basis of El Escorial Criteria. The criteria for diagnosis of parkinsonism were the presence of at least 2 of the 4 following signs: tremor, rigidity, bradykinesia, and postural impairment with a progressive course.

Results: During the survey, 46 cases of ALS and/or parkinsonism were diagnosed within a population range of 7000 (2001–2002) to 13,900 (2007–2012). The 46 cases consisted of 17 probable-definite cases of ALS, including 3 of cognitive impairment (CI); 13 cases of overlap of possible, probable, or definite ALS and parkinsonism, including 5 cases of CI; and 16 cases of parkinsonism, including 1 case of CI. The crude point prevalence rate of pure ALS was estimated as at least 73 (0–156) to 133 (27–240)/100,000 people and that of the overlap of ALS and parkinsonism was at least 53 (0–126) to 98 (2–193)/100,000 in 2007 or 2010 in some regions.

Conclusions: The prevalence of ALS should have decreased during the 35 years since Gajdusek et al.'s report. However, it remains higher than the global average. There was a high prevalence of overlap of ALS, parkinsonism, and CI, which has been previously

For peer review only - http://bmjopen.bmj.com/site/about/guidelines.xhtml

reported in Guam and Kii.

Strengths and limitations of this study

▪ This study is unique in the epidemiological survey of neurodegenerative diseases during the period between 2001 and 2012 in Papua, Indonesia, one of the foci with the highest incidence of ALS in the world.

▪ The prevalence of ALS is supposed to be decreased since Gajdusek et al.'s report during the 35 years, when there has been an influx of migrants and transmigrants from outside Papua that has changed the population total in the villages studied. However, the prevalence is still higher than the global average.

▪ This study was based only on clinical findings and there are the limitations of not having electromyogram data, DNA analysis, or any autopsy data from this population. Without such data, it is difficult to be certain how these patients fit into sporadic ALS or Parkinson's disease and how they compare with ALS/PDC on Guam and in the Kii peninsula.

For peer review only

INTRODUCTION

Between 1962 and 1980, Gajdusek et al. reported 97 cases of amyotrophic lateral sclerosis (ALS), 18 cases of parkinsonism, and 18 cases of poliomyeloradiculitis (PMR) in the south coastal area of Papua (former Irian Jaya, Indonesia), which had a population of 7,000. The Ia River is in the epicenter of the high-incidence focus of ALS and parkinsonism in Papua.[1,2] Along with Guam and Kii in Japan, Papua (Indonesia) has been considered one of the foci with the highest incidence of ALS in the world.[1-7] The high incidence of ALS and parkinsonism-dementia complex (PDC) in Guam and Kii reportedly no longer exists.[8,9] In 1987, Spencer et al. reported only 2 cases of ALS and three cases of parkinsonism in the villages along the Ia River. In 1990, no cases of ALS and three cases of parkinsonism were reported along the same river; thus, decline or disappearance of ALS was suspected to have also occurred in Papua.[10] However, no follow-up surveys on ALS and parkinsonism have been conducted in Papua since 1991.

To clarify the clinical epidemiological characteristics of ALS and parkinsonism in the south coastal region of Papua, we conducted a survey of neurodegenerative diseases documented between 2001 and 2012.

METHODS

The survey sites for between 2001 and 2002 (total population, 7000) were the villages of Kepi (population, 800), Emete (800), Ogoto (400), Senggo (2000), and Bade (3000).[11,12] For between 2007 and 2012, the survey sites were the villages along 4

For peer review only

rivers (total population, 13,900), namely Bade along the Digul River (population, 3800 people), Mur along the Mappi River (1500), the 8 villages along the Ia River (4500), and the 12 villages along the Edera and Dumut rivers (4100 people). The 8 villages along the Ia River were Gimikya (800), Geturki (500), Homlikya (700), Kobeta (600), Asset (600), Osso (300), Bosma (500), and Ogorito (500). The 12 villages along the Edera and Dumut rivers were Benggo (200), Kokoya (500), Sahapikya (400), Harapan (400), Yibin (300), Mopio (300), Pies (300), Memes (300), Habeske (300), Muya (300), Getio (200), and Sien (600; Figure 1). At least 2 of 6 Japanese-certified neurologists (KO, MF, YH, SK, YK, and KM) visited each village with a local neurologist (IM).

The village leaders and medical staff members of the primary healthcare centre in each village agreed to take part in the survey, which was conducted with the permission of the Department of Health of the Papua Provincial Government in cooperation with Cenderawasih University. The purpose of the neurological examinations was explained to all of the villagers. All the subjects volunteered to participate after the announcement of availability of neurological check-ups, which were performed in the community health centres or homes in each field site. While this was not a complete inventory survey, we asked each medical doctor or health staff, and village leader to call all patients with neurological signs or symptoms, including muscle weakness, gait disturbance, tremor, bradykinesia, or cognitive impairment (CI), in each village. The patients who were called also volunteered to participate. Indonesian collaborators and the co-authors (an English teacher in Bade Senior High School and Indonesian neurologist IM, and staff from Cenderawasih University) who spoke both English and

For peer review only - http://bmjopen.bmj.com/site/about/guidelines.xhtml

Indonesian; local people who spoke both Indonesian and the local language in each village; and co-author EG, who spoke English, Indonesian, and Japanese, cooperatively served as translators. Written informed consent was obtained from all the study participants with the support of their family members. All the participants agreed to undergo neurological examinations, and none of the patients declined annual re-examination. The survey was approved by the ethics committees of the Research Institute for Humanity and Nature in Japan and Cenderawasih University in Papua, Indonesia.

Among the many types of neurological and non-neurological cases encountered, diagnoses of ALS, parkinsonism, and CI were based on clinical examinations and discussions among the participating neurologists. ALS was classified as 'definite', 'probable', 'possible', and 'suspected' on the basis of combinations of upper motor neuron (UMN) and lower motor neuron (LMN) signs according to the El Escorial Criteria of the World Federation of Neurology.[13] Probable and definite ALS are defined as pure ALS in this report. A diagnosis of parkinsonism required the presence of at least 2 of the following 4 signs: tremor, rigidity, bradykinesia, and postural impairment with a progressive course without evidence/history of vascular accidents or history of taking drugs known to induce parkinsonism. The degree of disability of patients with parkinsonism was classified according to the scale of Hoehn and Yahr.[14] Overlap of possible, probable, or definite ALS with parkinsonism was defined as ALS-parkinsonism. CI was diagnosed when loss of memory or impairments of language usage, praxis, or executive functions were identified during interview and clinical examination by neurologists. As cognitive functional test such as the mini–mental state

For peer review only

examination was not performed for all of the participants, patients with complications of functional disabilities attributable to CI in activities of daily living or in their livelihood were screened during the interview of both the patients and their family members. PMR was evaluated using the definition of Gajdusek et al. that PMR is a subacute paralytic condition reminiscent of the Landry-Guillain-Barre syndrome, which combines radicular, or perhaps neuritic elements with more acute onset and sometimes asymmetric paralysis.[1,2] Neurological cases with overlapping cerebellar signs were excluded from this study.

In Bade and the villages along the Ia, Edera, and Dumut rivers, not only were new cases were identified but also previously diagnosed cases were followed up and assessed neurologically and documented in 2007, 2008, 2010, 2011, and 2012. Death years were confirmed, and the durations between subjective onset of illness and the last survey or death were recorded as durations of subjective illness.

The size of the populations of Bade and the villages along the Ia, Edera, and Dumut rivers were determined from a report by the public office of Edera district in 2006.[15] We were informed by the village leaders of the populations of other villages in the survey for the period 2001–2002.

RESULTS

Clinical types of ALS and/or parkinsonism

We identified 46 cases with signs of ALS and/or parkinsonism, including 17 cases with pure ALS (Table 1), 13 with ALS-parkinsonism (Table 2), and 16 with parkinsonism

For peer review only - http://bmjopen.bmj.com/site/about/guidelines.xhtml

(Table 3). We found no case of pure or dominant dementia and PMR consistent with the definition by Gajdusek et al. Relevant information about the cases, including neurological signs, age at last survey, age and year of onset, disease duration, certified death year, and ethnicity, is shown in Tables 1–3. The male-to-female sex distribution was 10:7 (male, 59%) for ALS, 11:2 (85%) for ALS-parkinsonism, and 8:8 (50%) for parkinsonism. The mean age at last survey was 51.3 years for ALS, 52.9 years for ALS-parkinsonism, and 51.6 years for parkinsonism. The mean age at onset was 46.2 years (range, 11–68 years) for pure ALS, 49.5 years (11–70 years) for ALS-parkinsonism, and 45.8 years (27–64 years) for parkinsonism. The mean duration of subjective illness was 7.0 years (2–20 years) for pure ALS, 6.3 years (2–17 years) for ALS-parkinsonism, and 6.3 years (2–14 years) for parkinsonism. We observed overlapping CI in three (18%) of the 17 cases of pure ALS, 5 (38%) of the 13 cases of ALS-parkinsonism, and 1 (6%) of the 16 cases of parkinsonism.

During the follow-up surveys of the cases in the Bade, Ia, and Edera rivers, we observed the following changes in diagnosis: 1 case, from possible ALS to probable ALS (case ALS10); 2 cases, from parkinsonism to ALS-parkinsonism (cases ALS-P 2 and ALS-P 3); and 1 case, from probable ALS to definite ALS (case ALS16) complicated with CI. We also identified an instance of a shared family history of parkinsonism (P 6) and ALS-parkinsonism (ALS-P 1). There were family histories of neurological disorders in 7 of 17 cases (41%) of pure ALS, 2 (15%) of 13 cases of ALS-parkinsonism, and 6 (38%) of 16 cases of parkinsonism (Tables 1–3).

Cases of ALS and parkinsonism in Bade and the villages along the Ia, Edera, and

For peer review only - http://bmjopen.bmj.com/site/about/guidelines.xhtml

For peer review only

Dumut rivers between 2007 and 2012

Between 2007 and 2012, we identified 4 cases of pure ALS, 2 cases of ALS-parkinsonism, and 1 case of parkinsonism in Bade (population, 3800 people). In addition, we found 7 cases of pure ALS, 4 cases of ALS-parkinsonism, and three cases of parkinsonism in the villages (population, 4500 people) along the Ia river. Between 2008 and 2012, we identified three cases of pure ALS, 5 cases of ALS-parkinsonism, and 6 cases of parkinsonism in the villages (population, 4100 people) along the Edera and Dumut rivers (Table 4).

In the 2007 survey, there were 4 active cases of pure ALS (crude point prevalence rate, 105 [2–208]/100,000 people), 2 cases of ALS-parkinsonism (53 [0–126]/100,000 people), and 1 case of parkinsonism (26 [0–78]/100,000 people) in Bade. In 2010, there were 6 active cases of pure ALS (133 [27–240]/100,000 people), 4 cases of ALS-parkinsonism (89 [2–176]/100,000 people), and three cases of parkinsonism (67 [0–142]/100,000 people) in the villages along the Ia River. In 2010, there were three active cases of pure ALS (73 [0–156]/100,000 people), 4 cases of ALS-parkinsonism (98 [2–193]/100,000 people), and 5 cases of parkinsonism (122 [15-229]/100,000 people) in the villages along the Edera and Dumut rivers (Table 4).

In the survey during the period 2007–2012, the number of each clinical type (ALS, ALS-parkinsonism, and parkinsonism) was shown respectively for Bade (4, 4, and 1); the villages along the Ia River, namely Ogorito (1,2, and 0), Bosma (1, 1, and 0), Homlikya (1, 1, and 1), Gimikya (1, 1, and 0), Geturki (1, 0, and 0), Asset (1, 0, and 1), and Osso (1, 0, and 1); and the villages along the Edera and Dumut rivers, namely Yibin

For peer review only - http://bmjopen.bmj.com/site/about/guidelines.xhtml

(1, 1, and 0), Sien (0, 1, and 2), Pies (1, 0, and 1), Benggo (0, 1, and 0), Harapan (0, 0, and 1), Mopio (0, 0, and 1), Memes (0, 0, and 1), Muya (1, 0, and 0), and Getio (0, 1, and 0).

Case reports

Case ALS 12

A 64-year-old (in 2012) man of the Auyu tribe, a resident of Bosma along the Ia River, had a history of progressive gait disturbance and motor weakness for 9 years in 2010; these symptoms were found to have progressed based on the findings from the follow-up check-ups between 2011 and 2012. Neurological examination revealed UMN and LMN signs, including a positive Babinski sign, hyperreflexia with ankle clonus, muscle atrophy, and weakness in the tongue and upper and lower limbs bilaterally, and fasciculation in the upper limbs and tongue. He had no CI or sensory disturbance. We diagnosed him as having definite ALS (Figure 2-A–D). The subject had ALS, and his brother reported that their father showed tremor and gait disturbance, which were interpreted to be consistent with parkinsonism.

Case ALS-CI 17

A 70-year-old (in 2012) man of the Auyu tribe, a resident of Ogorito along the Ia River, had a history of progressive memory loss for 4 years and slow gait for 2 years. Neurological examination revealed UMN and LMN signs, including a positive Babinski sign, hyperreflexia in the jaw jerk, bilateral upper and lower limbs, and muscle atrophy, weakness, and fasciculation in the distal upper and lower limbs bilaterally. He had

For peer review only - http://bmjopen.bmj.com/site/about/guidelines.xhtml

fasciculation in the tongue and respiratory muscle weakness with a low oxygen saturation level (SpO_2, 86%). He also had memory disturbances accompanied by positive snout and palmomental reflexes and difficulty with mental activities of daily living. He had no sensory disturbance. We diagnosed him as having definite ALS with CI (Figure 3A).

Case ALS-P 1

A 65-year-old (in 2008) woman of the Auyu tribe, a resident of Pies along the Edera River, had a history of progressive motor weakness and gait disturbance for 5 years. She could not walk without support. Neurological examination revealed a resting tremor, cogwheel rigidity in the upper and lower limbs, bradykinesia throughout, postural impairment, UMN and LMN signs with a positive Babinski sign, hyperreflexia in the upper and lower limbs bilaterally, and thenar and hypothenar muscle atrophies. She had no CI or sensory disturbance. We diagnosed her as having ALS-parkinsonism and, more specifically, with severe parkinsonism (Hoen and Yahr IV) simultaneously complicated by motor neuron signs indicative of possible ALS. She died in 2009. Her younger sister was case P 6. The subject had ALS-parkinsonism, and her sister reported that their cousin showed tremor and gait disturbance, which were interpreted to be consistent with parkinsonism (Figure 3B).

Case ALS-P 2

A 76-year-old (in 2011) man of the Auyu tribe, a resident of Ogorito, had a history of tremor in both hands for 5 years in 2010. Neurological examination revealed cogwheel rigidity in his upper and lower limbs, bradykinesia, and UMN signs with hyperreflexia

For peer review only - http://bmjopen.bmj.com/site/about/guidelines.xhtml

in bilateral upper and lower limbs. We diagnosed him as having parkinsonism (Hoen and Yahr II) in 2010. He was examined again in 2011, by which time his parkinsonism was complicated by not only postural impairment but also muscle weakness and atrophy, and spasticity of the upper and lower limbs with Babinski signs. He had no CI or sensory disturbance. We diagnosed him as having parkinsonism (Hoen and Yahr III) with possible ALS; parkinsonism was accompanied by ALS later. He died in 2011 of unknown causes.

We also diagnosed another case of ALS-P3 parkinsonism (Hoen and Yahr III) with possible ALS; parkinsonism was accompanied by ALS later.

Case ALS-P-CI 6

A 50-year-old (in 2012) man of the Auyu tribe, a resident of Benggo along the Edera River, had a history of dysarthria for 2 years. Neurological examination revealed UMN and LMN signs with a positive Babinski sign, muscle atrophy, and weakness in the tongue, proximal upper limb, shoulder, and tibialis anterior muscles. He had cogwheel rigidity in the wrist and elbow, bradykinesia throughout, a positive Myerson sign, and impairment of coordination and postural reflex. He had memory loss, bradyphrenia, slow speech, and positive snout and palmomental reflexes with difficulty performing activities of daily living. We diagnosed him as having a definite ALS and parkinsonism (Hoen and Yahr III) with CI (Figure 3C).

Case P 7

For peer review only - http://bmjopen.bmj.com/site/about/guidelines.xhtml

A 52-year-old (in 2010) woman of the Auyu tribe who lived in Mopio along the Edera River had a history of progressive gait disturbance with parkinsonian signs, including resting tremor, cogwheel rigidity, anterior bending, and bradykinesia for 3 years in 2008. In 2008, we diagnosed her as having parkinsonism (Hoen and Yahr II). By the time of the follow-up survey in 2010, she could not stand by herself and had postural impairment with parkinsonian hands bilaterally. She had no UMN or LMN signs, no CI, and no sensory disturbance. We diagnosed her as having parkinsonism (Hoen and Yahr IV; Figure 3D).

DISCUSSION

Internal migration and migration from outside the province are the most significant causes of demographic, social, and cultural change in Papua. The populations in the survey sites were composed of former hunter-gatherers who lived in the forest and who were brought out of the forest and assembled in villages by Dutch colonialists. The villages along the Ia and Digul rivers were founded in 1940–1950.[10] The Dutch colonial program of population distribution from highly populated regions such as Java and Bali to Papua and other less populated province was continued by the Indonesian Government. The emigration program called transmigration (*transmigrasi*) started in Irian Jaya in 1964. Local migrants and transmigrants from outside Irian Jaya increased, resulting in various changes, including the use of food and medicine.[16,17] Considering that Gadjusek et al. found out the high prevalence of ALS at the first survey in 1962, the neurodegenerative disease existed before the introduction of manufactured items.[1]

For peer review only - http://bmjopen.bmj.com/site/about/guidelines.xhtml

For peer review only

In the present survey, we performed full neurological examinations in 46 subjects with ALS and/or parkinsonism between 2001 and 2012. Among the subjects, 17 were probable-definite cases of pure ALS, 13 were overlapping ALS (possible, probable, or definite) and parkinsonism, and 16 were parkinsonism. Nine cases of ALS and/or parkinsonism had a complication of CI. During the study period, the diagnosis in some patients changed from pure parkinsonism to ALS-parkinsonism, and their disease progressed in severity. Some subjects had both ALS and parkinsonism on their first examination. It is thus evident that overlapping ALS and parkinsonism with or without CI still exists in the specified region of Papua.

Gajdusek et al. reported diagnosing 97 cases (57 cases clinically confirmed and 40 cases not clinically confirmed) of ALS during the 6 surveys they conducted from 1962 to 1980.[1] However, they reported neurological findings of only 32 of these cases, in only 16 of which the reported findings of UMN, LMN, and parkinsonian signs justify a diagnosis of probable or definite ALS. The 16 cases comprise 12 cases of pure ALS and 4 cases of overlapping ALS-parkinsonism. Among the 32 ALS patients, three had a positive family history (9%); that is, 2 had sisters with ALS, and 1 who had ALS-parkinsonism had a family history of ALS. None of the ALS patients had a family history of parkinsonism. The authors also reported their neurological findings in 13 subjects in whom parkinsonism was diagnosed. They reported their findings from full neurological examinations of 10 of these parkinsonism cases; according to their reported neurological signs, these comprised 8 patients of parkinsonism, including 5 with dementia and 2 with overlapping ALS-parkinsonism with dementia.[1] Among the

For peer review only - http://bmjopen.bmj.com/site/about/guidelines.xhtml

For peer review only

13 parkinsonism patients, three had a positive family history (23%). In summary, we believe that in the 26 cases reported by Gajdusek et al., the full neurological examination findings comprised 12 pure ALS cases (probable or definite), 6 overlapping ALS-parkinsonism, and 8 parkinsonism with/without dementia.[1] Spencer et al reported 2 cases of ALS and three cases of parkinsonism, including 1 with dementia, in the survey in 1987. He also reported 3 subjects with parkinsonism overlapping UMN signs, including 1 with dementia in 1991. We have here reported 17 cases of pure ALS (probable or definite), 13 cases of overlapping ALS-parkinsonism, and 16 cases of parkinsonism with or without CI in the 46 cases we neurologically examined fully. Thus, we identified many cases of overlapping ALS and parkinsonism (n = 13, 28%) in our study and in previous reports (n = 6, 23% and n = 3, 38%).[1, 10] We also identified many cases with CI (n = 9, 20%) in our survey and in previous reports (n = 7, 27% and n = 2, 25%).[1, 10] Functional disabilities attributable to CI were recognized in activities of daily living or in their livelihood in all the cases with CI in this report.

Gajdusek et al. reported 13 cases of ALS and three cases of parkinsonism in 1975 in the villages along the Ia River (population, 2000).[1] The 13 ALS cases consisted of 10 clinically confirmed ones and 3 others that were not clinically confirmed. We compared the prevalence of probable-definite ALS and parkinsonism according to the report of Gajdusek et al. and our more present findings. A crude point prevalence of ALS consistent with clinically probable to definite ALS (El Escorial criteria) may be estimated between 500 (95% confidence interval, 191–809/100,000 people) and 650 (298–1002) and that of parkinsonism was 150 (0–320/100,000 people) in 1975 along

For peer review only - http://bmjopen.bmj.com/site/about/guidelines.xhtml

the Ia River. In our 2010 survey (population, 4500), we identified 6 probable-definite cases of ALS in this region (ALS 8, 10–13, and 17; 133 [27–-240]/100,000 people) and 7 of parkinsonism (possible ALS-P 2, 3, 9, and 13, and P 10, 15, and 16; 156 [40–271]/100,000 people).

Gajdusek et al. reported 8 cases of ALS and 6 cases of parkinsonism in 1975 in the villages along the Edera and Dumut rivers (population, 1950). The 8 cases of ALS consisted of 7 clinically confirmed cases and 1 unclinically confirmed case. The crude point prevalence of ALS consistent with clinically probable to definite ALS (El Escorial criteria) may be estimated between 359 (94–624)/100,000 people and 410 (127–694)/100,000 people, and that of parkinsonism was 308 (62–554)/100,000 people in 1975 along the Edera and Dumut rivers. In our survey of this region in 2010 (population, 4100), we identified 5 probable-definite cases of ALS (ALS 9, 14, and 16, and ALS-P 5, 6; 122 [15–229]/100,000 people) and 7 cases of parkinsonism (possible ALS-P 10 and 11, and P 7, 8, and 11–13; 171 [44–297]/100,000 people; Table 4). As this was not a complete inventory survey, the actual prevalence may be a little higher than our estimation.

The population of Bade increased by a factor of 4 during 31 years (from 1000 in 1975 to 3800 in 2006), and those of villages along the Ia River (from 2000 to 4500) and the Edera and Dumut rivers (from 1950 to 4100) have increased 2-fold.[1,15] The subjects born outside Papua were only 2 (Maluku) among the 46 subjects in this survey. The prevalence of the diseases among the local Papuan residents in this survey may be underestimated compared with the former report after taking into account the increase

For peer review only - http://bmjopen.bmj.com/site/about/guidelines.xhtml

in the numbers of local migrants inside and transmigrants from outside Papua.[16,17,18]

As the median age and life expectancy in the field sites in Papua are estimated to be lower than the global median and fewer people may live beyond the age of 40 or 50 years,[18,19] the prevalence of the diseases in this survey may be underestimated compared with the global mean. As the median age and life expectancy in 1975 may be lower than those in 2010,[20] the prevalence of the diseases in the report by Gajdusek et al. might be underestimated compared with that in our survey.

The prevalence of ALS in this report was found to be three times lower than found by Gajdusek et al. Even after taking into account the change of population and age structure in the field sites, our findings show that the prevalence of ALS is supposed to be decreased during the 35 years since the report of Gajdusek et al.[10] This is consistent with the previous report by Spencer et al. However, it is still much higher than the global mean (incidence, approximately 2/100,000; prevalence, approximately 6/100,000) [21-26]; in particular, the prevalence of overlapping ALS and parkinsonism is especially high in Papua.

In the report by Gajdusek et al., who provided village-by-village data showing the number of cases of ALS and parkinsonism, there were many cases in the remote villages such as Bosma, Pies, Homlikya, Yibin, Sien, and Ogorite, which were less visited and least developed.[1, 2] In this survey, many cases (three ALS/ALS-parkinsonism) were found in the remotest village of Ogorito along the Ia River. Two cases of ALS/ALS-parkinsonism were also found in Bosma, Homlikya, and

For peer review only - http://bmjopen.bmj.com/site/about/guidelines.xhtml

Yibin; 1 ALS and 2 cases of parkinsonism in Sien; 1 ALS and 1 parkinsonism in Pies, which consisted also of remote villages. While there were 8 cases of ALS/ALS-parkinsonism in Bade and 2 in Gimikya, nearest to the port of the Digul River, none was reported by Gajdusek et al. in those sites. This increase may be brought about by local migrants from the affected villages.

Overlap of ALS-parkinsonism and dementia has also been reported in patients with ALS/PDC in Guam and Kii, Japan.[4, 27-33] Hirano et al. provided clinicopathological evidence of 47 cases of PDC in Guam in 1961, comprising the following 3 groups: (1) 30 cases of PDC; (2) 8 cases of parkinsonism with dementia with clinical evidence of UMN impairment; and (3) 9 cases of parkinsonism with dementia with clinical evidence of UMN and LMN impairment. At least 15 of these cases (31.9%) had a family history of ALS, parkinsonism, or both.[4] Kuzuhara and Kokubo et al. surveyed neurological diseases in Kii, Japan, in the 1990s and found not only a continuously high incidence of ALS but also neuropathologically verified cases of PDC. ALS and PDC frequently affected the same individuals simultaneously and the members of the same family. These authors reported 37 cases of ALS/PDC between 1996 and 2006, a high proportion of whom (78%) had a positive family history, as well as clinical examples of pure ALS, PDC, and overlapping ALS and PDC. They also reported neuropathological changes common to cases of ALS and PDC in 12 cases of ALS/PDC, resembling those found in patients with ALS/PDC in Guam [28-33] In our more recent study in Papua, such as the previous studies in Kii, Japan, and Guam, we identified many cases of pure ALS and ALS overlapping with parkinsonism with/without CI. The prevalence of CI was lower in our study sites than in Guam and Kii.[4, 27-33] CI might have been underestimated,

For peer review only - http://bmjopen.bmj.com/site/about/guidelines.xhtml

and mild CIs or some mild dementia might have been overlooked by our neurological examinations in the field.

Our study was based only on clinical findings, and there are the limitations of not having electromyogram data, DNA analysis data, or any autopsy data from this population. Without such data, it is difficult to be certain how these patients fit into sporadic ALS or Parkinson disease and how they compare with ALS/PDC on Guam and in the Kii peninsula. However, we did identify many patients with overlapping ALS-parkinsonism in this study. The overlap of ALS and/or parkinsonism with CI suggests that it may be the same disease entity as the ALS/PDC diagnosed in Guam and Kii.

The proportion of males in the populations of Bade, the villages along the Ia River, and the villages along the Edera and Dumut rivers were 53%, 51%, and 50%. The proportion of males was high especially among the subjects with ALS-parkinsonism (85%) and ALS (59%) compared with among those with pure parkinsonism (50%) in our report. The proportion of males was higher among the subjects with ALS (63%) and parkinsonism (76%) in the report by Gadjusek et al., in which there were 3 male cases (75%) among the 4 ALS-parkinsonism cases. The high risks for ALS and PDC among the males were recognized in Guam and Kii.[1,7,27]

Indigenous people of Papua still retain aspects of their traditional lifestyles, such as hunting, gathering, fishing, and eating *sago* (trunk of *Metroxylon* sp.). They also drink water from shallow wells. However, with the spread of a market economy, recent increases in the numbers of rubber plantations and progress in transportation have been causing lifestyle changes in the villages in the south coastal area of Papua. As the

For peer review only - http://bmjopen.bmj.com/site/about/guidelines.xhtml

diseases are declining as in Guam and Kii, the disease aetiology in Papua may be dominated by environmental factors. Genetic factors may also play a role as evidenced by the family history of disease in 40% of the subjects in this survey in Papua and 80% in Kii.

Various possible causal genetic and environmental factors for these conditions have been reported. The environmental factors of low concentrations of calcium and magnesium in drinking water were hypothesized. [1,34] There is a recent report showing an association between the increase in ALS incidence and the change in drinking water source in the Kii Peninsula.[35] Spencer et al. pointed out that this explanation was inconsistent with a declining prevalence of ALS in spite of drinking water from shallow wells, which this report may support.[10]

Spencer et al. reported the discovery that cycad seed was used as a poultice in Papua, as in Guam. [36] Cycad-derived products were shown as the risk factor of dementia, mild CI, and PDC in Guam with the epidemiological evidence.[37] Although cycad had been suspected as the causal factor of Kii ALS,[38] people in Japan, including the Kii Peninsula, had never consumed cycad as daily food, although a small amount of cycad flour might have been taken by a few people as an ingredient of herb medicine.[39] We did not examine the association of prior skin wounds and exposure to cycad poultice with the diseases. Skin change and pigmentary retinopathy were not examined in this survey.[40, 41] Further studies are needed.

Genetic abnormalities that had been previously established to be associated with

For peer review only

For peer review only - http://bmjopen.bmj.com/site/about/guidelines.xhtml

familial or sporadic ALS, dementia, familial Parkinson disease, and parkinsonism were looked for in the cases in Kii, but none of them were identified.[28,42] Hermousura et al. have discovered a nuclear mutation in the calcium/magnesium membrane ion channel transient receptor potential melastatin 7 (*TRPM7*) gene in a subset of patients with ALS/PDC in Guam.[43] They reported that a TRPM7 variant that is associated with altered sensitivity to magnesium may have contributed to the pathogenesis of ALS and PDC in 2 Guamanians. *TRPM7* was not found to be associated with ALS/PDC in the Kii peninsula of Japan.[44] Ishiura et al. have recently discovered *C9ORF72* repeat expansion in some cases of ALS in the Kii peninsula of Japan; this repeat expansion partly accounts for the high prevalence of ALS in this region.[45] However, the responsible gene(s) remain unproved in almost all cases of ALS/PDC in both Guam and Kii.

Various environmental factors and multiple genetic factors are suggested to be underlying causes of these diseases, but these links are so far unproven.[33,46]

In Guam, although the incidence of pure ALS has declined markedly, the prevalence of PDC is still high.[47] The prevalence of dementia among elderly Chamorros (earliest known inhabitants of the Mariana Islands, which include Guam) is also reportedly relatively high.[48] In addition, in Kii the incidence of pure ALS has declined markedly, and the prevalence of PDC and ALS/PDC or ALS with dementia recently increased.[30,33] These changing patterns of ALS/PDC incidence in Guam and Kii might be caused by changes in environmental and socioeconomic factors as well as aging.[48,49]

For peer review only - http://bmjopen.bmj.com/site/about/guidelines.xhtml

The mean age of onset in our cases was 46 years for ALS, 53 years for ALS-parkinsonism, and 46 years for parkinsonism. Thus, they were older than the previously reported cases of ALS (33 years) and parkinsonism (43 years).[1] In our cases, the mean duration of subjective illness was 7.0 years for ALS, 6.3 years for ALS-parkinsonism, and 6.3 years for parkinsonism. The duration of ALS in our data was longer than not only the world mean of 3 years[21-26] but also the previously reported 3.5 years for ALS in Papua.[1] The mean age of onset may recently have increased, and the mean disease duration became longer in subjects with ALS. The prevalence rate of cases with bulbar sign was 37% in 30 of pure ALS or ALS-parkinsonian in our report, and it was as the same as in the report of Gadjdusek (40%) and classical ALS (39%).[2] The change in onset and course of ALS in recent years might be attributable to the aging population and changing environmental and socioeconomic factors.

On-going follow-up surveys should be performed to evaluate changes in the clinical types of ALS and/or parkinsonism with or without CI and accompanying changes in environmental and socioeconomic factors, as have occurred in Guam and Kii.

In conclusion, The prevalence of ALS is supposed to be decreased during the 35 years since the report of Gajdusek et al., when there has been an influx of migrants inside and transmigrants from outside Papua that has changed the population in the villages studied. However, there are still many cases of pure ALS and overlapping ALS-parkinsonism and CI, as has previously been reported in Guam and Kii. Environmental factors may play important etiologic role. Further on-going follow-up surveys focusing on the

For peer review only

For peer review only - http://bmjopen.bmj.com/site/about/guidelines.xhtml

clinical epidemiology and aetiology of these neurodegenerative diseases in association with ecological and environmental factors in Papua are needed. Future studies, including neuropathological examinations, should be performed when the local villagers in this region of Papua, Indonesia, are willing to consent to autopsies.

For peer review only

ACKNOWLEDGMENTS

The authors wish to thank all the participants in the villages who volunteered for the survey, the village leaders, and Mappi Regency officials. We are deeply indebted to doctors Imelda Mandagi, Reginald Nangoy, and Christha Z. Tamburian and their staff at Puskesmas Bade, and to Arifin Wasaraka, Lazarus Revasy, Yosefina Griapon, Arius Togodly, Kerry Yarangga, and Dolfinus Y. Bouway at Cenderawasih University for their invaluable contributions to the study. Andre Liem and Herry Wondiwoy at Papua Tour Guides Community and S.E. Amos at Bade High School provided logistic and language assistance. On the Japanese side, we acknowledge the technical support and advice of Katsuyuki Eguchi (Nagasaki University), Yasushi Osaki (Kochi University), Hidekazu Tomimoto (Mie University), Taro Yamauchi (The University of Tokyo), Hideyuki Onishi (Doshisha Women's College), Tetsuya Inamura (Aichi Prefectural University), Wenling Chen, Yumi Kimura, Yasuko Ishimoto, and Hissei Imai (Kyoto University), and Yasuyuki Kosaka, Yukiko Kita, and Chizu Wada (Research Institute for Humanity and Nature).

For peer review only - http://bmjopen.bmj.com/site/about/guidelines.xhtml

AUTHOR CONTRIBUTIONS

Okumiya had full access to all the data in the study and takes responsibility for the integrity of the data and the accuracy of the data analysis.

Study concept and design: Okumiya, Matsubayashi.

Acquisition of data: Okumiya, Wada, Fujisawa, Ishine, Garcia del Saz Hirata, Kuzuhara, Kokubo, Seguchi, Sakamoto, Manuaba, Matsubayashi.

Analysis and interpretation of data: Okumiya,Wada, Sakamoto, Fujisawa, Matusbayashi.

Drafting of the manuscript: Okumiya,Wada, Sakamoto, Fujisawa, Matusbayashi.

Critical revision of the manuscript for important intellectual content: All authors.

Statistical analysis: Okumiya.

Obtained funding: Okumiya, Wada, Kuzuhara, Kokubo, Matsubayashi

Administrative, technical, or material support: Okumiya, Watofa, Rantetampang, Matsubayashi.

Study supervision: Okumiya, Matsubayashi.

COMPETING INTERESTS : None declared

For peer review only

FUNDING

K. Okumiya receives research support from Grant-in-Aid for Scientific Research (A) from the Japan Society for the Promotion of Science (JSPS). K. Matsubayashi receives research support from Grant-in-Aid for Scientific Research (A) from the JSPS and is an editor of the journal *Geriatrics & Gerontology International*. T. Wada receives research

For peer review only - http://bmjopen.bmj.com/site/about/guidelines.xhtml

support from Grant-in-Aid for Scientific Research (C) from the JSPS. Y. Kokubo and S. Kuzuhara receive support from Grants-in-Aid from the Research Committee of CNS Degenerative Diseases and the Research Committee on the Muro disease (Kii amyotrophic lateral sclerosis/Parkinsonism-dementia), the Ministry of Health, Labour and Welfare of Japan.

REFERENCES

1. Gajdusek DC, Salazar AM. Amyotrophic lateral sclerosis and parkinsonian syndromes in high incidence among the Auyu and Jakai people of West New Guinea. Neurology 1982;**32**:107–26.
2. Gajdusek DC. Foci of motor neuron disease in high incidence in isolated populations of East Asia and the Western Pacific. In: Lewis P Rowland, ed. Human Motor Neuron Diseases. New York: Raven Press 1982:363–93.
3. Kurland LT, Mulder DW. Epidemiologic investigations of amyotrophic lateral sclerosis. 1. Preliminary report on geographic distribution and special reference to the Mariana Islands, including clinical and pathologic observations. Neurology 1954;**4**:438–48.
4. Hirano A, Kurland LT, Krooth RS, et al. Parkinsonism-dementia complex, an endemic disease on the island of Guam. I. Clinical features. Brain 1961;**84**:642–61.
5. Hirano A, Malamud N, Kurland LT. Parkinsonism-dementia complex, an endemic disease on the island of Guam. II. Pathological features. Brain 1961;**84**:662–79.
6. Kimura K, Yase Y, Higashi Y, et al. Epidemiological and geomedical studies on ALS and allied diseases in Kii peninsula (Japan): preliminary report. Proc Jpn Acad 1961;**37**:417–20.

For peer review only

7. Shiraki H, Yase Y. Amyotrophic lateral sclerosis in Japan. In: Vinken PJ, Bruyn GW, eds. Handbook of clinical neurology Vol. 22, System disorders and atrophies, Part II. Amsterdam: Elsevier Science 1975:353–419.

8. Garruto RM, Yanagihara R, Gajdusek DC. Disappearance of high incidence ALS and PDC on Guam. Neurology 1985;**35**:193–8.

9. Yoshida S. ALS tahatsuchi niokeru kankyoyoin to aluminium no neuron hensei eno kanyo nitsuite.[Environmental factors in western Pacific foci of ALS and a possible pathogenetic role of aluminum (Al) in motor neuron degeneration] (in Japanese with English abstract). Rinsho Shinkeigaku [Clin Neurol] 1991;**31**:1310–2.

10. Spencer P, Palmer VS, Ludolph AC. On the decline and etiology of high-incidence motor system disease in West Papua (Southwest New Guinea). Mov Disord 2005;**20(**Suppl 12):S119–126.

11. Wada T, Matsubayashi K, Okumiya K, et al. Health status and subjective economic satisfaction in West Papua. Lancet 2002;**360**:951.

12. Okumiya K, Fujisawa M, Ishine M, et al. Nishi New Guinea chiiki (Indonesia/ Papua shu) no shinkeihensei shikkan no jittai. 2001-2002 nen, 2006-2007 nen no fieldwork yori. [Fieldwork survey of neurodegenerative diseases in West New-Guinea in 2001-02 and 2006-07] (in Japanese). Rinsho Shinkeigaku [Clin Neurol] 2007;**47**:977–8.

13. Brooks BR. El Escorial World Federation of Neurology criteria for the diagnosis of amyotrophic lateral sclerosis: subcommittee on Motor Neuron Diseases/Amyotrophic Lateral Sclerosis of the World Federation of Neurology Research Group on Neuromuscular Diseases and the El Escorial “Clinical Limits of ALS” Workshop Contributors. J Neurol Sci 1994;**124**:96–107.

For peer review only - http://bmjopen.bmj.com/site/about/guidelines.xhtml

For peer review only

14. Hoehn MM, Yahr MD. Parkinsonism: onset, progression and mortality. Neurology. 1967;**17**:427–42.

15. Kontor Statistik Distrik Edera. Laporan Kependudukan, Kabupaten Mappi Distrik Edera 2006 (December).

16. Manning C., Rumiak M. Irian Jaya: Economic change, migrants, and indinenous welfare. In Hill H ed. Unity and diversity: Regional Economic Development in Indonesia since 1970. Oxford University Press, Okford.p77-106, 1991.

17. Timmer J. A brief social and political history of Papua, 1962-2005. In Marshall AJ and Beehler BM ed. The Ecology of Indonesian Papua Part Two. Periplus Editions, Hong Kong. p1098-1124, 2007.

18. Badan Pusat Statistik, Sensus Penduduk 2010. http://sp2010.bps.go.id/index.php

19. United Nations , Department of Economic and Social Affairs, Population Division. World Population Prospects The 2012 Revision *Volume II: Demographic Profiles,* http://esa.un.org/unpd/wpp/index.htm

20. Kontor Statistik Kabupaten Merauke. Laporan Kependudukan, Kabupaten Merauke 1997

21. Armon C. Amyotrophic lateral sclerosis. In: Neuroepidemiology from principles to practice. USA: Oxford University Press 2004:162–87.

22. McGuire V, Longstreth WT Jr, Koepsell TD, et al. Incidence of amyotrophic lateral sclerosis in three counties in western Washington state. Neurology 1996 ;**47**:571–3.

23. Sorenson EJ, Stalker AP, Kurland LT, et al. Amyotrophic lateral sclerosis in Olmsted County, Minnesota, 1925 to 1998. Neurology 2002; **23**:280–2.

24. O'Toole O, Traynor BJ, Brennan P, et al. Epidemiology and clinical features of amyotrophic lateral sclerosis in Ireland between 1995 and 2004. J Neurol Neurosurg

For peer review only - http://bmjopen.bmj.com/site/about/guidelines.xhtml

Psychiatry 2008;**79**:30–2.

25. Chiò A, Mora G, Calvo A, et al. Epidemiology of ALS in Italy: a 10-year prospective population-based study. Neurology 2009;**72**:725–31.
26. Factor-Litvak P, Al-Chalabi A, Ascherio A, et al. Current pathways for epidemiological research in amyotrophic lateral sclerosis. Amyotroph Lateral Scler Frontotemporal Degener 2013;**14**(Suppl 1):33–43.
27. Rodgers-Johnson P, Garruto RM, Yanigahara R, et al. Amyotrophic lateral sclerosis and parkinsonism-dementia on Guam: a 30-year evaluation of clinical and neurological trends. *Neurology.* 1986;36:7-13.
28. Kuzuhara S, Kokubo Y, Sasaki R, et al. Familial amyotrophic lateral sclerosis and parkinsonism-dementia complex of the Kii peninsula of Japan: clinical and neuropathological study and tau analysis. Ann Neurol 2001;**49**:501–11.
29. Kuzuhara S, Kokubo Y. Atypical parkinsonism of Japan: amyotrophic lateral sclerosis-parkinsonism-dementia complex of the Kii peninsula of Japan (Muro disease): an update. Mov Disord 2005;**20**(Suppl 12):S108–13.
30. Kuzuhara S. Kii ALS/PDC (Murou byou) no rekishi, gainen, ekigaku. [ALS-parkinsonism-dementia complex of the Kii peninsula of Japan (Muro disease). Historical review, epidemiology and concept] (in Japanese with English abstract). Rinsho Shinkeigaku [Clin Neurol] 2007;**47**:962–5.
31. Kokubo Y. Kiihanto no kinishukuseisokusakukokasho/ parkinson chihou hukugo no rinshou, shinkei byourigakuteki kenkyu. [Clinical aspects, imaging and neuropathology of Kii ALS/PDC] (in Japanese with English abstract). Rinsho Shinkeigaku [Clin Neurol] 2007;**47**:966–9.
32. Kuzuhara S. Kii hanto no Murobyo (Kinishukuseisokusakukokasho/ parkinson/

For peer review only - http://bmjopen.bmj.com/site/about/guidelines.xhtml

ninchisho hukugo) [Muro disease or ALS–parkinsonism–dementia complex of the Kii peninsula of Japan] (in Japanese with English abstract). Rinsho Shinkeigaku [Clin Neurol] 2007;**47**:695–702.

33. Kuzuhara S, KokuboY. Amyotrophic lateral sclerosis-parkinsonism-dementia complex in the Kii peninsula of Japan (Muro disease): a review on recent research and new concept. In: Michael J. Strong, ed. Amyotrophic lateral sclerosis and the frontotemporal dementias. Oxford: Oxford University Press 2012:39–54.
34. Yase Y. The pathogenesis of amyotrophic lateral sclerosis. Lancet 1972;**2**(7772):292–6.
35. Kihara T, Yoshida S, Kondo T et al. An increase in ALS incidence on the Kii Peninsula, 1960-2009: A possible link to change in drinking water source. *Amyotrophic Lateral Sclerosis,* 2012; 13: 347–50.
36. Spencer PS, Nunn PB, Hugon J, et al. Guam amyotrophic lateral sclerosis-parkinsonism-dementia linked to a plant excitant neurotoxin. Science 1987;**237**(4814):517–22.
37. Borenstein AR, Mortimer JA, Schofield E. et al. Cycad exposure and risk of dementia, MCI, and PDC in the Chamorro population of Guam. Neurology. 2007;68:1764-71.
38. Spencer PS, Ohta M, Palmer VS. Cycad use and motor neuron disease in Kii peninsula of Japan. Lancet 1987;**2**(8573):1462–3.
39. Iwami O, Niki Y, Watanabe T, and Ikeda M. Motor neuron disease on the Kii Peninsula of Japan: Cycad exposure. Neuroepidemiology 1993;12:307-312.
40. Ono S, Waring SC, Kurland L et al. Guamanian neurodegenerative disease: ultrastructural studies of skin J. Neurol. Sci., 146 (1997), pp. 35–40
41. Kokubo Y, Ito K, Kuzuhara S. Ophthalmomyiasis-like pigmentary retinopathy in

For peer review only - http://bmjopen.bmj.com/site/about/guidelines.xhtml

ALS/PDC in the Kii peninsula of Japan. Neurology. 2003 May 27;60(10):1725-6.

42. Tomiyama H, Kokubo Y, Sasaki R, et al. Mutation analyses in amyotrophic lateral sclerosis/parkinsonism-dementia complex of the Kii peninsula, Japan. Mov Disord 2008;**23**(16):2344–8.

43. Hermosura MC, Nayakanti H, Dorovkov MV, et al. A TRPM7 variant shows altered sensitivity to magnesium that may contribute to the pathogenesis of two Guamanian neurodegenerative disorders. Proc Natl Acad Sci 2005;**102**(32):11510–5.

44. Hara K, Kokubo Y, Ishiura H, et al. TRPM7 is not associated with amyotrophic lateral sclerosis-parkinsonism dementia complex in the Kii peninsula of Japan. Am J Med Genet B Neuropsychiatr Genet 2010;**153B**(1):310–3.

45. Ishiura H, Takahashi Y, Mitsui J, et al. *C9ORF72* Repeat expansion in amyotrophic lateral sclerosis in the Kii peninsula of Japan. Arch Neurol 2012;**69**(9):1154–8.

46. Garruto RM, Yanagihara R. Contributions of isolated Pacific populations to understanding neurodegenerative diseases. Folia Neuropathol 2009;**47**(2):149–70.

47. Plato CC, Garruto RM, Galasko D, et al. Amyotrophic lateral sclerosis and parkinsonism-dementia complex of Guam: changing incidence rates during the past 60 years. Am J Epidemiol 2003;**157**:149–57.

48. Galasko D, Salmon D, Gamst A, et al. Prevalence of dementia in Chamorros on Guam: relationship to age, gender, education, and APOE. Neurology 2007;**68**:1772–81.

49. Plato CC, Galasko D, Garruto RM, et al. ALS and PDC of Guam: forty-year follow-up. Neurology 2002;**58**:765–73.

For peer review only

Figure and table legends

Table 1. Relevant characteristics and neurological signs of cases with ALS with/without cognitive impairment in Papua, 2001–2012.

Abbreviations: ALS = amyotrophic lateral sclerosis; BK = bradykinesia; BS = Babinski sign; DTR = deep tendon reflex; Dys = dysphagia/dysarthria; F= female; Fasc = fasciculation; LMN = lower motor neuron signs; M = male; MA = muscle atrophy with weakness; NC = not confirmed by examination; P = parkinsonism; PI = postural impairment; R = rigidity; T = tremor; TA = tongue atrophy; U/L: upper limbs/lower limbs; UMN = upper motor neuron signs.

Age: at the time of final survey; Duration: between subjective onset of illness and last survey or death; Alive: alive in 2012; Onset: subjective onset of the illness; Deep tendon reflexes, -: absent, 1: hypoactive, 2: normal, 3: hyperactive, 4: markedly hyperactive with ankle clonus.

Other signs are classified as: -: absent; ±: questionably present; +: present; ++: markedly present.

Family history 1: cousin (possible ALS); 2: grandfather (NC); 3: sister (ALS15); 4: cousin's daughter (P16); 5: cousin (NC); 6: father (NC); 7: sister (ALS 7); 8: sister (P 6) and cousin (NC); 9: father (NC); 10: son: (NC); 11: sister (ALS-P 1) and cousin (NC); 12: mother and father (NC); 13: grandmother (NC); 14: mother (NC); 15: ALS 10 (mother’s cousin).

For peer review only - http://bmjopen.bmj.com/site/about/guidelines.xhtml

Table 2. Relevant characteristics and neurological signs in cases with overlap of ALS and parkinsonism with/without cognitive impairment in Papua, 2001–2012.

Table 3. Relevant characteristics and neurological signs in cases with parkinsonism with/without cognitive impairment in Papua, 2001–2012.

Table 4. Cases of ALS and parkinsonism according to field site

*; Active cases in 2007 in Bade

#; Active cases in 2010 in the villages along the Ia, Edera, and Dumut rivers

Figure 1. Map of the field sites

A. Large-scale map of the southern coastal plain in Papua, Indonesia.

B. Smaller scale map of the southern coastal plain in Papua, Indonesia showing rivers.

C. Detailed map showing villages in the southern coastal area in Papua in which all confirmed cases of ALS and/or parkinsonism with/without cognitive impairment were seen from 2001 to 2012.

Figure 2. Photographs of patient with ALS (Case ALS 12**)**

A: The patient needs assistance from family members to stand.

B: Advanced atrophy of the tongue.

C: There is upper limb-girdle and truncal muscle atrophy with a positive Babinski sign.

D: Advanced thenar muscle atrophy.

Figure 3. Photographs of patients with ALS, ALS-parkinsonism, and parkinsonism

For peer review only - http://bmjopen.bmj.com/site/about/guidelines.xhtml

A: Patient with ALS (Case ALS-17) showing muscle atrophy in the distal upper and lower limbs.

B: Patient with ALS-parkinsonism (Case ALS-P 1) showing muscle atrophy in distal upper limbs.

C: Patient with ALS-parkinsonism (Case ALS-P 7) showing atrophy in muscles in the proximal upper limb, shoulder, and tibialis anterior.

D: Patient with parkinsonism (Case P 7) showing typical parkinsonian hands and anterior bending posture.

Parkinsonian hand: Striatal deformities of the hand with abnormal postures that are common in patients with advanced Parkinson's disease.

For peer review only

For peer review only - http://bmjopen.bmj.com/site/about/guidelines.xhtml

Okumiya

For peer review only

Table 1. Relevant characteristics and neurological signs in cases with pure ALS with/without cognitive impairment in Papua, 2001–2012.

Case number	ALS criteria Yahr stage	Survey year	Village	Age	Sex	Onset (age/year)	Duration (years)	Death (year)	Tribe (origin)	UMN signs: DTR U/L	UMN signs: BS	LMN signs: MA U/L	LMN signs: Fasc U/L	Bulbar signs: TA	Bulbar signs: Dys	Parkinsonian sign: T U/L	Parkinsonian sign: R U/L	Parkinsonian sign: BK	Parkinsonian sign: PI	Cognitive impairment	Family history
Pure ALS (n=17)																					
ALS 1	Definite	2001	Bade	35	M	NC	NC	NC	Auyu	3/3	+	++/++	+/+	++	-	-	-	-	-	-	-
ALS 2	Definite	2001	Ogoto	43	F	NC	NC	2002	Auyu	3/3	+	++/++	+/+	++	++	-	-	-	-	-	-
ALS 3	Definite	2002	Senggo	50	M	48/'00	2	NC	Citak	3/3	+	++/++	±/±	±	+	-	-	-	-	-	1
ALS 4	Definite	2007	Homilikia	53	M	51/'05	2	2007	Auyu	3/2	+	++/++	+/+	-	-	-	-	-	-	-	-
ALS 5	Definite	2007/'08	Bade	51	M	49/'06	3	2009	Auyu	3/4	+	+/-	-/-	-	+	-	-	-	-	-	-
ALS 6	Definite	2008	Bade	21	M	11/'98	11	2009	Auyu	4/4	+	++/++	-/-	-	-	-	-	-	-	-	2
ALS 7	Definite	2007/'08/'10	Bade	70	F	57/'97	13	2010	Maluku	3/3	+	++/++	+/-	-	+	-	-	-	-	-	3
ALS 8	Probable	2011	Geturki	53	M	48/'06	5	Alive	Auyu	3/3	-	+/+	-/+	-	-	-	-	-	-	-	-
ALS 9	Definite	2011	Muya	41	F	36/'06	6	2012	Auyu	3/3	+	+/+	-/-	-	-	-	-	-	-	-	-
ALS 10	Probable	2007/'10/'11/'12	Osso	65	F	59/'06	6	Alive	Auyu	3/4	+	+/-	-/-	+	-	-	-	-	-	-	4
ALS 11	Probable	2007/'08/'10/'12	Gimikya	32	M	12/'92	20	Alive	Auyu	4/4	+	++/+	++/-	±	-	-	+/-	-	-	-	5
ALS 12	Definite	2010/'11/'12	Bosma	64	M	53/'01	11	Alive	Auyu	3/4	+	++/++	+/-	++	-	-	-	-	-	-	6
ALS 13	Definite	2012	Asset	70	F	68/'10	2	Alive	Auyu	3/3	-	+/+	-/-	-	-	-	-	-	-	-	-
ALS 14	Probable	2012	Pies	65	F	60/'07	5	Alive	Auyu	3/4	-	+/+	-/-	-	-	-	-	-	-	-	-
ALS 15	Definite	2007	Bade	64	F	62/'05	2	2007	Maluku	3/3	+	++/++	-/-	-	+	-	-	-	-	+	7
ALS 16	Definite	2008/'10/'11	Yibin	25	M	13/'99	13	2012	Auyu	3/4	+	++/+	++/++	-	-	-	-	-	-	+	-
ALS 17	Definite	2012	Ogorito	70	M	66/'08	4	Alive	Auyu	3/3	+	+/+	+/+	-	-	-	-	-	-	+	-

Abbreviations: ALS = amyotrophic lateral sclerosis; BK = bradykinesia; BS = Babinski sign; DTR = deep tendon reflex; Dys = dysphagia/dysarthria; F = female; Fasc = fasciculation; LMN = lower motor neuron signs; M = male; MA = muscle atrophy with weakness; NC = not confirmed by examination; P = parkinsonism; PI = postural impairment; R = rigidity; T = tremor; TA = tongue atrophy; U/L: upper limbs/lower limbs; UMN = upper motor neuron signs.

Age: at the time of final survey; Duration: between subjective onset of illness and last survey or death; Alive: alive in 2012; Onset: subjective onset of illness; Deep tendon reflexes, -: absent, 1: hypoactive, 2: normal, 3: hyperactive, 4: markedly hyperactive with ankle clonus.

Other signs are classified as: -: absent; ±: questionably present; +: present; ++: markedly present.

Family history 1: cousin (possible ALS); 2: grandfather (NC); 3: sister (ALS15); 4: cousin's daughter (P16); 5: cousin (NC); 6: father (NC); 7: sister (ALS 7); 8: sister (P 6) and cousin (NC); 9: father (NC); 10: son: (NC); 11: sister (ALS-P 1) and cousin (NC); 12: mother and father (NC); 13: grandmother (NC); 14: mother (NC); 15: ALS 10 (mother’s cousin).

Table 2. Relevant characteristics and neurological signs in cases with overlap of ALS and parkinsonism with/without cognitive impairment in Papua, 2001–2012.

Case number	ALS criteria Yahr stage	Survey year	Village	Age	Sex	Onset (age/year)	Duration (year)	Death (year)	Tribe (origin)	UMN signs DTR U/L	UMN signs BS	LMN signs MA U/L	LMN signs Fasc U/L	Bulbar signs TA	Bulbar signs Dys	Parkinsonian sign T U/L	Parkinsonian sign R U/L	Parkinsonian sign BK	Parkinsonian sign PI	Cognitive impairment	Family history
ALS-parkinsonism (n = 13)																					
ALS-P 1	Possible-IV	2008	Pies	65	F	60/'03	6	2009	Auyu	3/3	+	+/-	-/-	-	-	+/+	++/++	++	+	-	8
ALS-P 2	Possible-III	2010/'11	Ogorito	76	M	70/'05	6	2011	Auyu	3/3	+	+/±	-/-	-	-	+/+	+/+	+	+	-	-
ALS-P 3	Possible-III	2010/'11/'12	Homilikia	69	M	65/'08	4	Alive	Auyu	3/2	+	+/-	-/-	-	-	+/+	+/+	+	+	-	-
ALS-P 4	Definite-III	2008	Bade	34	M	18/'92	17	2009	Muyu	3/3	+	+/-	-/-	++	++	-	+/+	+	+	+	9
ALS-P 5	Definite-III	2012	Sien	60	M	58/'10	2	Alive	Auyu	3/3	+	+/+	-/-	-	-	-	+/+	+	+	+	-
ALS-P 6	Definite-III	2012	Benggo	50	M	48/'10	2	Alive	Auyu	2/2	+	+/+	-/-	+	+	-	+/-	+	+	+	-
ALS-P 7	Possible-II	2001	Bade	36	M	NC	NC	NC	Auyu	3/3	+	-/-	-/-	-	-	+	+	+	-	-	-
ALS-P 8	Possible-II	2008/'10	Bade	23	M	11/'98	12	Alive	Auyu	3/4	+	-/-	-/-	-	-	+/+	+/±	+	-	-	-
ALS-P 9	Possible-III	2010/'11	Ogorito	76	M	70/'05	6	2011	Auyu	3/3	+	-/-	-/-	-	-	+/-	+/+	+	+	-	-
ALS-P 10	Possible-III	2008/'10/'11	Yibin	52	M	44/'03	8	Alive	Auyu	3/3	+	-/-	-/-	-	-	+/-	+/+	+	+	-	-
ALS-P 11	Possible-III	2011	Getio	55	F	53/09	2	2011	Auyu	3/3	+	±/-	-/-	-	-	+/+	+/+	+	+	-	-
ALS-P 12	Possible-IV	2001	Bade	40	M	NC	NC	NC	Auyu	3/3	+	-/-	-/-	-	+	+	+	+	+	+	-
ALS-P 13	Possible-III	2012	Bosma	52	M	48/'08	4	Alive	Auyu	3/4	+	-/-	-/-	-	-	-	++/+	+	+	+	-

Abbreviations: ALS = amyotrophic lateral sclerosis; BK = bradykinesia; BS = Babinski sign; DTR = deep tendon reflex; Dys = dysphagia/dysarthria; F = female; Fasc = fasciculation; LMN = lower motor neuron signs; M = male; MA = muscle atrophy with weakness; NC = not confirmed by examination; P = parkinsonism; PI = postural impairment; R = rigidity; T = tremor; TA = tongue atrophy; U/L: upper limbs/lower limbs; UMN = upper motor neuron signs.

Age: at the time of final survey; Duration: between subjective onset of illness and last survey or death; Alive: alive in 2012; Onset: subjective onset of illness; Deep tendon reflexes, -: absent, 1: hypoactive, 2: normal, 3: hyperactive, 4: markedly hyperactive with ankle clonus.

Other signs are classified as: -: absent; ±: questionably present; +: present; ++: markedly present.

Family history 1: cousin (possible ALS); 2: grandfather (NC); 3: sister (ALS15); 4: cousin's daughter (P16); 5: cousin (NC); 6: father (NC); 7: sister (ALS 7); 8: sister (P 6) and cousin (NC); 9: father (NC); 10: son: (NC); 11: sister (ALS-P 1) and cousin (NC); 12: mother and father (NC); 13: grandmother (NC); 14: mother (NC); 15: ALS 10 (mother's cousin).

For peer review only

Table 3. Relevant characteristics and neurological signs in cases with parkinsonism with/without cognitive impairment in Papua, 2001–2012.

Case number	ALS criteria Yahr stage	Survey year	Village	Age	Sex	Onset (age/year)	Duration (years.)	Death (year)	Ethnicity	UMN signs		LMN signs		Bulbar signs		Parkinsonian sign				Cognitive impairment	Family history
										DTR U/L	BS	MA U/L	Fasc U/L	TA	Dys	T U/L	R U/L	BK	PI		
Parkinsonism (n = 16)																					
P 1	Yahr IV	2001	Kepi	50	M	NC	NC	NC	Jakai	2/2	-	-/-	-/-	-	-	+/+	+/+	++	+	-	-
P 2	Yahr II	2001	Kepi	50	M	NC	NC	NC	Jakai	2/2	-	-/-	-/-	-	-	+	+	+	-	-	-
P 3	Yahr IV	2002	Emete	52	M	49/'99	3	NC	Jakai	2/2	-	-/-	-/-	-	-	+	+	+	+	-	-
P 4	Yahr II	2002	Senggo	41	M	36/'97	5	NC	Citak	2/2	-	-/-	-/-	-	-	+	+	-	-	-	-
P 5	Yahr I	2007	Mur	65	F	61/'03	4	NC	Jakai	2/3	-	-/-	-/-	-	-	+/-	+/-	-	-	-	10
P 6	Yahr IV	2008	Pies	60	F	55/'03	6	2009	Auyu	2/2	-	-/-	-/-	-	-	+/+	+/+	++	+	-	11
P 7	Yahr IV	2008/'10	Mopio	52	F	47/'05	5	Alive	Auyu	3/3	-	-/-	-/-	-	-	++/+	+/+	++	+	-	-
P 8	Yahr II	2011	Sien	57	F	49/03	9	2012	Auyu	3/3	-	-/-	-/-	-	-	+/+	+/+	+	-	-	12
P 9	Yahr IV	2007/'08/'10/'[illegible]	Bade	46	F	40/'06	6	Alive	Auyu	3/3	-	-/-	-/-	-	-	+/+	++/+	+	+	-	13
P 10	Yahr II	2011/'12	Asset	38	M	27/'01	11	Alive	Auyu	3/3	-	-/-	-/-	-	-	+/-	+/-	+	-	-	-
P 11	Yahr III	2011/'12	Sien	54	F	40/'98	14	Alive	Auyu	3/3	-	-/-	-/-	-	-	++/+	++/+	+	+	-	-
P 12	Yahr II	2011/'12	Memes	66	F	64/10	2	Alive	Auyu	3/3	-	-/-	-/-	-	-	+/+	+/+	+	-	-	14
P 13	Yahr II	2012	Harapan	60	M	57/09	3	Alive	Auyu	2/2	-	-/-	-/-	-	-	-	+/+	-	-	-	-
P 14	Yahr II	2007	Mur	50	M	38/'95	12	NC	Auyu	2/4	+	-/-	-/-	-	-	+	+	+	-	-	-
P 15	Yahr I	2007	Homilikia	50	M	NC	NC	Alive	Auyu	2/3	+	-/-	-/-	-	-	+/-	+/±	-	-	-	-
P 16	Yahr III	2011	Osso	35	F	33/'09	2	2011	Auyu	3/2	-	-/-	-/-	-	-	+/+	+/+	+	+	+	15

Abbreviations: ALS = amyotrophic lateral sclerosis; BK = bradykinesia; BS = Babinski sign; DTR = deep tendon reflex; Dys = dysphagia/dysarthria; F = female; Fasc = fasciculation; LMN = lower motor neuron signs; M = male; MA = muscle atrophy with weakness; NC = not confirmed by examination; P = parkinsonism; PI = postural impairment; R = rigidity; T = tremor; TA = tongue atrophy; U/L: upper limbs/lower limbs; UMN = upper motor neuron signs.
Age: at the time of final survey; Duration: between subjective onset of illness and last survey or death; Alive: alive in 2012; Onset: subjective onset of illness; Deep tendon reflexes, -: absent, 1: hypoactive, 2: normal, 3: hyperactive, 4: markedly hyperactive with ankle clonus.
Other signs are classified as: -: absent; ±: questionably present; +: present; ++: markedly present.
Family history 1: cousin (possible ALS); 2: grandfather (NC); 3: sister (ALS15); 4: cousin's daughter (P16); 5: cousin (NC); 6: father (NC); 7: sister (ALS 7); 8: sister (P 6) and cousin (NC); 9: father (NC); 10: son: (NC); 11: sister (ALS-P 1) and cousin (NC); 12: mother and father (NC); 13: grandmother (NC); 14: mother (NC); 15: ALS 10 (mother's cousin).

For peer review only

For peer review only - http://bmjopen.bmj.com/site/about/guidelines.xhtml

Table 4. Cases of ALS and parkinsonism in the field sites

	ALS criteria	Case No.	Crude point prevalence rate (95%CI) among ALS/ ALS-P/ Parkinsonism (2007* or 2010#)
Bade 2007–2012 (population 3800)			
ALS	Definite	ALS 5*	105 (2-208) /100,000 in 2007*
(n=4)	Definite	ALS 6*	
	Definite	ALS 7*	
	Definite	ALS 15*	
ALS-parkinsonism	Definite	ALS-P 4*	53 (0-126) /100,000 in 2007*
(n=2)	Possible	ALS-P 8*	
Parkinsonism (n=1)		P 9*	26 (0-78) /100,000 in 2007*
Ia river 2007–2012 (population 4500)			
ALS	Definite	ALS 4	133(27-240) /100,000 in 2010#
(n=7)	Probable	ALS 8#	
	Probable	ALS 10#	
	Probable	ALS 11#	
	Definite	ALS 12#	
	Definite	ALS 13#	
	Definite	ALS 17#	
ALS-parkinsonism	Possible	ALS-P 2#	89 (2-176) /100,000 in 2010#
(n=4)	Possible	ALS-P 3#	
	Possible	ALS-P 9#	
	Possible	ALS-P 13#	
Parkinsonism		P 10#	67 (0-142) /100,000 in 2010#
(n=3)		P 15#	
		P 16#	
Edera and Dumut rivers 2008–2012 (population: 4100)			
ALS	Definite	ALS 9#	73 (0-156) /100,000 in 2010#
(n=3)	Probable	ALS 14#	
	Definite	ALS 16#	
ALS-parkinsonism	Possible	ALS-P 1	98 (2-193) /100,000 in 2010#
(n=5)	Definite	ALS-P 5#	
	Definite	ALS-P 6#	
	Possible	ALS-P 10#	
	Possible	ALS-P 11#	
Parkinsonism		P 6	122 (15-229) /100,000 in 2010
(n=6)		P 7#	
		P 8#	
		P 11#	
		P 12#	
		P 13#	

*:Active cases in 2007 in Bade
#:Active cases in 2010 in the villages along Ia, Edera and Dumut rivers
95%CI: 95% confidence interval

For peer review only

For peer review only - http://bmjopen.bmj.com/site/about/guidelines.xhtml

Amyotrophic lateral sclerosis and parkinsonism in Papua, Indonesia: 2001–2012 survey results

Kiyohito Okumiya[1], Taizo Wada[2], Michiko Fujisawa[2], Masayuki Ishine[3], Eva Garcia del Saz[4], Yutaka Hirata[5], Shigeki Kuzuhara[6], Yasumasa Kokubo[7], Harumichi Seguchi[8], Ryota Sakamoto[9], Indrajaya Manuaba[10], Paulina Watofa[10], Andreas L. Rantetampang[11], Kozo Matsubayashi[2]

[1]Research Institute for Humanity and Nature, Kyoto, Japan
[2]Center for Southeast Asian Studies, Kyoto University, Kyoto, Japan
[3]Yasugi Clinic, Shimane, Japan
[4]Center for Regional & International Collaboration, Kochi University, Kochi, Japan
[5]Kitaakita Municipal Hospital, Akita, Japan
[6]Faculty of Health Science, Suzuka University of Medical Science, Mie, Japan
[7]Graduate School of Regional Innovation Studies, Mie University, Mie, Japan
[8]Faculty of Medicine, Kochi University, Kochi, Japan
[9]Hakubi Center for Advanced Research, Kyoto University, Kyoto, Japan
[10]Faculty of Medicine, Cenderawasih University, Papua, Indonesia
[11]Faculty of Public Health, Cenderawasih University, Papua, Indonesia

Correspondence to: Kiyohito Okumiya,
Research Institute for Humanity and Nature
Motoyama 457-4, Kamigamo, Kita-ku, Kyoto 603-8047, Japan
Tel: +81-75-707-2411, Fax: +81-75-707-2509
E-mail: okumiyak@chikyu.ac.jp

Abstract:300 words
Text:5238 words
4 tables and 3 figures

For peer review only

ABSTRACT

Objective: Only one prior follow-up study of amyotrophic lateral sclerosis (ALS) and parkinsonism in Papua, Indonesia has been carried out since a survey undertaken from 1962 to 1980 by Gajdusek and colleagues. Therefore, to clarify the clinical epidemiology of ALS and parkinsonism in the south coastal region of Papua, the clinical characteristics and prevalence of the diseases in this region were examined and assessed.

Methods: Cases of ALS and parkinsonism were clinically examined during a 2001–2012 survey in Bade and other villages along the Ia, Edera, Dumut, and Obaa rivers in Papua, Indonesia. Possible, probable, and definite ALS were diagnosed clinically by certified neurologists on the basis of El Escorial Criteria. The criteria for diagnosis of parkinsonism were the presence of at least 2 of the 4 following signs: tremor, rigidity, bradykinesia, and postural impairment with a progressive course.

Results: During the survey, 46 cases of ALS and/or parkinsonism were diagnosed within a population range of 7000 (2001–2002) to 13,900 (2007–2012). The 46 cases consisted of 17 probable-definite cases of ALS, including 3 of cognitive impairment (CI); 13 cases of overlap of possible, probable, or definite ALS and parkinsonism, including 5 cases of CI; and 16 cases of parkinsonism, including 1 case of CI. The crude point prevalence rate of pure ALS was estimated as at least 73 (0–156) to 133 (27–240)/100,000 people and that of the overlap of ALS and parkinsonism was at least 53 (0–126) to 98 (2–193)/100,000 in 2007 or 2010 in some regions.

Conclusions: The prevalence of ALS should have decreased during the 35 years since Gajdusek et al.'s report. However, it remains higher than the global average. There was a high prevalence of overlap of ALS, parkinsonism, and CI, which has been previously

For peer review only - http://bmjopen.bmj.com/site/about/guidelines.xhtml

reported in Guam and Kii.

Strengths and limitations of this study

- This study is unique in the epidemiological survey of neurodegenerative diseases during the period between 2001 and 2012 in Papua, Indonesia, one of the foci with the highest incidence of ALS in the world.
- The prevalence of ALS is supposed to be decreased since Gajdusek et al.'s report during the 35 years, when there has been an influx of migrants and transmigrants from outside Papua that has changed the population total in the villages studied. However, the prevalence is still higher than the global average.
- This study was based only on clinical findings and there are the limitations of not having electromyogram data, DNA analysis, or any autopsy data from this population. Without such data, it is difficult to be certain how these patients fit into sporadic ALS or Parkinson's disease and how they compare with ALS/PDC on Guam and in the Kii peninsula.

For peer review only - http://bmjopen.bmj.com/site/about/guidelines.xhtml

INTRODUCTION

Between 1962 and 1980, Gajdusek et al. reported 97 cases of amyotrophic lateral sclerosis (ALS), 18 cases of parkinsonism, and 18 cases of poliomyeloradiculitis (PMR) in the south coastal area of Papua (former Irian Jaya, Indonesia), which had a population of 7,000. The Ia River is in the epicenter of the high-incidence focus of ALS and parkinsonism in Papua.[1,2] Along with Guam and Kii in Japan, Papua (Indonesia) has been considered one of the foci with the highest incidence of ALS in the world.[1-7] The high incidence of ALS and parkinsonism-dementia complex (PDC) in Guam and Kii reportedly no longer exists.[8,9] In 1987, Spencer et al. reported only 2 cases of ALS and three cases of parkinsonism in the villages along the Ia River. In 1990, no cases of ALS and three cases of parkinsonism were reported along the same river; thus, decline or disappearance of ALS was suspected to have also occurred in Papua.[10] However, no follow-up surveys on ALS and parkinsonism have been conducted in Papua since 1991.

To clarify the clinical epidemiological characteristics of ALS and parkinsonism in the south coastal region of Papua, we conducted a survey of neurodegenerative diseases documented between 2001 and 2012.

METHODS

The survey sites for between 2001 and 2002 (total population, 7000) were the villages of Kepi (population, 800), Emete (800), Ogoto (400), Senggo (2000), and Bade (3000).[11,12] For between 2007 and 2012, the survey sites were the villages along 4

For peer review only - http://bmjopen.bmj.com/site/about/guidelines.xhtml

rivers (total population, 13,900), namely Bade along the Digul River (population, 3800 people), Mur along the Mappi River (1500), the 8 villages along the Ia River (4500), and the 12 villages along the Edera and Dumut rivers (4100 people). The 8 villages along the Ia River were Gimikya (800), Geturki (500), Homlikya (700), Kobeta (600), Asset (600), Osso (300), Bosma (500), and Ogorito (500). The 12 villages along the Edera and Dumut rivers were Benggo (200), Kokoya (500), Sahapikya (400), Harapan (400), Yibin (300), Mopio (300), Pies (300), Memes (300), Habeske (300), Muya (300), Getio (200), and Sien (600; Figure 1). At least 2 of 6 Japanese-certified neurologists (KO, MF, YH, SK, YK, and KM) visited each village with a local neurologist (IM).

The village leaders and medical staff members of the primary healthcare centre in each village agreed to take part in the survey, which was conducted with the permission of the Department of Health of the Papua Provincial Government in cooperation with Cenderawasih University. The purpose of the neurological examinations was explained to all of the villagers. All the subjects volunteered to participate after the announcement of availability of neurological check-ups, which were performed in the community health centres or homes in each field site. While this was not a complete inventory survey, we asked each medical doctor or health staff, and village leader to call all patients with neurological signs or symptoms, including muscle weakness, gait disturbance, tremor, bradykinesia, or cognitive impairment (CI), in each village. The patients who were called also volunteered to participate. Indonesian collaborators and the co-authors (an English teacher in Bade Senior High School and Indonesian neurologist IM, and staff from Cenderawasih University) who spoke both English and

For peer review only - http://bmjopen.bmj.com/site/about/guidelines.xhtml

Indonesian; local people who spoke both Indonesian and the local language in each village; and co-author EG, who spoke English, Indonesian, and Japanese, cooperatively served as translators. Written informed consent was obtained from all the study participants with the support of their family members. All the participants agreed to undergo neurological examinations, and none of the patients declined annual re-examination. The survey was approved by the ethics committees of the Research Institute for Humanity and Nature in Japan and Cenderawasih University in Papua, Indonesia.

Among the many types of neurological and non-neurological cases encountered, diagnoses of ALS, parkinsonism, and CI were based on clinical examinations and discussions among the participating neurologists. ALS was classified as ‘definite’, ‘probable’, ‘possible’, and ‘suspected’ on the basis of combinations of upper motor neuron (UMN) and lower motor neuron (LMN) signs according to the El Escorial Criteria of the World Federation of Neurology.[13] Probable and definite ALS are defined as pure ALS in this report. A diagnosis of parkinsonism required the presence of at least 2 of the following 4 signs: tremor, rigidity, bradykinesia, and postural impairment with a progressive course without evidence/history of vascular accidents or history of taking drugs known to induce parkinsonism. The degree of disability of patients with parkinsonism was classified according to the scale of Hoehn and Yahr.[14] Overlap of possible, probable, or definite ALS with parkinsonism was defined as ALS-parkinsonism. CI was diagnosed when loss of memory or impairments of language usage, praxis, or executive functions were identified during interview and clinical examination by neurologists. As cognitive functional test such as the mini–mental state

For peer review only - http://bmjopen.bmj.com/site/about/guidelines.xhtml

examination was not performed for all of the participants, patients with complications of functional disabilities attributable to CI in activities of daily living or in their livelihood were screened during the interview of both the patients and their family members. PMR was evaluated using the definition of Gajdusek et al. that PMR is a subacute paralytic condition reminiscent of the Landry-Guillain-Barre syndrome, which combines radicular, or perhaps neuritic elements with more acute onset and sometimes asymmetric paralysis.[1,2] Neurological cases with overlapping cerebellar signs were excluded from this study.

In Bade and the villages along the Ia, Edera, and Dumut rivers, not only were new cases were identified but also previously diagnosed cases were followed up and assessed neurologically and documented in 2007, 2008, 2010, 2011, and 2012. Death years were confirmed, and the durations between subjective onset of illness and the last survey or death were recorded as durations of subjective illness.

The size of the populations of Bade and the villages along the Ia, Edera, and Dumut rivers were determined from a report by the public office of Edera district in 2006.[15] We were informed by the village leaders of the populations of other villages in the survey for the period 2001–2002.

RESULTS

Clinical types of ALS and/or parkinsonism

We identified 46 cases with signs of ALS and/or parkinsonism, including 17 cases with pure ALS (Table 1), 13 with ALS-parkinsonism (Table 2), and 16 with parkinsonism

For peer review only - http://bmjopen.bmj.com/site/about/guidelines.xhtml

(Table 3). We found no case of pure or dominant dementia and PMR consistent with the definition by Gajdusek et al. Relevant information about the cases, including neurological signs, age at last survey, age and year of onset, disease duration, certified death year, and ethnicity, is shown in Tables 1–3. The male-to-female sex distribution was 10:7 (male, 59%) for ALS, 11:2 (85%) for ALS-parkinsonism, and 8:8 (50%) for parkinsonism. The mean age at last survey was 51.3 years for ALS, 52.9 years for ALS-parkinsonism, and 51.6 years for parkinsonism. The mean age at onset was 46.2 years (range, 11–68 years) for pure ALS, 49.5 years (11–70 years) for ALS-parkinsonism, and 45.8 years (27–64 years) for parkinsonism. The mean duration of subjective illness was 7.0 years (2–20 years) for pure ALS, 6.3 years (2–17 years) for ALS-parkinsonism, and 6.3 years (2–14 years) for parkinsonism. We observed overlapping CI in three (18%) of the 17 cases of pure ALS, 5 (38%) of the 13 cases of ALS-parkinsonism, and 1 (6%) of the 16 cases of parkinsonism.

During the follow-up surveys of the cases in the Bade, Ia, and Edera rivers, we observed the following changes in diagnosis: 1 case, from possible ALS to probable ALS (case ALS10); 2 cases, from parkinsonism to ALS-parkinsonism (cases ALS-P 2 and ALS-P 3); and 1 case, from probable ALS to definite ALS (case ALS16) complicated with CI. We also identified an instance of a shared family history of parkinsonism (P 6) and ALS-parkinsonism (ALS-P 1). There were family histories of neurological disorders in 7 of 17 cases (41%) of pure ALS, 2 (15%) of 13 cases of ALS-parkinsonism, and 6 (38%) of 16 cases of parkinsonism (Tables 1–3).

Cases of ALS and parkinsonism in Bade and the villages along the Ia, Edera, and

For peer review only - http://bmjopen.bmj.com/site/about/guidelines.xhtml

Dumut rivers between 2007 and 2012

Between 2007 and 2012, we identified 4 cases of pure ALS, 2 cases of ALS-parkinsonism, and 1 case of parkinsonism in Bade (population, 3800 people). In addition, we found 7 cases of pure ALS, 4 cases of ALS-parkinsonism, and three cases of parkinsonism in the villages (population, 4500 people) along the Ia river. Between 2008 and 2012, we identified three cases of pure ALS, 5 cases of ALS-parkinsonism, and 6 cases of parkinsonism in the villages (population, 4100 people) along the Edera and Dumut rivers (Table 4).

In the 2007 survey, there were 4 active cases of pure ALS (crude point prevalence rate, 105 [2–208]/100,000 people), 2 cases of ALS-parkinsonism (53 [0–126]/100,000 people), and 1 case of parkinsonism (26 [0–78]/100,000 people) in Bade. In 2010, there were 6 active cases of pure ALS (133 [27–240]/100,000 people), 4 cases of ALS-parkinsonism (89 [2–176]/100,000 people), and three cases of parkinsonism (67 [0–142]/100,000 people) in the villages along the Ia River. In 2010, there were three active cases of pure ALS (73 [0–156]/100,000 people), 4 cases of ALS-parkinsonism (98 [2–193]/100,000 people), and 5 cases of parkinsonism (122 [15-229]/100,000 people) in the villages along the Edera and Dumut rivers (Table 4).

In the survey during the period 2007–2012, the number of each clinical type (ALS, ALS-parkinsonism, and parkinsonism) was shown respectively for Bade (4, 4, and 1); the villages along the Ia River, namely Ogorito (1,2, and 0), Bosma (1, 1, and 0), Homlikya (1, 1, and 1), Gimikya (1, 1, and 0), Geturki (1, 0, and 0), Asset (1, 0, and 1), and Osso (1, 0, and 1); and the villages along the Edera and Dumut rivers, namely Yibin

For peer review only - http://bmjopen.bmj.com/site/about/guidelines.xhtml

(1, 1, and 0), Sien (0, 1, and 2), Pies (1, 0, and 1), Benggo (0, 1, and 0), Harapan (0, 0, and 1), Mopio (0, 0, and 1), Memes (0, 0, and 1), Muya (1, 0, and 0), and Getio (0, 1, and 0).

Case reports

Case ALS 12

A 64-year-old (in 2012) man of the Auyu tribe, a resident of Bosma along the Ia River, had a history of progressive gait disturbance and motor weakness for 9 years in 2010; these symptoms were found to have progressed based on the findings from the follow-up check-ups between 2011 and 2012. Neurological examination revealed UMN and LMN signs, including a positive Babinski sign, hyperreflexia with ankle clonus, muscle atrophy, and weakness in the tongue and upper and lower limbs bilaterally, and fasciculation in the upper limbs and tongue. He had no CI or sensory disturbance. We diagnosed him as having definite ALS (Figure 2-A–D). The subject had ALS, and his brother reported that their father showed tremor and gait disturbance, which were interpreted to be consistent with parkinsonism.

Case ALS-CI 17

A 70-year-old (in 2012) man of the Auyu tribe, a resident of Ogorito along the Ia River, had a history of progressive memory loss for 4 years and slow gait for 2 years. Neurological examination revealed UMN and LMN signs, including a positive Babinski sign, hyperreflexia in the jaw jerk, bilateral upper and lower limbs, and muscle atrophy, weakness, and fasciculation in the distal upper and lower limbs bilaterally. He had

For peer review only - http://bmjopen.bmj.com/site/about/guidelines.xhtml

fasciculation in the tongue and respiratory muscle weakness with a low oxygen saturation level (SpO_2, 86%). He also had memory disturbances accompanied by positive snout and palmomental reflexes and difficulty with mental activities of daily living. He had no sensory disturbance. We diagnosed him as having definite ALS with CI (Figure 3A).

Case ALS-P 1

A 65-year-old (in 2008) woman of the Auyu tribe, a resident of Pies along the Edera River, had a history of progressive motor weakness and gait disturbance for 5 years. She could not walk without support. Neurological examination revealed a resting tremor, cogwheel rigidity in the upper and lower limbs, bradykinesia throughout, postural impairment, UMN and LMN signs with a positive Babinski sign, hyperreflexia in the upper and lower limbs bilaterally, and thenar and hypothenar muscle atrophies. She had no CI or sensory disturbance. We diagnosed her as having ALS-parkinsonism and, more specifically, with severe parkinsonism (Hoen and Yahr IV) simultaneously complicated by motor neuron signs indicative of possible ALS. She died in 2009. Her younger sister was case P 6. The subject had ALS-parkinsonism, and her sister reported that their cousin showed tremor and gait disturbance, which were interpreted to be consistent with parkinsonism (Figure 3B).

Case ALS-P 2

A 76-year-old (in 2011) man of the Auyu tribe, a resident of Ogorito, had a history of tremor in both hands for 5 years in 2010. Neurological examination revealed cogwheel rigidity in his upper and lower limbs, bradykinesia, and UMN signs with hyperreflexia

For peer review only - http://bmjopen.bmj.com/site/about/guidelines.xhtml

in bilateral upper and lower limbs. We diagnosed him as having parkinsonism (Hoen and Yahr II) in 2010. He was examined again in 2011, by which time his parkinsonism was complicated by not only postural impairment but also muscle weakness and atrophy, and spasticity of the upper and lower limbs with Babinski signs. He had no CI or sensory disturbance. We diagnosed him as having parkinsonism (Hoen and Yahr III) with possible ALS; parkinsonism was accompanied by ALS later. He died in 2011 of unknown causes.

We also diagnosed another case of ALS-P3 parkinsonism (Hoen and Yahr III) with possible ALS; parkinsonism was accompanied by ALS later.

Case ALS-P-CI 6

A 50-year-old (in 2012) man of the Auyu tribe, a resident of Benggo along the Edera River, had a history of dysarthria for 2 years. Neurological examination revealed UMN and LMN signs with a positive Babinski sign, muscle atrophy, and weakness in the tongue, proximal upper limb, shoulder, and tibialis anterior muscles. He had cogwheel rigidity in the wrist and elbow, bradykinesia throughout, a positive Myerson sign, and impairment of coordination and postural reflex. He had memory loss, bradyphrenia, slow speech, and positive snout and palmomental reflexes with difficulty performing activities of daily living. We diagnosed him as having a definite ALS and parkinsonism (Hoen and Yahr III) with CI (Figure 3C).

Case P 7

For peer review only - http://bmjopen.bmj.com/site/about/guidelines.xhtml

A 52-year-old (in 2010) woman of the Auyu tribe who lived in Mopio along the Edera River had a history of progressive gait disturbance with parkinsonian signs, including resting tremor, cogwheel rigidity, anterior bending, and bradykinesia for 3 years in 2008. In 2008, we diagnosed her as having parkinsonism (Hoen and Yahr II). By the time of the follow-up survey in 2010, she could not stand by herself and had postural impairment with parkinsonian hands bilaterally. She had no UMN or LMN signs, no CI, and no sensory disturbance. We diagnosed her as having parkinsonism (Hoen and Yahr IV; Figure 3D).

DISCUSSION

Internal migration and migration from outside the province are the most significant causes of demographic, social, and cultural change in Papua. The populations in the survey sites were composed of former hunter-gatherers who lived in the forest and who were brought out of the forest and assembled in villages by Dutch colonialists. The villages along the Ia and Digul rivers were founded in 1940–1950.[10] The Dutch colonial program of population distribution from highly populated regions such as Java and Bali to Papua and other less populated province was continued by the Indonesian Government. The emigration program called transmigration (*transmigrasi*) started in Irian Jaya in 1964. Local migrants and transmigrants from outside Irian Jaya increased, resulting in various changes, including the use of food and medicine.[16,17] Considering that Gadjusek et al. found out the high prevalence of ALS at the first survey in 1962, the neurodegenerative disease existed before the introduction of manufactured items.[1]

For peer review only - http://bmjopen.bmj.com/site/about/guidelines.xhtml

In the present survey, we performed full neurological examinations in 46 subjects with ALS and/or parkinsonism between 2001 and 2012. Among the subjects, 17 were probable-definite cases of pure ALS, 13 were overlapping ALS (possible, probable, or definite) and parkinsonism, and 16 were parkinsonism. Nine cases of ALS and/or parkinsonism had a complication of CI. During the study period, the diagnosis in some patients changed from pure parkinsonism to ALS-parkinsonism, and their disease progressed in severity. Some subjects had both ALS and parkinsonism on their first examination. It is thus evident that overlapping ALS and parkinsonism with or without CI still exists in the specified region of Papua.

Gajdusek et al. reported diagnosing 97 cases (57 cases clinically confirmed and 40 cases not clinically confirmed) of ALS during the 6 surveys they conducted from 1962 to 1980.[1] However, they reported neurological findings of only 32 of these cases, in only 16 of which the reported findings of UMN, LMN, and parkinsonian signs justify a diagnosis of probable or definite ALS. The 16 cases comprise 12 cases of pure ALS and 4 cases of overlapping ALS-parkinsonism. Among the 32 ALS patients, three had a positive family history (9%); that is, 2 had sisters with ALS, and 1 who had ALS-parkinsonism had a family history of ALS. None of the ALS patients had a family history of parkinsonism. The authors also reported their neurological findings in 13 subjects in whom parkinsonism was diagnosed. They reported their findings from full neurological examinations of 10 of these parkinsonism cases; according to their reported neurological signs, these comprised 8 patients of parkinsonism, including 5 with dementia and 2 with overlapping ALS-parkinsonism with dementia.[1] Among the

For peer review only - http://bmjopen.bmj.com/site/about/guidelines.xhtml

For peer review only

13 parkinsonism patients, three had a positive family history (23%). In summary, we believe that in the 26 cases reported by Gajdusek et al., the full neurological examination findings comprised 12 pure ALS cases (probable or definite), 6 overlapping ALS-parkinsonism, and 8 parkinsonism with/without dementia.[1] Spencer et al reported 2 cases of ALS and three cases of parkinsonism, including 1 with dementia, in the survey in 1987. He also reported 3 subjects with parkinsonism overlapping UMN signs, including 1 with dementia in 1991. We have here reported 17 cases of pure ALS (probable or definite), 13 cases of overlapping ALS-parkinsonism, and 16 cases of parkinsonism with or without CI in the 46 cases we neurologically examined fully. Thus, we identified many cases of overlapping ALS and parkinsonism (n = 13, 28%) in our study and in previous reports (n = 6, 23% and n = 3, 38%).[1, 10] We also identified many cases with CI (n = 9, 20%) in our survey and in previous reports (n = 7, 27% and n = 2, 25%).[1, 10] Functional disabilities attributable to CI were recognized in activities of daily living or in their livelihood in all the cases with CI in this report.

Gajdusek et al. reported 13 cases of ALS and three cases of parkinsonism in 1975 in the villages along the Ia River (population, 2000).[1] The 13 ALS cases consisted of 10 clinically confirmed ones and 3 others that were not clinically confirmed. We compared the prevalence of probable-definite ALS and parkinsonism according to the report of Gajdusek et al. and our more present findings. A crude point prevalence of ALS consistent with clinically probable to definite ALS (El Escorial criteria) may be estimated between 500 (95% confidence interval, 191–809/100,000 people) and 650 (298–1002) and that of parkinsonism was 150 (0–320/100,000 people) in 1975 along

For peer review only - http://bmjopen.bmj.com/site/about/guidelines.xhtml

the Ia River. In our 2010 survey (population, 4500), we identified 6 probable-definite cases of ALS in this region (ALS 8, 10–13, and 17; 133 [27–-240]/100,000 people) and 7 of parkinsonism (possible ALS-P 2, 3, 9, and 13, and P 10, 15, and 16; 156 [40–271]/100,000 people).

Gajdusek et al. reported 8 cases of ALS and 6 cases of parkinsonism in 1975 in the villages along the Edera and Dumut rivers (population, 1950). The 8 cases of ALS consisted of 7 clinically confirmed cases and 1 unclinically confirmed case. The crude point prevalence of ALS consistent with clinically probable to definite ALS (El Escorial criteria) may be estimated between 359 (94–624)/100,000 people and 410 (127–694)/100,000 people, and that of parkinsonism was 308 (62–554)/100,000 people in 1975 along the Edera and Dumut rivers. In our survey of this region in 2010 (population, 4100), we identified 5 probable-definite cases of ALS (ALS 9, 14, and 16, and ALS-P 5, 6; 122 [15–229]/100,000 people) and 7 cases of parkinsonism (possible ALS-P 10 and 11, and P 7, 8, and 11–13; 171 [44–297]/100,000 people; Table 4). As this was not a complete inventory survey, the actual prevalence may be a little higher than our estimation.

The population of Bade increased by a factor of 4 during 31 years (from 1000 in 1975 to 3800 in 2006), and those of villages along the Ia River (from 2000 to 4500) and the Edera and Dumut rivers (from 1950 to 4100) have increased 2-fold.[1,15] The subjects born outside Papua were only 2 (Maluku) among the 46 subjects in this survey. The prevalence of the diseases among the local Papuan residents in this survey may be underestimated compared with the former report after taking into account the increase

For peer review only - http://bmjopen.bmj.com/site/about/guidelines.xhtml

in the numbers of local migrants inside and transmigrants from outside Papua.[16,17,18]

As the median age and life expectancy in the field sites in Papua are estimated to be lower than the global median and fewer people may live beyond the age of 40 or 50 years,[18,19] the prevalence of the diseases in this survey may be underestimated compared with the global mean. As the median age and life expectancy in 1975 may be lower than those in 2010,[20] the prevalence of the diseases in the report by Gajdusek et al. might be underestimated compared with that in our survey.

The prevalence of ALS in this report was found to be three times lower than found by Gajdusek et al. Even after taking into account the change of population and age structure in the field sites, our findings show that the prevalence of ALS is supposed to be decreased during the 35 years since the report of Gajdusek et al.[10] This is consistent with the previous report by Spencer et al. However, it is still much higher than the global mean (incidence, approximately 2/100,000; prevalence, approximately 6/100,000) [21-26]; in particular, the prevalence of overlapping ALS and parkinsonism is especially high in Papua.

In the report by Gajdusek et al., who provided village-by-village data showing the number of cases of ALS and parkinsonism, there were many cases in the remote villages such as Bosma, Pies, Homlikya, Yibin, Sien, and Ogorite, which were less visited and least developed.[1, 2] In this survey, many cases (three ALS/ALS-parkinsonism) were found in the remotest village of Ogorito along the Ia River. Two cases of ALS/ALS-parkinsonism were also found in Bosma, Homlikya, and

For peer review only - http://bmjopen.bmj.com/site/about/guidelines.xhtml

Yibin; 1 ALS and 2 cases of parkinsonism in Sien; 1 ALS and 1 parkinsonism in Pies, which consisted also of remote villages. While there were 8 cases of ALS/ALS-parkinsonism in Bade and 2 in Gimikya, nearest to the port of the Digul River, none was reported by Gajdusek et al. in those sites. This increase may be brought about by local migrants from the affected villages.

Overlap of ALS-parkinsonism and dementia has also been reported in patients with ALS/PDC in Guam and Kii, Japan.[4, 27-33] Hirano et al. provided clinicopathological evidence of 47 cases of PDC in Guam in 1961, comprising the following 3 groups: (1) 30 cases of PDC; (2) 8 cases of parkinsonism with dementia with clinical evidence of UMN impairment; and (3) 9 cases of parkinsonism with dementia with clinical evidence of UMN and LMN impairment. At least 15 of these cases (31.9%) had a family history of ALS, parkinsonism, or both.[4] Kuzuhara and Kokubo et al. surveyed neurological diseases in Kii, Japan, in the 1990s and found not only a continuously high incidence of ALS but also neuropathologically verified cases of PDC. ALS and PDC frequently affected the same individuals simultaneously and the members of the same family. These authors reported 37 cases of ALS/PDC between 1996 and 2006, a high proportion of whom (78%) had a positive family history, as well as clinical examples of pure ALS, PDC, and overlapping ALS and PDC. They also reported neuropathological changes common to cases of ALS and PDC in 12 cases of ALS/PDC, resembling those found in patients with ALS/PDC in Guam [28-33] In our more recent study in Papua, such as the previous studies in Kii, Japan, and Guam, we identified many cases of pure ALS and ALS overlapping with parkinsonism with/without CI. The prevalence of CI was lower in our study sites than in Guam and Kii.[4, 27-33] CI might have been underestimated,

For peer review only - http://bmjopen.bmj.com/site/about/guidelines.xhtml

and mild CIs or some mild dementia might have been overlooked by our neurological examinations in the field.

Our study was based only on clinical findings, and there are the limitations of not having electromyogram data, DNA analysis data, or any autopsy data from this population. Without such data, it is difficult to be certain how these patients fit into sporadic ALS or Parkinson disease and how they compare with ALS/PDC on Guam and in the Kii peninsula. However, we did identify many patients with overlapping ALS-parkinsonism in this study. The overlap of ALS and/or parkinsonism with CI suggests that it may be the same disease entity as the ALS/PDC diagnosed in Guam and Kii.

The proportion of males in the populations of Bade, the villages along the Ia River, and the villages along the Edera and Dumut rivers were 53%, 51%, and 50%. The proportion of males was high especially among the subjects with ALS-parkinsonism (85%) and ALS (59%) compared with among those with pure parkinsonism (50%) in our report. The proportion of males was higher among the subjects with ALS (63%) and parkinsonism (76%) in the report by Gadjusek et al., in which there were 3 male cases (75%) among the 4 ALS-parkinsonism cases. The high risks for ALS and PDC among the males were recognized in Guam and Kii.[1,7,27]

Indigenous people of Papua still retain aspects of their traditional lifestyles, such as hunting, gathering, fishing, and eating *sago* (trunk of *Metroxylon* sp.). They also drink water from shallow wells. However, with the spread of a market economy, recent increases in the numbers of rubber plantations and progress in transportation have been causing lifestyle changes in the villages in the south coastal area of Papua. As the

For peer review only - http://bmjopen.bmj.com/site/about/guidelines.xhtml

diseases are declining as in Guam and Kii, the disease aetiology in Papua may be dominated by environmental factors. Genetic factors may also play a role as evidenced by the family history of disease in 40% of the subjects in this survey in Papua and 80% in Kii.

Various possible causal genetic and environmental factors for these conditions have been reported. The environmental factors of low concentrations of calcium and magnesium in drinking water were hypothesized. [1,34] There is a recent report showing an association between the increase in ALS incidence and the change in drinking water source in the Kii Peninsula.[35] Spencer et al. pointed out that this explanation was inconsistent with a declining prevalence of ALS in spite of drinking water from shallow wells, which this report may support.[10]

Spencer et al. reported the discovery that cycad seed was used as a poultice in Papua, as in Guam. [36] Cycad-derived products were shown as the risk factor of dementia, mild CI, and PDC in Guam with the epidemiological evidence.[37] Although cycad had been suspected as the causal factor of Kii ALS,[38] people in Japan, including the Kii Peninsula, had never consumed cycad as daily food, although a small amount of cycad flour might have been taken by a few people as an ingredient of herb medicine.[39] We did not examine the association of prior skin wounds and exposure to cycad poultice with the diseases. Skin change and pigmentary retinopathy were not examined in this survey.[40, 41] Further studies are needed.

Genetic abnormalities that had been previously established to be associated with

For peer review only - http://bmjopen.bmj.com/site/about/guidelines.xhtml

familial or sporadic ALS, dementia, familial Parkinson disease, and parkinsonism were looked for in the cases in Kii, but none of them were identified.[28,42] Hermousura et al. have discovered a nuclear mutation in the calcium/magnesium membrane ion channel transient receptor potential melastatin 7 (*TRPM7*) gene in a subset of patients with ALS/PDC in Guam.[43] They reported that a TRPM7 variant that is associated with altered sensitivity to magnesium may have contributed to the pathogenesis of ALS and PDC in 2 Guamanians. *TRPM7* was not found to be associated with ALS/PDC in the Kii peninsula of Japan.[44] Ishiura et al. have recently discovered *C9ORF72* repeat expansion in some cases of ALS in the Kii peninsula of Japan; this repeat expansion partly accounts for the high prevalence of ALS in this region.[45] However, the responsible gene(s) remain unproved in almost all cases of ALS/PDC in both Guam and Kii.

Various environmental factors and multiple genetic factors are suggested to be underlying causes of these diseases, but these links are so far unproven.[33,46]

In Guam, although the incidence of pure ALS has declined markedly, the prevalence of PDC is still high.[47] The prevalence of dementia among elderly Chamorros (earliest known inhabitants of the Mariana Islands, which include Guam) is also reportedly relatively high.[48] In addition, in Kii the incidence of pure ALS has declined markedly, and the prevalence of PDC and ALS/PDC or ALS with dementia recently increased.[30,33] These changing patterns of ALS/PDC incidence in Guam and Kii might be caused by changes in environmental and socioeconomic factors as well as aging.[48,49]

For peer review only - http://bmjopen.bmj.com/site/about/guidelines.xhtml

The mean age of onset in our cases was 46 years for ALS, 53 years for ALS-parkinsonism, and 46 years for parkinsonism. Thus, they were older than the previously reported cases of ALS (33 years) and parkinsonism (43 years).[1] In our cases, the mean duration of subjective illness was 7.0 years for ALS, 6.3 years for ALS-parkinsonism, and 6.3 years for parkinsonism. The duration of ALS in our data was longer than not only the world mean of 3 years[21-26] but also the previously reported 3.5 years for ALS in Papua.[1] The mean age of onset may recently have increased, and the mean disease duration became longer in subjects with ALS. The prevalence rate of cases with bulbar sign was 37% in 30 of pure ALS or ALS-parkinsonian in our report, and it was as the same as in the report of Gadjdusek (40%) and classical ALS (39%).[2] The change in onset and course of ALS in recent years might be attributable to the aging population and changing environmental and socioeconomic factors.

On-going follow-up surveys should be performed to evaluate changes in the clinical types of ALS and/or parkinsonism with or without CI and accompanying changes in environmental and socioeconomic factors, as have occurred in Guam and Kii.

In conclusion, The prevalence of ALS is supposed to be decreased during the 35 years since the report of Gajdusek et al., when there has been an influx of migrants inside and transmigrants from outside Papua that has changed the population in the villages studied. However, there are still many cases of pure ALS and overlapping ALS-parkinsonism and CI, as has previously been reported in Guam and Kii. Environmental factors may play important etiologic role. Further on-going follow-up surveys focusing on the

For peer review only - http://bmjopen.bmj.com/site/about/guidelines.xhtml

clinical epidemiology and aetiology of these neurodegenerative diseases in association with ecological and environmental factors in Papua are needed. Future studies, including neuropathological examinations, should be performed when the local villagers in this region of Papua, Indonesia, are willing to consent to autopsies.

For peer review only

ACKNOWLEDGMENTS

The authors wish to thank all the participants in the villages who volunteered for the survey, the village leaders, and Mappi Regency officials. We are deeply indebted to doctors Imelda Mandagi, Reginald Nangoy, and Christha Z. Tamburian and their staff at Puskesmas Bade, and to Arifin Wasaraka, Lazarus Revasy, Yosefina Griapon, Arius Togodly, Kerry Yarangga, and Dolfinus Y. Bouway at Cenderawasih University for their invaluable contributions to the study. Andre Liem and Herry Wondiwoy at Papua Tour Guides Community and S.E. Amos at Bade High School provided logistic and language assistance. On the Japanese side, we acknowledge the technical support and advice of Katsuyuki Eguchi (Nagasaki University), Yasushi Osaki (Kochi University), Hidekazu Tomimoto (Mie University), Taro Yamauchi (The University of Tokyo), Hideyuki Onishi (Doshisha Women's College), Tetsuya Inamura (Aichi Prefectural University), Wenling Chen, Yumi Kimura, Yasuko Ishimoto, and Hissei Imai (Kyoto University), and Yasuyuki Kosaka, Yukiko Kita, and Chizu Wada (Research Institute for Humanity and Nature).

For peer review only - http://bmjopen.bmj.com/site/about/guidelines.xhtml

AUTHOR CONTRIBUTIONS

Okumiya had full access to all the data in the study and takes responsibility for the integrity of the data and the accuracy of the data analysis.

Study concept and design: Okumiya, Matsubayashi.

Acquisition of data: Okumiya, Wada, Fujisawa, Ishine, Garcia del Saz Hirata, Kuzuhara, Kokubo, Seguchi, Sakamoto, Manuaba, Matsubayashi.

Analysis and interpretation of data: Okumiya,Wada, Sakamoto, Fujisawa, Matusbayashi.

Drafting of the manuscript: Okumiya,Wada, Sakamoto, Fujisawa, Matusbayashi.

Critical revision of the manuscript for important intellectual content: All authors.

Statistical analysis: Okumiya.

Obtained funding: Okumiya, Wada, Kuzuhara, Kokubo, Matsubayashi

Administrative, technical, or material support: Okumiya, Watofa, Rantetampang, Matsubayashi.

Study supervision: Okumiya, Matsubayashi.

COMPETING INTERESTS : None declared

FUNDING

K. Okumiya receives research support from Grant-in-Aid for Scientific Research (A) from the Japan Society for the Promotion of Science (JSPS). K. Matsubayashi receives research support from Grant-in-Aid for Scientific Research (A) from the JSPS and is an editor of the journal *Geriatrics & Gerontology International*. T. Wada receives research

For peer review only

support from Grant-in-Aid for Scientific Research (C) from the JSPS. Y. Kokubo and S. Kuzuhara receive support from Grants-in-Aid from the Research Committee of CNS Degenerative Diseases and the Research Committee on the Muro disease (Kii amyotrophic lateral sclerosis/Parkinsonism-dementia), the Ministry of Health, Labour and Welfare of Japan.

REFERENCES

1. Gajdusek DC, Salazar AM. Amyotrophic lateral sclerosis and parkinsonian syndromes in high incidence among the Auyu and Jakai people of West New Guinea. Neurology 1982;**32**:107–26.
2. Gajdusek DC. Foci of motor neuron disease in high incidence in isolated populations of East Asia and the Western Pacific. In: Lewis P Rowland, ed. Human Motor Neuron Diseases. New York: Raven Press 1982:363–93.
3. Kurland LT, Mulder DW. Epidemiologic investigations of amyotrophic lateral sclerosis. 1. Preliminary report on geographic distribution and special reference to the Mariana Islands, including clinical and pathologic observations. Neurology 1954;**4**:438–48.
4. Hirano A, Kurland LT, Krooth RS, et al. Parkinsonism-dementia complex, an endemic disease on the island of Guam. I. Clinical features. Brain 1961;**84**:642–61.
5. Hirano A, Malamud N, Kurland LT. Parkinsonism-dementia complex, an endemic disease on the island of Guam. II. Pathological features. Brain 1961;**84**:662–79.
6. Kimura K, Yase Y, Higashi Y, et al. Epidemiological and geomedical studies on ALS and allied diseases in Kii peninsula (Japan): preliminary report. Proc Jpn Acad 1961;**37**:417–20.

For peer review only - http://bmjopen.bmj.com/site/about/guidelines.xhtml

7. Shiraki H, Yase Y. Amyotrophic lateral sclerosis in Japan. In: Vinken PJ, Bruyn GW, eds. Handbook of clinical neurology Vol. 22, System disorders and atrophies, Part II. Amsterdam: Elsevier Science 1975:353–419.

8. Garruto RM, Yanagihara R, Gajdusek DC. Disappearance of high incidence ALS and PDC on Guam. Neurology 1985;**35**:193–8.

9. Yoshida S. ALS tahatsuchi niokeru kankyoyoin to aluminium no neuron hensei eno kanyo nitsuite.[Environmental factors in western Pacific foci of ALS and a possible pathogenetic role of aluminum (Al) in motor neuron degeneration] (in Japanese with English abstract). Rinsho Shinkeigaku [Clin Neurol] 1991;**31**:1310–2.

10. Spencer P, Palmer VS, Ludolph AC. On the decline and etiology of high-incidence motor system disease in West Papua (Southwest New Guinea). Mov Disord 2005;**20(**Suppl 12):S119–126.

11. Wada T, Matsubayashi K, Okumiya K, et al. Health status and subjective economic satisfaction in West Papua. Lancet 2002;**360**:951.

12. Okumiya K, Fujisawa M, Ishine M, et al. Nishi New Guinea chiiki (Indonesia/ Papua shu) no shinkeihensei shikkan no jittai. 2001-2002 nen, 2006-2007 nen no fieldwork yori. [Fieldwork survey of neurodegenerative diseases in West New-Guinea in 2001-02 and 2006-07] (in Japanese). Rinsho Shinkeigaku [Clin Neurol] 2007;**47**:977–8.

13. Brooks BR. El Escorial World Federation of Neurology criteria for the diagnosis of amyotrophic lateral sclerosis: subcommittee on Motor Neuron Diseases/Amyotrophic Lateral Sclerosis of the World Federation of Neurology Research Group on Neuromuscular Diseases and the El Escorial "Clinical Limits of ALS" Workshop Contributors. J Neurol Sci 1994;**124**:96–107.

For peer review only

14. Hoehn MM, Yahr MD. Parkinsonism: onset, progression and mortality. Neurology. 1967;**17**:427–42.

15. Kontor Statistik Distrik Edera. Laporan Kependudukan, Kabupaten Mappi Distrik Edera 2006 (December).

16. Manning C., Rumiak M. Irian Jaya: Economic change, migrants, and indinenous welfare. In Hill H ed. Unity and diversity: Regional Economic Development in Indonesia since 1970. Oxford University Press, Okford.p77-106, 1991.

17. Timmer J. A brief social and political history of Papua, 1962-2005. In Marshall AJ and Beehler BM ed. The Ecology of Indonesian Papua Part Two. Periplus Editions, Hong Kong. p1098-1124, 2007.

18. Badan Pusat Statistik, Sensus Penduduk 2010. http://sp2010.bps.go.id/index.php

19. United Nations , Department of Economic and Social Affairs, Population Division. World Population Prospects The 2012 Revision *Volume II: Demographic Profiles,* http://esa.un.org/unpd/wpp/index.htm

20. Kontor Statistik Kabupaten Merauke. Laporan Kependudukan, Kabupaten Merauke 1997

21. Armon C. Amyotrophic lateral sclerosis. In: Neuroepidemiology from principles to practice. USA: Oxford University Press 2004:162–87.

22. McGuire V, Longstreth WT Jr, Koepsell TD, et al. Incidence of amyotrophic lateral sclerosis in three counties in western Washington state. Neurology 1996 ;**47**:571–3.

23. Sorenson EJ, Stalker AP, Kurland LT, et al. Amyotrophic lateral sclerosis in Olmsted County, Minnesota, 1925 to 1998. Neurology 2002; **23**:280–2.

24. O'Toole O, Traynor BJ, Brennan P, et al. Epidemiology and clinical features of amyotrophic lateral sclerosis in Ireland between 1995 and 2004. J Neurol Neurosurg

For peer review only - http://bmjopen.bmj.com/site/about/guidelines.xhtml

Psychiatry 2008;**79**:30–2.

25. Chiò A, Mora G, Calvo A, et al. Epidemiology of ALS in Italy: a 10-year prospective population-based study. Neurology 2009;**72**:725–31.

26. Factor-Litvak P, Al-Chalabi A, Ascherio A, et al. Current pathways for epidemiological research in amyotrophic lateral sclerosis. Amyotroph Lateral Scler Frontotemporal Degener 2013;**14**(Suppl 1):33–43.

27. Rodgers-Johnson P, Garruto RM, Yanigahara R, et al. Amyotrophic lateral sclerosis and parkinsonism-dementia on Guam: a 30-year evaluation of clinical and neurological trends. *Neurology.* 1986;36:7-13.

28. Kuzuhara S, Kokubo Y, Sasaki R, et al. Familial amyotrophic lateral sclerosis and parkinsonism-dementia complex of the Kii peninsula of Japan: clinical and neuropathological study and tau analysis. Ann Neurol 2001;**49**:501–11.

29. Kuzuhara S, Kokubo Y. Atypical parkinsonism of Japan: amyotrophic lateral sclerosis-parkinsonism-dementia complex of the Kii peninsula of Japan (Muro disease): an update. Mov Disord 2005;**20**(Suppl 12):S108–13.

30. Kuzuhara S. Kii ALS/PDC (Murou byou) no rekishi, gainen, ekigaku. [ALS-parkinsonism-dementia complex of the Kii peninsula of Japan (Muro disease). Historical review, epidemiology and concept] (in Japanese with English abstract). Rinsho Shinkeigaku [Clin Neurol] 2007;**47**:962–5.

31. Kokubo Y. Kiihanto no kinishukuseisokusakukokasho/ parkinson chihou hukugo no rinshou, shinkei byourigakuteki kenkyu. [Clinical aspects, imaging and neuropathology of Kii ALS/PDC] (in Japanese with English abstract). Rinsho Shinkeigaku [Clin Neurol] 2007;**47**:966–9.

32. Kuzuhara S. Kii hanto no Murobyo (Kinishukuseisokusakukokasho/ parkinson/

For peer review only - http://bmjopen.bmj.com/site/about/guidelines.xhtml

ninchisho hukugo) [Muro disease or ALS–parkinsonism–dementia complex of the Kii peninsula of Japan] (in Japanese with English abstract). Rinsho Shinkeigaku [Clin Neurol] 2007;**47**:695–702.

33. Kuzuhara S, KokuboY. Amyotrophic lateral sclerosis-parkinsonism-dementia complex in the Kii peninsula of Japan (Muro disease): a review on recent research and new concept. In: Michael J. Strong, ed. Amyotrophic lateral sclerosis and the frontotemporal dementias. Oxford: Oxford University Press 2012:39–54.
34. Yase Y. The pathogenesis of amyotrophic lateral sclerosis. Lancet 1972;**2**(7772):292–6.
35. Kihara T, Yoshida S, Kondo T et al. An increase in ALS incidence on the Kii Peninsula, 1960-2009: A possible link to change in drinking water source. *Amyotrophic Lateral Sclerosis,* 2012; 13: 347–50.
36. Spencer PS, Nunn PB, Hugon J, et al. Guam amyotrophic lateral sclerosis-parkinsonism-dementia linked to a plant excitant neurotoxin. Science 1987;**237**(4814):517–22.
37. Borenstein AR, Mortimer JA, Schofield E. et al. Cycad exposure and risk of dementia, MCI, and PDC in the Chamorro population of Guam. Neurology. 2007;68:1764-71.
38. Spencer PS, Ohta M, Palmer VS. Cycad use and motor neuron disease in Kii peninsula of Japan. Lancet 1987;**2**(8573):1462–3.
39. Iwami O, Niki Y, Watanabe T, and Ikeda M. Motor neuron disease on the Kii Peninsula of Japan: Cycad exposure. Neuroepidemiology 1993;12:307-312.
40. Ono S, Waring SC, Kurland L et al. Guamanian neurodegenerative disease: ultrastructural studies of skin J. Neurol. Sci., 146 (1997), pp. 35–40
41. Kokubo Y, Ito K, Kuzuhara S. Ophthalmomyiasis-like pigmentary retinopathy in

For peer review only - http://bmjopen.bmj.com/site/about/guidelines.xhtml

ALS/PDC in the Kii peninsula of Japan. Neurology. 2003 May 27;60(10):1725-6.

42. Tomiyama H, Kokubo Y, Sasaki R, et al. Mutation analyses in amyotrophic lateral sclerosis/parkinsonism-dementia complex of the Kii peninsula, Japan. Mov Disord 2008;**23**(16):2344–8.

43. Hermosura MC, Nayakanti H, Dorovkov MV, et al. A TRPM7 variant shows altered sensitivity to magnesium that may contribute to the pathogenesis of two Guamanian neurodegenerative disorders. Proc Natl Acad Sci 2005;**102**(32):11510–5.

44. Hara K, Kokubo Y, Ishiura H, et al. TRPM7 is not associated with amyotrophic lateral sclerosis-parkinsonism dementia complex in the Kii peninsula of Japan. Am J Med Genet B Neuropsychiatr Genet 2010;**153B**(1):310–3.

45. Ishiura H, Takahashi Y, Mitsui J, et al. *C9ORF72* Repeat expansion in amyotrophic lateral sclerosis in the Kii peninsula of Japan. Arch Neurol 2012;**69**(9):1154–8.

46. Garruto RM, Yanagihara R. Contributions of isolated Pacific populations to understanding neurodegenerative diseases. Folia Neuropathol 2009;**47**(2):149–70.

47. Plato CC, Garruto RM, Galasko D, et al. Amyotrophic lateral sclerosis and parkinsonism-dementia complex of Guam: changing incidence rates during the past 60 years. Am J Epidemiol 2003;**157**:149–57.

48. Galasko D, Salmon D, Gamst A, et al. Prevalence of dementia in Chamorros on Guam: relationship to age, gender, education, and APOE. Neurology 2007;**68**:1772–81.

49. Plato CC, Galasko D, Garruto RM, et al. ALS and PDC of Guam: forty-year follow-up. Neurology 2002;**58**:765–73.

For peer review only - http://bmjopen.bmj.com/site/about/guidelines.xhtml

Figure and table legends

Table 1. Relevant characteristics and neurological signs of cases with ALS with/without cognitive impairment in Papua, 2001–2012.

Abbreviations: ALS = amyotrophic lateral sclerosis; BK = bradykinesia; BS = Babinski sign; DTR = deep tendon reflex; Dys = dysphagia/dysarthria; F= female; Fasc = fasciculation; LMN = lower motor neuron signs; M = male; MA = muscle atrophy with weakness; NC = not confirmed by examination; P = parkinsonism; PI = postural impairment; R = rigidity; T = tremor; TA = tongue atrophy; U/L: upper limbs/lower limbs; UMN = upper motor neuron signs.

Age: at the time of final survey; Duration: between subjective onset of illness and last survey or death; Alive: alive in 2012; Onset: subjective onset of the illness; Deep tendon reflexes, -: absent, 1: hypoactive, 2: normal, 3: hyperactive, 4: markedly hyperactive with ankle clonus.

Other signs are classified as: -: absent; ±: questionably present; +: present; ++: markedly present.

Family history 1: cousin (possible ALS); 2: grandfather (NC); 3: sister (ALS15); 4: cousin's daughter (P16); 5: cousin (NC); 6: father (NC); 7: sister (ALS 7); 8: sister (P 6) and cousin (NC); 9: father (NC); 10: son: (NC); 11: sister (ALS-P 1) and cousin (NC); 12: mother and father (NC); 13: grandmother (NC); 14: mother (NC); 15: ALS 10 (mother’s cousin).

For peer review only - http://bmjopen.bmj.com/site/about/guidelines.xhtml

Table 2. Relevant characteristics and neurological signs in cases with overlap of ALS and parkinsonism with/without cognitive impairment in Papua, 2001–2012.

Table 3. Relevant characteristics and neurological signs in cases with parkinsonism with/without cognitive impairment in Papua, 2001–2012.

Table 4. Cases of ALS and parkinsonism according to field site

*; Active cases in 2007 in Bade

#; Active cases in 2010 in the villages along the Ia, Edera, and Dumut rivers

Figure 1. Map of the field sites

A. Large-scale map of the southern coastal plain in Papua, Indonesia.

B. Smaller scale map of the southern coastal plain in Papua, Indonesia showing rivers.

C. Detailed map showing villages in the southern coastal area in Papua in which all confirmed cases of ALS and/or parkinsonism with/without cognitive impairment were seen from 2001 to 2012.

Figure 2. Photographs of patient with ALS (Case ALS 12**)**

A: The patient needs assistance from family members to stand.

B: Advanced atrophy of the tongue.

C: There is upper limb-girdle and truncal muscle atrophy with a positive Babinski sign.

D: Advanced thenar muscle atrophy.

Figure 3. Photographs of patients with ALS, ALS-parkinsonism, and parkinsonism

For peer review only - http://bmjopen.bmj.com/site/about/guidelines.xhtml

A: Patient with ALS (Case ALS-17) showing muscle atrophy in the distal upper and lower limbs.

B: Patient with ALS-parkinsonism (Case ALS-P 1) showing muscle atrophy in distal upper limbs.

C: Patient with ALS-parkinsonism (Case ALS-P 7) showing atrophy in muscles in the proximal upper limb, shoulder, and tibialis anterior.

D: Patient with parkinsonism (Case P 7) showing typical parkinsonian hands and anterior bending posture.

Parkinsonian hand: Striatal deformities of the hand with abnormal postures that are common in patients with advanced Parkinson's disease.

For peer review only

For peer review only - http://bmjopen.bmj.com/site/about/guidelines.xhtml

Okumiya

Table 1. Relevant characteristics and neurological signs in cases with pure ALS with/without cognitive impairment in Papua, 2001–2012.

Case number	ALS criteria Yahr stage	Survey year	Village	Age	Sex	Onset (age/year)	Duration (years)	Death (year)	Tribe (origin)	UMN signs DTR U/L	UMN signs BS	LMN signs MA U/L	LMN signs Fasc U/L	Bulbar signs TA	Bulbar signs Dys	Parkinsonian sign T U/L	Parkinsonian sign R U/L	Parkinsonian sign BK	Parkinsonian sign PI	Cognitive impairment	Family history
Pure ALS (n=17)																					
ALS 1	Definite	2001	Bade	35	M	NC	NC	NC	Auyu	3/3	+	++/++	+/+	++	-	-	-	-	-	-	-
ALS 2	Definite	2001	Ogoto	43	F	NC	NC	2002	Auyu	3/3	+	++/++	+/+	++	++	-	-	-	-	-	-
ALS 3	Definite	2002	Senggo	50	M	48/'00	2	NC	Citak	3/3	+	++/++	±/±	±	+	-	-	-	-	-	1
ALS 4	Definite	2007	Homilikia	53	M	51/'05	2	2007	Auyu	3/2	+	++/++	+/+	-	-	-	-	-	-	-	-
ALS 5	Definite	2007/'08	Bade	51	M	49/'06	3	2009	Auyu	3/4	+	+/-	-/-	-	+	-	-	-	-	-	-
ALS 6	Definite	2008	Bade	21	M	11/'98	11	2009	Auyu	4/4	+	++/++	-/-	-	-	-	-	-	-	-	2
ALS 7	Definite	2007/'08/'10	Bade	70	F	57/'97	13	2010	Maluku	3/3	+	++/++	+/-	-	+	-	-	-	-	-	3
ALS 8	Probable	2011	Geturki	53	M	48/'06	5	Alive	Auyu	3/3	-	+/+	-/+	-	-	-	-	-	-	-	-
ALS 9	Definite	2011	Muya	41	F	36/'06	6	2012	Auyu	3/3	+	+/+	-/-	-	-	-	-	-	-	-	-
ALS 10	Probable	2007/'10/'11/'12	Osso	65	F	59/'06	6	Alive	Auyu	3/4	+	+/-	-/-	+	-	-	-	-	-	-	4
ALS 11	Probable	2007/'08/'10/'12	Gimikya	32	M	12/'92	20	Alive	Auyu	4/4	+	++/+	++/-	±	-	-	+/-	-	-	-	5
ALS 12	Definite	2010/'11/'12	Bosma	64	M	53/'01	11	Alive	Auyu	3/4	+	++/++	+/-	++	-	-	-	-	-	-	6
ALS 13	Definite	2012	Asset	70	F	68/'10	2	Alive	Auyu	3/3	-	+/+	-/-	-	-	-	-	-	-	-	-
ALS 14	Probable	2012	Pies	65	F	60/'07	5	Alive	Auyu	3/4	-	+/+	-/-	-	-	-	-	-	-	-	-
ALS 15	Definite	2007	Bade	64	F	62/'05	2	2007	Maluku	3/3	+	++/++	-/-	-	+	-	-	-	-	+	7
ALS 16	Definite	2008/'10/'11	Yibin	25	M	13/'99	13	2012	Auyu	3/4	+	++/+	++/++	-	-	-	-	-	-	+	-
ALS 17	Definite	2012	Ogorito	70	M	66/'08	4	Alive	Auyu	3/3	+	+/+	+/+	-	-	-	-	-	-	+	-

Abbreviations: ALS = amyotrophic lateral sclerosis; BK = bradykinesia; BS = Babinski sign; DTR = deep tendon reflex; Dys = dysphagia/dysarthria; F = female; Fasc = fasciculation; LMN = lower motor neuron signs; M = male; MA = muscle atrophy with weakness; NC = not confirmed by examination; P = parkinsonism; PI = postural impairment; R = rigidity; T = tremor; TA = tongue atrophy; U/L: upper limbs/lower limbs; UMN = upper motor neuron signs.
Age: at the time of final survey; Duration: between subjective onset of illness and last survey or death; Alive: alive in 2012; Onset: subjective onset of illness; Deep tendon reflexes, -: absent, 1: hypoactive, 2: normal, 3: hyperactive, 4: markedly hyperactive with ankle clonus.
Other signs are classified as: -: absent; ±: questionably present; +: present; ++: markedly present.
Family history 1: cousin (possible ALS); 2: grandfather (NC); 3: sister (ALS15); 4: cousin's daughter (P16); 5: cousin (NC); 6: father (NC); 7: sister (ALS 7); 8: sister (P 6) and cousin (NC); 9: father (NC); 10: son: (NC); 11: sister (ALS-P 1) and cousin (NC); 12: mother and father (NC); 13: grandmother (NC); 14: mother (NC); 15: ALS 10 (mother's cousin).

For peer review only - http://bmjopen.bmj.com/site/about/guidelines.xhtml

Table 2. Relevant characteristics and neurological signs in cases with overlap of ALS and parkinsonism with/without cognitive impairment in Papua, 2001–2012.

Case number	ALS criteria Yahr stage	Survey year	Village	Age	Sex	Onset (age/year)	Duration (year)	Death (year)	Tribe (origin)	UMN signs: DTR U/L	UMN signs: BS	LMN signs: MA U/L	LMN signs: Fasc U/L	Bulbar signs: TA	Bulbar signs: Dys	Parkinsonian sign: T U/L	Parkinsonian sign: R U/L	Parkinsonian sign: BK	Parkinsonian sign: PI	Cognitive impairment	Family history
ALS-parkinsonism (n = 13)																					
ALS-P 1	Possible-IV	2008	Pies	65	F	60/'03	6	2009	Auyu	3/3	+	+/-	-/-	-	-	+/+	++/++	++	+	-	8
ALS-P 2	Possible-III	2010/'11	Ogorito	76	M	70/'05	6	2011	Auyu	3/3	+	+/±	-/-	-	-	+/+	+/+	+	+	-	-
ALS-P 3	Possible-III	2010/'11/'12	Homilikia	69	M	65/'08	4	Alive	Auyu	3/2	+	+/-	-/-	-	-	+/+	+/+	+	+	-	-
ALS-P 4	Definite-III	2008	Bade	34	M	18/'92	17	2009	Muyu	3/3	+	+/-	-/-	++	++	-	+/+	+	+	+	9
ALS-P 5	Definite-III	2012	Sien	60	M	58/'10	2	Alive	Auyu	3/3	+	+/+	-/-	-	-	-	+/+	+	+	+	-
ALS-P 6	Definite-III	2012	Benggo	50	M	48/'10	2	Alive	Auyu	2/2	+	+/+	-/-	+	+	-	+/-	+	+	+	-
ALS-P 7	Possible-II	2001	Bade	36	M	NC	NC	NC	Auyu	3/3	+	-/-	-/-	-	-	+	+	+	-	-	-
ALS-P 8	Possible-II	2008/'10	Bade	23	M	11/'98	12	Alive	Auyu	3/4	+	-/-	-/-	-	-	+/+	+/±	+	-	-	-
ALS-P 9	Possible-III	2010/'11	Ogorito	76	M	70/'05	6	2011	Auyu	3/3	+	-/-	-/-	-	-	+/-	+/+	+	+	-	-
ALS-P 10	Possible-III	2008/'10/'11	Yibin	52	M	44/'03	8	Alive	Auyu	3/3	+	-/-	-/-	-	-	+/-	+/+	+	+	-	-
ALS-P 11	Possible-III	2011	Getio	55	F	53/09	2	2011	Auyu	3/3	+	±/-	-/-	-	-	+/+	+/+	+	+	-	-
ALS-P 12	Possible-IV	2001	Bade	40	M	NC	NC	NC	Auyu	3/3	+	-/-	-/-	-	+	+	+	+	+	+	-
ALS-P 13	Possible-III	2012	Bosma	52	M	48/'08	4	Alive	Auyu	3/4	+	-/-	-/-	-	-	-	++/+	+	+	+	-

Abbreviations: ALS = amyotrophic lateral sclerosis; BK = bradykinesia; BS = Babinski sign; DTR = deep tendon reflex; Dys = dysphagia/dysarthria; F = female; Fasc = fasciculation; LMN = lower motor neuron signs; M = male; MA = muscle atrophy with weakness; NC = not confirmed by examination; P = parkinsonism; PI = postural impairment; R = rigidity; T = tremor; TA = tongue atrophy; U/L: upper limbs/lower limbs; UMN = upper motor neuron signs.

Age: at the time of final survey; Duration: between subjective onset of illness and last survey or death; Alive: alive in 2012; Onset: subjective onset of illness; Deep tendon reflexes, -: absent, 1: hypoactive, 2: normal, 3: hyperactive, 4: markedly hyperactive with ankle clonus.

Other signs are classified as: -: absent; ±: questionably present; +: present; ++: markedly present.

Family history 1: cousin (possible ALS); 2: grandfather (NC); 3: sister (ALS15); 4: cousin's daughter (P16); 5: cousin (NC); 6: father (NC); 7: sister (ALS 7); 8: sister (P 6) and cousin (NC); 9: father (NC); 10: son: (NC); 11: sister (ALS-P 1) and cousin (NC); 12: mother and father (NC); 13: grandmother (NC); 14: mother (NC); 15: ALS 10 (mother’s cousin).

For peer review only

Table 3. Relevant characteristics and neurological signs in cases with parkinsonism with/without cognitive impairment in Papua, 2001–2012.

Case number	ALS criteria Yahr stage	Survey year	Village	Age	Sex	Onset (age/year)	Duration (years.)	Death (year)	Ethnicity	UMN signs		LMN signs		Bulbar signs		Parkinsonian sign				Cognitive impairment	Family history
										DTR U/L	BS	MA U/L	Fasc U/L	TA	Dys	T U/L	R U/L	BK	PI		
Parkinsonism (n = 16)																					
P 1	Yahr IV	2001	Kepi	50	M	NC	NC	NC	Jakai	2/2	-	-/-	-/-	-	-	+/+	+/+	++	+	-	-
P 2	Yahr II	2001	Kepi	50	M	NC	NC	NC	Jakai	2/2	-	-/-	-/-	-	-	+	+	+	-	-	-
P 3	Yahr IV	2002	Emete	52	M	49/'99	3	NC	Jakai	2/2	-	-/-	-/-	-	-	+	+	+	+	-	-
P 4	Yahr II	2002	Senggo	41	M	36/'97	5	NC	Citak	2/2	-	-/-	-/-	-	-	+	+	-	-	-	-
P 5	Yahr I	2007	Mur	65	F	61/'03	4	NC	Jakai	2/3	-	-/-	-/-	-	-	+/-	+/-	-	-	-	10
P 6	Yahr IV	2008	Pies	60	F	55/'03	6	2009	Auyu	2/2	-	-/-	-/-	-	-	+/+	+/+	++	+	-	11
P 7	Yahr IV	2008/'10	Mopio	52	F	47/'05	5	Alive	Auyu	3/3	-	-/-	-/-	-	-	++/+	+/+	++	+	-	-
P 8	Yahr II	2011	Sien	57	F	49/03	9	2012	Auyu	3/3	-	-/-	-/-	-	-	+/+	+/+	+	-	-	12
P 9	Yahr IV	2007/'08/'10/'[illegible]	Bade	46	F	40/'06	6	Alive	Auyu	3/3	-	-/-	-/-	-	-	+/+	++/+	+	+	-	13
P 10	Yahr II	2011/'12	Asset	38	M	27/'01	11	Alive	Auyu	3/3	-	-/-	-/-	-	-	+/-	+/-	+	-	-	-
P 11	Yahr III	2011/'12	Sien	54	F	40/'98	14	Alive	Auyu	3/3	-	-/-	-/-	-	-	++/+	++/+	+	+	-	-
P 12	Yahr II	2011/'12	Memes	66	F	64/10	2	Alive	Auyu	3/3	-	-/-	-/-	-	-	+/+	+/+	+	-	-	14
P 13	Yahr II	2012	Harapan	60	M	57/09	3	Alive	Auyu	2/2	-	-/-	-/-	-	-	-	+/+	-	-	-	-
P 14	Yahr II	2007	Mur	50	M	38/'95	12	NC	Auyu	2/4	+	-/-	-/-	-	-	+	+	+	-	-	-
P 15	Yahr I	2007	Homilikia	50	M	NC	NC	Alive	Auyu	2/3	+	-/-	-/-	-	-	+/-	+/±	-	-	-	-
P 16	Yahr III	2011	Osso	35	F	33/'09	2	2011	Auyu	3/2	-	-/-	-/-	-	-	+/+	+/+	+	+	+	15

Abbreviations: ALS = amyotrophic lateral sclerosis; BK = bradykinesia; BS = Babinski sign; DTR = deep tendon reflex; Dys = dysphagia/dysarthria; F = female; Fasc = fasciculation; LMN = lower motor neuron signs; M = male; MA = muscle atrophy with weakness; NC = not confirmed by examination; P = parkinsonism; PI = postural impairment; R = rigidity; T = tremor; TA = tongue atrophy; U/L: upper limbs/lower limbs; UMN = upper motor neuron signs.

Age: at the time of final survey; Duration: between subjective onset of illness and last survey or death; Alive: alive in 2012; Onset: subjective onset of illness; Deep tendon reflexes, -: absent, 1: hypoactive, 2: normal, 3: hyperactive, 4: markedly hyperactive with ankle clonus.

Other signs are classified as: -: absent; ±: questionably present; +: present; ++: markedly present.

Family history 1: cousin (possible ALS); 2: grandfather (NC); 3: sister (ALS15); 4: cousin's daughter (P16); 5: cousin (NC); 6: father (NC); 7: sister (ALS 7); 8: sister (P 6) and cousin (NC); 9: father (NC); 10: son: (NC); 11: sister (ALS-P 1) and cousin (NC); 12: mother and father (NC); 13: grandmother (NC); 14: mother (NC); 15: ALS 10 (mother’s cousin).

For peer review only

For peer review only - http://bmjopen.bmj.com/site/about/guidelines.xhtml

Table 4. Cases of ALS and parkinsonism in the field sites

	ALS criteria	Case No.	Crude point prevalence rate (95%CI) among ALS/ ALS-P/ Parkinsonism (2007* or 2010#)
Bade 2007–2012 (population 3800)			
ALS	Definite	ALS 5*	105 (2-208) /100,000 in 2007*
(n=4)	Definite	ALS 6*	
	Definite	ALS 7*	
	Definite	ALS 15*	
ALS-parkinsonism	Definite	ALS-P 4*	53 (0-126) /100,000 in 2007*
(n=2)	Possible	ALS-P 8*	
Parkinsonism (n=1)		P 9*	26 (0-78) /100,000 in 2007*
Ia river 2007–2012 (population 4500)			
ALS	Definite	ALS 4	133(27-240) /100,000 in 2010#
(n=7)	Probable	ALS 8#	
	Probable	ALS 10#	
	Probable	ALS 11#	
	Definite	ALS 12#	
	Definite	ALS 13#	
	Definite	ALS 17#	
ALS-parkinsonism	Possible	ALS-P 2#	89 (2-176) /100,000 in 2010#
(n=4)	Possible	ALS-P 3#	
	Possible	ALS-P 9#	
	Possible	ALS-P 13#	
Parkinsonism		P 10#	67 (0-142) /100,000 in 2010#
(n=3)		P 15#	
		P 16#	
Edera and Dumut rivers 2008–2012 (population: 4100)			
ALS	Definite	ALS 9#	73 (0-156) /100,000 in 2010#
(n=3)	Probable	ALS 14#	
	Definite	ALS 16#	
ALS-parkinsonism	Possible	ALS-P 1	98 (2-193) /100,000 in 2010#
(n=5)	Definite	ALS-P 5#	
	Definite	ALS-P 6#	
	Possible	ALS-P 10#	
	Possible	ALS-P 11#	
Parkinsonism		P 6	122 (15-229) /100,000 in 2010
(n=6)		P 7#	
		P 8#	
		P 11#	
		P 12#	
		P 13#	

*:Active cases in 2007 in Bade
#:Active cases in 2010 in the villages along Ia, Edera and Dumut rivers
95%CI: 95% confidence interval

For peer review only - http://bmjopen.bmj.com/site/about/guidelines.xhtml

For peer review only

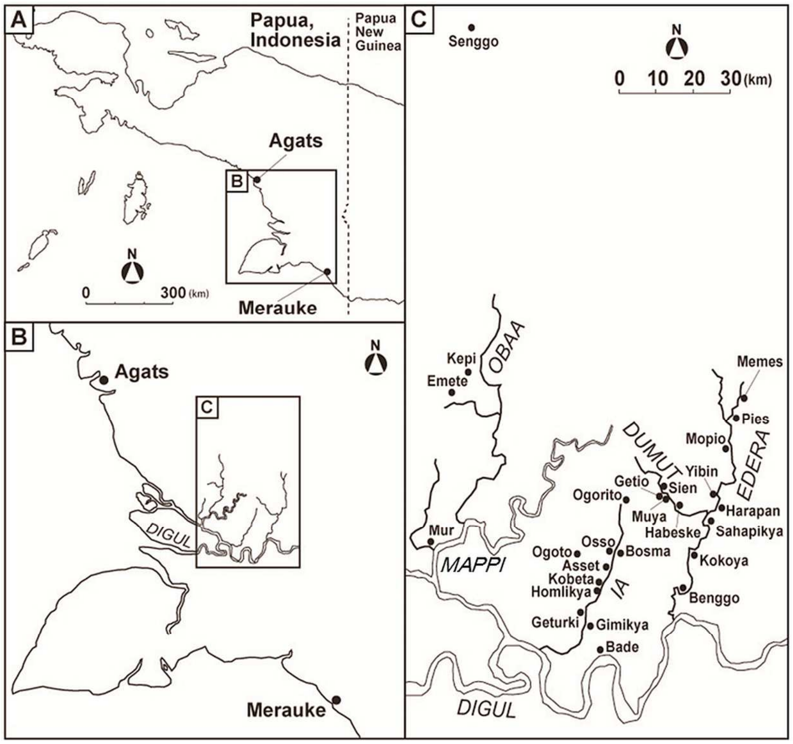

119x90mm (300 x 300 DPI)

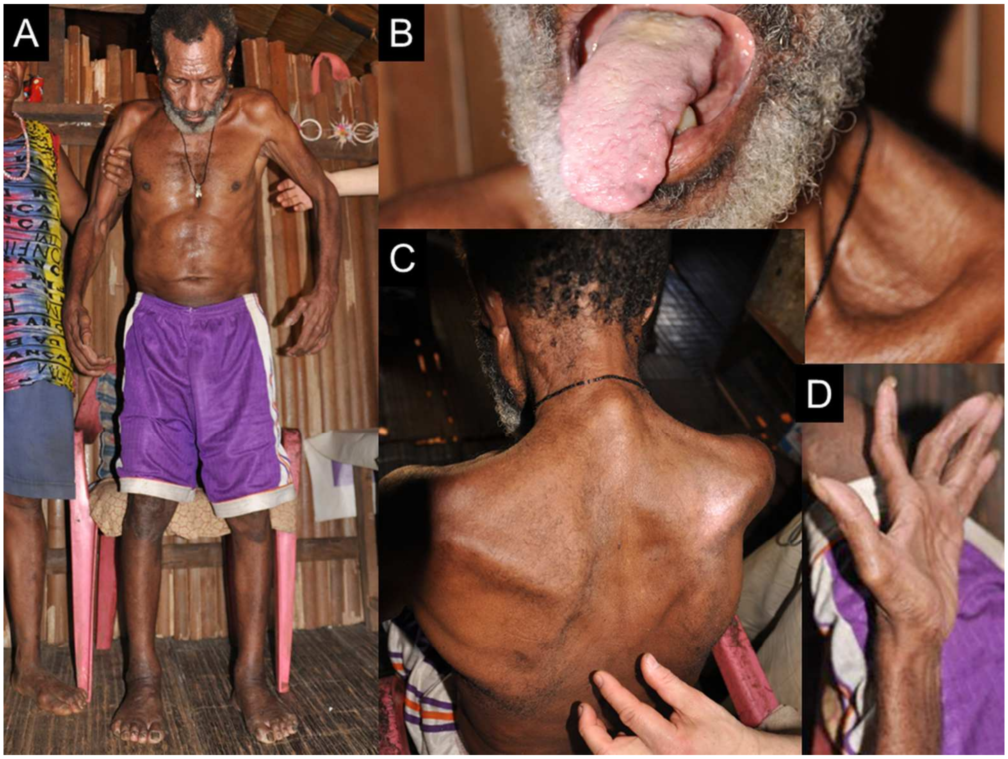

119x90mm (300 x 300 DPI)

For peer review only

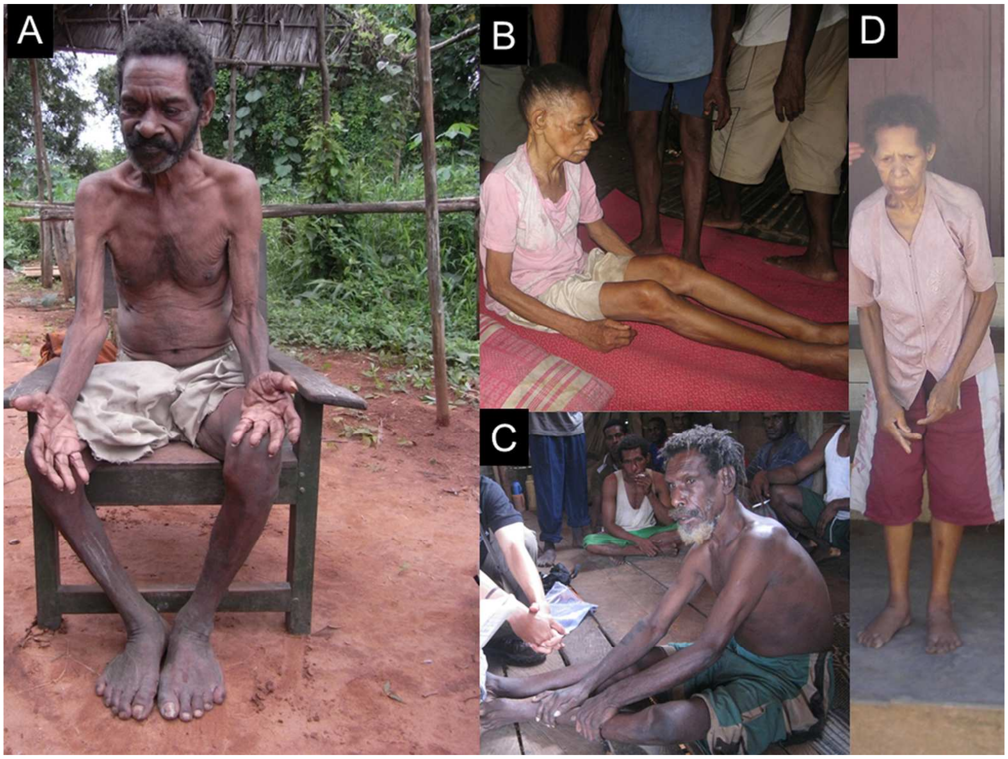

119x90mm (300 x 300 DPI)

For peer review only

Amyotrophic lateral sclerosis and parkinsonism in Papua, Indonesia: 2001–2012 survey results

Journal:	*BMJ Open*
Manuscript ID:	bmjopen-2013-004353.R2
Article Type:	Research
Date Submitted by the Author:	03-Mar-2014

Complete List of Authors:	Okumiya, Kiyohito; Research Institute for Humanity and Nature,<br>Wada, Taizo; Kyoto University, Center for Southeast Asian Studies<br>Fujisawa, Michiko; Center for Southeast Asian Studies,<br>Ishine, Masayuki; Yasugi Clinic,<br>Garcia del Saz, Eva; Kochi University, Center for Regional & International Collaboration<br>Hirata, Yutaka; Kitaakita Municipal Hospital,<br>Kuzuhara, Shigeki; Suzuka University of Medical Science, Faculty of Health Science<br>Kokubo, Yasumasa; Mie University, Graduate School of Regional Innovation Studies<br>Seguchi, Harumichi; Kochi University, Faculty of Medicine<br>Sakamoto, Ryota; Kyoto University, Hakubi Center for Advanced Research<br>Manuaba, Indrajaya; Cenderawasih University, Faculty of Medicine<br>Watofa, Paulina; Cenderawasih University, Faculty of Medicine<br>Rantetampang, Andreas; Cenderawasih University, Faculty of Public Health<br>Matsubayashi, Kozo; Kyoto University, Center for Southeast Asian Studies

**Primary Subject Heading**:	Epidemiology
Secondary Subject Heading:	Neurology
Keywords:	Motor neurone disease < NEUROLOGY, Parkinson-s disease < NEUROLOGY, Dementia < NEUROLOGY

SCHOLARONE™
Manuscripts

Okumiya

Amyotrophic lateral sclerosis and parkinsonism in Papua, Indonesia: 2001–2012 survey results

Kiyohito Okumiya[1,2], Taizo Wada[2], Michiko Fujisawa[2], Masayuki Ishine[3], Eva Garcia del Saz[4], Yutaka Hirata[5], Shigeki Kuzuhara[6], Yasumasa Kokubo[7], Harumichi Seguchi[8], Ryota Sakamoto[9], Indrajaya Manuaba[10], Paulina Watofa[10], Andreas L. Rantetampang[11], Kozo Matsubayashi[2]

[1]Research Institute for Humanity and Nature, Kyoto, Japan
[2]Center for Southeast Asian Studies, Kyoto University, Kyoto, Japan
[3]Yasugi Clinic, Shimane, Japan
[4]Center for Regional & International Collaboration, Kochi University, Kochi, Japan
[5]Kitaakita Municipal Hospital, Akita, Japan
[6]Faculty of Health Science, Suzuka University of Medical Science, Mie, Japan
[7]Graduate School of Regional Innovation Studies, Mie University, Mie, Japan
[8]Faculty of Medicine, Kochi University, Kochi, Japan
[9]Hakubi Center for Advanced Research, Kyoto University, Kyoto, Japan
[10]Faculty of Medicine, Cenderawasih University, Papua, Indonesia
[11]Faculty of Public Health, Cenderawasih University, Papua, Indonesia

Correspondence to: Kiyohito Okumiya,
Research Institute for Humanity and Nature
Motoyama 457-4, Kamigamo, Kita-ku, Kyoto 603-8047, Japan
Tel: +81-75-707-2411, Fax: +81-75-707-2509
E-mail: okumiyak@chikyu.ac.jp
Center for Southeast Asian Studies
Kyoto University
46, Shimoadachi-cho, Yoshida , Sakyo-ku, 606-9501, Japan
Tel: +81-75-753-7320
E-mail: okumiya@cseas.kyoto-u.ac.jp

Abstract:296 words
Text:5299 words
4 tables and 3 figures

For peer review only

ABSTRACT

Objective: Only one prior follow-up study of amyotrophic lateral sclerosis (ALS) and parkinsonism in Papua, Indonesia has been carried out since a survey undertaken from 1962 to 1981 by Gajdusek and colleagues. Therefore, to clarify the clinical epidemiology of ALS and parkinsonism in the south coastal region of Papua, the clinical characteristics and prevalence of the diseases in this region were examined and assessed.

Methods: Cases of ALS and parkinsonism were clinically examined during a 2001–2012 survey in Bade and other villages along the Ia, Edera, Dumut, and Obaa rivers in Papua, Indonesia. Possible, probable, and definite ALS were diagnosed clinically by certified neurologists on the basis of El Escorial Criteria. The criteria for diagnosis of parkinsonism were the presence of at least 2 of the 4 following signs: tremor, rigidity, bradykinesia, and postural impairment with a progressive course.

Results: During the survey, 46 cases of ALS and/or parkinsonism were diagnosed within a population range of 7000 (2001–2002) to 13,900 (2007–2012). The 46 cases consisted of 17 probable-definite cases of ALS, including 3 of cognitive impairment (CI); 13 cases of overlap of possible, probable, or definite ALS and parkinsonism, including 5 cases of CI; and 16 cases of parkinsonism, including 1 case of CI. The crude point prevalence rate of pure ALS was estimated as at least 73 (0–156) to 133 (27–240)/100,000 people and that of the overlap of ALS and parkinsonism was at least 53 (0–126) to 98 (2–193)/100,000 in 2007 or 2010 in some regions.

Conclusions: While the prevalence of ALS has decreased over the past ~30-35 years, it remains higher than the global average. There was a high prevalence of overlap of ALS, parkinsonism, and CI, which has been previously reported in Guam and Kii.

For peer review only - http://bmjopen.bmj.com/site/about/guidelines.xhtml

Strengths and limitations of this study

- This study is unique in the epidemiological survey of neurodegenerative diseases during the period between 2001 and 2012 in Papua, Indonesia, one of the foci with the highest incidence of ALS in the world.
- This study recognized the clinical findings of significant overlap of ALS with parkinsonism and cognitive impairment.
- This study was based only on clinical findings and there are the limitations of not having electromyogram data, DNA analysis, or any autopsy data from this population. Without such data, it is difficult to be certain how these patients fit into sporadic ALS or Parkinson's disease and how they compare with ALS/PDC on Guam and in the Kii peninsula.

For peer review only - http://bmjopen.bmj.com/site/about/guidelines.xhtml

INTRODUCTION

Between 1962 and 1980, Gajdusek et al. reported 97 cases of amyotrophic lateral sclerosis (ALS), 18 cases of parkinsonism, and 18 cases of poliomyeloradiculitis (PMR) in the south coastal area of Papua (former Irian Jaya, Indonesia), which had a population of 7,000. The Ia River is in the epicenter of the high-incidence focus of ALS and parkinsonism in Papua.[1,2] Along with Guam and Kii in Japan, Papua (Indonesia) has been considered one of the foci with the highest incidence of ALS in the world.[1-7] The high incidence of ALS and parkinsonism-dementia complex (PDC) in Guam and Kii reportedly no longer exists.[8,9] In 1987, Spencer et al. reported only 2 cases of ALS and three cases of parkinsonism in the villages along the Ia River. In 1990, no cases of ALS and three cases of parkinsonism were reported along the same river; thus, decline or disappearance of ALS was suspected to have also occurred in Papua.[10] No follow-up surveys on ALS and parkinsonism have been conducted in Papua since 1991.

To clarify the clinical epidemiological characteristics of ALS and parkinsonism in the south coastal region of Papua, we conducted a survey of neurodegenerative diseases documented between 2001 and 2012.

For peer review only

METHODS

The survey sites for between 2001 and 2002 (total population, 7000) were the villages of Kepi (population, 800), Emete (800), Ogoto (400), Senggo (2000), and Bade (3000).[11,12] For between 2007 and 2012, the survey sites were the villages along 4 rivers (total population, 13,900), namely Bade along the Digul River (population, 3800

For peer review only - http://bmjopen.bmj.com/site/about/guidelines.xhtml

people), Mur along the Mappi River (1500), the 8 villages along the Ia River (4500), and the 12 villages along the Edera and Dumut rivers (4100 people). The 8 villages along the Ia River were Gimikya (800), Geturki (500), Homlikya (700), Kobeta (600), Asset (600), Osso (300), Bosma (500), and Ogorito (500). The 12 villages along the Edera and Dumut rivers were Benggo (200), Kokoya (500), Sahapikya (400), Harapan (400), Yibin (300), Mopio (300), Pies (300), Memes (300), Habeske (300), Muya (300), Getio (200), and Sien (600; Figure 1). At least 2 of 6 Japanese-certified neurologists (KO, MF, YH, SK, YK, and KM) visited each village with a local neurologist (IM).

The village leaders and medical staff members of the primary healthcare centre in each village agreed to take part in the survey, which was conducted with the permission of the Department of Health of the Papua Provincial Government in cooperation with Cenderawasih University. The purpose of the neurological examinations was explained to all of the villagers. All the subjects volunteered to participate after the announcement of availability of neurological check-ups, which were performed in the community health centres or homes in each field site. While this was not a complete inventory survey, we asked each medical doctor or health staff, and village leader to call all patients with neurological signs or symptoms, including muscle weakness, gait disturbance, tremor, bradykinesia, or cognitive impairment (CI), in each village. The patients who were called also volunteered to participate. Indonesian collaborators and the co-authors (an English teacher in Bade Senior High School and Indonesian neurologist IM, and staff from Cenderawasih University) who spoke both English and Indonesian; local people who spoke both Indonesian and the local language in each

For peer review only - http://bmjopen.bmj.com/site/about/guidelines.xhtml

village; and co-author EG, who spoke English, Indonesian, and Japanese, cooperatively served as translators. Written informed consent was obtained from all the study participants with the support of their family members. All the participants agreed to undergo neurological examinations, and none of the patients declined annual re-examination. The survey was approved by the ethics committees of the Research Institute for Humanity and Nature in Japan and Cenderawasih University in Papua, Indonesia.

Among the many types of neurological and non-neurological cases encountered, diagnoses of ALS, parkinsonism, and CI were based on clinical examinations and discussions among the participating neurologists. ALS was classified as 'definite', 'probable', 'possible', and 'suspected' on the basis of combinations of upper motor neuron (UMN) and lower motor neuron (LMN) signs according to the El Escorial Criteria of the World Federation of Neurology.[13] Probable and definite ALS are defined as pure ALS in this report. A diagnosis of parkinsonism required the presence of at least 2 of the following 4 signs: tremor, rigidity, bradykinesia, and postural impairment with a progressive course without evidence/history of vascular accidents or history of taking drugs known to induce parkinsonism. The degree of disability of patients with parkinsonism was classified according to the scale of Hoehn and Yahr.[14] Overlap of possible, probable, or definite ALS with parkinsonism was defined as ALS-parkinsonism. CI was diagnosed when loss of memory or impairments of language usage, praxis, or executive functions were identified during interview and clinical examination by neurologists. As cognitive functional test such as the mini–mental state examination was not performed for all of the participants, patients with complications

For peer review only - http://bmjopen.bmj.com/site/about/guidelines.xhtml

of functional disabilities attributable to CI in activities of daily living or in their livelihood were screened during the interview of both the patients and their family members. PMR was evaluated using the definition of Gajdusek et al. that PMR is a subacute paralytic condition reminiscent of the Landry-Guillain-Barre syndrome, which combines radicular, or perhaps neuritic elements with more acute onset and sometimes asymmetric paralysis.[1,2] Neurological cases with overlapping cerebellar signs (2 cases) were excluded from this study.

In Bade and the villages along the Ia, Edera, and Dumut rivers, not only were new cases were identified but also previously diagnosed cases were followed up and assessed neurologically and documented in 2007, 2008, 2010, 2011, and 2012. Death years were confirmed, and the durations between subjective onset of illness and the last survey or death were recorded as durations of subjective illness.

The size of the populations of Bade and the villages along the Ia, Edera, and Dumut rivers were determined from a report by the public office of Edera district in 2006.[15] We were informed by the village leaders of the populations of other villages in the survey for the period 2001–2002.

RESULTS

Clinical types of ALS and/or parkinsonism

We identified 46 cases with signs of ALS and/or parkinsonism, including 17 cases with pure ALS (Table 1), 13 with ALS-parkinsonism (Table 2), and 16 with parkinsonism (Table 3). We found no case of pure or dominant dementia and PMR consistent with the

For peer review only - http://bmjopen.bmj.com/site/about/guidelines.xhtml

definition by Gajdusek et al; similarly, no cases of PMR were found by Spencer et al. in 1987 or 1990. [10] Relevant information about the cases, including neurological signs, age at last survey, age and year of onset, disease duration, certified death year, and ethnicity, is shown in Tables 1–3. The male-to-female sex distribution was 10:7 (male, 59%) for ALS, 11:2 (85%) for ALS-parkinsonism, and 8:8 (50%) for parkinsonism. The mean age at last survey was 51.3 years for ALS, 52.9 years for ALS-parkinsonism, and 51.6 years for parkinsonism. The mean age at onset was 46.2 years (range, 11–68 years) for pure ALS, 49.5 years (11–70 years) for ALS-parkinsonism, and 45.8 years (27–64 years) for parkinsonism. The mean duration of subjective illness was 7.0 years (2–20 years) for pure ALS, 6.3 years (2–17 years) for ALS-parkinsonism, and 6.3 years (2–14 years) for parkinsonism. We observed overlapping CI in three (18%) of the 17 cases of pure ALS, 5 (38%) of the 13 cases of ALS-parkinsonism, and 1 (6%) of the 16 cases of parkinsonism.

During the follow-up surveys of the cases in Bade, Ia, and Edera rivers, we observed the following changes in diagnosis: 1 case, from possible ALS to probable ALS (case ALS10); 2 cases, from parkinsonism to ALS-parkinsonism (cases ALS-P 2 and ALS-P 3); and 1 case, from probable ALS to definite ALS (case ALS16) complicated with CI. We also identified an instance of a shared family history of parkinsonism (P 6) and ALS-parkinsonism (ALS-P 1). There were family histories of neurological disorders in 7 of 17 cases (41%) of pure ALS, 2 (15%) of 13 cases of ALS-parkinsonism, and 6 (38%) of 16 cases of parkinsonism (Tables 1–3).

Cases of ALS and parkinsonism in Bade and the villages along the Ia, Edera, and

For peer review only - http://bmjopen.bmj.com/site/about/guidelines.xhtml

Dumut rivers between 2007 and 2012

Between 2007 and 2012, we identified 4 cases of pure ALS, 2 cases of ALS-parkinsonism, and 1 case of parkinsonism in Bade (population, 3800 people). In addition, we found 7 cases of pure ALS, 4 cases of ALS-parkinsonism, and three cases of parkinsonism in the villages (population, 4500 people) along the Ia river. Between 2008 and 2012, we identified three cases of pure ALS, 5 cases of ALS-parkinsonism, and 6 cases of parkinsonism in the villages (population, 4100 people) along the Edera and Dumut rivers (Table 4).

In the 2007 survey, there were 4 active cases of pure ALS (crude point prevalence rate, 105 [2–208]/100,000 people), 2 cases of ALS-parkinsonism (53 [0–126]/100,000 people), and 1 case of parkinsonism (26 [0–78]/100,000 people) in Bade. In 2010, there were 6 active cases of pure ALS (133 [27–240]/100,000 people), 4 cases of ALS-parkinsonism (89 [2–176]/100,000 people), and three cases of parkinsonism (67 [0–142]/100,000 people) in the villages along the Ia River. In 2010, there were three active cases of pure ALS (73 [0–156]/100,000 people), 4 cases of ALS-parkinsonism (98 [2–193]/100,000 people), and 5 cases of parkinsonism (122 [15-229]/100,000 people) in the villages along the Edera and Dumut rivers (Table 4).

In the survey during the period 2007–2012, the number of each clinical type (ALS, ALS-parkinsonism, and parkinsonism) was shown respectively for Bade (4, 4, and 1); the villages along the Ia River, namely Ogorito (1,2, and 0), Bosma (1, 1, and 0), Homlikya (1, 1, and 1), Gimikya (1, 1, and 0), Geturki (1, 0, and 0), Asset (1, 0, and 1), and Osso (1, 0, and 1); and the villages along the Edera and Dumut rivers, namely Yibin

For peer review only - http://bmjopen.bmj.com/site/about/guidelines.xhtml

For peer review only

(1, 1, and 0), Sien (0, 1, and 2), Pies (1, 0, and 1), Benggo (0, 1, and 0), Harapan (0, 0, and 1), Mopio (0, 0, and 1), Memes (0, 0, and 1), Muya (1, 0, and 0), and Getio (0, 1, and 0).

Case reports

Case ALS 12

A 64-year-old (in 2012) man of the Auyu tribe, a resident of Bosma along the Ia River, had a history of progressive gait disturbance and motor weakness for 9 years in 2010; these symptoms were found to have progressed based on the findings from the follow-up check-ups between 2011 and 2012. Neurological examination revealed UMN and LMN signs, including a positive Babinski sign, hyperreflexia with ankle clonus, muscle atrophy, and weakness in the tongue and upper and lower limbs bilaterally, and fasciculation in the upper limbs and tongue. He had no CI or sensory disturbance. We diagnosed him as having definite ALS (Figure 2-A–D). The subject had ALS, and his brother reported that their father showed tremor and gait disturbance, which were interpreted to be consistent with parkinsonism.

Case ALS-CI 17

A 70-year-old (in 2012) man of the Auyu tribe, a resident of Ogorito along the Ia River, had a history of progressive memory loss for 4 years and slow gait for 2 years. Neurological examination revealed UMN and LMN signs, including a positive Babinski sign, hyperreflexia in the jaw jerk, bilateral upper and lower limbs, and muscle atrophy, weakness, and fasciculation in the distal upper and lower limbs bilaterally. He had

For peer review only - http://bmjopen.bmj.com/site/about/guidelines.xhtml

For peer review only

fasciculation in the tongue and respiratory muscle weakness with a low oxygen saturation level (SpO_2, 86%). He also had memory disturbances accompanied by positive snout and palmomental reflexes and difficulty with mental activities of daily living. He had no sensory disturbance. We diagnosed him as having definite ALS with CI (Figure 3A).

Case ALS-P 1

A 65-year-old (in 2008) woman of the Auyu tribe, a resident of Pies along the Edera River, had a history of progressive motor weakness and gait disturbance for 5 years. She could not walk without support. Neurological examination revealed a resting tremor, cogwheel rigidity in the upper and lower limbs, bradykinesia throughout, postural impairment, UMN and LMN signs with a positive Babinski sign, hyperreflexia in the upper and lower limbs bilaterally, and thenar and hypothenar muscle atrophies. She had no CI or sensory disturbance. We diagnosed her as having ALS-parkinsonism and, more specifically, with severe parkinsonism (Hoen and Yahr IV) simultaneously complicated by motor neuron signs indicative of possible ALS. She died in 2009. Her younger sister was case P 6. The subject had ALS-parkinsonism, and her sister reported that their cousin showed tremor and gait disturbance, which were interpreted to be consistent with parkinsonism (Figure 3B).

Case ALS-P 2

A 76-year-old (in 2011) man of the Auyu tribe, a resident of Ogorito, had a history of tremor in both hands for 5 years in 2010. Neurological examination revealed cogwheel rigidity in his upper and lower limbs, bradykinesia, and UMN signs with hyperreflexia

For peer review only - http://bmjopen.bmj.com/site/about/guidelines.xhtml

For peer review only

in bilateral upper and lower limbs. We diagnosed him as having parkinsonism (Hoen and Yahr II) in 2010. He was examined again in 2011, by which time his parkinsonism was complicated by not only postural impairment but also muscle weakness and atrophy, and spasticity of the upper and lower limbs with Babinski signs. He had no CI or sensory disturbance. We diagnosed him as having parkinsonism (Hoen and Yahr III) with possible ALS; parkinsonism was accompanied by ALS later. He died in 2011 of unknown causes.

We also diagnosed another case of ALS-P3 parkinsonism (Hoen and Yahr III) with possible ALS; parkinsonism was accompanied by ALS later.

Case ALS-P-CI 6

A 50-year-old (in 2012) man of the Auyu tribe, a resident of Benggo along the Edera River, had a history of dysarthria for 2 years. Neurological examination revealed UMN and LMN signs with a positive Babinski sign, muscle atrophy, and weakness in the tongue, proximal upper limb, shoulder, and tibialis anterior muscles. He had cogwheel rigidity in the wrist and elbow, bradykinesia throughout, a positive Myerson sign, and impairment of coordination and postural reflex. He had memory loss, bradyphrenia, slow speech, and positive snout and palmomental reflexes with difficulty performing activities of daily living. We diagnosed him as having a definite ALS and parkinsonism (Hoen and Yahr III) with CI (Figure 3C).

Case P 7

A 52-year-old (in 2010) woman of the Auyu tribe who lived in Mopio along the Edera

For peer review only - http://bmjopen.bmj.com/site/about/guidelines.xhtml

River had a history of progressive gait disturbance with parkinsonian signs, including resting tremor, cogwheel rigidity, anterior bending, and bradykinesia for 3 years in 2008. In 2008, we diagnosed her as having parkinsonism (Hoen and Yahr II). By the time of the follow-up survey in 2010, she could not stand by herself and had postural impairment with parkinsonian hands bilaterally. She had no UMN or LMN signs, no CI, and no sensory disturbance. We diagnosed her as having parkinsonism (Hoen and Yahr IV; Figure 3D).

DISCUSSION

Internal migration and migration from outside the province are the most significant causes of demographic, social, and cultural change in Papua. The populations in the survey sites were composed of former hunter-gatherers who lived in the forest and who were brought out of the forest and assembled in villages by Dutch colonialists. The villages along the Ia River were founded in 1937–1951.[10] The emigration program called transmigration (*transmigrasi*) started in Irian Jaya in 1964. Local migrants and transmigrants from outside Irian Jaya increased, resulting in various changes, including the use of food and medicine.[16,17] Considering that Gadjusek et al. found out the high prevalence of ALS at the first survey in 1962, the neurodegenerative disease existed before the introduction of manufactured items.[1]

In the present survey, we performed full neurological examinations in 46 subjects with ALS and/or parkinsonism between 2001 and 2012. Among the subjects, 17 were probable-definite cases of pure ALS, 13 were overlapping ALS (possible, probable, or definite) and parkinsonism, and 16 were parkinsonism. Nine cases of ALS and/or

For peer review only - http://bmjopen.bmj.com/site/about/guidelines.xhtml

parkinsonism had a complication of CI. During the study period, the diagnosis in some patients changed from pure parkinsonism to ALS-parkinsonism, and their disease progressed in severity. Some subjects had both ALS and parkinsonism on their first examination. It is thus evident that overlapping ALS and parkinsonism with or without CI still exists in the specified region of Papua.

Gajdusek et al. reported diagnosing 97 cases (57 cases clinically confirmed and 40 cases not clinically confirmed) of ALS during the 6 surveys they conducted from 1974 to 1981.[1] However, they reported neurological findings of only 32 of these cases, in only 16 of which the reported findings of UMN, LMN, and parkinsonian signs justify a diagnosis of probable or definite ALS. The 16 cases comprise 12 cases of pure ALS and 4 cases of overlapping ALS-parkinsonism. Among the 32 ALS patients, three had a positive family history (9%); that is, 2 had sisters with ALS, and 1 who had ALS-parkinsonism had a family history of ALS. None of the ALS patients had a family history of parkinsonism. The authors also reported their neurological findings in 13 subjects in whom parkinsonism was diagnosed. They reported their findings from full neurological examinations of 10 of these parkinsonism cases; according to their reported neurological signs, these comprised 8 patients of parkinsonism, including 5 with dementia and 2 with overlapping ALS-parkinsonism with dementia.[1] Among the 13 parkinsonism patients, three had a positive family history (23%). In summary, we believe that in the 26 cases reported by Gajdusek et al., the full neurological examination findings comprised 12 pure ALS cases (probable or definite), 6 overlapping ALS-parkinsonism, and 8 parkinsonism with/without dementia.[1] Spencer

For peer review only - http://bmjopen.bmj.com/site/about/guidelines.xhtml

et al reported 2 cases of ALS and three cases of parkinsonism, including 1 with dementia, in the survey in 1987. He also reported 3 subjects with parkinsonism overlapping UMN signs, including 1 with dementia in 1990. We have here reported 17 cases of pure ALS (probable or definite), 13 cases of overlapping ALS-parkinsonism, and 16 cases of parkinsonism with or without CI in the 46 cases we neurologically examined fully. Thus, we identified many cases of overlapping ALS and parkinsonism (n = 13, 28%) like that observed in previous reports (n = 6, 23% and n = 3, 38%).[1, 10] We also identified many cases with CI (n = 9, 20%) like that observed in previous reports (n = 7, 27% and n = 2, 25%).[1, 10] Functional disabilities attributable to CI were recognized in activities of daily living or in their livelihood in all the cases with CI in this report.

Gajdusek et al. reported 13 cases of ALS and three cases of parkinsonism in 1975 in the villages along the Ia River (population, 2000).[1] The 13 ALS cases consisted of 10 clinically confirmed ones and 3 others that were not clinically confirmed. We compared the prevalence of probable-definite ALS and parkinsonism according to the report of Gajdusek et al. with our more recent findings. A crude point prevalence of ALS consistent with clinically probable to definite ALS (El Escorial criteria) may be estimated between 500 (95% confidence interval, 191–809/100,000 people) and 650 (298–1002) and that of parkinsonism was 150 (0–320/100,000 people) in 1975 along the Ia River. In our 2010 survey (population, 4500), we identified 6 probable-definite cases of ALS in this region (ALS 8, 10–13, and 17; 133 [27–-240]/100,000 people) and 7 of parkinsonism (possible ALS-P 2, 3, 9, and 13, and P 10, 15, and 16; 156 [40–271]/100,000 people).

For peer review only - http://bmjopen.bmj.com/site/about/guidelines.xhtml

Gajdusek et al. reported 8 cases of ALS and 6 cases of parkinsonism in 1975 in the villages along the Edera and Dumut rivers (population, 1950). The 8 cases of ALS consisted of 7 clinically confirmed cases and 1 unconfirmed case. The crude point prevalence of ALS consistent with clinically probable to definite ALS (El Escorial criteria) may be estimated between 359 (94–624)/100,000 people and 410 (127–694)/100,000 people, and that of parkinsonism was 308 (62–554)/100,000 people in 1975 along the Edera and Dumut rivers. In our survey of this region in 2010 (population, 4100), we identified 5 probable-definite cases of ALS (ALS 9, 14, and 16, and ALS-P 5, 6; 122 [15–229]/100,000 people) and 7 cases of parkinsonism (possible ALS-P 10 and 11, and P 7, 8, and 11–13; 171 [44–297]/100,000 people; Table 4). As this was not a complete inventory survey, the actual prevalence may be a little higher than our estimation.

The population of Bade increased by a factor of 4 during 31 years (from 1000 in 1975 to 3800 in 2006), and those of villages along the Ia River (from 2000 to 4500) and the Edera and Dumut rivers (from 1950 to 4100) have increased 2-fold.[1,15] The subjects born outside Papua were only 2 (Maluku) among the 46 subjects with ALS and/or parkinsonism with/without CI in this survey, ie 44 were of Papuan genetic stock and 2 (from Maluku) were non-Papuan. The prevalence of the diseases among the local Papuan residents in this survey may be underestimated compared with the former report after taking into account the increase in the numbers of local migrants inside and transmigrants from outside Papua.[16,17,18]

For peer review only - http://bmjopen.bmj.com/site/about/guidelines.xhtml

As the median age and life expectancy in the field sites in Papua are estimated to be lower than the global median and fewer people may live beyond the age of 40 or 50 years,[18,19] the prevalence of the diseases in this survey may be underestimated compared with the global mean. As the median age and life expectancy in 1975 may be lower than those in 2010,[20] the prevalence of the diseases in the report by Gajdusek et al. might be underestimated compared with that in our survey.

The prevalence of ALS in this report was found to be three times lower than found by Gajdusek et al. Even after taking into account the change of population and age structure in the field sites, our findings show that the prevalence of ALS appears to have decreased over the past ~30-35 years since the report of Gajdusek et al.[10] This is consistent with the previous report by Spencer et al. However, it is still much higher than the global mean (incidence, approximately 2/100,000; prevalence, approximately 6/100,000) [21-26]; in particular, the prevalence of overlapping ALS and parkinsonism is especially high in Papua.

In the report by Gajdusek et al., who provided village-by-village data showing the number of cases of ALS and parkinsonism, there were many cases in the remote villages such as Bosma, Pies, Homlikya, Yibin, Sien, and Ogorite, which were less visited and least developed.[1, 2] In this survey, many cases (three ALS/ALS-parkinsonism) were found in the remotest village of Ogorito along the Ia River. Two cases of ALS/ALS-parkinsonism were also found in Bosma, Homlikya, and Yibin; 1 ALS and 2 cases of parkinsonism in Sien; 1 ALS and 1 parkinsonism in Pies, which consisted also of remote villages. While there were 8 cases of

For peer review only - http://bmjopen.bmj.com/site/about/guidelines.xhtml

ALS/ALS-parkinsonism in Bade and 2 in Gimikya, nearest to the port of the Digul River, none was reported by Gajdusek et al. in those sites. This increase may be brought about by local migrants from the affected villages.

Overlap of ALS-parkinsonism and dementia has also been reported in patients with ALS/PDC in Guam and Kii, Japan.[4, 27-33] Hirano et al. provided clinicopathological evidence of 47 cases of PDC in Guam in 1961, comprising the following 3 groups: (1) 30 cases of PDC; (2) 8 cases of parkinsonism with dementia with clinical evidence of UMN impairment; and (3) 9 cases of parkinsonism with dementia with clinical evidence of UMN and LMN impairment. At least 15 of these cases (31.9%) had a family history of ALS, parkinsonism, or both.[4] Kuzuhara and Kokubo et al. surveyed neurological diseases in Kii, Japan, in the 1990s and found not only a continuously high incidence of ALS but also neuropathologically verified cases of PDC. ALS and PDC frequently affected the same individuals simultaneously and the members of the same family. These authors reported 37 cases of ALS/PDC between 1996 and 2006, a high proportion of whom (78%) had a positive family history, as well as clinical examples of pure ALS, PDC, and overlapping ALS and PDC. They also reported neuropathological changes common to cases of ALS and PDC in 12 cases of ALS/PDC, resembling those found in patients with ALS/PDC in Guam [28-33] In our more recent study in Papua, such as the previous studies in Kii, Japan, and Guam, we identified many cases of pure ALS and ALS overlapping with parkinsonism with/without CI. The prevalence of CI was lower in our study sites than in Guam and Kii.[4, 27-33] CI might have been underestimated, and mild CIs or some mild dementia might have been overlooked by our neurological examinations in the field.

For peer review only - http://bmjopen.bmj.com/site/about/guidelines.xhtml

Our study was based only on clinical findings, and there are the limitations of not having electromyogram data, DNA analysis data, or any autopsy data from this population. Without such data, it is difficult to be certain how these patients fit into sporadic ALS or Parkinson disease and how they compare with ALS/PDC on Guam and in the Kii peninsula. However, we did identify many patients with overlapping ALS-parkinsonism in this study. The overlap of ALS and/or parkinsonism with CI suggests that it may be the same disease entity as the ALS/PDC diagnosed in Guam and Kii.

The proportion of males in the populations of Bade, the villages along the Ia River, and the villages along the Edera and Dumut rivers were 53%, 51%, and 50%. The proportion of males was high especially among the subjects with ALS-parkinsonism (85%) and ALS (59%) compared with among those with pure parkinsonism (50%) in our report. The proportion of males was higher among the subjects with ALS (63%) and parkinsonism (76%) in the report by Gadjusek et al., in which there were 3 male cases (75%) among the 4 ALS-parkinsonism cases. The high risks for ALS and PDC among the males were recognized in Guam and Kii.[1,7,27]

Indigenous people of Papua still retain aspects of their traditional lifestyles, such as hunting, gathering, fishing, and eating *sago* (trunk of *Metroxylon* sp.). They also drink water from shallow wells. However, with the spread of a market economy, recent increases in the numbers of rubber plantations and progress in transportation have been causing lifestyle changes in the villages in the south coastal area of Papua. As the diseases are declining as in Guam and Kii, the disease aetiology in Papua may be dominated by environmental factors. Genetic, culture-related and/or family-specific

For peer review only - http://bmjopen.bmj.com/site/about/guidelines.xhtml

factors may also play a role as evidenced by the family history of disease in 40% of the subjects in this survey in Papua and 80% in Kii.

Various possible causal genetic and environmental factors for these conditions have been reported. The environmental factors of low concentrations of calcium and magnesium in drinking water were hypothesized. [1,34] There is a recent report showing an association between the increase in ALS incidence and the change in drinking water source in the Kii Peninsula.[35] Spencer et al. pointed out that this explanation was inconsistent with a declining prevalence of ALS in a sessile population dependent on an unchanging supply of river water, which this report may support.[10]

Spencer et al. reported the discovery that cycad seed was used as a poultice in Papua, as in Guam. [36] Cycad-derived products were shown as the risk factor of dementia, mild CI, and PDC in Guam with the epidemiological evidence.[37] Although cycad had been suspected as the causal factor of Kii ALS,[38] people in Japan, including the Kii Peninsula, had never consumed cycad as daily food, although a small amount of cycad flour might have been taken by a few people as an ingredient of herb medicine.[39] We did not examine the association of prior skin wounds and exposure to cycad poultice with the diseases. Skin change and pigmentary retinopathy were not examined in this survey.[40, 41] Further studies are needed.

Genetic abnormalities that had been previously established to be associated with familial or sporadic ALS, dementia, familial Parkinson disease, and parkinsonism were looked for in the cases in Kii, but none of them were identified.[28,42] Hermousura et

For peer review only - http://bmjopen.bmj.com/site/about/guidelines.xhtml

al. have discovered a nuclear mutation in the calcium/magnesium membrane ion channel transient receptor potential melastatin 7 (*TRPM7*) gene in a subset of patients with ALS/PDC in Guam.[43] They reported that a TRPM7 variant that is associated with altered sensitivity to magnesium may have contributed to the pathogenesis of ALS and PDC in 2 Guamanians. *TRPM7* was not found to be associated with ALS/PDC in the Kii peninsula of Japan.[44] Ishiura et al. have recently discovered *C9ORF72* repeat expansion in some cases of ALS in the Kii peninsula of Japan; this repeat expansion partly accounts for the high prevalence of ALS in this region.[45] However, the responsible gene(s) remain unproved in almost all cases of ALS/PDC in both Guam and Kii.

Various environmental factors and multiple genetic factors are suggested to be underlying causes of these diseases, but these links are so far unproven.[33,46]

In Guam, although the incidence of pure ALS has declined markedly, the prevalence of PDC is still high.[47] The prevalence of dementia among elderly Chamorros (earliest known inhabitants of the Mariana Islands, which include Guam) is also reportedly relatively high.[48] In addition, in Kii the incidence of pure ALS has declined markedly, and the prevalence of PDC and ALS/PDC or ALS with dementia recently increased.[30,33] These changing patterns of ALS/PDC incidence in Guam and Kii might be caused by changes in environmental and socioeconomic factors as well as aging.[48,49]

The mean age of onset in our cases was 46 years for ALS, 53 years for ALS-parkinsonism, and 46 years for parkinsonism. Thus, they were older than the

For peer review only - http://bmjopen.bmj.com/site/about/guidelines.xhtml

previously reported cases of ALS (33 years) and parkinsonism (43 years).[1] In our cases, the mean duration of subjective illness was 7.0 years for ALS, 6.3 years for ALS-parkinsonism, and 6.3 years for parkinsonism. The duration of ALS in our data was longer than not only the world mean of 3 years[21-26] but also the previously reported 3.5 years for ALS in Papua.[1] The mean age of onset may recently have increased, and the mean disease duration became longer in subjects with ALS. The prevalence rate of cases with bulbar sign was 37% in 30 of pure ALS or ALS-parkinsonian in our report, and it was as the same as in the report of Gadjdusek (40%) and classical ALS (39%).[2] The change in onset and course of ALS in recent years might be attributable to the aging population and changing environmental and socioeconomic factors.

On-going follow-up surveys should be performed to evaluate changes in the clinical types of ALS and/or parkinsonism with or without CI and accompanying changes in environmental and socioeconomic factors, as have occurred in Guam and Kii.

In conclusion, The prevalence of ALS in the Papuan disease focus has decreased over the past ~30-35 years but, in 2010, it remained higher than the global average. During this period, there has been an influx of migrants from inside and transmigrants from outside Papua that has increased the size and changed the composition of the population in the villages studied. There may be the possible impact of the expanded population on prevalence estimates. There are still many cases of pure ALS and overlapping ALS-parkinsonism and CI in Papua, as has previously been reported in Guam and Kii. The disease continues to impact native Papuans but may also affect a small number of

For peer review only - http://bmjopen.bmj.com/site/about/guidelines.xhtml

non-Papuan immigrants (2 of 46 or 3-4%) who adopt the local lifestyle. Environmental factors may play important etiologic role. Further on-going follow-up surveys focusing on the clinical epidemiology and aetiology of these neurodegenerative diseases in association with ecological and environmental factors in Papua are needed. Future studies, including neuropathological examinations, should be performed when the local villagers in this region of Papua, Indonesia, are willing to consent to autopsies.

ACKNOWLEDGMENTS

The authors wish to thank all the participants in the villages who volunteered for the survey, the village leaders, and Mappi Regency officials. We are deeply indebted to doctors Imelda Mandagi, Reginald Nangoy, and Christha Z. Tamburian and their staff at Puskesmas Bade, and to Arifin Wasaraka, Lazarus Revasy, Yosefina Griapon, Arius Togodly, Kerry Yarangga, and Dolfinus Y. Bouway at Cenderawasih University for their invaluable contributions to the study. Andre Liem and Herry Wondiwoy at Papua Tour Guides Community and S.E. Amos at Bade High School provided logistic and language assistance. On the Japanese side, we acknowledge the technical support and advice of Katsuyuki Eguchi (Nagasaki University), Yasushi Osaki (Kochi University), Hidekazu Tomimoto (Mie University), Taro Yamauchi (The University of Tokyo), Hideyuki Onishi (Doshisha Women's College), Tetsuya Inamura (Aichi Prefectural University), Wenling Chen, Yumi Kimura, Yasuko Ishimoto, and Hissei Imai (Kyoto University), and Yasuyuki Kosaka, Yukiko Kita, and Chizu Wada (Research Institute for Humanity and Nature).

For peer review only - http://bmjopen.bmj.com/site/about/guidelines.xhtml

FUNDING

K. Okumiya receives research support from Grant-in-Aid for Scientific Research (A) from the Japan Society for the Promotion of Science (JSPS). K. Matsubayashi receives research support from Grant-in-Aid for Scientific Research (A) from the JSPS and is an editor of the journal *Geriatrics & Gerontology International*. T. Wada receives research support from Grant-in-Aid for Scientific Research (C) from the JSPS. Y. Kokubo and S. Kuzuhara receive support from Grants-in-Aid from the Research Committee of CNS Degenerative Diseases and the Research Committee on the Muro disease (Kii amyotrophic lateral sclerosis/Parkinsonism-dementia), the Ministry of Health, Labour and Welfare of Japan.

AUTHOR CONTRIBUTIONS

Okumiya had full access to all the data in the study and takes responsibility for the integrity of the data and the accuracy of the data analysis.

Study concept and design: Okumiya, Matsubayashi.

Acquisition of data: Okumiya, Wada, Fujisawa, Ishine, Garcia del Saz Hirata, Kuzuhara, Kokubo, Seguchi, Sakamoto, Manuaba, Matsubayashi.

Analysis and interpretation of data: Okumiya,Wada, Sakamoto, Fujisawa, Matusbayashi.

Drafting of the manuscript: Okumiya,Wada, Sakamoto, Fujisawa, Matusbayashi.

Critical revision of the manuscript for important intellectual content: All authors.

Statistical analysis: Okumiya.

Obtained funding: Okumiya, Wada, Kuzuhara, Kokubo, Matsubayashi

Administrative, technical, or material support: Okumiya, Watofa, Rantetampang,

For peer review only - http://bmjopen.bmj.com/site/about/guidelines.xhtml

Matsubayashi.

Study supervision: Okumiya, Matsubayashi.

COMPETING INTERESTS : None declared

DATA SHARING STATEMENT: Okumiya had full access to all the data in the study and takes responsibility for the integrity of the data and the accuracy of the data analysis.

For peer review only

For peer review only - http://bmjopen.bmj.com/site/about/guidelines.xhtml

REFERENCES

1. Gajdusek DC, Salazar AM. Amyotrophic lateral sclerosis and parkinsonian syndromes in high incidence among the Auyu and Jakai people of West New Guinea. Neurology 1982;**32**:107–26.
2. Gajdusek DC. Foci of motor neuron disease in high incidence in isolated populations of East Asia and the Western Pacific. In: Lewis P Rowland, ed. Human Motor Neuron Diseases. New York: Raven Press 1982:363–93.
3. Kurland LT, Mulder DW. Epidemiologic investigations of amyotrophic lateral sclerosis. 1. Preliminary report on geographic distribution and special reference to the Mariana Islands, including clinical and pathologic observations. Neurology 1954;**4**:438–48.
4. Hirano A, Kurland LT, Krooth RS, et al. Parkinsonism-dementia complex, an endemic disease on the island of Guam. I. Clinical features. Brain 1961;**84**:642–61.
5. Hirano A, Malamud N, Kurland LT. Parkinsonism-dementia complex, an endemic disease on the island of Guam. II. Pathological features. Brain 1961;**84**:662–79.
6. Kimura K, Yase Y, Higashi Y, et al. Epidemiological and geomedical studies on ALS and allied diseases in Kii peninsula (Japan): preliminary report. Proc Jpn Acad 1961;**37**:417–20.
7. Shiraki H, Yase Y. Amyotrophic lateral sclerosis in Japan. In: Vinken PJ, Bruyn GW, eds. Handbook of clinical neurology Vol. 22, System disorders and atrophies, Part II. Amsterdam: Elsevier Science 1975:353–419.
8. Garruto RM, Yanagihara R, Gajdusek DC. Disappearance of high incidence ALS and PDC on Guam. Neurology 1985;**35**:193–8.
9. Yoshida S. ALS tahatsuchi niokeru kankyoyoin to aluminium no neuron hensei eno

For peer review only - http://bmjopen.bmj.com/site/about/guidelines.xhtml

kanyo nitsuite.[Environmental factors in western Pacific foci of ALS and a possible pathogenetic role of aluminum (Al) in motor neuron degeneration] (in Japanese with English abstract). Rinsho Shinkeigaku [Clin Neurol] 1991;**31**:1310–2.

10. Spencer P, Palmer VS, Ludolph AC. On the decline and etiology of high-incidence motor system disease in West Papua (Southwest New Guinea). Mov Disord 2005;**20(**Suppl 12):S119–126.
11. Wada T, Matsubayashi K, Okumiya K, et al. Health status and subjective economic satisfaction in West Papua. Lancet 2002;**360**:951.
12. Okumiya K, Fujisawa M, Ishine M, et al. Nishi New Guinea chiiki (Indonesia/ Papua shu) no shinkeihensei shikkan no jittai. 2001-2002 nen, 2006-2007 nen no fieldwork yori. [Fieldwork survey of neurodegenerative diseases in West New-Guinea in 2001-02 and 2006-07] (in Japanese). Rinsho Shinkeigaku [Clin Neurol] 2007;**47**:977–8.
13. Brooks BR. El Escorial World Federation of Neurology criteria for the diagnosis of amyotrophic lateral sclerosis: subcommittee on Motor Neuron Diseases/Amyotrophic Lateral Sclerosis of the World Federation of Neurology Research Group on Neuromuscular Diseases and the El Escorial "Clinical Limits of ALS" Workshop Contributors. J Neurol Sci 1994;**124**:96–107.
14. Hoehn MM, Yahr MD. Parkinsonism: onset, progression and mortality. Neurology. 1967;**17**:427–42.
15. Kontor Statistik Distrik Edera. Laporan Kependudukan, Kabupaten Mappi Distrik Edera 2006 (December).
16. Manning C., Rumiak M. Irian Jaya: Economic change, migrants, and indinenous welfare. In Hill H ed. Unity and diversity: Regional Economic Development in

For peer review only

For peer review only - http://bmjopen.bmj.com/site/about/guidelines.xhtml

Indonesia since 1970. Oxford University Press, Okford.p77-106, 1991.

17. Timmer J. A brief social and political history of Papua, 1962-2005. In Marshall AJ and Beehler BM ed. The Ecology of Indonesian Papua Part Two. Periplus Editions, Hong Kong. p1098-1124, 2007.

18. Badan Pusat Statistik, Sensus Penduduk 2010. http://sp2010.bps.go.id/index.php

19. United Nations , Department of Economic and Social Affairs, Population Division. World Population Prospects The 2012 Revision *Volume II: Demographic Profiles,* http://esa.un.org/unpd/wpp/index.htm

20. Kontor Statistik Kabupaten Merauke. Laporan Kependudukan, Kabupaten Merauke 1997

21. Armon C. Amyotrophic lateral sclerosis. In: Neuroepidemiology from principles to practice. USA: Oxford University Press 2004:162–87.

22. McGuire V, Longstreth WT Jr, Koepsell TD, et al. Incidence of amyotrophic lateral sclerosis in three counties in western Washington state. Neurology 1996 ;**47**:571–3.

23. Sorenson EJ, Stalker AP, Kurland LT, et al. Amyotrophic lateral sclerosis in Olmsted County, Minnesota, 1925 to 1998. Neurology 2002; **23**:280–2.

24. O'Toole O, Traynor BJ, Brennan P, et al. Epidemiology and clinical features of amyotrophic lateral sclerosis in Ireland between 1995 and 2004. J Neurol Neurosurg Psychiatry 2008;**79**:30–2.

25. Chiò A, Mora G, Calvo A, et al. Epidemiology of ALS in Italy: a 10-year prospective population-based study. Neurology 2009;**72**:725–31.

26. Factor-Litvak P, Al-Chalabi A, Ascherio A, et al. Current pathways for epidemiological research in amyotrophic lateral sclerosis. Amyotroph Lateral Scler Frontotemporal Degener 2013;**14**(Suppl 1):33–43.

For peer review only

For peer review only - http://bmjopen.bmj.com/site/about/guidelines.xhtml

27. Rodgers-Johnson P, Garruto RM, Yanigahara R, et al. Amyotrophic lateral sclerosis and parkinsonism-dementia on Guam: a 30-year evaluation of clinical and neurological trends. *Neurology.* 1986;36:7-13.

28. Kuzuhara S, Kokubo Y, Sasaki R, et al. Familial amyotrophic lateral sclerosis and parkinsonism-dementia complex of the Kii peninsula of Japan: clinical and neuropathological study and tau analysis. Ann Neurol 2001;**49**:501–11.

29. Kuzuhara S, Kokubo Y. Atypical parkinsonism of Japan: amyotrophic lateral sclerosis-parkinsonism-dementia complex of the Kii peninsula of Japan (Muro disease): an update. Mov Disord 2005;**20**(Suppl 12):S108–13.

30. Kuzuhara S. Kii ALS/PDC (Murou byou) no rekishi, gainen, ekigaku. [ALS-parkinsonism-dementia complex of the Kii peninsula of Japan (Muro disease). Historical review, epidemiology and concept] (in Japanese with English abstract). Rinsho Shinkeigaku [Clin Neurol] 2007;**47**:962–5.

31. Kokubo Y. Kiihanto no kinishukuseisokusakukokasho/ parkinson chihou hukugo no rinshou, shinkei byourigakuteki kenkyu. [Clinical aspects, imaging and neuropathology of Kii ALS/PDC] (in Japanese with English abstract). Rinsho Shinkeigaku [Clin Neurol] 2007;**47**:966–9.

32. Kuzuhara S. Kii hanto no Murobyo (Kinishukuseisokusakukokasho/ parkinson/ ninchisho hukugo) [Muro disease or ALS–parkinsonism–dementia complex of the Kii peninsula of Japan] (in Japanese with English abstract). Rinsho Shinkeigaku [Clin Neurol] 2007;**47**:695–702.

33. Kuzuhara S, KokuboY. Amyotrophic lateral sclerosis-parkinsonism-dementia complex in the Kii peninsula of Japan (Muro disease): a review on recent research and new concept. In: Michael J. Strong, ed. Amyotrophic lateral sclerosis and the

For peer review only - http://bmjopen.bmj.com/site/about/guidelines.xhtml

frontotemporal dementias. Oxford: Oxford University Press 2012:39–54.

34. Yase Y. The pathogenesis of amyotrophic lateral sclerosis. Lancet 1972;**2**(7772):292–6.

35. Kihara T, Yoshida S, Kondo T et al. An increase in ALS incidence on the Kii Peninsula, 1960-2009: A possible link to change in drinking water source. *Amyotrophic Lateral Sclerosis,* 2012; 13: 347–50.

36. Spencer PS, Nunn PB, Hugon J, et al. Guam amyotrophic lateral sclerosis-parkinsonism-dementia linked to a plant excitant neurotoxin. Science 1987;**237**(4814):517–22.

37. Borenstein AR, Mortimer JA, Schofield E. et al. Cycad exposure and risk of dementia, MCI, and PDC in the Chamorro population of Guam. Neurology. 2007;68:1764-71.

38. Spencer PS, Ohta M, Palmer VS. Cycad use and motor neuron disease in Kii peninsula of Japan. Lancet 1987;**2**(8573):1462–3.

39. Iwami O, Niki Y, Watanabe T, et al. Motor neuron disease on the Kii Peninsula of Japan: Cycad exposure. Neuroepidemiology 1993;12:307-312.

40. Ono S, Waring SC, Kurland L et al. Guamanian neurodegenerative disease: ultrastructural studies of skin J. Neurol. Sci., 146 (1997), pp. 35–40

41. Kokubo Y, Ito K, Kuzuhara S. Ophthalmomyiasis-like pigmentary retinopathy in ALS/PDC in the Kii peninsula of Japan. Neurology. 2003 May 27;60(10):1725-6.

42. Tomiyama H, Kokubo Y, Sasaki R, et al. Mutation analyses in amyotrophic lateral sclerosis/parkinsonism-dementia complex of the Kii peninsula, Japan. Mov Disord 2008;**23**(16):2344–8.

43. Hermosura MC, Nayakanti H, Dorovkov MV, et al. A TRPM7 variant shows altered sensitivity to magnesium that may contribute to the pathogenesis of two Guamanian

For peer review only - http://bmjopen.bmj.com/site/about/guidelines.xhtml

neurodegenerative disorders. Proc Natl Acad Sci 2005;**102**(32):11510–5.

44. Hara K, Kokubo Y, Ishiura H, et al. TRPM7 is not associated with amyotrophic lateral sclerosis-parkinsonism dementia complex in the Kii peninsula of Japan. Am J Med Genet B Neuropsychiatr Genet 2010;**153B**(1):310–3.

45. Ishiura H, Takahashi Y, Mitsui J, et al. *C9ORF72* Repeat expansion in amyotrophic lateral sclerosis in the Kii peninsula of Japan. Arch Neurol 2012;**69**(9):1154–8.

46. Garruto RM, Yanagihara R. Contributions of isolated Pacific populations to understanding neurodegenerative diseases. Folia Neuropathol 2009;**47**(2):149–70.

47. Plato CC, Garruto RM, Galasko D, et al. Amyotrophic lateral sclerosis and parkinsonism-dementia complex of Guam: changing incidence rates during the past 60 years. Am J Epidemiol 2003;**157**:149–57.

48. Galasko D, Salmon D, Gamst A, et al. Prevalence of dementia in Chamorros on Guam: relationship to age, gender, education, and APOE. Neurology 2007;**68**:1772–81.

49. Plato CC, Galasko D, Garruto RM, et al. ALS and PDC of Guam: forty-year follow-up. Neurology 2002;**58**:765–73.

Figure and table legends

Table 1. Relevant characteristics and neurological signs of cases with ALS with/without cognitive impairment in Papua, 2001–2012.

Abbreviations: ALS = amyotrophic lateral sclerosis; BK = bradykinesia; BS = Babinski sign; DTR = deep tendon reflex; Dys = dysphagia/dysarthria; F= female; Fasc =

For peer review only

For peer review only - http://bmjopen.bmj.com/site/about/guidelines.xhtml

fasciculation; LMN = lower motor neuron signs; M = male; MA = muscle atrophy with weakness; NC = not confirmed by examination; P = parkinsonism; PI = postural impairment; R = rigidity; T = tremor; TA = tongue atrophy; U/L: upper limbs/lower limbs; UMN = upper motor neuron signs.

Age: at the time of final survey; Duration: between subjective onset of illness and last survey or death; Alive: alive in 2012; Onset: subjective onset of the illness; Deep tendon reflexes, -: absent, 1: hypoactive, 2: normal, 3: hyperactive, 4: markedly hyperactive with ankle clonus.

Other signs are classified as: -: absent; ±: questionably present; +: present; ++: markedly present.

Family history 1: cousin (possible ALS); 2: grandfather (NC); 3: sister (ALS15); 4: cousin's daughter (P16); 5: cousin (NC); 6: father (NC); 7: sister (ALS 7); 8: sister (P 6) and cousin (NC); 9: father (NC); 10: son: (NC); 11: sister (ALS-P 1) and cousin (NC); 12: mother and father (NC); 13: grandmother (NC); 14: mother (NC); 15: ALS 10 (mother’s cousin).

Table 2. Relevant characteristics and neurological signs in cases with overlap of ALS and parkinsonism with/without cognitive impairment in Papua, 2001–2012.

Table 3. Relevant characteristics and neurological signs in cases with parkinsonism with/without cognitive impairment in Papua, 2001–2012.

Table 4. Cases of ALS and parkinsonism according to field site

For peer review only

*; Active cases in 2007 in Bade

#; Active cases in 2010 in the villages along the Ia, Edera, and Dumut rivers

Figure 1. Map of the field sites

A. Large-scale map of the southern coastal plain in Papua, Indonesia.

B. Smaller scale map of the southern coastal plain in Papua, Indonesia showing rivers.

C. Detailed map showing villages in the southern coastal area in Papua in which all confirmed cases of ALS and/or parkinsonism with/without cognitive impairment were seen from 2001 to 2012.

Figure 2. Photographs of patient with ALS (Case ALS 12**)**

A: The patient needs assistance from family members to stand.

B: Advanced atrophy of the tongue.

C: There is upper limb-girdle and truncal muscle atrophy with a positive Babinski sign.

D: Advanced thenar muscle atrophy.

Figure 3. Photographs of patients with ALS, ALS-parkinsonism, and parkinsonism

A: Patient with ALS (Case ALS-17) showing muscle atrophy in the distal upper and lower limbs.

B: Patient with ALS-parkinsonism (Case ALS-P 1) showing muscle atrophy in distal upper limbs.

C: Patient with ALS-parkinsonism (Case ALS-P 7) showing atrophy in muscles in the proximal upper limb, shoulder, and tibialis anterior.

For peer review only - http://bmjopen.bmj.com/site/about/guidelines.xhtml

D: Patient with parkinsonism (Case P 7) showing typical parkinsonian hands and anterior bending posture.

Parkinsonian hand: Striatal deformities of the hand with abnormal postures that are common in patients with advanced Parkinson's disease.

For peer review only

For peer review only - http://bmjopen.bmj.com/site/about/guidelines.xhtml

Okumiya

Table 1. Relevant characteristics and neurological signs in cases with pure ALS with/without cognitive impairment in Papua, 2001–2012.

Case number	ALS criteria Yahr stage	Survey year	Village	Age	Sex	Onset (age/year)	Duration (years)	Death (year)	Tribe (origin)	UMN signs DTR U/L	UMN signs BS	LMN signs MA U/L	LMN signs Fasc U/L	Bulbar signs TA	Bulbar signs Dys	Parkinsonian sign T U/L	Parkinsonian sign R U/L	Parkinsonian sign BK	Parkinsonian sign PI	Cognitive impairment	Family history
Pure ALS (n=17)																					
ALS 1	Definite	2001	Bade	35	M	NC	NC	NC	Auyu	3/3	+	++/++	+/+	++	-	-	-	-	-	-	-
ALS 2	Definite	2001	Ogoto	43	F	NC	NC	2002	Auyu	3/3	+	++/++	+/+	++	++	-	-	-	-	-	-
ALS 3	Definite	2002	Senggo	50	M	48/'00	2	NC	Citak	3/3	+	++/++	±/±	±	+	-	-	-	-	-	1
ALS 4	Definite	2007	Homilikia	53	M	51/'05	2	2007	Auyu	3/2	+	++/++	+/+	-	-	-	-	-	-	-	-
ALS 5	Definite	2007/'08	Bade	51	M	49/'06	3	2009	Auyu	3/4	+	+/-	-/-	-	+	-	-	-	-	-	-
ALS 6	Definite	2008	Bade	21	M	11/'98	11	2009	Auyu	4/4	+	++/++	-/-	-	-	-	-	-	-	-	2
ALS 7	Definite	2007/'08/'10	Bade	70	F	57/'97	13	2010	Maluku	3/3	+	++/++	+/-	-	+	-	-	-	-	-	3
ALS 8	Probable	2011	Geturki	53	M	48/'06	5	Alive	Auyu	3/3	-	+/+	-/+	-	-	-	-	-	-	-	-
ALS 9	Definite	2011	Muya	41	F	36/'06	6	2012	Auyu	3/3	+	+/+	-/-	-	-	-	-	-	-	-	-
ALS 10	Probable	2007/'10/'11/'12	Osso	65	F	59/'06	6	Alive	Auyu	3/4	+	+/-	-/-	+	-	-	-	-	-	-	4
ALS 11	Probable	2007/'08/'10/'12	Gimikya	32	M	12/'92	20	Alive	Auyu	4/4	+	++/+	++/-	±	-	-	+/-	-	-	-	5
ALS 12	Definite	2010/'11/'12	Bosma	64	M	53/'01	11	Alive	Auyu	3/4	+	++/++	+/-	++	-	-	-	-	-	-	6
ALS 13	Definite	2012	Asset	70	F	68/'10	2	Alive	Auyu	3/3	-	+/+	-/-	-	-	-	-	-	-	-	-
ALS 14	Probable	2012	Pies	65	F	60/'07	5	Alive	Auyu	3/4	-	+/+	-/-	-	-	-	-	-	-	-	-
ALS 15	Definite	2007	Bade	64	F	62/'05	2	2007	Maluku	3/3	+	++/++	-/-	-	+	-	-	-	-	+	7
ALS 16	Definite	2008/'10/'11	Yibin	25	M	13/'99	13	2012	Auyu	3/4	+	++/+	++/++	-	-	-	-	-	-	+	-
ALS 17	Definite	2012	Ogorito	70	M	66/'08	4	Alive	Auyu	3/3	+	+/+	+/+	-	-	-	-	-	-	+	-

Abbreviations: ALS = amyotrophic lateral sclerosis; BK = bradykinesia; BS = Babinski sign; DTR = deep tendon reflex; Dys = dysphagia/dysarthria; F = female; Fasc = fasciculation; LMN = lower motor neuron signs; M = male; MA = muscle atrophy with weakness; NC = not confirmed by examination; P = parkinsonism; PI = postural impairment; R = rigidity; T = tremor; TA = tongue atrophy; U/L: upper limbs/lower limbs; UMN = upper motor neuron signs.
Age: at the time of final survey; Duration: between subjective onset of illness and last survey or death; Alive: alive in 2012; Onset: subjective onset of illness; Deep tendon reflexes, -: absent, 1: hypoactive, 2: normal, 3: hyperactive, 4: markedly hyperactive with ankle clonus.
Other signs are classified as: -: absent; ±: questionably present; +: present; ++: markedly present.
Family history 1: cousin (possible ALS); 2: grandfather (NC); 3: sister (ALS15); 4: cousin's daughter (P16); 5: cousin (NC); 6: father (NC); 7: sister (ALS 7); 8: sister (P 6) and cousin (NC); 9: father (NC); 10: son: (NC); 11: sister (ALS-P 1) and cousin (NC); 12: mother and father (NC); 13: grandmother (NC); 14: mother (NC); 15: ALS 10 (mother's cousin).

For peer review only

Okumiya

Table 2. Relevant characteristics and neurological signs in cases with overlap of ALS and parkinsonism with/without cognitive impairment in Papua, 2001–2012.

Case number	ALS criteria Yahr stage	Survey year	Village	Age	Sex	Onset (age/year)	Duration (year)	Death (year)	Tribe (origin)	UMN signs		LMN signs		Bulbar signs		Parkinsonian sign				Cognitive impairment	Family history
										DTR	BS	MA	Fasc	TA	Dys	T	R	BK	PI		
										U/L		U/L	U/L			U/L	U/L				
ALS-parkinsonism (n = 13)																					
ALS-P 1	Possible-IV	2008	Pies	65	F	60/'03	6	2009	Auyu	3/3	+	+/-	-/-	-	-	+/+	++/++	++	+	-	8
ALS-P 2	Possible-III	2010/'11	Ogorito	76	M	70/'05	6	2011	Auyu	3/3	+	+/±	-/-	-	-	+/+	+/+	+	+	-	-
ALS-P 3	Possible-III	2010/'11/'12	Homilikia	69	M	65/'08	4	Alive	Auyu	3/2	+	+/-	-/-	-	-	+/+	+/+	+	+	-	-
ALS-P 4	Definite-III	2008	Bade	34	M	18/'92	17	2009	Muyu	3/3	+	+/-	-/-	++	++	-	+/+	+	+	+	9
ALS-P 5	Definite-III	2012	Sien	60	M	58/'10	2	Alive	Auyu	3/3	+	+/+	-/-	-	-	-	+/+	+	+	+	-
ALS-P 6	Definite-III	2012	Benggo	50	M	48/'10	2	Alive	Auyu	2/2	+	+/+	-/-	+	+	-	+/-	+	+	+	-
ALS-P 7	Possible-II	2001	Bade	36	M	NC	NC	NC	Auyu	3/3	+	-/-	-/-	-	-	+	+	+	-	-	-
ALS-P 8	Possible-II	2008/'10	Bade	23	M	11/'98	12	Alive	Auyu	3/4	+	-/-	-/-	-	-	+/+	+/±	+	-	-	-
ALS-P 9	Possible-III	2010/'11	Ogorito	76	M	70/'05	6	2011	Auyu	3/3	+	-/-	-/-	-	-	+/-	+/+	+	+	-	-
ALS-P 10	Possible-III	2008/'10/'11	Yibin	52	M	44/'03	8	Alive	Auyu	3/3	+	-/-	-/-	-	-	+/-	+/+	+	+	-	-
ALS-P 11	Possible-III	2011	Getio	55	F	53/09	2	2011	Auyu	3/3	+	±/-	-/-	-	-	+/+	+/+	+	+	-	-
ALS-P 12	Possible-IV	2001	Bade	40	M	NC	NC	NC	Auyu	3/3	+	-/-	-/-	-	+	+	+	+	+	+	-
ALS-P 13	Possible-III	2012	Bosma	52	M	48/'08	4	Alive	Auyu	3/4	+	-/-	-/-	-	-	-	++/+	+	+	+	-

Abbreviations: ALS = amyotrophic lateral sclerosis; BK = bradykinesia; BS = Babinski sign; DTR = deep tendon reflex; Dys = dysphagia/dysarthria; F = female; Fasc = fasciculation; LMN = lower motor neuron signs; M = male; MA = muscle atrophy with weakness; NC = not confirmed by examination; P = parkinsonism; PI = postural impairment; R = rigidity; T = tremor; TA = tongue atrophy; U/L: upper limbs/lower limbs; UMN = upper motor neuron signs.

Age: at the time of final survey; Duration: between subjective onset of illness and last survey or death; Alive: alive in 2012; Onset: subjective onset of illness; Deep tendon reflexes, -: absent, 1: hypoactive, 2: normal, 3: hyperactive, 4: markedly hyperactive with ankle clonus.

Other signs are classified as: -: absent; ±: questionably present; +: present; ++: markedly present.

Family history 1: cousin (possible ALS); 2: grandfather (NC); 3: sister (ALS15); 4: cousin's daughter (P16); 5: cousin (NC); 6: father (NC); 7: sister (ALS 7); 8: sister (P 6) and cousin (NC); 9: father (NC); 10: son: (NC); 11: sister (ALS-P 1) and cousin (NC); 12: mother and father (NC); 13: grandmother (NC); 14: mother (NC); 15: ALS 10 (mother's cousin).

For peer review only

For peer review only - http://bmjopen.bmj.com/site/about/guidelines.xhtml

Okumiya

Table 3. Relevant characteristics and neurological signs in cases with parkinsonism with/without cognitive impairment in Papua, 2001–2012.

Case number	ALS criteria Yahr stage	Survey year	Village	Age	Sex	Onset (age/year)	Duration (years.)	Death (year)	Ethnicity	UMN signs DTR U/L	UMN signs BS	LMN signs MA U/L	LMN signs Fasc U/L	Bulbar signs TA	Bulbar signs Dys	Parkinsonian sign T U/L	Parkinsonian sign R U/L	Parkinsonian sign BK	Parkinsonian sign PI	Cognitive impairment	Family history
Parkinsonism (n = 16)																					
P 1	Yahr IV	2001	Kepi	50	M	NC	NC	NC	Jakai	2/2	-	-/-	-/-	-	-	+/+	+/+	++	+	-	-
P 2	Yahr II	2001	Kepi	50	M	NC	NC	NC	Jakai	2/2	-	-/-	-/-	-	-	+	+	+	-	-	-
P 3	Yahr IV	2002	Emete	52	M	49/'99	3	NC	Jakai	2/2	-	-/-	-/-	-	-	+	+	+	+	-	-
P 4	Yahr II	2002	Senggo	41	M	36/'97	5	NC	Citak	2/2	-	-/-	-/-	-	-	+	+	-	-	-	-
P 5	Yahr I	2007	Mur	65	F	61/'03	4	NC	Jakai	2/3	-	-/-	-/-	-	-	+/-	+/-	-	-	-	10
P 6	Yahr IV	2008	Pies	60	F	55/'03	6	2009	Auyu	2/2	-	-/-	-/-	-	-	+/+	+/+	++	+	-	11
P 7	Yahr IV	2008/'10	Mopio	52	F	47/'05	5	Alive	Auyu	3/3	-	-/-	-/-	-	-	++/+	+/+	++	+	-	-
P 8	Yahr II	2011	Sien	57	F	49/03	9	2012	Auyu	3/3	-	-/-	-/-	-	-	+/+	+/+	+	-	-	12
P 9	Yahr IV	2007/'08/'10/'[illegible]	Bade	46	F	40/'06	6	Alive	Auyu	3/3	-	-/-	-/-	-	-	+/+	++/+	+	+	-	13
P 10	Yahr II	2011/'12	Asset	38	M	27/'01	11	Alive	Auyu	3/3	-	-/-	-/-	-	-	+/-	+/-	+	-	-	-
P 11	Yahr III	2011/'12	Sien	54	F	40/'98	14	Alive	Auyu	3/3	-	-/-	-/-	-	-	++/+	++/+	+	+	-	-
P 12	Yahr II	2011/'12	Memes	66	F	64/10	2	Alive	Auyu	3/3	-	-/-	-/-	-	-	+/+	+/+	+	-	-	14
P 13	Yahr II	2012	Harapan	60	M	57/09	3	Alive	Auyu	2/2	-	-/-	-/-	-	-	-	+/+	-	-	-	-
P 14	Yahr II	2007	Mur	50	M	38/'95	12	NC	Auyu	2/4	+	-/-	-/-	-	-	+	+	+	-	-	-
P 15	Yahr I	2007	Homilikia	50	M	NC	NC	Alive	Auyu	2/3	+	-/-	-/-	-	-	+/-	+/±	-	-	-	-
P 16	Yahr III	2011	Osso	35	F	33/'09	2	2011	Auyu	3/2	-	-/-	-/-	-	-	+/+	+/+	+	+	+	15

Abbreviations: ALS = amyotrophic lateral sclerosis; BK = bradykinesia; BS = Babinski sign; DTR = deep tendon reflex; Dys = dysphagia/dysarthria; F = female; Fasc = fasciculation; LMN = lower motor neuron signs; M = male; MA = muscle atrophy with weakness; NC = not confirmed by examination; P = parkinsonism; PI = postural impairment; R = rigidity; T = tremor; TA = tongue atrophy; U/L: upper limbs/lower limbs; UMN = upper motor neuron signs.

Age: at the time of final survey; Duration: between subjective onset of illness and last survey or death; Alive: alive in 2012; Onset: subjective onset of illness; Deep tendon reflexes, -: absent, 1: hypoactive, 2: normal, 3: hyperactive, 4: markedly hyperactive with ankle clonus.

Other signs are classified as: -: absent; ±: questionably present; +: present; ++: markedly present.

Family history 1: cousin (possible ALS); 2: grandfather (NC); 3: sister (ALS15); 4: cousin's daughter (P16); 5: cousin (NC); 6: father (NC); 7: sister (ALS 7); 8: sister (P 6) and cousin (NC); 9: father (NC); 10: son: (NC); 11: sister (ALS-P 1) and cousin (NC); 12: mother and father (NC); 13: grandmother (NC); 14: mother (NC); 15: ALS 10 (mother’s cousin).

For peer review only - http://bmjopen.bmj.com/site/about/guidelines.xhtml

Table 4. Cases of ALS and parkinsonism in the field sites

	ALS criteria	Case No.	Crude point prevalence rate (95%CI) among ALS/ ALS-P/ Parkinsonism (2007* or 2010#)
Bade 2007–2012 (population 3800)			
ALS	Definite	ALS 5*	105 (2-208) /100,000 in 2007*
(n=4)	Definite	ALS 6*	
	Definite	ALS 7*	
	Definite	ALS 15*	
ALS-parkinsonism	Definite	ALS-P 4*	53 (0-126) /100,000 in 2007*
(n=2)	Possible	ALS-P 8*	
Parkinsonism (n=1)		P 9*	26 (0-78) /100,000 in 2007*
Ia river 2007–2012 (population 4500)			
ALS	Definite	ALS 4	133(27-240) /100,000 in 2010#
(n=7)	Probable	ALS 8#	
	Probable	ALS 10#	
	Probable	ALS 11#	
	Definite	ALS 12#	
	Definite	ALS 13#	
	Definite	ALS 17#	
ALS-parkinsonism	Possible	ALS-P 2#	89 (2-176) /100,000 in 2010#
(n=4)	Possible	ALS-P 3#	
	Possible	ALS-P 9#	
	Possible	ALS-P 13#	
Parkinsonism		P 10#	67 (0-142) /100,000 in 2010#
(n=3)		P 15#	
		P 16#	
Edera and Dumut rivers 2008–2012 (population: 4100)			
ALS	Definite	ALS 9#	73 (0-156) /100,000 in 2010#
(n=3)	Probable	ALS 14#	
	Definite	ALS 16#	
ALS-parkinsonism	Possible	ALS-P 1	98 (2-193) /100,000 in 2010#
(n=5)	Definite	ALS-P 5#	
	Definite	ALS-P 6#	
	Possible	ALS-P 10#	
	Possible	ALS-P 11#	
Parkinsonism		P 6	122 (15-229) /100,000 in 2010
(n=6)		P 7#	
		P 8#	
		P 11#	
		P 12#	
		P 13#	

*:Active cases in 2007 in Bade
#:Active cases in 2010 in the villages along Ia, Edera and Dumut rivers
95%CI: 95% confidence interval

For peer review only - http://bmjopen.bmj.com/site/about/guidelines.xhtml

Amyotrophic lateral sclerosis and parkinsonism in Papua, Indonesia: 2001–2012 survey results

Kiyohito Okumiya[1,2], Taizo Wada[2], Michiko Fujisawa[2], Masayuki Ishine[3], Eva Garcia del Saz[4], Yutaka Hirata[5], Shigeki Kuzuhara[6], Yasumasa Kokubo[7], Harumichi Seguchi[8], Ryota Sakamoto[9], Indrajaya Manuaba[10], Paulina Watofa[10], Andreas L. Rantetampang[11], Kozo Matsubayashi[2]

[1]Research Institute for Humanity and Nature, Kyoto, Japan
[2]Center for Southeast Asian Studies, Kyoto University, Kyoto, Japan
[3]Yasugi Clinic, Shimane, Japan
[4]Center for Regional & International Collaboration, Kochi University, Kochi, Japan
[5]Kitaakita Municipal Hospital, Akita, Japan
[6]Faculty of Health Science, Suzuka University of Medical Science, Mie, Japan
[7]Graduate School of Regional Innovation Studies, Mie University, Mie, Japan
[8]Faculty of Medicine, Kochi University, Kochi, Japan
[9]Hakubi Center for Advanced Research, Kyoto University, Kyoto, Japan
[10]Faculty of Medicine, Cenderawasih University, Papua, Indonesia
[11]Faculty of Public Health, Cenderawasih University, Papua, Indonesia

Correspondence to: Kiyohito Okumiya,
Research Institute for Humanity and Nature
Motoyama 457-4, Kamigamo, Kita-ku, Kyoto 603-8047, Japan
Tel: +81-75-707-2411, Fax: +81-75-707-2509
E-mail: okumiyak@chikyu.ac.jp
Center for Southeast Asian Studies
Kyoto University
46, Shimoadachi-cho, Yoshida , Sakyo-ku, 606-9501, Japan
Tel: +81-75-753-7320
E-mail: okumiya@cseas.kyoto-u.ac.jp

Abstract:296 words
Text:5299 words
4 tables and 3 figures

For peer review only - http://bmjopen.bmj.com/site/about/guidelines.xhtml

ABSTRACT

Objective: Only one prior follow-up study of amyotrophic lateral sclerosis (ALS) and parkinsonism in Papua, Indonesia has been carried out since a survey undertaken from 1962 to 1981 by Gajdusek and colleagues. Therefore, to clarify the clinical epidemiology of ALS and parkinsonism in the south coastal region of Papua, the clinical characteristics and prevalence of the diseases in this region were examined and assessed.

Methods: Cases of ALS and parkinsonism were clinically examined during a 2001–2012 survey in Bade and other villages along the Ia, Edera, Dumut, and Obaa rivers in Papua, Indonesia. Possible, probable, and definite ALS were diagnosed clinically by certified neurologists on the basis of El Escorial Criteria. The criteria for diagnosis of parkinsonism were the presence of at least 2 of the 4 following signs: tremor, rigidity, bradykinesia, and postural impairment with a progressive course.

Results: During the survey, 46 cases of ALS and/or parkinsonism were diagnosed within a population range of 7000 (2001–2002) to 13,900 (2007–2012). The 46 cases consisted of 17 probable-definite cases of ALS, including 3 of cognitive impairment (CI); 13 cases of overlap of possible, probable, or definite ALS and parkinsonism, including 5 cases of CI; and 16 cases of parkinsonism, including 1 case of CI. The crude point prevalence rate of pure ALS was estimated as at least 73 (0–156) to 133 (27–240)/100,000 people and that of the overlap of ALS and parkinsonism was at least 53 (0–126) to 98 (2–193)/100,000 in 2007 or 2010 in some regions.

Conclusions: While the prevalence of ALS has decreased over the past ~30-35 years, it remains higher than the global average. There was a high prevalence of overlap of ALS, parkinsonism, and CI, which has been previously reported in Guam and Kii.

For peer review only

Strengths and limitations of this study

- This study is unique in the epidemiological survey of neurodegenerative diseases during the period between 2001 and 2012 in Papua, Indonesia, one of the foci with the highest incidence of ALS in the world.
- This study recognized the clinical findings of significant overlap of ALS with parkinsonism and cognitive impairment.
- This study was based only on clinical findings and there are the limitations of not having electromyogram data, DNA analysis, or any autopsy data from this population. Without such data, it is difficult to be certain how these patients fit into sporadic ALS or Parkinson's disease and how they compare with ALS/PDC on Guam and in the Kii peninsula.

For peer review only - http://bmjopen.bmj.com/site/about/guidelines.xhtml

INTRODUCTION

Between 1962 and 1980, Gajdusek et al. reported 97 cases of amyotrophic lateral sclerosis (ALS), 18 cases of parkinsonism, and 18 cases of poliomyeloradiculitis (PMR) in the south coastal area of Papua (former Irian Jaya, Indonesia), which had a population of 7,000. The Ia River is in the epicenter of the high-incidence focus of ALS and parkinsonism in Papua.[1,2] Along with Guam and Kii in Japan, Papua (Indonesia) has been considered one of the foci with the highest incidence of ALS in the world.[1-7] The high incidence of ALS and parkinsonism-dementia complex (PDC) in Guam and Kii reportedly no longer exists.[8,9] In 1987, Spencer et al. reported only 2 cases of ALS and three cases of parkinsonism in the villages along the Ia River. In 1990, no cases of ALS and three cases of parkinsonism were reported along the same river; thus, decline or disappearance of ALS was suspected to have also occurred in Papua.[10] No follow-up surveys on ALS and parkinsonism have been conducted in Papua since 1991.

To clarify the clinical epidemiological characteristics of ALS and parkinsonism in the south coastal region of Papua, we conducted a survey of neurodegenerative diseases documented between 2001 and 2012.

METHODS

The survey sites for between 2001 and 2002 (total population, 7000) were the villages of Kepi (population, 800), Emete (800), Ogoto (400), Senggo (2000), and Bade (3000).[11,12] For between 2007 and 2012, the survey sites were the villages along 4 rivers (total population, 13,900), namely Bade along the Digul River (population, 3800

For peer review only - http://bmjopen.bmj.com/site/about/guidelines.xhtml

For peer review only

people), Mur along the Mappi River (1500), the 8 villages along the Ia River (4500), and the 12 villages along the Edera and Dumut rivers (4100 people). The 8 villages along the Ia River were Gimikya (800), Geturki (500), Homlikya (700), Kobeta (600), Asset (600), Osso (300), Bosma (500), and Ogorito (500). The 12 villages along the Edera and Dumut rivers were Benggo (200), Kokoya (500), Sahapikya (400), Harapan (400), Yibin (300), Mopio (300), Pies (300), Memes (300), Habeske (300), Muya (300), Getio (200), and Sien (600; Figure 1). At least 2 of 6 Japanese-certified neurologists (KO, MF, YH, SK, YK, and KM) visited each village with a local neurologist (IM).

The village leaders and medical staff members of the primary healthcare centre in each village agreed to take part in the survey, which was conducted with the permission of the Department of Health of the Papua Provincial Government in cooperation with Cenderawasih University. The purpose of the neurological examinations was explained to all of the villagers. All the subjects volunteered to participate after the announcement of availability of neurological check-ups, which were performed in the community health centres or homes in each field site. While this was not a complete inventory survey, we asked each medical doctor or health staff, and village leader to call all patients with neurological signs or symptoms, including muscle weakness, gait disturbance, tremor, bradykinesia, or cognitive impairment (CI), in each village. The patients who were called also volunteered to participate. Indonesian collaborators and the co-authors (an English teacher in Bade Senior High School and Indonesian neurologist IM, and staff from Cenderawasih University) who spoke both English and Indonesian; local people who spoke both Indonesian and the local language in each

For peer review only

village; and co-author EG, who spoke English, Indonesian, and Japanese, cooperatively served as translators. Written informed consent was obtained from all the study participants with the support of their family members. All the participants agreed to undergo neurological examinations, and none of the patients declined annual re-examination. The survey was approved by the ethics committees of the Research Institute for Humanity and Nature in Japan and Cenderawasih University in Papua, Indonesia.

Among the many types of neurological and non-neurological cases encountered, diagnoses of ALS, parkinsonism, and CI were based on clinical examinations and discussions among the participating neurologists. ALS was classified as 'definite', 'probable', 'possible', and 'suspected' on the basis of combinations of upper motor neuron (UMN) and lower motor neuron (LMN) signs according to the El Escorial Criteria of the World Federation of Neurology.[13] Probable and definite ALS are defined as pure ALS in this report. A diagnosis of parkinsonism required the presence of at least 2 of the following 4 signs: tremor, rigidity, bradykinesia, and postural impairment with a progressive course without evidence/history of vascular accidents or history of taking drugs known to induce parkinsonism. The degree of disability of patients with parkinsonism was classified according to the scale of Hoehn and Yahr.[14] Overlap of possible, probable, or definite ALS with parkinsonism was defined as ALS-parkinsonism. CI was diagnosed when loss of memory or impairments of language usage, praxis, or executive functions were identified during interview and clinical examination by neurologists. As cognitive functional test such as the mini–mental state examination was not performed for all of the participants, patients with complications

For peer review only - http://bmjopen.bmj.com/site/about/guidelines.xhtml

For peer review only

of functional disabilities attributable to CI in activities of daily living or in their livelihood were screened during the interview of both the patients and their family members. PMR was evaluated using the definition of Gajdusek et al. that PMR is a subacute paralytic condition reminiscent of the Landry-Guillain-Barre syndrome, which combines radicular, or perhaps neuritic elements with more acute onset and sometimes asymmetric paralysis.[1,2] Neurological cases with overlapping cerebellar signs (2 cases) were excluded from this study.

In Bade and the villages along the Ia, Edera, and Dumut rivers, not only were new cases were identified but also previously diagnosed cases were followed up and assessed neurologically and documented in 2007, 2008, 2010, 2011, and 2012. Death years were confirmed, and the durations between subjective onset of illness and the last survey or death were recorded as durations of subjective illness.

The size of the populations of Bade and the villages along the Ia, Edera, and Dumut rivers were determined from a report by the public office of Edera district in 2006.[15] We were informed by the village leaders of the populations of other villages in the survey for the period 2001–2002.

RESULTS

Clinical types of ALS and/or parkinsonism

We identified 46 cases with signs of ALS and/or parkinsonism, including 17 cases with pure ALS (Table 1), 13 with ALS-parkinsonism (Table 2), and 16 with parkinsonism (Table 3). We found no case of pure or dominant dementia and PMR consistent with the

For peer review only - http://bmjopen.bmj.com/site/about/guidelines.xhtml

For peer review only

definition by Gajdusek et al; similarly, no cases of PMR were found by Spencer et al. in 1987 or 1990. [10] Relevant information about the cases, including neurological signs, age at last survey, age and year of onset, disease duration, certified death year, and ethnicity, is shown in Tables 1–3. The male-to-female sex distribution was 10:7 (male, 59%) for ALS, 11:2 (85%) for ALS-parkinsonism, and 8:8 (50%) for parkinsonism. The mean age at last survey was 51.3 years for ALS, 52.9 years for ALS-parkinsonism, and 51.6 years for parkinsonism. The mean age at onset was 46.2 years (range, 11–68 years) for pure ALS, 49.5 years (11–70 years) for ALS-parkinsonism, and 45.8 years (27–64 years) for parkinsonism. The mean duration of subjective illness was 7.0 years (2–20 years) for pure ALS, 6.3 years (2–17 years) for ALS-parkinsonism, and 6.3 years (2–14 years) for parkinsonism. We observed overlapping CI in three (18%) of the 17 cases of pure ALS, 5 (38%) of the 13 cases of ALS-parkinsonism, and 1 (6%) of the 16 cases of parkinsonism.

During the follow-up surveys of the cases in Bade, Ia, and Edera rivers, we observed the following changes in diagnosis: 1 case, from possible ALS to probable ALS (case ALS10); 2 cases, from parkinsonism to ALS-parkinsonism (cases ALS-P 2 and ALS-P 3); and 1 case, from probable ALS to definite ALS (case ALS16) complicated with CI. We also identified an instance of a shared family history of parkinsonism (P 6) and ALS-parkinsonism (ALS-P 1). There were family histories of neurological disorders in 7 of 17 cases (41%) of pure ALS, 2 (15%) of 13 cases of ALS-parkinsonism, and 6 (38%) of 16 cases of parkinsonism (Tables 1–3).

Cases of ALS and parkinsonism in Bade and the villages along the Ia, Edera, and

For peer review only - http://bmjopen.bmj.com/site/about/guidelines.xhtml

Dumut rivers between 2007 and 2012

Between 2007 and 2012, we identified 4 cases of pure ALS, 2 cases of ALS-parkinsonism, and 1 case of parkinsonism in Bade (population, 3800 people). In addition, we found 7 cases of pure ALS, 4 cases of ALS-parkinsonism, and three cases of parkinsonism in the villages (population, 4500 people) along the Ia river. Between 2008 and 2012, we identified three cases of pure ALS, 5 cases of ALS-parkinsonism, and 6 cases of parkinsonism in the villages (population, 4100 people) along the Edera and Dumut rivers (Table 4).

In the 2007 survey, there were 4 active cases of pure ALS (crude point prevalence rate, 105 [2–208]/100,000 people), 2 cases of ALS-parkinsonism (53 [0–126]/100,000 people), and 1 case of parkinsonism (26 [0–78]/100,000 people) in Bade. In 2010, there were 6 active cases of pure ALS (133 [27–240]/100,000 people), 4 cases of ALS-parkinsonism (89 [2–176]/100,000 people), and three cases of parkinsonism (67 [0–142]/100,000 people) in the villages along the Ia River. In 2010, there were three active cases of pure ALS (73 [0–156]/100,000 people), 4 cases of ALS-parkinsonism (98 [2–193]/100,000 people), and 5 cases of parkinsonism (122 [15-229]/100,000 people) in the villages along the Edera and Dumut rivers (Table 4).

In the survey during the period 2007–2012, the number of each clinical type (ALS, ALS-parkinsonism, and parkinsonism) was shown respectively for Bade (4, 4, and 1); the villages along the Ia River, namely Ogorito (1,2, and 0), Bosma (1, 1, and 0), Homlikya (1, 1, and 1), Gimikya (1, 1, and 0), Geturki (1, 0, and 0), Asset (1, 0, and 1), and Osso (1, 0, and 1); and the villages along the Edera and Dumut rivers, namely Yibin

For peer review only - http://bmjopen.bmj.com/site/about/guidelines.xhtml

(1, 1, and 0), Sien (0, 1, and 2), Pies (1, 0, and 1), Benggo (0, 1, and 0), Harapan (0, 0, and 1), Mopio (0, 0, and 1), Memes (0, 0, and 1), Muya (1, 0, and 0), and Getio (0, 1, and 0).

Case reports

Case ALS 12

A 64-year-old (in 2012) man of the Auyu tribe, a resident of Bosma along the Ia River, had a history of progressive gait disturbance and motor weakness for 9 years in 2010; these symptoms were found to have progressed based on the findings from the follow-up check-ups between 2011 and 2012. Neurological examination revealed UMN and LMN signs, including a positive Babinski sign, hyperreflexia with ankle clonus, muscle atrophy, and weakness in the tongue and upper and lower limbs bilaterally, and fasciculation in the upper limbs and tongue. He had no CI or sensory disturbance. We diagnosed him as having definite ALS (Figure 2-A–D). The subject had ALS, and his brother reported that their father showed tremor and gait disturbance, which were interpreted to be consistent with parkinsonism.

Case ALS-CI 17

A 70-year-old (in 2012) man of the Auyu tribe, a resident of Ogorito along the Ia River, had a history of progressive memory loss for 4 years and slow gait for 2 years. Neurological examination revealed UMN and LMN signs, including a positive Babinski sign, hyperreflexia in the jaw jerk, bilateral upper and lower limbs, and muscle atrophy, weakness, and fasciculation in the distal upper and lower limbs bilaterally. He had

For peer review only - http://bmjopen.bmj.com/site/about/guidelines.xhtml

For peer review only

fasciculation in the tongue and respiratory muscle weakness with a low oxygen saturation level (SpO_2, 86%). He also had memory disturbances accompanied by positive snout and palmomental reflexes and difficulty with mental activities of daily living. He had no sensory disturbance. We diagnosed him as having definite ALS with CI (Figure 3A).

Case ALS-P 1

A 65-year-old (in 2008) woman of the Auyu tribe, a resident of Pies along the Edera River, had a history of progressive motor weakness and gait disturbance for 5 years. She could not walk without support. Neurological examination revealed a resting tremor, cogwheel rigidity in the upper and lower limbs, bradykinesia throughout, postural impairment, UMN and LMN signs with a positive Babinski sign, hyperreflexia in the upper and lower limbs bilaterally, and thenar and hypothenar muscle atrophies. She had no CI or sensory disturbance. We diagnosed her as having ALS-parkinsonism and, more specifically, with severe parkinsonism (Hoen and Yahr IV) simultaneously complicated by motor neuron signs indicative of possible ALS. She died in 2009. Her younger sister was case P 6. The subject had ALS-parkinsonism, and her sister reported that their cousin showed tremor and gait disturbance, which were interpreted to be consistent with parkinsonism (Figure 3B).

Case ALS-P 2

A 76-year-old (in 2011) man of the Auyu tribe, a resident of Ogorito, had a history of tremor in both hands for 5 years in 2010. Neurological examination revealed cogwheel rigidity in his upper and lower limbs, bradykinesia, and UMN signs with hyperreflexia

For peer review only - http://bmjopen.bmj.com/site/about/guidelines.xhtml

For peer review only

in bilateral upper and lower limbs. We diagnosed him as having parkinsonism (Hoen and Yahr II) in 2010. He was examined again in 2011, by which time his parkinsonism was complicated by not only postural impairment but also muscle weakness and atrophy, and spasticity of the upper and lower limbs with Babinski signs. He had no CI or sensory disturbance. We diagnosed him as having parkinsonism (Hoen and Yahr III) with possible ALS; parkinsonism was accompanied by ALS later. He died in 2011 of unknown causes.

We also diagnosed another case of ALS-P3 parkinsonism (Hoen and Yahr III) with possible ALS; parkinsonism was accompanied by ALS later.

Case ALS-P-CI 6

A 50-year-old (in 2012) man of the Auyu tribe, a resident of Benggo along the Edera River, had a history of dysarthria for 2 years. Neurological examination revealed UMN and LMN signs with a positive Babinski sign, muscle atrophy, and weakness in the tongue, proximal upper limb, shoulder, and tibialis anterior muscles. He had cogwheel rigidity in the wrist and elbow, bradykinesia throughout, a positive Myerson sign, and impairment of coordination and postural reflex. He had memory loss, bradyphrenia, slow speech, and positive snout and palmomental reflexes with difficulty performing activities of daily living. We diagnosed him as having a definite ALS and parkinsonism (Hoen and Yahr III) with CI (Figure 3C).

Case P 7

A 52-year-old (in 2010) woman of the Auyu tribe who lived in Mopio along the Edera

For peer review only - http://bmjopen.bmj.com/site/about/guidelines.xhtml

River had a history of progressive gait disturbance with parkinsonian signs, including resting tremor, cogwheel rigidity, anterior bending, and bradykinesia for 3 years in 2008. In 2008, we diagnosed her as having parkinsonism (Hoen and Yahr II). By the time of the follow-up survey in 2010, she could not stand by herself and had postural impairment with parkinsonian hands bilaterally. She had no UMN or LMN signs, no CI, and no sensory disturbance. We diagnosed her as having parkinsonism (Hoen and Yahr IV; Figure 3D).

DISCUSSION

Internal migration and migration from outside the province are the most significant causes of demographic, social, and cultural change in Papua. The populations in the survey sites were composed of former hunter-gatherers who lived in the forest and who were brought out of the forest and assembled in villages by Dutch colonialists. The villages along the Ia River were founded in 1937–1951.[10] The emigration program called transmigration (*transmigrasi*) started in Irian Jaya in 1964. Local migrants and transmigrants from outside Irian Jaya increased, resulting in various changes, including the use of food and medicine.[16,17] Considering that Gadjusek et al. found out the high prevalence of ALS at the first survey in 1962, the neurodegenerative disease existed before the introduction of manufactured items.[1]

In the present survey, we performed full neurological examinations in 46 subjects with ALS and/or parkinsonism between 2001 and 2012. Among the subjects, 17 were probable-definite cases of pure ALS, 13 were overlapping ALS (possible, probable, or definite) and parkinsonism, and 16 were parkinsonism. Nine cases of ALS and/or

For peer review only - http://bmjopen.bmj.com/site/about/guidelines.xhtml

parkinsonism had a complication of CI. During the study period, the diagnosis in some patients changed from pure parkinsonism to ALS-parkinsonism, and their disease progressed in severity. Some subjects had both ALS and parkinsonism on their first examination. It is thus evident that overlapping ALS and parkinsonism with or without CI still exists in the specified region of Papua.

Gajdusek et al. reported diagnosing 97 cases (57 cases clinically confirmed and 40 cases not clinically confirmed) of ALS during the 6 surveys they conducted from 1974 to 1981.[1] However, they reported neurological findings of only 32 of these cases, in only 16 of which the reported findings of UMN, LMN, and parkinsonian signs justify a diagnosis of probable or definite ALS. The 16 cases comprise 12 cases of pure ALS and 4 cases of overlapping ALS-parkinsonism. Among the 32 ALS patients, three had a positive family history (9%); that is, 2 had sisters with ALS, and 1 who had ALS-parkinsonism had a family history of ALS. None of the ALS patients had a family history of parkinsonism. The authors also reported their neurological findings in 13 subjects in whom parkinsonism was diagnosed. They reported their findings from full neurological examinations of 10 of these parkinsonism cases; according to their reported neurological signs, these comprised 8 patients of parkinsonism, including 5 with dementia and 2 with overlapping ALS-parkinsonism with dementia.[1] Among the 13 parkinsonism patients, three had a positive family history (23%). In summary, we believe that in the 26 cases reported by Gajdusek et al., the full neurological examination findings comprised 12 pure ALS cases (probable or definite), 6 overlapping ALS-parkinsonism, and 8 parkinsonism with/without dementia.[1] Spencer

For peer review only - http://bmjopen.bmj.com/site/about/guidelines.xhtml

et al reported 2 cases of ALS and three cases of parkinsonism, including 1 with dementia, in the survey in 1987. He also reported 3 subjects with parkinsonism overlapping UMN signs, including 1 with dementia in 1990. We have here reported 17 cases of pure ALS (probable or definite), 13 cases of overlapping ALS-parkinsonism, and 16 cases of parkinsonism with or without CI in the 46 cases we neurologically examined fully. Thus, we identified many cases of overlapping ALS and parkinsonism (n = 13, 28%) like that observed in previous reports (n = 6, 23% and n = 3, 38%).[1, 10] We also identified many cases with CI (n = 9, 20%) like that observed in previous reports (n = 7, 27% and n = 2, 25%).[1, 10] Functional disabilities attributable to CI were recognized in activities of daily living or in their livelihood in all the cases with CI in this report.

Gajdusek et al. reported 13 cases of ALS and three cases of parkinsonism in 1975 in the villages along the Ia River (population, 2000).[1] The 13 ALS cases consisted of 10 clinically confirmed ones and 3 others that were not clinically confirmed. We compared the prevalence of probable-definite ALS and parkinsonism according to the report of Gajdusek et al. with our more recent findings. A crude point prevalence of ALS consistent with clinically probable to definite ALS (El Escorial criteria) may be estimated between 500 (95% confidence interval, 191–809/100,000 people) and 650 (298–1002) and that of parkinsonism was 150 (0–320/100,000 people) in 1975 along the Ia River. In our 2010 survey (population, 4500), we identified 6 probable-definite cases of ALS in this region (ALS 8, 10–13, and 17; 133 [27—240]/100,000 people) and 7 of parkinsonism (possible ALS-P 2, 3, 9, and 13, and P 10, 15, and 16; 156 [40–271]/100,000 people).

For peer review only - http://bmjopen.bmj.com/site/about/guidelines.xhtml

For peer review only

Gajdusek et al. reported 8 cases of ALS and 6 cases of parkinsonism in 1975 in the villages along the Edera and Dumut rivers (population, 1950). The 8 cases of ALS consisted of 7 clinically confirmed cases and 1 unconfirmed case. The crude point prevalence of ALS consistent with clinically probable to definite ALS (El Escorial criteria) may be estimated between 359 (94–624)/100,000 people and 410 (127–694)/100,000 people, and that of parkinsonism was 308 (62–554)/100,000 people in 1975 along the Edera and Dumut rivers. In our survey of this region in 2010 (population, 4100), we identified 5 probable-definite cases of ALS (ALS 9, 14, and 16, and ALS-P 5, 6; 122 [15–229]/100,000 people) and 7 cases of parkinsonism (possible ALS-P 10 and 11, and P 7, 8, and 11–13; 171 [44–297]/100,000 people; Table 4). As this was not a complete inventory survey, the actual prevalence may be a little higher than our estimation.

The population of Bade increased by a factor of 4 during 31 years (from 1000 in 1975 to 3800 in 2006), and those of villages along the Ia River (from 2000 to 4500) and the Edera and Dumut rivers (from 1950 to 4100) have increased 2-fold.[1,15] The subjects born outside Papua were only 2 (Maluku) among the 46 subjects with ALS and/or parkinsonism with/without CI in this survey, ie 44 were of Papuan genetic stock and 2 (from Maluku) were non-Papuan. The prevalence of the diseases among the local Papuan residents in this survey may be underestimated compared with the former report after taking into account the increase in the numbers of local migrants inside and transmigrants from outside Papua.[16,17,18]

For peer review only - http://bmjopen.bmj.com/site/about/guidelines.xhtml

As the median age and life expectancy in the field sites in Papua are estimated to be lower than the global median and fewer people may live beyond the age of 40 or 50 years,[18,19] the prevalence of the diseases in this survey may be underestimated compared with the global mean. As the median age and life expectancy in 1975 may be lower than those in 2010,[20] the prevalence of the diseases in the report by Gajdusek et al. might be underestimated compared with that in our survey.

The prevalence of ALS in this report was found to be three times lower than found by Gajdusek et al. Even after taking into account the change of population and age structure in the field sites, our findings show that the prevalence of ALS appears to have decreased over the past ~30-35 years since the report of Gajdusek et al.[10] This is consistent with the previous report by Spencer et al. However, it is still much higher than the global mean (incidence, approximately 2/100,000; prevalence, approximately 6/100,000) [21-26]; in particular, the prevalence of overlapping ALS and parkinsonism is especially high in Papua.

In the report by Gajdusek et al., who provided village-by-village data showing the number of cases of ALS and parkinsonism, there were many cases in the remote villages such as Bosma, Pies, Homlikya, Yibin, Sien, and Ogorite, which were less visited and least developed.[1, 2] In this survey, many cases (three ALS/ALS-parkinsonism) were found in the remotest village of Ogorito along the Ia River. Two cases of ALS/ALS-parkinsonism were also found in Bosma, Homlikya, and Yibin; 1 ALS and 2 cases of parkinsonism in Sien; 1 ALS and 1 parkinsonism in Pies, which consisted also of remote villages. While there were 8 cases of

For peer review only - http://bmjopen.bmj.com/site/about/guidelines.xhtml

ALS/ALS-parkinsonism in Bade and 2 in Gimikya, nearest to the port of the Digul River, none was reported by Gajdusek et al. in those sites. This increase may be brought about by local migrants from the affected villages.

Overlap of ALS-parkinsonism and dementia has also been reported in patients with ALS/PDC in Guam and Kii, Japan.[4, 27-33] Hirano et al. provided clinicopathological evidence of 47 cases of PDC in Guam in 1961, comprising the following 3 groups: (1) 30 cases of PDC; (2) 8 cases of parkinsonism with dementia with clinical evidence of UMN impairment; and (3) 9 cases of parkinsonism with dementia with clinical evidence of UMN and LMN impairment. At least 15 of these cases (31.9%) had a family history of ALS, parkinsonism, or both.[4] Kuzuhara and Kokubo et al. surveyed neurological diseases in Kii, Japan, in the 1990s and found not only a continuously high incidence of ALS but also neuropathologically verified cases of PDC. ALS and PDC frequently affected the same individuals simultaneously and the members of the same family. These authors reported 37 cases of ALS/PDC between 1996 and 2006, a high proportion of whom (78%) had a positive family history, as well as clinical examples of pure ALS, PDC, and overlapping ALS and PDC. They also reported neuropathological changes common to cases of ALS and PDC in 12 cases of ALS/PDC, resembling those found in patients with ALS/PDC in Guam [28-33] In our more recent study in Papua, such as the previous studies in Kii, Japan, and Guam, we identified many cases of pure ALS and ALS overlapping with parkinsonism with/without CI. The prevalence of CI was lower in our study sites than in Guam and Kii.[4, 27-33] CI might have been underestimated, and mild CIs or some mild dementia might have been overlooked by our neurological examinations in the field.

For peer review only - http://bmjopen.bmj.com/site/about/guidelines.xhtml

Our study was based only on clinical findings, and there are the limitations of not having electromyogram data, DNA analysis data, or any autopsy data from this population. Without such data, it is difficult to be certain how these patients fit into sporadic ALS or Parkinson disease and how they compare with ALS/PDC on Guam and in the Kii peninsula. However, we did identify many patients with overlapping ALS-parkinsonism in this study. The overlap of ALS and/or parkinsonism with CI suggests that it may be the same disease entity as the ALS/PDC diagnosed in Guam and Kii.

The proportion of males in the populations of Bade, the villages along the Ia River, and the villages along the Edera and Dumut rivers were 53%, 51%, and 50%. The proportion of males was high especially among the subjects with ALS-parkinsonism (85%) and ALS (59%) compared with among those with pure parkinsonism (50%) in our report. The proportion of males was higher among the subjects with ALS (63%) and parkinsonism (76%) in the report by Gadjusek et al., in which there were 3 male cases (75%) among the 4 ALS-parkinsonism cases. The high risks for ALS and PDC among the males were recognized in Guam and Kii.[1,7,27]

Indigenous people of Papua still retain aspects of their traditional lifestyles, such as hunting, gathering, fishing, and eating *sago* (trunk of *Metroxylon* sp.). They also drink water from shallow wells. However, with the spread of a market economy, recent increases in the numbers of rubber plantations and progress in transportation have been causing lifestyle changes in the villages in the south coastal area of Papua. As the diseases are declining as in Guam and Kii, the disease aetiology in Papua may be dominated by environmental factors. Genetic, culture-related and/or family-specific

For peer review only - http://bmjopen.bmj.com/site/about/guidelines.xhtml

factors may also play a role as evidenced by the family history of disease in 40% of the subjects in this survey in Papua and 80% in Kii.

Various possible causal genetic and environmental factors for these conditions have been reported. The environmental factors of low concentrations of calcium and magnesium in drinking water were hypothesized. [1,34] There is a recent report showing an association between the increase in ALS incidence and the change in drinking water source in the Kii Peninsula.[35] Spencer et al. pointed out that this explanation was inconsistent with a declining prevalence of ALS in a sessile population dependent on an unchanging supply of river water, which this report may support.[10]

Spencer et al. reported the discovery that cycad seed was used as a poultice in Papua, as in Guam. [36] Cycad-derived products were shown as the risk factor of dementia, mild CI, and PDC in Guam with the epidemiological evidence.[37] Although cycad had been suspected as the causal factor of Kii ALS,[38] people in Japan, including the Kii Peninsula, had never consumed cycad as daily food, although a small amount of cycad flour might have been taken by a few people as an ingredient of herb medicine.[39] We did not examine the association of prior skin wounds and exposure to cycad poultice with the diseases. Skin change and pigmentary retinopathy were not examined in this survey.[40, 41] Further studies are needed.

Genetic abnormalities that had been previously established to be associated with familial or sporadic ALS, dementia, familial Parkinson disease, and parkinsonism were looked for in the cases in Kii, but none of them were identified.[28,42] Hermousura et

For peer review only - http://bmjopen.bmj.com/site/about/guidelines.xhtml

al. have discovered a nuclear mutation in the calcium/magnesium membrane ion channel transient receptor potential melastatin 7 (*TRPM7*) gene in a subset of patients with ALS/PDC in Guam.[43] They reported that a TRPM7 variant that is associated with altered sensitivity to magnesium may have contributed to the pathogenesis of ALS and PDC in 2 Guamanians. *TRPM7* was not found to be associated with ALS/PDC in the Kii peninsula of Japan.[44] Ishiura et al. have recently discovered *C9ORF72* repeat expansion in some cases of ALS in the Kii peninsula of Japan; this repeat expansion partly accounts for the high prevalence of ALS in this region.[45] However, the responsible gene(s) remain unproved in almost all cases of ALS/PDC in both Guam and Kii.

Various environmental factors and multiple genetic factors are suggested to be underlying causes of these diseases, but these links are so far unproven.[33,46]

In Guam, although the incidence of pure ALS has declined markedly, the prevalence of PDC is still high.[47] The prevalence of dementia among elderly Chamorros (earliest known inhabitants of the Mariana Islands, which include Guam) is also reportedly relatively high.[48] In addition, in Kii the incidence of pure ALS has declined markedly, and the prevalence of PDC and ALS/PDC or ALS with dementia recently increased.[30,33] These changing patterns of ALS/PDC incidence in Guam and Kii might be caused by changes in environmental and socioeconomic factors as well as aging.[48,49]

The mean age of onset in our cases was 46 years for ALS, 53 years for ALS-parkinsonism, and 46 years for parkinsonism. Thus, they were older than the

For peer review only - http://bmjopen.bmj.com/site/about/guidelines.xhtml

previously reported cases of ALS (33 years) and parkinsonism (43 years).[1] In our cases, the mean duration of subjective illness was 7.0 years for ALS, 6.3 years for ALS-parkinsonism, and 6.3 years for parkinsonism. The duration of ALS in our data was longer than not only the world mean of 3 years[21-26] but also the previously reported 3.5 years for ALS in Papua.[1] The mean age of onset may recently have increased, and the mean disease duration became longer in subjects with ALS. The prevalence rate of cases with bulbar sign was 37% in 30 of pure ALS or ALS-parkinsonian in our report, and it was as the same as in the report of Gadjdusek (40%) and classical ALS (39%).[2] The change in onset and course of ALS in recent years might be attributable to the aging population and changing environmental and socioeconomic factors.

On-going follow-up surveys should be performed to evaluate changes in the clinical types of ALS and/or parkinsonism with or without CI and accompanying changes in environmental and socioeconomic factors, as have occurred in Guam and Kii.

In conclusion, The prevalence of ALS in the Papuan disease focus has decreased over the past ~30-35 years but, in 2010, it remained higher than the global average. During this period, there has been an influx of migrants from inside and transmigrants from outside Papua that has increased the size and changed the composition of the population in the villages studied. There may be the possible impact of the expanded population on prevalence estimates. There are still many cases of pure ALS and overlapping ALS-parkinsonism and CI in Papua, as has previously been reported in Guam and Kii. The disease continues to impact native Papuans but may also affect a small number of

For peer review only - http://bmjopen.bmj.com/site/about/guidelines.xhtml

non-Papuan immigrants (2 of 46 or 3-4%) who adopt the local lifestyle. Environmental factors may play important etiologic role. Further on-going follow-up surveys focusing on the clinical epidemiology and aetiology of these neurodegenerative diseases in association with ecological and environmental factors in Papua are needed. Future studies, including neuropathological examinations, should be performed when the local villagers in this region of Papua, Indonesia, are willing to consent to autopsies.

ACKNOWLEDGMENTS

The authors wish to thank all the participants in the villages who volunteered for the survey, the village leaders, and Mappi Regency officials. We are deeply indebted to doctors Imelda Mandagi, Reginald Nangoy, and Christha Z. Tamburian and their staff at Puskesmas Bade, and to Arifin Wasaraka, Lazarus Revasy, Yosefina Griapon, Arius Togodly, Kerry Yarangga, and Dolfinus Y. Bouway at Cenderawasih University for their invaluable contributions to the study. Andre Liem and Herry Wondiwoy at Papua Tour Guides Community and S.E. Amos at Bade High School provided logistic and language assistance. On the Japanese side, we acknowledge the technical support and advice of Katsuyuki Eguchi (Nagasaki University), Yasushi Osaki (Kochi University), Hidekazu Tomimoto (Mie University), Taro Yamauchi (The University of Tokyo), Hideyuki Onishi (Doshisha Women's College), Tetsuya Inamura (Aichi Prefectural University), Wenling Chen, Yumi Kimura, Yasuko Ishimoto, and Hissei Imai (Kyoto University), and Yasuyuki Kosaka, Yukiko Kita, and Chizu Wada (Research Institute for Humanity and Nature).

For peer review only - http://bmjopen.bmj.com/site/about/guidelines.xhtml

AUTHOR CONTRIBUTIONS

Okumiya had full access to all the data in the study and takes responsibility for the integrity of the data and the accuracy of the data analysis.

Study concept and design: Okumiya, Matsubayashi.

Acquisition of data: Okumiya, Wada, Fujisawa, Ishine, Garcia del Saz Hirata, Kuzuhara, Kokubo, Seguchi, Sakamoto, Manuaba, Matsubayashi.

Analysis and interpretation of data: Okumiya,Wada, Sakamoto, Fujisawa, Matusbayashi.

Drafting of the manuscript: Okumiya,Wada, Sakamoto, Fujisawa, Matusbayashi.

Critical revision of the manuscript for important intellectual content: All authors.

Statistical analysis: Okumiya.

Obtained funding: Okumiya, Wada, Kuzuhara, Kokubo, Matsubayashi

Administrative, technical, or material support: Okumiya, Watofa, Rantetampang, Matsubayashi.

Study supervision: Okumiya, Matsubayashi.

COMPETING INTERESTS : None declared

FUNDING

K. Okumiya receives research support from Grant-in-Aid for Scientific Research (A) from the Japan Society for the Promotion of Science (JSPS). K. Matsubayashi receives research support from Grant-in-Aid for Scientific Research (A) from the JSPS and is an editor of the journal *Geriatrics & Gerontology International*. T. Wada receives research

For peer review only

support from Grant-in-Aid for Scientific Research (C) from the JSPS. Y. Kokubo and S. Kuzuhara receive support from Grants-in-Aid from the Research Committee of CNS Degenerative Diseases and the Research Committee on the Muro disease (Kii amyotrophic lateral sclerosis/Parkinsonism-dementia), the Ministry of Health, Labour and Welfare of Japan.

For peer review only

REFERENCES

1. Gajdusek DC, Salazar AM. Amyotrophic lateral sclerosis and parkinsonian syndromes in high incidence among the Auyu and Jakai people of West New Guinea. Neurology 1982;**32**:107–26.
2. Gajdusek DC. Foci of motor neuron disease in high incidence in isolated populations of East Asia and the Western Pacific. In: Lewis P Rowland, ed. Human Motor Neuron Diseases. New York: Raven Press 1982:363–93.
3. Kurland LT, Mulder DW. Epidemiologic investigations of amyotrophic lateral sclerosis. 1. Preliminary report on geographic distribution and special reference to the Mariana Islands, including clinical and pathologic observations. Neurology 1954;**4**:438–48.
4. Hirano A, Kurland LT, Krooth RS, et al. Parkinsonism-dementia complex, an endemic disease on the island of Guam. I. Clinical features. Brain 1961;**84**:642–61.
5. Hirano A, Malamud N, Kurland LT. Parkinsonism-dementia complex, an endemic disease on the island of Guam. II. Pathological features. Brain 1961;**84**:662–79.
6. Kimura K, Yase Y, Higashi Y, et al. Epidemiological and geomedical studies on ALS and allied diseases in Kii peninsula (Japan): preliminary report. Proc Jpn Acad

For peer review only - http://bmjopen.bmj.com/site/about/guidelines.xhtml

1961;**37**:417–20.

7. Shiraki H, Yase Y. Amyotrophic lateral sclerosis in Japan. In: Vinken PJ, Bruyn GW, eds. Handbook of clinical neurology Vol. 22, System disorders and atrophies, Part II. Amsterdam: Elsevier Science 1975:353–419.

8. Garruto RM, Yanagihara R, Gajdusek DC. Disappearance of high incidence ALS and PDC on Guam. Neurology 1985;**35**:193–8.

9. Yoshida S. ALS tahatsuchi niokeru kankyoyoin to aluminium no neuron hensei eno kanyo nitsuite.[Environmental factors in western Pacific foci of ALS and a possible pathogenetic role of aluminum (Al) in motor neuron degeneration] (in Japanese with English abstract). Rinsho Shinkeigaku [Clin Neurol] 1991;**31**:1310–2.

10. Spencer P, Palmer VS, Ludolph AC. On the decline and etiology of high-incidence motor system disease in West Papua (Southwest New Guinea). Mov Disord 2005;**20(**Suppl 12):S119–126.

11. Wada T, Matsubayashi K, Okumiya K, et al. Health status and subjective economic satisfaction in West Papua. Lancet 2002;**360**:951.

12. Okumiya K, Fujisawa M, Ishine M, et al. Nishi New Guinea chiiki (Indonesia/ Papua shu) no shinkeihensei shikkan no jittai. 2001-2002 nen, 2006-2007 nen no fieldwork yori. [Fieldwork survey of neurodegenerative diseases in West New-Guinea in 2001-02 and 2006-07] (in Japanese). Rinsho Shinkeigaku [Clin Neurol] 2007;**47**:977–8.

13. Brooks BR. El Escorial World Federation of Neurology criteria for the diagnosis of amyotrophic lateral sclerosis: subcommittee on Motor Neuron Diseases/Amyotrophic Lateral Sclerosis of the World Federation of Neurology Research Group on Neuromuscular Diseases and the El Escorial “Clinical Limits of

For peer review only - http://bmjopen.bmj.com/site/about/guidelines.xhtml

ALS" Workshop Contributors. J Neurol Sci 1994;**124**:96–107.

14. Hoehn MM, Yahr MD. Parkinsonism: onset, progression and mortality. Neurology. 1967;**17**:427–42.

15. Kontor Statistik Distrik Edera. Laporan Kependudukan, Kabupaten Mappi Distrik Edera 2006 (December).

16. Manning C., Rumiak M. Irian Jaya: Economic change, migrants, and indinenous welfare. In Hill H ed. Unity and diversity: Regional Economic Development in Indonesia since 1970. Oxford University Press, Okford.p77-106, 1991.

17. Timmer J. A brief social and political history of Papua, 1962-2005. In Marshall AJ and Beehler BM ed. The Ecology of Indonesian Papua Part Two. Periplus Editions, Hong Kong. p1098-1124, 2007.

18. Badan Pusat Statistik, Sensus Penduduk 2010. http://sp2010.bps.go.id/index.php

19. United Nations , Department of Economic and Social Affairs, Population Division. World Population Prospects The 2012 Revision *Volume II: Demographic Profiles,* http://esa.un.org/unpd/wpp/index.htm

20. Kontor Statistik Kabupaten Merauke. Laporan Kependudukan, Kabupaten Merauke 1997

21. Armon C. Amyotrophic lateral sclerosis. In: Neuroepidemiology from principles to practice. USA: Oxford University Press 2004:162–87.

22. McGuire V, Longstreth WT Jr, Koepsell TD, et al. Incidence of amyotrophic lateral sclerosis in three counties in western Washington state. Neurology 1996 ;**47**:571–3.

23. Sorenson EJ, Stalker AP, Kurland LT, et al. Amyotrophic lateral sclerosis in Olmsted County, Minnesota, 1925 to 1998. Neurology 2002; **23**:280–2.

24. O'Toole O, Traynor BJ, Brennan P, et al. Epidemiology and clinical features of

For peer review only - http://bmjopen.bmj.com/site/about/guidelines.xhtml

amyotrophic lateral sclerosis in Ireland between 1995 and 2004. J Neurol Neurosurg Psychiatry 2008;**79**:30–2.

25. Chiò A, Mora G, Calvo A, et al. Epidemiology of ALS in Italy: a 10-year prospective population-based study. Neurology 2009;**72**:725–31.
26. Factor-Litvak P, Al-Chalabi A, Ascherio A, et al. Current pathways for epidemiological research in amyotrophic lateral sclerosis. Amyotroph Lateral Scler Frontotemporal Degener 2013;**14**(Suppl 1):33–43.
27. Rodgers-Johnson P, Garruto RM, Yanigahara R, et al. Amyotrophic lateral sclerosis and parkinsonism-dementia on Guam: a 30-year evaluation of clinical and neurological trends. *Neurology.* 1986;36:7-13.
28. Kuzuhara S, Kokubo Y, Sasaki R, et al. Familial amyotrophic lateral sclerosis and parkinsonism-dementia complex of the Kii peninsula of Japan: clinical and neuropathological study and tau analysis. Ann Neurol 2001;**49**:501–11.
29. Kuzuhara S, Kokubo Y. Atypical parkinsonism of Japan: amyotrophic lateral sclerosis-parkinsonism-dementia complex of the Kii peninsula of Japan (Muro disease): an update. Mov Disord 2005;**20**(Suppl 12):S108–13.
30. Kuzuhara S. Kii ALS/PDC (Murou byou) no rekishi, gainen, ekigaku. [ALS-parkinsonism-dementia complex of the Kii peninsula of Japan (Muro disease). Historical review, epidemiology and concept] (in Japanese with English abstract). Rinsho Shinkeigaku [Clin Neurol] 2007;**47**:962–5.
31. Kokubo Y. Kiihanto no kinishukuseisokusakukokasho/ parkinson chihou hukugo no rinshou, shinkei byourigakuteki kenkyu. [Clinical aspects, imaging and neuropathology of Kii ALS/PDC] (in Japanese with English abstract). Rinsho Shinkeigaku [Clin Neurol] 2007;**47**:966–9.

For peer review only - http://bmjopen.bmj.com/site/about/guidelines.xhtml

32. Kuzuhara S. Kii hanto no Murobyo (Kinishukuseisokusakukokasho/ parkinson/ ninchisho hukugo) [Muro disease or ALS–parkinsonism–dementia complex of the Kii peninsula of Japan] (in Japanese with English abstract). Rinsho Shinkeigaku [Clin Neurol] 2007;**47**:695–702.

33. Kuzuhara S, KokuboY. Amyotrophic lateral sclerosis-parkinsonism-dementia complex in the Kii peninsula of Japan (Muro disease): a review on recent research and new concept. In: Michael J. Strong, ed. Amyotrophic lateral sclerosis and the frontotemporal dementias. Oxford: Oxford University Press 2012:39–54.

34. Yase Y. The pathogenesis of amyotrophic lateral sclerosis. Lancet 1972;**2**(7772):292–6.

35. Kihara T, Yoshida S, Kondo T et al. An increase in ALS incidence on the Kii Peninsula, 1960-2009: A possible link to change in drinking water source. *Amyotrophic Lateral Sclerosis,* 2012; 13: 347–50.

36. Spencer PS, Nunn PB, Hugon J, et al. Guam amyotrophic lateral sclerosis-parkinsonism-dementia linked to a plant excitant neurotoxin. Science 1987;**237**(4814):517–22.

37. Borenstein AR, Mortimer JA, Schofield E. et al. Cycad exposure and risk of dementia, MCI, and PDC in the Chamorro population of Guam. Neurology. 2007;68:1764-71.

38. Spencer PS, Ohta M, Palmer VS. Cycad use and motor neuron disease in Kii peninsula of Japan. Lancet 1987;**2**(8573):1462–3.

39. Iwami O, Niki Y, Watanabe T, and Ikeda M. Motor neuron disease on the Kii Peninsula of Japan: Cycad exposure. Neuroepidemiology 1993;12:307-312.

40. Ono S, Waring SC, Kurland L et al. Guamanian neurodegenerative disease: ultrastructural studies of skin J. Neurol. Sci., 146 (1997), pp. 35–40

For peer review only - http://bmjopen.bmj.com/site/about/guidelines.xhtml

41. Kokubo Y, Ito K, Kuzuhara S. Ophthalmomyiasis-like pigmentary retinopathy in ALS/PDC in the Kii peninsula of Japan. Neurology. 2003 May 27;60(10):1725-6.

42. Tomiyama H, Kokubo Y, Sasaki R, et al. Mutation analyses in amyotrophic lateral sclerosis/parkinsonism-dementia complex of the Kii peninsula, Japan. Mov Disord 2008;**23**(16):2344–8.

43. Hermosura MC, Nayakanti H, Dorovkov MV, et al. A TRPM7 variant shows altered sensitivity to magnesium that may contribute to the pathogenesis of two Guamanian neurodegenerative disorders. Proc Natl Acad Sci 2005;**102**(32):11510–5.

44. Hara K, Kokubo Y, Ishiura H, et al. TRPM7 is not associated with amyotrophic lateral sclerosis-parkinsonism dementia complex in the Kii peninsula of Japan. Am J Med Genet B Neuropsychiatr Genet 2010;**153B**(1):310–3.

45. Ishiura H, Takahashi Y, Mitsui J, et al. *C9ORF72* Repeat expansion in amyotrophic lateral sclerosis in the Kii peninsula of Japan. Arch Neurol 2012;**69**(9):1154–8.

46. Garruto RM, Yanagihara R. Contributions of isolated Pacific populations to understanding neurodegenerative diseases. Folia Neuropathol 2009;**47**(2):149–70.

47. Plato CC, Garruto RM, Galasko D, et al. Amyotrophic lateral sclerosis and parkinsonism-dementia complex of Guam: changing incidence rates during the past 60 years. Am J Epidemiol 2003;**157**:149–57.

48. Galasko D, Salmon D, Gamst A, et al. Prevalence of dementia in Chamorros on Guam: relationship to age, gender, education, and APOE. Neurology 2007;**68**:1772–81.

49. Plato CC, Galasko D, Garruto RM, et al. ALS and PDC of Guam: forty-year follow-up. Neurology 2002;**58**:765–73.

For peer review only - http://bmjopen.bmj.com/site/about/guidelines.xhtml

Figure and table legends

Table 1. Relevant characteristics and neurological signs of cases with ALS with/without cognitive impairment in Papua, 2001–2012.

Abbreviations: ALS = amyotrophic lateral sclerosis; BK = bradykinesia; BS = Babinski sign; DTR = deep tendon reflex; Dys = dysphagia/dysarthria; F= female; Fasc = fasciculation; LMN = lower motor neuron signs; M = male; MA = muscle atrophy with weakness; NC = not confirmed by examination; P = parkinsonism; PI = postural impairment; R = rigidity; T = tremor; TA = tongue atrophy; U/L: upper limbs/lower limbs; UMN = upper motor neuron signs.

Age: at the time of final survey; Duration: between subjective onset of illness and last survey or death; Alive: alive in 2012; Onset: subjective onset of the illness; Deep tendon reflexes, -: absent, 1: hypoactive, 2: normal, 3: hyperactive, 4: markedly hyperactive with ankle clonus.

Other signs are classified as: -: absent; ±: questionably present; +: present; ++: markedly present.

Family history 1: cousin (possible ALS); 2: grandfather (NC); 3: sister (ALS15); 4: cousin's daughter (P16); 5: cousin (NC); 6: father (NC); 7: sister (ALS 7); 8: sister (P 6) and cousin (NC); 9: father (NC); 10: son: (NC); 11: sister (ALS-P 1) and cousin (NC); 12: mother and father (NC); 13: grandmother (NC); 14: mother (NC); 15: ALS 10 (mother’s cousin).

For peer review only - http://bmjopen.bmj.com/site/about/guidelines.xhtml

Table 2. Relevant characteristics and neurological signs in cases with overlap of ALS and parkinsonism with/without cognitive impairment in Papua, 2001–2012.

Table 3. Relevant characteristics and neurological signs in cases with parkinsonism with/without cognitive impairment in Papua, 2001–2012.

Table 4. Cases of ALS and parkinsonism according to field site

*; Active cases in 2007 in Bade

#; Active cases in 2010 in the villages along the Ia, Edera, and Dumut rivers

Figure 1. Map of the field sites

A. Large-scale map of the southern coastal plain in Papua, Indonesia.

B. Smaller scale map of the southern coastal plain in Papua, Indonesia showing rivers.

C. Detailed map showing villages in the southern coastal area in Papua in which all confirmed cases of ALS and/or parkinsonism with/without cognitive impairment were seen from 2001 to 2012.

Figure 2. Photographs of patient with ALS (Case ALS 12)

A: The patient needs assistance from family members to stand.

B: Advanced atrophy of the tongue.

C: There is upper limb-girdle and truncal muscle atrophy with a positive Babinski sign.

D: Advanced thenar muscle atrophy.

For peer review only

For peer review only - http://bmjopen.bmj.com/site/about/guidelines.xhtml

Figure 3. Photographs of patients with ALS, ALS-parkinsonism, and parkinsonism

A: Patient with ALS (Case ALS-17) showing muscle atrophy in the distal upper and lower limbs.

B: Patient with ALS-parkinsonism (Case ALS-P 1) showing muscle atrophy in distal upper limbs.

C: Patient with ALS-parkinsonism (Case ALS-P 7) showing atrophy in muscles in the proximal upper limb, shoulder, and tibialis anterior.

D: Patient with parkinsonism (Case P 7) showing typical parkinsonian hands and anterior bending posture.

Parkinsonian hand: Striatal deformities of the hand with abnormal postures that are common in patients with advanced Parkinson's disease.

For peer review only

For peer review only - http://bmjopen.bmj.com/site/about/guidelines.xhtml

Table 1. Relevant characteristics and neurological signs in cases with pure ALS with/without cognitive impairment in Papua, 2001–2012.

Case number	ALS criteria Yahr stage	Survey year	Village	Age	Sex	Onset (age/year)	Duration (years)	Death (year)	Tribe (origin)	UMN signs		LMN signs		Bulbar signs		Parkinsonian sign				Cognitive impairment	Family history
										DTR U/L	BS	MA U/L	Fasc U/L	TA	Dys	T U/L	R U/L	BK	PI		
Pure ALS (n=17)																					
ALS 1	Definite	2001	Bade	35	M	NC	NC	NC	Auyu	3/3	+	++/++	+/+	++	-	-	-	-	-	-	-
ALS 2	Definite	2001	Ogoto	43	F	NC	NC	2002	Auyu	3/3	+	++/++	+/+	++	++	-	-	-	-	-	-
ALS 3	Definite	2002	Senggo	50	M	48/'00	2	NC	Citak	3/3	+	++/++	±/±	±	+	-	-	-	-	-	1
ALS 4	Definite	2007	Homilikia	53	M	51/'05	2	2007	Auyu	3/2	+	++/++	+/+	-	-	-	-	-	-	-	-
ALS 5	Definite	2007/'08	Bade	51	M	49/'06	3	2009	Auyu	3/4	+	+/-	-/-	-	+	-	-	-	-	-	-
ALS 6	Definite	2008	Bade	21	M	11/'98	11	2009	Auyu	4/4	+	++/++	-/-	-	-	-	-	-	-	-	2
ALS 7	Definite	2007/'08/'10	Bade	70	F	57/'97	13	2010	Maluku	3/3	+	++/++	+/-	-	+	-	-	-	-	-	3
ALS 8	Probable	2011	Geturki	53	M	48/'06	5	Alive	Auyu	3/3	-	+/+	-/+	-	-	-	-	-	-	-	-
ALS 9	Definite	2011	Muya	41	F	36/'06	6	2012	Auyu	3/3	+	+/+	-/-	-	-	-	-	-	-	-	-
ALS 10	Probable	2007/'10/'11/'12	Osso	65	F	59/'06	6	Alive	Auyu	3/4	+	+/-	-/-	+	-	-	-	-	-	-	4
ALS 11	Probable	2007/'08/'10/'12	Gimikya	32	M	12/'92	20	Alive	Auyu	4/4	+	++/+	++/-	±	-	-	+/-	-	-	-	5
ALS 12	Definite	2010/'11/'12	Bosma	64	M	53/'01	11	Alive	Auyu	3/4	+	++/++	+/-	++	-	-	-	-	-	-	6
ALS 13	Definite	2012	Asset	70	F	68/'10	2	Alive	Auyu	3/3	-	+/+	-/-	-	-	-	-	-	-	-	-
ALS 14	Probable	2012	Pies	65	F	60/'07	5	Alive	Auyu	3/4	-	+/+	-/-	-	-	-	-	-	-	-	-
ALS 15	Definite	2007	Bade	64	F	62/'05	2	2007	Maluku	3/3	+	++/++	-/-	-	+	-	-	-	-	+	7
ALS 16	Definite	2008/'10/'11	Yibin	25	M	13/'99	13	2012	Auyu	3/4	+	++/+	++/++	-	-	-	-	-	-	+	-
ALS 17	Definite	2012	Ogorito	70	M	66/'08	4	Alive	Auyu	3/3	+	+/+	+/+	-	-	-	-	-	-	+	-

Abbreviations: ALS = amyotrophic lateral sclerosis; BK = bradykinesia; BS = Babinski sign; DTR = deep tendon reflex; Dys = dysphagia/dysarthria; F = female; Fasc = fasciculation; LMN = lower motor neuron signs; M = male; MA = muscle atrophy with weakness; NC = not confirmed by examination; P = parkinsonism; PI = postural impairment; R = rigidity; T = tremor; TA = tongue atrophy; U/L: upper limbs/lower limbs; UMN = upper motor neuron signs.

Age: at the time of final survey; Duration: between subjective onset of illness and last survey or death; Alive: alive in 2012; Onset: subjective onset of illness; Deep tendon reflexes, -: absent, 1: hypoactive, 2: normal, 3: hyperactive, 4: markedly hyperactive with ankle clonus.

Other signs are classified as: -: absent; ±: questionably present; +: present; ++: markedly present.

Family history 1: cousin (possible ALS); 2: grandfather (NC); 3: sister (ALS15); 4: cousin's daughter (P16); 5: cousin (NC); 6: father (NC); 7: sister (ALS 7); 8: sister (P 6) and cousin (NC); 9: father (NC); 10: son: (NC); 11: sister (ALS-P 1) and cousin (NC); 12: mother and father (NC); 13: grandmother (NC); 14: mother (NC); 15: ALS 10 (mother’s cousin).

For peer review only - http://bmjopen.bmj.com/site/about/guidelines.xhtml

Okumiya

Table 2. Relevant characteristics and neurological signs in cases with overlap of ALS and parkinsonism with/without cognitive impairment in Papua, 2001–2012.

Case number	ALS criteria Yahr stage	Survey year	Village	Age	Sex	Onset (age/year)	Duration (year)	Death (year)	Tribe (origin)	UMN signs DTR U/L	UMN signs BS	LMN signs MA U/L	LMN signs Fasc U/L	Bulbar signs TA	Bulbar signs Dys	Parkinsonian sign T U/L	Parkinsonian sign R U/L	Parkinsonian sign BK	Parkinsonian sign PI	Cognitive impairment	Family history
ALS-parkinsonism (n = 13)																					
ALS-P 1	Possible-IV	2008	Pies	65	F	60/'03	6	2009	Auyu	3/3	+	+/-	-/-	-	-	+/+	++/++	++	+	-	8
ALS-P 2	Possible-III	2010/'11	Ogorito	76	M	70/'05	6	2011	Auyu	3/3	+	+/±	-/-	-	-	+/+	+/+	+	+	-	-
ALS-P 3	Possible-III	2010/'11/'12	Homilikia	69	M	65/'08	4	Alive	Auyu	3/2	+	+/-	-/-	-	-	+/+	+/+	+	+	-	-
ALS-P 4	Definite-III	2008	Bade	34	M	18/'92	17	2009	Muyu	3/3	+	+/-	-/-	++	++	-	+/+	+	+	+	9
ALS-P 5	Definite-III	2012	Sien	60	M	58/'10	2	Alive	Auyu	3/3	+	+/+	-/-	-	-	-	+/+	+	+	+	-
ALS-P 6	Definite-III	2012	Benggo	50	M	48/'10	2	Alive	Auyu	2/2	+	+/+	-/-	+	+	-	+/-	+	+	+	-
ALS-P 7	Possible-II	2001	Bade	36	M	NC	NC	NC	Auyu	3/3	+	-/-	-/-	-	-	+	+	+	-	-	-
ALS-P 8	Possible-II	2008/'10	Bade	23	M	11/'98	12	Alive	Auyu	3/4	+	-/-	-/-	-	-	+/+	+/±	+	-	-	-
ALS-P 9	Possible-III	2010/'11	Ogorito	76	M	70/'05	6	2011	Auyu	3/3	+	-/-	-/-	-	-	+/-	+/+	+	+	-	-
ALS-P 10	Possible-III	2008/'10/'11	Yibin	52	M	44/'03	8	Alive	Auyu	3/3	+	-/-	-/-	-	-	+/-	+/+	+	+	-	-
ALS-P 11	Possible-III	2011	Getio	55	F	53/09	2	2011	Auyu	3/3	+	±/-	-/-	-	-	+/+	+/+	+	+	-	-
ALS-P 12	Possible-IV	2001	Bade	40	M	NC	NC	NC	Auyu	3/3	+	-/-	-/-	-	+	+	+	+	+	+	-
ALS-P 13	Possible-III	2012	Bosma	52	M	48/'08	4	Alive	Auyu	3/4	+	-/-	-/-	-	-	-	++/+	+	+	+	-

Abbreviations: ALS = amyotrophic lateral sclerosis; BK = bradykinesia; BS = Babinski sign; DTR = deep tendon reflex; Dys = dysphagia/dysarthria; F = female; Fasc = fasciculation; LMN = lower motor neuron signs; M = male; MA = muscle atrophy with weakness; NC = not confirmed by examination; P = parkinsonism; PI = postural impairment; R = rigidity; T = tremor; TA = tongue atrophy; U/L: upper limbs/lower limbs; UMN = upper motor neuron signs.

Age: at the time of final survey; Duration: between subjective onset of illness and last survey or death; Alive: alive in 2012; Onset: subjective onset of illness; Deep tendon reflexes, -: absent, 1: hypoactive, 2: normal, 3: hyperactive, 4: markedly hyperactive with ankle clonus.

Other signs are classified as: -: absent; ±: questionably present; +: present; ++: markedly present.

Family history 1: cousin (possible ALS); 2: grandfather (NC); 3: sister (ALS15); 4: cousin's daughter (P16); 5: cousin (NC); 6: father (NC); 7: sister (ALS 7); 8: sister (P 6) and cousin (NC); 9: father (NC); 10: son: (NC); 11: sister (ALS-P 1) and cousin (NC); 12: mother and father (NC); 13: grandmother (NC); 14: mother (NC); 15: ALS 10 (mother's cousin).

For peer review only

Okumiya

Table 3. Relevant characteristics and neurological signs in cases with parkinsonism with/without cognitive impairment in Papua, 2001–2012.

Case number	ALS criteria Yahr stage	Survey year	Village	Age	Sex	Onset (age/year)	Duration (years.)	Death (year)	Ethnicity	UMN signs DTR U/L	UMN signs BS	LMN signs MA U/L	LMN signs Fasc U/L	Bulbar signs TA	Bulbar signs Dys	Parkinsonian sign T U/L	Parkinsonian sign R U/L	Parkinsonian sign BK	Parkinsonian sign PI	Cognitive impairment	Family history
Parkinsonism (n = 16)																					
P 1	Yahr IV	2001	Kepi	50	M	NC	NC	NC	Jakai	2/2	-	-/-	-/-	-	-	+/+	+/+	++	+	-	-
P 2	Yahr II	2001	Kepi	50	M	NC	NC	NC	Jakai	2/2	-	-/-	-/-	-	-	+	+	+	-	-	-
P 3	Yahr IV	2002	Emete	52	M	49/'99	3	NC	Jakai	2/2	-	-/-	-/-	-	-	+	+	+	+	-	-
P 4	Yahr II	2002	Senggo	41	M	36/'97	5	NC	Citak	2/2	-	-/-	-/-	-	-	+	+	-	-	-	-
P 5	Yahr I	2007	Mur	65	F	61/'03	4	NC	Jakai	2/3	-	-/-	-/-	-	-	+/-	+/-	-	-	-	10
P 6	Yahr IV	2008	Pies	60	F	55/'03	6	2009	Auyu	2/2	-	-/-	-/-	-	-	+/+	+/+	++	+	-	11
P 7	Yahr IV	2008/'10	Mopio	52	F	47/'05	5	Alive	Auyu	3/3	-	-/-	-/-	-	-	++/+	+/+	++	+	-	-
P 8	Yahr II	2011	Sien	57	F	49/03	9	2012	Auyu	3/3	-	-/-	-/-	-	-	+/+	+/+	+	-	-	12
P 9	Yahr IV	2007/'08/'10/'[illegible]	Bade	46	F	40/'06	6	Alive	Auyu	3/3	-	-/-	-/-	-	-	+/+	++/+	+	+	-	13
P 10	Yahr II	2011/'12	Asset	38	M	27/'01	11	Alive	Auyu	3/3	-	-/-	-/-	-	-	+/-	+/-	+	-	-	-
P 11	Yahr III	2011/'12	Sien	54	F	40/'98	14	Alive	Auyu	3/3	-	-/-	-/-	-	-	++/+	++/+	+	+	-	-
P 12	Yahr II	2011/'12	Memes	66	F	64/10	2	Alive	Auyu	3/3	-	-/-	-/-	-	-	+/+	+/+	+	-	-	14
P 13	Yahr II	2012	Harapan	60	M	57/09	3	Alive	Auyu	2/2	-	-/-	-/-	-	-	-	+/+	-	-	-	-
P 14	Yahr II	2007	Mur	50	M	38/'95	12	NC	Auyu	2/4	+	-/-	-/-	-	-	+	+	+	-	-	-
P 15	Yahr I	2007	Homilikia	50	M	NC	NC	Alive	Auyu	2/3	+	-/-	-/-	-	-	+/-	+/±	-	-	-	-
P 16	Yahr III	2011	Osso	35	F	33/'09	2	2011	Auyu	3/2	-	-/-	-/-	-	-	+/+	+/+	+	+	+	15

Abbreviations: ALS = amyotrophic lateral sclerosis; BK = bradykinesia; BS = Babinski sign; DTR = deep tendon reflex; Dys = dysphagia/dysarthria; F = female; Fasc = fasciculation; LMN = lower motor neuron signs; M = male; MA = muscle atrophy with weakness; NC = not confirmed by examination; P = parkinsonism; PI = postural impairment; R = rigidity; T = tremor; TA = tongue atrophy; U/L: upper limbs/lower limbs; UMN = upper motor neuron signs.

Age: at the time of final survey; Duration: between subjective onset of illness and last survey or death; Alive: alive in 2012; Onset: subjective onset of illness; Deep tendon reflexes, -: absent, 1: hypoactive, 2: normal, 3: hyperactive, 4: markedly hyperactive with ankle clonus.

Other signs are classified as: -: absent; ±: questionably present; +: present; ++: markedly present.

Family history 1: cousin (possible ALS); 2: grandfather (NC); 3: sister (ALS15); 4: cousin's daughter (P16); 5: cousin (NC); 6: father (NC); 7: sister (ALS 7); 8: sister (P 6) and cousin (NC); 9: father (NC); 10: son: (NC); 11: sister (ALS-P 1) and cousin (NC); 12: mother and father (NC); 13: grandmother (NC); 14: mother (NC); 15: ALS 10 (mother’s cousin).

For peer review only - http://bmjopen.bmj.com/site/about/guidelines.xhtml

For peer review only

Table 4. Cases of ALS and parkinsonism in the field sites

	ALS criteria	Case No.	Crude point prevalence rate (95%CI) among ALS/ ALS-P/ Parkinsonism (2007* or 2010#)
Bade 2007–2012 (population 3800)			
ALS	Definite	ALS 5*	105 (2-208) /100,000 in 2007*
(n=4)	Definite	ALS 6*	
	Definite	ALS 7*	
	Definite	ALS 15*	
ALS-parkinsonism	Definite	ALS-P 4*	53 (0-126) /100,000 in 2007*
(n=2)	Possible	ALS-P 8*	
Parkinsonism (n=1)		P 9*	26 (0-78) /100,000 in 2007*
Ia river 2007–2012 (population 4500)			
ALS	Definite	ALS 4	133(27-240) /100,000 in 2010#
(n=7)	Probable	ALS 8#	
	Probable	ALS 10#	
	Probable	ALS 11#	
	Definite	ALS 12#	
	Definite	ALS 13#	
	Definite	ALS 17#	
ALS-parkinsonism	Possible	ALS-P 2#	89 (2-176) /100,000 in 2010#
(n=4)	Possible	ALS-P 3#	
	Possible	ALS-P 9#	
	Possible	ALS-P 13#	
Parkinsonism		P 10#	67 (0-142) /100,000 in 2010#
(n=3)		P 15#	
		P 16#	
Edera and Dumut rivers 2008–2012 (population: 4100)			
ALS	Definite	ALS 9#	73 (0-156) /100,000 in 2010#
(n=3)	Probable	ALS 14#	
	Definite	ALS 16#	
ALS-parkinsonism	Possible	ALS-P 1	98 (2-193) /100,000 in 2010#
(n=5)	Definite	ALS-P 5#	
	Definite	ALS-P 6#	
	Possible	ALS-P 10#	
	Possible	ALS-P 11#	
Parkinsonism		P 6	122 (15-229) /100,000 in 2010
(n=6)		P 7#	
		P 8#	
		P 11#	
		P 12#	
		P 13#	

*:Active cases in 2007 in Bade
#:Active cases in 2010 in the villages along Ia, Edera and Dumut rivers
95%CI: 95% confidence interval

For peer review only - http://bmjopen.bmj.com/site/about/guidelines.xhtml

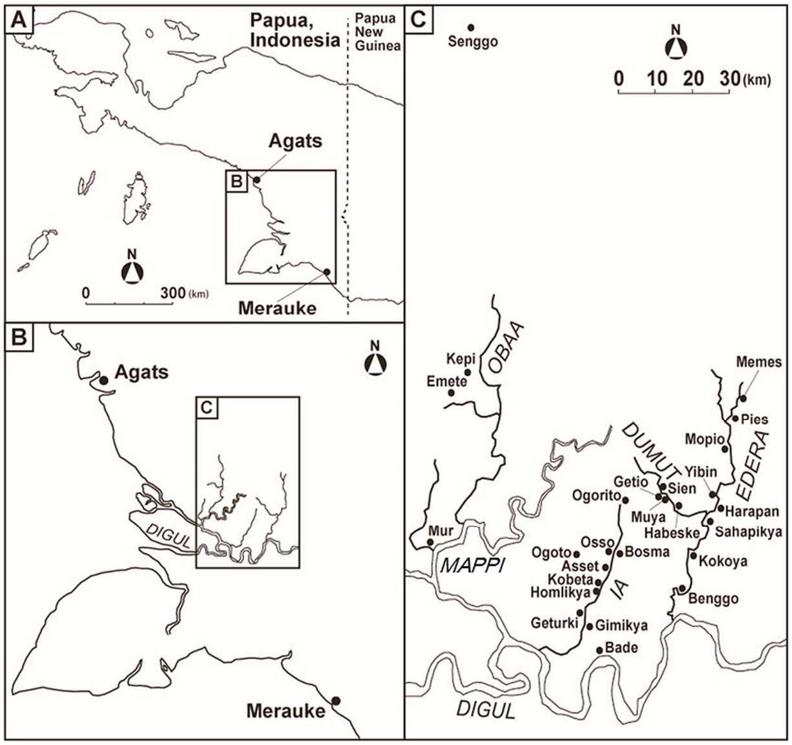

119x90mm (300 x 300 DPI)

For peer review only

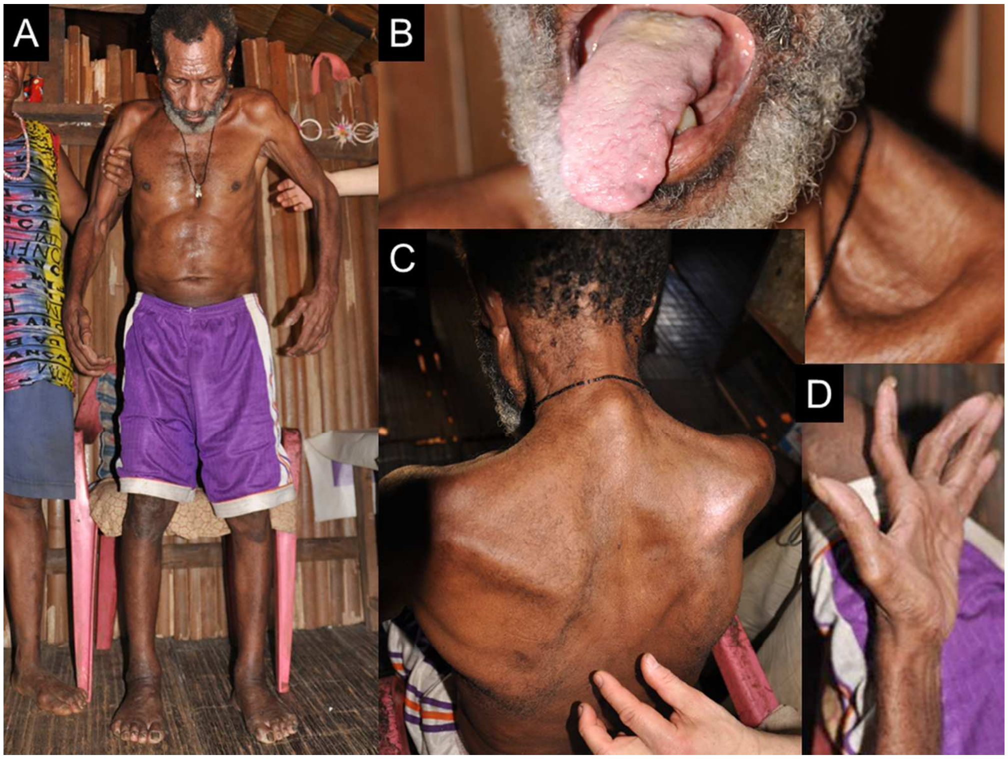

119x90mm (300 x 300 DPI)

For peer review only

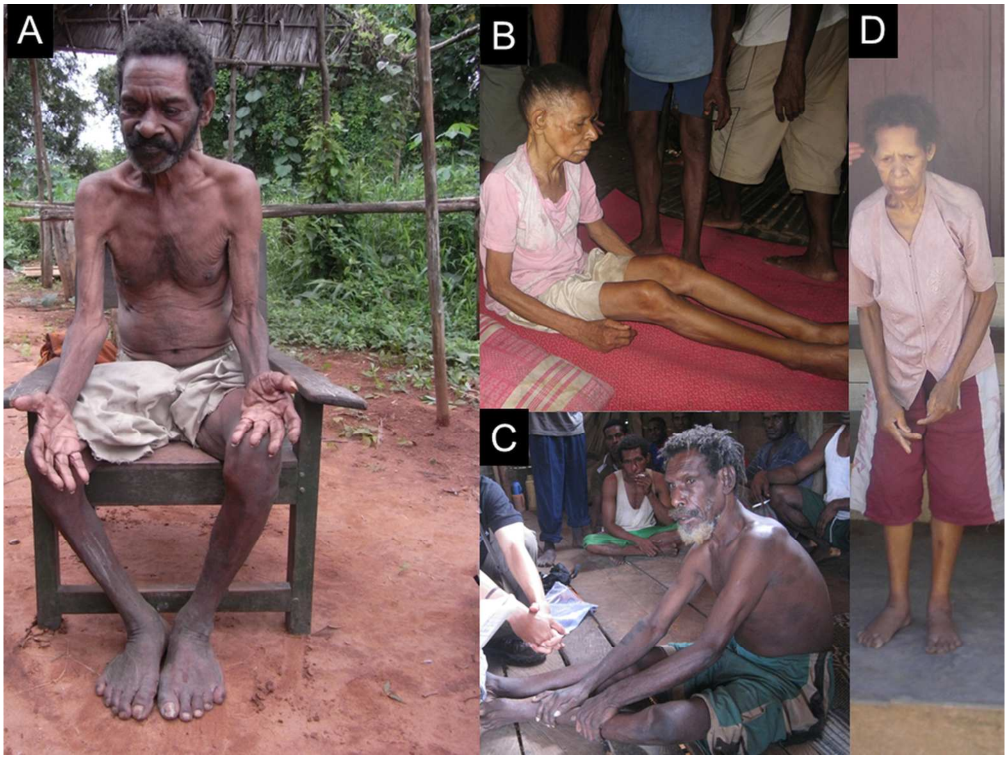

119x90mm (300 x 300 DPI)

For peer review only

Amyotrophic lateral sclerosis and parkinsonism in Papua, Indonesia: 2001–2012 survey results

Journal:	*BMJ Open*
Manuscript ID:	bmjopen-2013-004353.R3
Article Type:	Research
Date Submitted by the Author:	07-Mar-2014

Complete List of Authors:	Okumiya, Kiyohito; Research Institute for Humanity and Nature,<br>Wada, Taizo; Kyoto University, Center for Southeast Asian Studies<br>Fujisawa, Michiko; Center for Southeast Asian Studies,<br>Ishine, Masayuki; Yasugi Clinic,<br>Garcia del Saz, Eva; Kochi University, Center for Regional & International Collaboration<br>Hirata, Yutaka; Kitaakita Municipal Hospital,<br>Kuzuhara, Shigeki; Suzuka University of Medical Science, Faculty of Health Science<br>Kokubo, Yasumasa; Mie University, Graduate School of Regional Innovation Studies<br>Seguchi, Harumichi; Kochi University, Faculty of Medicine<br>Sakamoto, Ryota; Kyoto University, Hakubi Center for Advanced Research<br>Manuaba, Indrajaya; Cenderawasih University, Faculty of Medicine<br>Watofa, Paulina; Cenderawasih University, Faculty of Medicine<br>Rantetampang, Andreas; Cenderawasih University, Faculty of Public Health<br>Matsubayashi, Kozo; Kyoto University, Center for Southeast Asian Studies

**Primary Subject Heading**:	Epidemiology
Secondary Subject Heading:	Neurology
Keywords:	Motor neurone disease < NEUROLOGY, Parkinson-s disease < NEUROLOGY, Dementia < NEUROLOGY

SCHOLARONE™ Manuscripts

Amyotrophic lateral sclerosis and parkinsonism in Papua, Indonesia: 2001–2012 survey results

Kiyohito Okumiya[1,2], Taizo Wada[2], Michiko Fujisawa[2], Masayuki Ishine[3], Eva Garcia del Saz[4], Yutaka Hirata[5], Shigeki Kuzuhara[6], Yasumasa Kokubo[7], Harumichi Seguchi[8], Ryota Sakamoto[9], Indrajaya Manuaba[10], Paulina Watofa[10], Andreas L. Rantetampang[11], Kozo Matsubayashi[2]

[1]Research Institute for Humanity and Nature, Kyoto, Japan
[2]Center for Southeast Asian Studies, Kyoto University, Kyoto, Japan
[3]Yasugi Clinic, Shimane, Japan
[4]Center for Regional & International Collaboration, Kochi University, Kochi, Japan
[5]Kitaakita Municipal Hospital, Akita, Japan
[6]Faculty of Health Science, Suzuka University of Medical Science, Mie, Japan
[7]Graduate School of Regional Innovation Studies, Mie University, Mie, Japan
[8]Faculty of Medicine, Kochi University, Kochi, Japan
[9]Hakubi Center for Advanced Research, Kyoto University, Kyoto, Japan
[10]Faculty of Medicine, Cenderawasih University, Papua, Indonesia
[11]Faculty of Public Health, Cenderawasih University, Papua, Indonesia

Correspondence to: Kiyohito Okumiya,
Research Institute for Humanity and Nature
Motoyama 457-4, Kamigamo, Kita-ku, Kyoto 603-8047, Japan
Tel: +81-75-707-2411, Fax: +81-75-707-2509
E-mail: okumiyak@chikyu.ac.jp
Center for Southeast Asian Studies
Kyoto University
46, Shimoadachi-cho, Yoshida , Sakyo-ku, 606-9501, Japan
Tel: +81-75-753-7320
E-mail: okumiya@cseas.kyoto-u.ac.jp

Abstract:296 words
Text:5315 words
4 tables and 3 figures

For peer review only

ABSTRACT

Objective: Only one prior follow-up study of amyotrophic lateral sclerosis (ALS) and parkinsonism in Papua, Indonesia has been carried out since a survey undertaken from 1962 to 1981 by Gajdusek and colleagues. Therefore, to clarify the clinical epidemiology of ALS and parkinsonism in the south coastal region of Papua, the clinical characteristics and prevalence of the diseases in this region were examined and assessed.

Methods: Cases of ALS and parkinsonism were clinically examined during a 2001–2012 survey in Bade and other villages along the Ia, Edera, Dumut, and Obaa rivers in Papua, Indonesia. Possible, probable, and definite ALS were diagnosed clinically by certified neurologists on the basis of El Escorial Criteria. The criteria for diagnosis of parkinsonism were the presence of at least 2 of the 4 following signs: tremor, rigidity, bradykinesia, and postural impairment with a progressive course.

Results: During the survey, 46 cases of ALS and/or parkinsonism were diagnosed within a population range of 7000 (2001–2002) to 13,900 (2007–2012). The 46 cases consisted of 17 probable-definite cases of ALS, including 3 of cognitive impairment (CI); 13 cases of overlap of possible, probable, or definite ALS and parkinsonism, including 5 cases of CI; and 16 cases of parkinsonism, including 1 case of CI. The crude point prevalence rate of pure ALS was estimated as at least 73 (0–156) to 133 (27–240)/100,000 people and that of the overlap of ALS and parkinsonism was at least 53 (0–126) to 98 (2–193)/100,000 in 2007 or 2010 in some regions.

Conclusions: While the prevalence of ALS has decreased over the past ~30-35 years, it remains higher than the global average. There was a high prevalence of overlap of ALS, parkinsonism, and CI, which has been previously reported in Guam and Kii.

For peer review only - http://bmjopen.bmj.com/site/about/guidelines.xhtml

Strengths and limitations of this study

・This study is unique in the epidemiological survey of neurodegenerative diseases during the period between 2001 and 2012 in Papua, Indonesia, one of the foci with the highest incidence of ALS in the world.

・This study recognized the clinical findings of significant overlap of ALS with parkinsonism and cognitive impairment.

・ This study was based only on clinical findings and there are the limitations of not having electromyogram data, DNA analysis, or any autopsy data from this population. Without such data, it is difficult to be certain how these patients fit into sporadic ALS or Parkinson's disease and how they compare with ALS/PDC on Guam and in the Kii peninsula.

For peer review only

For peer review only - http://bmjopen.bmj.com/site/about/guidelines.xhtml

INTRODUCTION

Between 1962 and 1980, Gajdusek et al. reported 97 cases of amyotrophic lateral sclerosis (ALS), 18 cases of parkinsonism, and 18 cases of poliomyeloradiculitis (PMR) in the south coastal area of Papua (former Irian Jaya, Indonesia), which had a population of 7,000. The Ia River is in the epicenter of the high-incidence focus of ALS and parkinsonism in Papua.[1,2] Along with Guam and Kii in Japan, Papua (Indonesia) has been considered one of the foci with the highest incidence of ALS in the world.[1-7] The high incidence of ALS and parkinsonism-dementia complex (PDC) in Guam and Kii reportedly no longer exists.[8,9] In 1987, Spencer et al. reported only 2 cases of ALS and three cases of parkinsonism in the villages along the Ia River. In 1990, no cases of ALS and three cases of parkinsonism were reported along the same river; thus, decline or disappearance of ALS was suspected to have also occurred in Papua.[10] No follow-up surveys on ALS and parkinsonism have been conducted in Papua since 1991.

To clarify the clinical epidemiological characteristics of ALS and parkinsonism in the south coastal region of Papua, we conducted a survey of neurodegenerative diseases documented between 2001 and 2012.

METHODS

The survey sites for between 2001 and 2002 (total population, 7000) were the villages of Kepi (population, 800), Emete (800), Ogoto (400), Senggo (2000), and Bade (3000).[11,12] For between 2007 and 2012, the survey sites were the villages along 4 rivers (total population, 13,900), namely Bade along the Digul River (population, 3800

For peer review only - http://bmjopen.bmj.com/site/about/guidelines.xhtml

people), Mur along the Mappi River (1500), the 8 villages along the Ia River (4500), and the 12 villages along the Edera and Dumut rivers (4100 people). The 8 villages along the Ia River were Gimikya (800), Geturki (500), Homlikya (700), Kobeta (600), Asset (600), Osso (300), Bosma (500), and Ogorito (500). The 12 villages along the Edera and Dumut rivers were Benggo (200), Kokoya (500), Sahapikya (400), Harapan (400), Yibin (300), Mopio (300), Pies (300), Memes (300), Habeske (300), Muya (300), Getio (200), and Sien (600; Figure 1). At least 2 of 6 Japanese-certified neurologists (KO, MF, YH, SK, YK, and KM) visited each village with a local neurologist (IM).

The village leaders and medical staff members of the primary healthcare centre in each village agreed to take part in the survey, which was conducted with the permission of the Department of Health of the Papua Provincial Government in cooperation with Cenderawasih University. The purpose of the neurological examinations was explained to all of the villagers. All the subjects volunteered to participate after the announcement of availability of neurological check-ups, which were performed in the community health centres or homes in each field site. While this was not a complete inventory survey, we asked each medical doctor or health staff, and village leader to call all patients with neurological signs or symptoms, including muscle weakness, gait disturbance, tremor, bradykinesia, or cognitive impairment (CI), in each village. The patients who were called also volunteered to participate. Indonesian collaborators and the co-authors (an English teacher in Bade Senior High School and Indonesian neurologist IM, and staff from Cenderawasih University) who spoke both English and Indonesian; local people who spoke both Indonesian and the local language in each

For peer review only

village; and co-author EG, who spoke English, Indonesian, and Japanese, cooperatively served as translators. Written informed consent was obtained from all the study participants. For the participants who could not read/write, verbal communication was carried out with the support of their family members and the translators. All the participants agreed to undergo neurological examinations, and none of the patients declined annual re-examination. The survey was approved by the ethics committees of the Research Institute for Humanity and Nature in Japan and Cenderawasih University in Papua, Indonesia.

Among the many types of neurological and non-neurological cases encountered, diagnoses of ALS, parkinsonism, and CI were based on clinical examinations and discussions among the participating neurologists. ALS was classified as 'definite', 'probable', 'possible', and 'suspected' on the basis of combinations of upper motor neuron (UMN) and lower motor neuron (LMN) signs according to the El Escorial Criteria of the World Federation of Neurology.[13] Probable and definite ALS are defined as pure ALS in this report. A diagnosis of parkinsonism required the presence of at least 2 of the following 4 signs: tremor, rigidity, bradykinesia, and postural impairment with a progressive course without evidence/history of vascular accidents or history of taking drugs known to induce parkinsonism. The degree of disability of patients with parkinsonism was classified according to the scale of Hoehn and Yahr.[14] Overlap of possible, probable, or definite ALS with parkinsonism was defined as ALS-parkinsonism. CI was diagnosed when loss of memory or impairments of language usage, praxis, or executive functions were identified during interview and clinical examination by neurologists. As cognitive functional test such as the mini–mental state

For peer review only

examination was not performed for all of the participants, patients with complications of functional disabilities attributable to CI in activities of daily living or in their livelihood were screened during the interview of both the patients and their family members. PMR was evaluated using the definition of Gajdusek et al. that PMR is a subacute paralytic condition reminiscent of the Landry-Guillain-Barre syndrome, which combines radicular, or perhaps neuritic elements with more acute onset and sometimes asymmetric paralysis.[1,2] Neurological cases with overlapping cerebellar signs (2 cases) were excluded from this study.

In Bade and the villages along the Ia, Edera, and Dumut rivers, not only were new cases were identified but also previously diagnosed cases were followed up and assessed neurologically and documented in 2007, 2008, 2010, 2011, and 2012. Death years were confirmed, and the durations between subjective onset of illness and the last survey or death were recorded as durations of subjective illness.

The size of the populations of Bade and the villages along the Ia, Edera, and Dumut rivers were determined from a report by the public office of Edera district in 2006.[15] We were informed by the village leaders of the populations of other villages in the survey for the period 2001–2002.

RESULTS

Clinical types of ALS and/or parkinsonism

We identified 46 cases with signs of ALS and/or parkinsonism, including 17 cases with pure ALS (Table 1), 13 with ALS-parkinsonism (Table 2), and 16 with parkinsonism

For peer review only - http://bmjopen.bmj.com/site/about/guidelines.xhtml

(Table 3). We found no case of pure or dominant dementia and PMR consistent with the definition by Gajdusek et al; similarly, no cases of PMR were found by Spencer et al. in 1987 or 1990. [10] Relevant information about the cases, including neurological signs, age at last survey, age and year of onset, disease duration, certified death year, and ethnicity, is shown in Tables 1–3. The male-to-female sex distribution was 10:7 (male, 59%) for ALS, 11:2 (85%) for ALS-parkinsonism, and 8:8 (50%) for parkinsonism. The mean age at last survey was 51.3 years for ALS, 52.9 years for ALS-parkinsonism, and 51.6 years for parkinsonism. The mean age at onset was 46.2 years (range, 11–68 years) for pure ALS, 49.5 years (11–70 years) for ALS-parkinsonism, and 45.8 years (27–64 years) for parkinsonism. The mean duration of subjective illness was 7.0 years (2–20 years) for pure ALS, 6.3 years (2–17 years) for ALS-parkinsonism, and 6.3 years (2–14 years) for parkinsonism. We observed overlapping CI in three (18%) of the 17 cases of pure ALS, 5 (38%) of the 13 cases of ALS-parkinsonism, and 1 (6%) of the 16 cases of parkinsonism.

During the follow-up surveys of the cases in Bade, Ia, and Edera rivers, we observed the following changes in diagnosis: 1 case, from possible ALS to probable ALS (case ALS10); 2 cases, from parkinsonism to ALS-parkinsonism (cases ALS-P 2 and ALS-P 3); and 1 case, from probable ALS to definite ALS (case ALS16) complicated with CI. We also identified an instance of a shared family history of parkinsonism (P 6) and ALS-parkinsonism (ALS-P 1). There were family histories of neurological disorders in 7 of 17 cases (41%) of pure ALS, 2 (15%) of 13 cases of ALS-parkinsonism, and 6 (38%) of 16 cases of parkinsonism (Tables 1–3).

For peer review only - http://bmjopen.bmj.com/site/about/guidelines.xhtml

Cases of ALS and parkinsonism in Bade and the villages along the Ia, Edera, and Dumut rivers between 2007 and 2012

Between 2007 and 2012, we identified 4 cases of pure ALS, 2 cases of ALS-parkinsonism, and 1 case of parkinsonism in Bade (population, 3800 people). In addition, we found 7 cases of pure ALS, 4 cases of ALS-parkinsonism, and three cases of parkinsonism in the villages (population, 4500 people) along the Ia river. Between 2008 and 2012, we identified three cases of pure ALS, 5 cases of ALS-parkinsonism, and 6 cases of parkinsonism in the villages (population, 4100 people) along the Edera and Dumut rivers (Table 4).

In the 2007 survey, there were 4 active cases of pure ALS (crude point prevalence rate, 105 [2–208]/100,000 people), 2 cases of ALS-parkinsonism (53 [0–126]/100,000 people), and 1 case of parkinsonism (26 [0–78]/100,000 people) in Bade. In 2010, there were 6 active cases of pure ALS (133 [27–240]/100,000 people), 4 cases of ALS-parkinsonism (89 [2–176]/100,000 people), and three cases of parkinsonism (67 [0–142]/100,000 people) in the villages along the Ia River. In 2010, there were three active cases of pure ALS (73 [0–156]/100,000 people), 4 cases of ALS-parkinsonism (98 [2–193]/100,000 people), and 5 cases of parkinsonism (122 [15-229]/100,000 people) in the villages along the Edera and Dumut rivers (Table 4).

In the survey during the period 2007–2012, the number of each clinical type (ALS, ALS-parkinsonism, and parkinsonism) was shown respectively for Bade (4, 4, and 1); the villages along the Ia River, namely Ogorito (1,2, and 0), Bosma (1, 1, and 0), Homlikya (1, 1, and 1), Gimikya (1, 1, and 0), Geturki (1, 0, and 0), Asset (1, 0, and 1),

For peer review only - http://bmjopen.bmj.com/site/about/guidelines.xhtml

and Osso (1, 0, and 1); and the villages along the Edera and Dumut rivers, namely Yibin (1, 1, and 0), Sien (0, 1, and 2), Pies (1, 0, and 1), Benggo (0, 1, and 0), Harapan (0, 0, and 1), Mopio (0, 0, and 1), Memes (0, 0, and 1), Muya (1, 0, and 0), and Getio (0, 1, and 0).

Case reports

Case ALS 12

A 64-year-old (in 2012) man of the Auyu tribe, a resident of Bosma along the Ia River, had a history of progressive gait disturbance and motor weakness for 9 years in 2010; these symptoms were found to have progressed based on the findings from the follow-up check-ups between 2011 and 2012. Neurological examination revealed UMN and LMN signs, including a positive Babinski sign, hyperreflexia with ankle clonus, muscle atrophy, and weakness in the tongue and upper and lower limbs bilaterally, and fasciculation in the upper limbs and tongue. He had no CI or sensory disturbance. We diagnosed him as having definite ALS (Figure 2-A–D). The subject had ALS, and his brother reported that their father showed tremor and gait disturbance, which were interpreted to be consistent with parkinsonism.

Case ALS-CI 17

A 70-year-old (in 2012) man of the Auyu tribe, a resident of Ogorito along the Ia River, had a history of progressive memory loss for 4 years and slow gait for 2 years. Neurological examination revealed UMN and LMN signs, including a positive Babinski sign, hyperreflexia in the jaw jerk, bilateral upper and lower limbs, and muscle atrophy,

For peer review only - http://bmjopen.bmj.com/site/about/guidelines.xhtml

weakness, and fasciculation in the distal upper and lower limbs bilaterally. He had fasciculation in the tongue and respiratory muscle weakness with a low oxygen saturation level (SpO_2, 86%). He also had memory disturbances accompanied by positive snout and palmomental reflexes and difficulty with mental activities of daily living. He had no sensory disturbance. We diagnosed him as having definite ALS with CI (Figure 3A).

Case ALS-P 1

A 65-year-old (in 2008) woman of the Auyu tribe, a resident of Pies along the Edera River, had a history of progressive motor weakness and gait disturbance for 5 years. She could not walk without support. Neurological examination revealed a resting tremor, cogwheel rigidity in the upper and lower limbs, bradykinesia throughout, postural impairment, UMN and LMN signs with a positive Babinski sign, hyperreflexia in the upper and lower limbs bilaterally, and thenar and hypothenar muscle atrophies. She had no CI or sensory disturbance. We diagnosed her as having ALS-parkinsonism and, more specifically, with severe parkinsonism (Hoen and Yahr IV) simultaneously complicated by motor neuron signs indicative of possible ALS. She died in 2009. Her younger sister was case P 6. The subject had ALS-parkinsonism, and her sister reported that their cousin showed tremor and gait disturbance, which were interpreted to be consistent with parkinsonism (Figure 3B).

Case ALS-P 2

A 76-year-old (in 2011) man of the Auyu tribe, a resident of Ogorito, had a history of tremor in both hands for 5 years in 2010. Neurological examination revealed cogwheel

For peer review only - http://bmjopen.bmj.com/site/about/guidelines.xhtml

rigidity in his upper and lower limbs, bradykinesia, and UMN signs with hyperreflexia in bilateral upper and lower limbs. We diagnosed him as having parkinsonism (Hoen and Yahr II) in 2010. He was examined again in 2011, by which time his parkinsonism was complicated by not only postural impairment but also muscle weakness and atrophy, and spasticity of the upper and lower limbs with Babinski signs. He had no CI or sensory disturbance. We diagnosed him as having parkinsonism (Hoen and Yahr III) with possible ALS; parkinsonism was accompanied by ALS later. He died in 2011 of unknown causes.

We also diagnosed another case of ALS-P3 parkinsonism (Hoen and Yahr III) with possible ALS; parkinsonism was accompanied by ALS later.

Case ALS-P-CI 6

A 50-year-old (in 2012) man of the Auyu tribe, a resident of Benggo along the Edera River, had a history of dysarthria for 2 years. Neurological examination revealed UMN and LMN signs with a positive Babinski sign, muscle atrophy, and weakness in the tongue, proximal upper limb, shoulder, and tibialis anterior muscles. He had cogwheel rigidity in the wrist and elbow, bradykinesia throughout, a positive Myerson sign, and impairment of coordination and postural reflex. He had memory loss, bradyphrenia, slow speech, and positive snout and palmomental reflexes with difficulty performing activities of daily living. We diagnosed him as having a definite ALS and parkinsonism (Hoen and Yahr III) with CI (Figure 3C).

Case P 7

For peer review only - http://bmjopen.bmj.com/site/about/guidelines.xhtml

For peer review only

A 52-year-old (in 2010) woman of the Auyu tribe who lived in Mopio along the Edera River had a history of progressive gait disturbance with parkinsonian signs, including resting tremor, cogwheel rigidity, anterior bending, and bradykinesia for 3 years in 2008. In 2008, we diagnosed her as having parkinsonism (Hoen and Yahr II). By the time of the follow-up survey in 2010, she could not stand by herself and had postural impairment with parkinsonian hands bilaterally. She had no UMN or LMN signs, no CI, and no sensory disturbance. We diagnosed her as having parkinsonism (Hoen and Yahr IV; Figure 3D).

DISCUSSION

Internal migration and migration from outside the province are the most significant causes of demographic, social, and cultural change in Papua. The populations in the survey sites were composed of former hunter-gatherers who lived in the forest and who were brought out of the forest and assembled in villages by Dutch colonialists. The villages along the Ia River were founded in 1937–1951.[10] The emigration program called transmigration (*transmigrasi*) started in Irian Jaya in 1964. Local migrants and transmigrants from outside Irian Jaya increased, resulting in various changes, including the use of food and medicine.[16,17] Considering that Gadjusek et al. found out the high prevalence of ALS at the first survey in 1962, the neurodegenerative disease existed before the introduction of manufactured items.[1]

In the present survey, we performed full neurological examinations in 46 subjects with ALS and/or parkinsonism between 2001 and 2012. Among the subjects, 17 were probable-definite cases of pure ALS, 13 were overlapping ALS (possible, probable, or

For peer review only - http://bmjopen.bmj.com/site/about/guidelines.xhtml

definite) and parkinsonism, and 16 were parkinsonism. Nine cases of ALS and/or parkinsonism had a complication of CI. During the study period, the diagnosis in some patients changed from pure parkinsonism to ALS-parkinsonism, and their disease progressed in severity. Some subjects had both ALS and parkinsonism on their first examination. It is thus evident that overlapping ALS and parkinsonism with or without CI still exists in the specified region of Papua.

Gajdusek et al. reported diagnosing 97 cases (57 cases clinically confirmed and 40 cases not clinically confirmed) of ALS during the 6 surveys they conducted from 1974 to 1981.[1] However, they reported neurological findings of only 32 of these cases, in only 16 of which the reported findings of UMN, LMN, and parkinsonian signs justify a diagnosis of probable or definite ALS. The 16 cases comprise 12 cases of pure ALS and 4 cases of overlapping ALS-parkinsonism. Among the 32 ALS patients, three had a positive family history (9%); that is, 2 had sisters with ALS, and 1 who had ALS-parkinsonism had a family history of ALS. None of the ALS patients had a family history of parkinsonism. The authors also reported their neurological findings in 13 subjects in whom parkinsonism was diagnosed. They reported their findings from full neurological examinations of 10 of these parkinsonism cases; according to their reported neurological signs, these comprised 8 patients of parkinsonism, including 5 with dementia and 2 with overlapping ALS-parkinsonism with dementia.[1] Among the 13 parkinsonism patients, three had a positive family history (23%). In summary, we believe that in the 26 cases reported by Gajdusek et al., the full neurological examination findings comprised 12 pure ALS cases (probable or definite), 6

For peer review only - http://bmjopen.bmj.com/site/about/guidelines.xhtml

overlapping ALS-parkinsonism, and 8 parkinsonism with/without dementia.[1] Spencer et al reported 2 cases of ALS and three cases of parkinsonism, including 1 with dementia, in the survey in 1987. He also reported 3 subjects with parkinsonism overlapping UMN signs, including 1 with dementia in 1990. We have here reported 17 cases of pure ALS (probable or definite), 13 cases of overlapping ALS-parkinsonism, and 16 cases of parkinsonism with or without CI in the 46 cases we neurologically examined fully. Thus, we identified many cases of overlapping ALS and parkinsonism (n = 13, 28%) like that observed in previous reports (n = 6, 23% and n = 3, 38%).[1, 10] We also identified many cases with CI (n = 9, 20%) like that observed in previous reports (n = 7, 27% and n = 2, 25%).[1, 10] Functional disabilities attributable to CI were recognized in activities of daily living or in their livelihood in all the cases with CI in this report.

Gajdusek et al. reported 13 cases of ALS and three cases of parkinsonism in 1975 in the villages along the Ia River (population, 2000).[1] The 13 ALS cases consisted of 10 clinically confirmed ones and 3 others that were not clinically confirmed. We compared the prevalence of probable-definite ALS and parkinsonism according to the report of Gajdusek et al. with our more recent findings. A crude point prevalence of ALS consistent with clinically probable to definite ALS (El Escorial criteria) may be estimated between 500 (95% confidence interval, 191–809/100,000 people) and 650 (298–1002) and that of parkinsonism was 150 (0–320/100,000 people) in 1975 along the Ia River. In our 2010 survey (population, 4500), we identified 6 probable-definite cases of ALS in this region (ALS 8, 10–13, and 17; 133 [27–-240]/100,000 people) and 7 of parkinsonism (possible ALS-P 2, 3, 9, and 13, and P 10, 15, and 16; 156 [40–

For peer review only - http://bmjopen.bmj.com/site/about/guidelines.xhtml

271]/100,000 people).

Gajdusek et al. reported 8 cases of ALS and 6 cases of parkinsonism in 1975 in the villages along the Edera and Dumut rivers (population, 1950). The 8 cases of ALS consisted of 7 clinically confirmed cases and 1 unconfirmed case. The crude point prevalence of ALS consistent with clinically probable to definite ALS (El Escorial criteria) may be estimated between 359 (94–624)/100,000 people and 410 (127–694)/100,000 people, and that of parkinsonism was 308 (62–554)/100,000 people in 1975 along the Edera and Dumut rivers. In our survey of this region in 2010 (population, 4100), we identified 5 probable-definite cases of ALS (ALS 9, 14, and 16, and ALS-P 5, 6; 122 [15–229]/100,000 people) and 7 cases of parkinsonism (possible ALS-P 10 and 11, and P 7, 8, and 11–13; 171 [44–297]/100,000 people; Table 4). As this was not a complete inventory survey, the actual prevalence may be a little higher than our estimation.

The population of Bade increased by a factor of 4 during 31 years (from 1000 in 1975 to 3800 in 2006), and those of villages along the Ia River (from 2000 to 4500) and the Edera and Dumut rivers (from 1950 to 4100) have increased 2-fold.[1,15] The subjects born outside Papua were only 2 (Maluku) among the 46 subjects with ALS and/or parkinsonism with/without CI in this survey, ie 44 were of Papuan genetic stock and 2 (from Maluku) were non-Papuan. The prevalence of the diseases among the local Papuan residents in this survey may be underestimated compared with the former report after taking into account the increase in the numbers of local migrants inside and transmigrants from outside Papua.[16,17,18]

For peer review only - http://bmjopen.bmj.com/site/about/guidelines.xhtml

As the median age and life expectancy in the field sites in Papua are estimated to be lower than the global median and fewer people may live beyond the age of 40 or 50 years,[18,19] the prevalence of the diseases in this survey may be underestimated compared with the global mean. As the median age and life expectancy in 1975 may be lower than those in 2010,[20] the prevalence of the diseases in the report by Gajdusek et al. might be underestimated compared with that in our survey.

The prevalence of ALS in this report was found to be three times lower than found by Gajdusek et al. Even after taking into account the change of population and age structure in the field sites, our findings show that the prevalence of ALS appears to have decreased over the past ~30-35 years since the report of Gajdusek et al.[10] This is consistent with the previous report by Spencer et al. However, it is still much higher than the global mean (incidence, approximately 2/100,000; prevalence, approximately 6/100,000) [21-26]; in particular, the prevalence of overlapping ALS and parkinsonism is especially high in Papua.

In the report by Gajdusek et al., who provided village-by-village data showing the number of cases of ALS and parkinsonism, there were many cases in the remote villages such as Bosma, Pies, Homlikya, Yibin, Sien, and Ogorite, which were less visited and least developed.[1, 2] In this survey, many cases (three ALS/ALS-parkinsonism) were found in the remotest village of Ogorito along the Ia River. Two cases of ALS/ALS-parkinsonism were also found in Bosma, Homlikya, and Yibin; 1 ALS and 2 cases of parkinsonism in Sien; 1 ALS and 1 parkinsonism in Pies, which consisted also of remote villages. While there were 8 cases of

For peer review only - http://bmjopen.bmj.com/site/about/guidelines.xhtml

ALS/ALS-parkinsonism in Bade and 2 in Gimikya, nearest to the port of the Digul River, none was reported by Gajdusek et al. in those sites. This increase may be brought about by local migrants from the affected villages.

Overlap of ALS-parkinsonism and dementia has also been reported in patients with ALS/PDC in Guam and Kii, Japan.[4, 27-33] Hirano et al. provided clinicopathological evidence of 47 cases of PDC in Guam in 1961, comprising the following 3 groups: (1) 30 cases of PDC; (2) 8 cases of parkinsonism with dementia with clinical evidence of UMN impairment; and (3) 9 cases of parkinsonism with dementia with clinical evidence of UMN and LMN impairment. At least 15 of these cases (31.9%) had a family history of ALS, parkinsonism, or both.[4] Kuzuhara and Kokubo et al. surveyed neurological diseases in Kii, Japan, in the 1990s and found not only a continuously high incidence of ALS but also neuropathologically verified cases of PDC. ALS and PDC frequently affected the same individuals simultaneously and the members of the same family. These authors reported 37 cases of ALS/PDC between 1996 and 2006, a high proportion of whom (78%) had a positive family history, as well as clinical examples of pure ALS, PDC, and overlapping ALS and PDC. They also reported neuropathological changes common to cases of ALS and PDC in 12 cases of ALS/PDC, resembling those found in patients with ALS/PDC in Guam [28-33] In our more recent study in Papua, such as the previous studies in Kii, Japan, and Guam, we identified many cases of pure ALS and ALS overlapping with parkinsonism with/without CI. The prevalence of CI was lower in our study sites than in Guam and Kii.[4, 27-33] CI might have been underestimated, and mild CIs or some mild dementia might have been overlooked by our neurological examinations in the field.

For peer review only

For peer review only - http://bmjopen.bmj.com/site/about/guidelines.xhtml

Our study was based only on clinical findings, and there are the limitations of not having electromyogram data, DNA analysis data, or any autopsy data from this population. Without such data, it is difficult to be certain how these patients fit into sporadic ALS or Parkinson disease and how they compare with ALS/PDC on Guam and in the Kii peninsula. However, we did identify many patients with overlapping ALS-parkinsonism in this study. The overlap of ALS and/or parkinsonism with CI suggests that it may be the same disease entity as the ALS/PDC diagnosed in Guam and Kii.

The proportion of males in the populations of Bade, the villages along the Ia River, and the villages along the Edera and Dumut rivers were 53%, 51%, and 50%. The proportion of males was high especially among the subjects with ALS-parkinsonism (85%) and ALS (59%) compared with among those with pure parkinsonism (50%) in our report. The proportion of males was higher among the subjects with ALS (63%) and parkinsonism (76%) in the report by Gadjusek et al., in which there were 3 male cases (75%) among the 4 ALS-parkinsonism cases. The high risks for ALS and PDC among the males were recognized in Guam and Kii.[1,7,27]

Indigenous people of Papua still retain aspects of their traditional lifestyles, such as hunting, gathering, fishing, and eating *sago* (trunk of *Metroxylon* sp.). They also drink water from shallow wells. However, with the spread of a market economy, recent increases in the numbers of rubber plantations and progress in transportation have been causing lifestyle changes in the villages in the south coastal area of Papua. As the diseases are declining as in Guam and Kii, the disease aetiology in Papua may be dominated by environmental factors. Genetic, culture-related and/or family-specific

For peer review only - http://bmjopen.bmj.com/site/about/guidelines.xhtml

factors may also play a role as evidenced by the family history of disease in 40% of the subjects in this survey in Papua and 80% in Kii.

Various possible causal genetic and environmental factors for these conditions have been reported. The environmental factors of low concentrations of calcium and magnesium in drinking water were hypothesized. [1,34] There is a recent report showing an association between the increase in ALS incidence and the change in drinking water source in the Kii Peninsula.[35] Spencer et al. pointed out that this explanation was inconsistent with a declining prevalence of ALS in a sessile population dependent on an unchanging supply of river water, which this report may support.[10]

Spencer et al. reported the discovery that cycad seed was used as a poultice in Papua, as in Guam. [36] Cycad-derived products were shown as the risk factor of dementia, mild CI, and PDC in Guam with the epidemiological evidence.[37] Although cycad had been suspected as the causal factor of Kii ALS,[38] people in Japan, including the Kii Peninsula, had never consumed cycad as daily food, although a small amount of cycad flour might have been taken by a few people as an ingredient of herb medicine.[39] We did not examine the association of prior skin wounds and exposure to cycad poultice with the diseases. Skin change and pigmentary retinopathy were not examined in this survey.[40, 41] Further studies are needed.

Genetic abnormalities that had been previously established to be associated with familial or sporadic ALS, dementia, familial Parkinson disease, and parkinsonism were looked for in the cases in Kii, but none of them were identified.[28,42] Hermousura et

For peer review only - http://bmjopen.bmj.com/site/about/guidelines.xhtml

al. have discovered a nuclear mutation in the calcium/magnesium membrane ion channel transient receptor potential melastatin 7 (*TRPM7*) gene in a subset of patients with ALS/PDC in Guam.[43] They reported that a TRPM7 variant that is associated with altered sensitivity to magnesium may have contributed to the pathogenesis of ALS and PDC in 2 Guamanians. *TRPM7* was not found to be associated with ALS/PDC in the Kii peninsula of Japan.[44] Ishiura et al. have recently discovered *C9ORF72* repeat expansion in some cases of ALS in the Kii peninsula of Japan; this repeat expansion partly accounts for the high prevalence of ALS in this region.[45] However, the responsible gene(s) remain unproved in almost all cases of ALS/PDC in both Guam and Kii.

Various environmental factors and multiple genetic factors are suggested to be underlying causes of these diseases, but these links are so far unproven.[33,46]

In Guam, although the incidence of pure ALS has declined markedly, the prevalence of PDC is still high.[47] The prevalence of dementia among elderly Chamorros (earliest known inhabitants of the Mariana Islands, which include Guam) is also reportedly relatively high.[48] In addition, in Kii the incidence of pure ALS has declined markedly, and the prevalence of PDC and ALS/PDC or ALS with dementia recently increased.[30,33] These changing patterns of ALS/PDC incidence in Guam and Kii might be caused by changes in environmental and socioeconomic factors as well as aging.[48,49]

The mean age of onset in our cases was 46 years for ALS, 53 years for ALS-parkinsonism, and 46 years for parkinsonism. Thus, they were older than the

For peer review only - http://bmjopen.bmj.com/site/about/guidelines.xhtml

previously reported cases of ALS (33 years) and parkinsonism (43 years).[1] In our cases, the mean duration of subjective illness was 7.0 years for ALS, 6.3 years for ALS-parkinsonism, and 6.3 years for parkinsonism. The duration of ALS in our data was longer than not only the world mean of 3 years[21-26] but also the previously reported 3.5 years for ALS in Papua.[1] The mean age of onset may recently have increased, and the mean disease duration became longer in subjects with ALS. The prevalence rate of cases with bulbar sign was 37% in 30 of pure ALS or ALS-parkinsonian in our report, and it was as the same as in the report of Gadjdusek (40%) and classical ALS (39%).[2] The change in onset and course of ALS in recent years might be attributable to the aging population and changing environmental and socioeconomic factors.

On-going follow-up surveys should be performed to evaluate changes in the clinical types of ALS and/or parkinsonism with or without CI and accompanying changes in environmental and socioeconomic factors, as have occurred in Guam and Kii.

In conclusion, the prevalence of ALS in the Papuan disease focus has decreased over the past ~30-35 years but, in 2010, it remained higher than the global average. During this period, there has been an influx of migrants from inside and transmigrants from outside Papua that has increased the size and changed the composition of the population in the villages studied. There may be the possible impact of the expanded population on prevalence estimates. There are still many cases of pure ALS and overlapping ALS-parkinsonism and CI in Papua, as has previously been reported in Guam and Kii. The disease continues to impact native Papuans but may also affect a small number of

For peer review only - http://bmjopen.bmj.com/site/about/guidelines.xhtml

non-Papuan immigrants (2 of 46 or 3-4%) who adopt the local lifestyle. Environmental factors may play important etiologic role. Further on-going follow-up surveys focusing on the clinical epidemiology and aetiology of these neurodegenerative diseases in association with ecological and environmental factors in Papua are needed. Future studies, including neuropathological examinations, should be performed when the local villagers in this region of Papua, Indonesia, are willing to consent to autopsies.

ACKNOWLEDGMENTS

The authors wish to thank all the participants in the villages who volunteered for the survey, the village leaders, and Mappi Regency officials. We are deeply indebted to doctors Imelda Mandagi, Reginald Nangoy, and Christha Z. Tamburian and their staff at Puskesmas Bade, and to Arifin Wasaraka, Lazarus Revasy, Yosefina Griapon, Arius Togodly, Kerry Yarangga, and Dolfinus Y. Bouway at Cenderawasih University for their invaluable contributions to the study. Andre Liem and Herry Wondiwoy at Papua Tour Guides Community and S.E. Amos at Bade High School provided logistic and language assistance. On the Japanese side, we acknowledge the technical support and advice of Katsuyuki Eguchi (Nagasaki University), Yasushi Osaki (Kochi University), Hidekazu Tomimoto (Mie University), Taro Yamauchi (The University of Tokyo), Hideyuki Onishi (Doshisha Women's College), Tetsuya Inamura (Aichi Prefectural University), Wenling Chen, Yumi Kimura, Yasuko Ishimoto, and Hissei Imai (Kyoto University), and Yasuyuki Kosaka, Yukiko Kita, and Chizu Wada (Research Institute for Humanity and Nature).

For peer review only - http://bmjopen.bmj.com/site/about/guidelines.xhtml

AUTHOR CONTRIBUTIONS

Okumiya had full access to all the data in the study and takes responsibility for the integrity of the data and the accuracy of the data analysis.

Study concept and design: Okumiya, Matsubayashi.

Acquisition of data: Okumiya, Wada, Fujisawa, Ishine, Garcia del Saz Hirata, Kuzuhara, Kokubo, Seguchi, Sakamoto, Manuaba, Matsubayashi.

Analysis and interpretation of data: Okumiya,Wada, Sakamoto, Fujisawa, Matusbayashi.

Drafting of the manuscript: Okumiya,Wada, Sakamoto, Fujisawa, Matusbayashi.

Critical revision of the manuscript for important intellectual content: All authors.

Statistical analysis: Okumiya.

Obtained funding: Okumiya, Wada, Kuzuhara, Kokubo, Matsubayashi

Administrative, technical, or material support: Okumiya, Watofa, Rantetampang, Matsubayashi.

Study supervision: Okumiya, Matsubayashi.

COMPETING INTERESTS : None declared

FUNDING

K. Okumiya receives research support from Grant-in-Aid for Scientific Research (A) from the Japan Society for the Promotion of Science (JSPS). K. Matsubayashi receives research support from Grant-in-Aid for Scientific Research (A) from the JSPS and is an editor of the journal *Geriatrics & Gerontology International*. T. Wada receives research

For peer review only

support from Grant-in-Aid for Scientific Research (C) from the JSPS. Y. Kokubo and S. Kuzuhara receive support from Grants-in-Aid from the Research Committee of CNS Degenerative Diseases and the Research Committee on the Muro disease (Kii amyotrophic lateral sclerosis/Parkinsonism-dementia), the Ministry of Health, Labour and Welfare of Japan.

DATA SHARING

Okumiya had full access to all the data in the study and takes responsibility for the integrity of the data and the accuracy of the data analysis.

REFERENCES

1. Gajdusek DC, Salazar AM. Amyotrophic lateral sclerosis and parkinsonian syndromes in high incidence among the Auyu and Jakai people of West New Guinea. Neurology 1982;**32**:107–26.
2. Gajdusek DC. Foci of motor neuron disease in high incidence in isolated populations of East Asia and the Western Pacific. In: Lewis P Rowland, ed. Human Motor Neuron Diseases. New York: Raven Press 1982:363–93.
3. Kurland LT, Mulder DW. Epidemiologic investigations of amyotrophic lateral sclerosis. 1. Preliminary report on geographic distribution and special reference to the Mariana Islands, including clinical and pathologic observations. Neurology 1954;**4**:438–48.
4. Hirano A, Kurland LT, Krooth RS, et al. Parkinsonism-dementia complex, an endemic disease on the island of Guam. I. Clinical features. Brain 1961;**84**:642–61.

For peer review only - http://bmjopen.bmj.com/site/about/guidelines.xhtml

5. Hirano A, Malamud N, Kurland LT. Parkinsonism-dementia complex, an endemic disease on the island of Guam. II. Pathological features. Brain 1961;**84**:662–79.

6. Kimura K, Yase Y, Higashi Y, et al. Epidemiological and geomedical studies on ALS and allied diseases in Kii peninsula (Japan): preliminary report. Proc Jpn Acad 1961;**37**:417–20.

7. Shiraki H, Yase Y. Amyotrophic lateral sclerosis in Japan. In: Vinken PJ, Bruyn GW, eds. Handbook of clinical neurology Vol. 22, System disorders and atrophies, Part II. Amsterdam: Elsevier Science 1975:353–419.

8. Garruto RM, Yanagihara R, Gajdusek DC. Disappearance of high incidence ALS and PDC on Guam. Neurology 1985;**35**:193–8.

9. Yoshida S. ALS tahatsuchi niokeru kankyoyoin to aluminium no neuron hensei eno kanyo nitsuite.[Environmental factors in western Pacific foci of ALS and a possible pathogenetic role of aluminum (Al) in motor neuron degeneration] (in Japanese with English abstract). Rinsho Shinkeigaku [Clin Neurol] 1991;**31**:1310–2.

10. Spencer P, Palmer VS, Ludolph AC. On the decline and etiology of high-incidence motor system disease in West Papua (Southwest New Guinea). Mov Disord 2005;**20(**Suppl 12):S119–126.

11. Wada T, Matsubayashi K, Okumiya K, et al. Health status and subjective economic satisfaction in West Papua. Lancet 2002;**360**:951.

12. Okumiya K, Fujisawa M, Ishine M, et al. Nishi New Guinea chiiki (Indonesia/ Papua shu) no shinkeihensei shikkan no jittai. 2001-2002 nen, 2006-2007 nen no fieldwork yori. [Fieldwork survey of neurodegenerative diseases in West New-Guinea in 2001-02 and 2006-07] (in Japanese). Rinsho Shinkeigaku [Clin Neurol] 2007;**47**:977–8.

For peer review only

13. Brooks BR. El Escorial World Federation of Neurology criteria for the diagnosis of amyotrophic lateral sclerosis: subcommittee on Motor Neuron Diseases/Amyotrophic Lateral Sclerosis of the World Federation of Neurology Research Group on Neuromuscular Diseases and the El Escorial "Clinical Limits of ALS" Workshop Contributors. J Neurol Sci 1994;**124**:96–107.

14. Hoehn MM, Yahr MD. Parkinsonism: onset, progression and mortality. Neurology. 1967;**17**:427–42.

15. Kontor Statistik Distrik Edera. Laporan Kependudukan, Kabupaten Mappi Distrik Edera 2006 (December).

16. Manning C., Rumiak M. Irian Jaya: Economic change, migrants, and indinenous welfare. In Hill H ed. Unity and diversity: Regional Economic Development in Indonesia since 1970. Oxford University Press, Okford.p77-106, 1991.

17. Timmer J. A brief social and political history of Papua, 1962-2005. In Marshall AJ and Beehler BM ed. The Ecology of Indonesian Papua Part Two. Periplus Editions, Hong Kong. p1098-1124, 2007.

18. Badan Pusat Statistik, Sensus Penduduk 2010. http://sp2010.bps.go.id/index.php

19. United Nations , Department of Economic and Social Affairs, Population Division. World Population Prospects The 2012 Revision *Volume II: Demographic Profiles,* http://esa.un.org/unpd/wpp/index.htm

20. Kontor Statistik Kabupaten Merauke. Laporan Kependudukan, Kabupaten Merauke 1997

21. Armon C. Amyotrophic lateral sclerosis. In: Neuroepidemiology from principles to practice. USA: Oxford University Press 2004:162–87.

22. McGuire V, Longstreth WT Jr, Koepsell TD, et al. Incidence of amyotrophic lateral

For peer review only

sclerosis in three counties in western Washington state. Neurology 1996 ;**47**:571–3.

23. Sorenson EJ, Stalker AP, Kurland LT, et al. Amyotrophic lateral sclerosis in Olmsted County, Minnesota, 1925 to 1998. Neurology 2002; **23**:280–2.

24. O'Toole O, Traynor BJ, Brennan P, et al. Epidemiology and clinical features of amyotrophic lateral sclerosis in Ireland between 1995 and 2004. J Neurol Neurosurg Psychiatry 2008;**79**:30–2.

25. Chiò A, Mora G, Calvo A, et al. Epidemiology of ALS in Italy: a 10-year prospective population-based study. Neurology 2009;**72**:725–31.

26. Factor-Litvak P, Al-Chalabi A, Ascherio A, et al. Current pathways for epidemiological research in amyotrophic lateral sclerosis. Amyotroph Lateral Scler Frontotemporal Degener 2013;**14**(Suppl 1):33–43.

27. Rodgers-Johnson P, Garruto RM, Yanigahara R, et al. Amyotrophic lateral sclerosis and parkinsonism-dementia on Guam: a 30-year evaluation of clinical and neurological trends. *Neurology.* 1986;36:7-13.

28. Kuzuhara S, Kokubo Y, Sasaki R, et al. Familial amyotrophic lateral sclerosis and parkinsonism-dementia complex of the Kii peninsula of Japan: clinical and neuropathological study and tau analysis. Ann Neurol 2001;**49**:501–11.

29. Kuzuhara S, Kokubo Y. Atypical parkinsonism of Japan: amyotrophic lateral sclerosis-parkinsonism-dementia complex of the Kii peninsula of Japan (Muro disease): an update. Mov Disord 2005;**20**(Suppl 12):S108–13.

30. Kuzuhara S. Kii ALS/PDC (Murou byou) no rekishi, gainen, ekigaku. [ALS-parkinsonism-dementia complex of the Kii peninsula of Japan (Muro disease). Historical review, epidemiology and concept] (in Japanese with English abstract). Rinsho Shinkeigaku [Clin Neurol] 2007;**47**:962–5.

For peer review only - http://bmjopen.bmj.com/site/about/guidelines.xhtml

31. Kokubo Y. Kiihanto no kinishukuseisokusakukokasho/ parkinson chihou hukugo no rinshou, shinkei byourigakuteki kenkyu. [Clinical aspects, imaging and neuropathology of Kii ALS/PDC] (in Japanese with English abstract). Rinsho Shinkeigaku [Clin Neurol] 2007;**47**:966–9.

32. Kuzuhara S. Kii hanto no Murobyo (Kinishukuseisokusakukokasho/ parkinson/ ninchisho hukugo) [Muro disease or ALS–parkinsonism–dementia complex of the Kii peninsula of Japan] (in Japanese with English abstract). Rinsho Shinkeigaku [Clin Neurol] 2007;**47**:695–702.

33. Kuzuhara S, KokuboY. Amyotrophic lateral sclerosis-parkinsonism-dementia complex in the Kii peninsula of Japan (Muro disease): a review on recent research and new concept. In: Michael J. Strong, ed. Amyotrophic lateral sclerosis and the frontotemporal dementias. Oxford: Oxford University Press 2012:39–54.

34. Yase Y. The pathogenesis of amyotrophic lateral sclerosis. Lancet 1972;**2**(7772):292–6.

35. Kihara T, Yoshida S, Kondo T et al. An increase in ALS incidence on the Kii Peninsula, 1960-2009: A possible link to change in drinking water source. *Amyotrophic Lateral Sclerosis,* 2012; 13: 347–50.

36. Spencer PS, Nunn PB, Hugon J, et al. Guam amyotrophic lateral sclerosis-parkinsonism-dementia linked to a plant excitant neurotoxin. Science 1987;**237**(4814):517–22.

37. Borenstein AR, Mortimer JA, Schofield E. et al. Cycad exposure and risk of dementia, MCI, and PDC in the Chamorro population of Guam. Neurology. 2007;68:1764-71.

38. Spencer PS, Ohta M, Palmer VS. Cycad use and motor neuron disease in Kii peninsula of Japan. Lancet 1987;**2**(8573):1462–3.

For peer review only

For peer review only - http://bmjopen.bmj.com/site/about/guidelines.xhtml

39. Iwami O, Niki Y, Watanabe T, and Ikeda M. Motor neuron disease on the Kii Peninsula of Japan: Cycad exposure. Neuroepidemiology 1993;12:307-312.

40. Ono S, Waring SC, Kurland L et al. Guamanian neurodegenerative disease: ultrastructural studies of skin J. Neurol. Sci., 146 (1997), pp. 35–40

41. Kokubo Y, Ito K, Kuzuhara S. Ophthalmomyiasis-like pigmentary retinopathy in ALS/PDC in the Kii peninsula of Japan. Neurology. 2003 May 27;60(10):1725-6.

42. Tomiyama H, Kokubo Y, Sasaki R, et al. Mutation analyses in amyotrophic lateral sclerosis/parkinsonism-dementia complex of the Kii peninsula, Japan. Mov Disord 2008;**23**(16):2344–8.

43. Hermosura MC, Nayakanti H, Dorovkov MV, et al. A TRPM7 variant shows altered sensitivity to magnesium that may contribute to the pathogenesis of two Guamanian neurodegenerative disorders. Proc Natl Acad Sci 2005;**102**(32):11510–5.

44. Hara K, Kokubo Y, Ishiura H, et al. TRPM7 is not associated with amyotrophic lateral sclerosis-parkinsonism dementia complex in the Kii peninsula of Japan. Am J Med Genet B Neuropsychiatr Genet 2010;**153B**(1):310–3.

45. Ishiura H, Takahashi Y, Mitsui J, et al. *C9ORF72* Repeat expansion in amyotrophic lateral sclerosis in the Kii peninsula of Japan. Arch Neurol 2012;**69**(9):1154–8.

46. Garruto RM, Yanagihara R. Contributions of isolated Pacific populations to understanding neurodegenerative diseases. Folia Neuropathol 2009;**47**(2):149–70.

47. Plato CC, Garruto RM, Galasko D, et al. Amyotrophic lateral sclerosis and parkinsonism-dementia complex of Guam: changing incidence rates during the past 60 years. Am J Epidemiol 2003;**157**:149–57.

48. Galasko D, Salmon D, Gamst A, et al. Prevalence of dementia in Chamorros on Guam: relationship to age, gender, education, and APOE. Neurology 2007;**68**:1772–

For peer review only - http://bmjopen.bmj.com/site/about/guidelines.xhtml

81.

49. Plato CC, Galasko D, Garruto RM, et al. ALS and PDC of Guam: forty-year follow-up. Neurology 2002;**58**:765–73.

Figure and table legends

Table 1. Relevant characteristics and neurological signs of cases with ALS with/without cognitive impairment in Papua, 2001–2012.

Abbreviations: ALS = amyotrophic lateral sclerosis; BK = bradykinesia; BS = Babinski sign; DTR = deep tendon reflex; Dys = dysphagia/dysarthria; F= female; Fasc = fasciculation; LMN = lower motor neuron signs; M = male; MA = muscle atrophy with weakness; NC = not confirmed by examination; P = parkinsonism; PI = postural impairment; R = rigidity; T = tremor; TA = tongue atrophy; U/L: upper limbs/lower limbs; UMN = upper motor neuron signs.

Age: at the time of final survey; Duration: between subjective onset of illness and last survey or death; Alive: alive in 2012; Onset: subjective onset of the illness; Deep tendon reflexes, -: absent, 1: hypoactive, 2: normal, 3: hyperactive, 4: markedly hyperactive with ankle clonus.

Other signs are classified as: -: absent; ±: questionably present; +: present; ++: markedly present.

Family history 1: cousin (possible ALS); 2: grandfather (NC); 3: sister (ALS15); 4: cousin's daughter (P16); 5: cousin (NC); 6: father (NC); 7: sister (ALS 7); 8: sister (P 6) and cousin (NC); 9: father (NC); 10: son: (NC); 11: sister (ALS-P 1) and cousin (NC);

For peer review only - http://bmjopen.bmj.com/site/about/guidelines.xhtml

12: mother and father (NC); 13: grandmother (NC); 14: mother (NC); 15: ALS 10 (mother's cousin).

Table 2. Relevant characteristics and neurological signs in cases with overlap of ALS and parkinsonism with/without cognitive impairment in Papua, 2001–2012.

Table 3. Relevant characteristics and neurological signs in cases with parkinsonism with/without cognitive impairment in Papua, 2001–2012.

Table 4. Cases of ALS and parkinsonism according to field site

*; Active cases in 2007 in Bade

#; Active cases in 2010 in the villages along the Ia, Edera, and Dumut rivers

Figure 1. Map of the field sites

A. Large-scale map of the southern coastal plain in Papua, Indonesia.

B. Smaller scale map of the southern coastal plain in Papua, Indonesia showing rivers.

C. Detailed map showing villages in the southern coastal area in Papua in which all confirmed cases of ALS and/or parkinsonism with/without cognitive impairment were seen from 2001 to 2012.

Figure 2. Photographs of patient with ALS (Case ALS 12**)**

A: The patient needs assistance from family members to stand.

B: Advanced atrophy of the tongue.

For peer review only

C: There is upper limb-girdle and truncal muscle atrophy with a positive Babinski sign.

D: Advanced thenar muscle atrophy.

Figure 3. Photographs of patients with ALS, ALS-parkinsonism, and parkinsonism

A: Patient with ALS (Case ALS-17) showing muscle atrophy in the distal upper and lower limbs.

B: Patient with ALS-parkinsonism (Case ALS-P 1) showing muscle atrophy in distal upper limbs.

C: Patient with ALS-parkinsonism (Case ALS-P 7) showing atrophy in muscles in the proximal upper limb, shoulder, and tibialis anterior.

D: Patient with parkinsonism (Case P 7) showing typical parkinsonian hands and anterior bending posture.

Parkinsonian hand: Striatal deformities of the hand with abnormal postures that are common in patients with advanced Parkinson's disease.

For peer review only

For peer review only - http://bmjopen.bmj.com/site/about/guidelines.xhtml

For peer review only

For peer review only - http://bmjopen.bmj.com/site/about/guidelines.xhtml

Okumiya

Table 1. Relevant characteristics and neurological signs in cases with pure ALS with/without cognitive impairment in Papua, 2001–2012.

Case number	ALS criteria Yahr stage	Survey year	Village	Age	Sex	Onset (age/year)	Duration (years)	Death (year)	Tribe (origin)	UMN signs DTR U/L	UMN signs BS	LMN signs MA U/L	LMN signs Fasc U/L	Bulbar signs TA	Bulbar signs Dys	Parkinsonian sign T U/L	Parkinsonian sign R U/L	Parkinsonian sign BK	Parkinsonian sign PI	Cognitive impairment	Family history
Pure ALS (n=17)																					
ALS 1	Definite	2001	Bade	35	M	NC	NC	NC	Auyu	3/3	+	++/++	+/+	++	-	-	-	-	-	-	-
ALS 2	Definite	2001	Ogoto	43	F	NC	NC	2002	Auyu	3/3	+	++/++	+/+	++	++	-	-	-	-	-	-
ALS 3	Definite	2002	Senggo	50	M	48/'00	2	NC	Citak	3/3	+	++/++	±/±	±	+	-	-	-	-	-	1
ALS 4	Definite	2007	Homilikia	53	M	51/'05	2	2007	Auyu	3/2	+	++/++	+/+	-	-	-	-	-	-	-	-
ALS 5	Definite	2007/'08	Bade	51	M	49/'06	3	2009	Auyu	3/4	+	+/-	-/-	-	+	-	-	-	-	-	-
ALS 6	Definite	2008	Bade	21	M	11/'98	11	2009	Auyu	4/4	+	++/++	-/-	-	-	-	-	-	-	-	2
ALS 7	Definite	2007/'08/'10	Bade	70	F	57/'97	13	2010	Maluku	3/3	+	++/++	+/-	-	+	-	-	-	-	-	3
ALS 8	Probable	2011	Geturki	53	M	48/'06	5	Alive	Auyu	3/3	-	+/+	-/+	-	-	-	-	-	-	-	-
ALS 9	Definite	2011	Muya	41	F	36/'06	6	2012	Auyu	3/3	+	+/+	-/-	-	-	-	-	-	-	-	-
ALS 10	Probable	2007/'10/'11/'12	Osso	65	F	59/'06	6	Alive	Auyu	3/4	+	+/-	-/-	+	-	-	-	-	-	-	4
ALS 11	Probable	2007/'08/'10/'12	Gimikya	32	M	12/'92	20	Alive	Auyu	4/4	+	++/+	++/-	±	-	-	+/-	-	-	-	5
ALS 12	Definite	2010/'11/'12	Bosma	64	M	53/'01	11	Alive	Auyu	3/4	+	++/++	+/-	++	-	-	-	-	-	-	6
ALS 13	Definite	2012	Asset	70	F	68/'10	2	Alive	Auyu	3/3	-	+/+	-/-	-	-	-	-	-	-	-	-
ALS 14	Probable	2012	Pies	65	F	60/'07	5	Alive	Auyu	3/4	-	+/+	-/-	-	-	-	-	-	-	-	-
ALS 15	Definite	2007	Bade	64	F	62/'05	2	2007	Maluku	3/3	+	++/++	-/-	-	+	-	-	-	-	+	7
ALS 16	Definite	2008/'10/'11	Yibin	25	M	13/'99	13	2012	Auyu	3/4	+	++/+	++/++	-	-	-	-	-	-	+	-
ALS 17	Definite	2012	Ogorito	70	M	66/'08	4	Alive	Auyu	3/3	+	+/+	+/+	-	-	-	-	-	-	+	-

Abbreviations: ALS = amyotrophic lateral sclerosis; BK = bradykinesia; BS = Babinski sign; DTR = deep tendon reflex; Dys = dysphagia/dysarthria; F = female; Fasc = fasciculation; LMN = lower motor neuron signs; M = male; MA = muscle atrophy with weakness; NC = not confirmed by examination; P = parkinsonism; PI = postural impairment; R = rigidity; T = tremor; TA = tongue atrophy; U/L: upper limbs/lower limbs; UMN = upper motor neuron signs.

Age: at the time of final survey; Duration: between subjective onset of illness and last survey or death; Alive: alive in 2012; Onset: subjective onset of illness; Deep tendon reflexes, -: absent, 1: hypoactive, 2: normal, 3: hyperactive, 4: markedly hyperactive with ankle clonus.

Other signs are classified as: -: absent; ±: questionably present; +: present; ++: markedly present.

Family history 1: cousin (possible ALS); 2: grandfather (NC); 3: sister (ALS15); 4: cousin's daughter (P16); 5: cousin (NC); 6: father (NC); 7: sister (ALS 7); 8: sister (P 6) and cousin (NC); 9: father (NC); 10: son: (NC); 11: sister (ALS-P 1) and cousin (NC); 12: mother and father (NC); 13: grandmother (NC); 14: mother (NC); 15: ALS 10 (mother's cousin).

For peer review only - http://bmjopen.bmj.com/site/about/guidelines.xhtml

For peer review only

Okumiya

Table 2. Relevant characteristics and neurological signs in cases with overlap of ALS and parkinsonism with/without cognitive impairment in Papua, 2001–2012.

Case number	ALS criteria Yahr stage	Survey year	Village	Age	Sex	Onset (age/year)	Duration (year)	Death (year)	Tribe (origin)	UMN signs		LMN signs		Bulbar signs		Parkinsonian sign				Cognitive impairment	Family history
										DTR U/L	BS	MA U/L	Fasc U/L	TA	Dys	T U/L	R U/L	BK	PI		
ALS-parkinsonism (n = 13)																					
ALS-P 1	Possible-IV	2008	Pies	65	F	60/'03	6	2009	Auyu	3/3	+	+/-	-/-	-	-	+/+	++/++	++	+	-	8
ALS-P 2	Possible-III	2010/'11	Ogorito	76	M	70/'05	6	2011	Auyu	3/3	+	+/±	-/-	-	-	+/+	+/+	+	+	-	-
ALS-P 3	Possible-III	2010/'11/'12	Homilikia	69	M	65/'08	4	Alive	Auyu	3/2	+	+/-	-/-	-	-	+/+	+/+	+	+	-	-
ALS-P 4	Definite-III	2008	Bade	34	M	18/'92	17	2009	Muyu	3/3	+	+/-	-/-	++	++	-	+/+	+	+	+	9
ALS-P 5	Definite-III	2012	Sien	60	M	58/'10	2	Alive	Auyu	3/3	+	+/+	-/-	-	-	-	+/+	+	+	+	-
ALS-P 6	Definite-III	2012	Benggo	50	M	48/'10	2	Alive	Auyu	2/2	+	+/+	-/-	+	+	-	+/-	+	+	+	-
ALS-P 7	Possible-II	2001	Bade	36	M	NC	NC	NC	Auyu	3/3	+	-/-	-/-	-	-	+	+	+	-	-	-
ALS-P 8	Possible-II	2008/'10	Bade	23	M	11/'98	12	Alive	Auyu	3/4	+	-/-	-/-	-	-	+/+	+/±	+	-	-	-
ALS-P 9	Possible-III	2010/'11	Ogorito	76	M	70/'05	6	2011	Auyu	3/3	+	-/-	-/-	-	-	+/-	+/+	+	+	-	-
ALS-P 10	Possible-III	2008/'10/'11	Yibin	52	M	44/'03	8	Alive	Auyu	3/3	+	-/-	-/-	-	-	+/-	+/+	+	+	-	-
ALS-P 11	Possible-III	2011	Getio	55	F	53/09	2	2011	Auyu	3/3	+	±/-	-/-	-	-	+/+	+/+	+	+	-	-
ALS-P 12	Possible-IV	2001	Bade	40	M	NC	NC	NC	Auyu	3/3	+	-/-	-/-	-	+	+	+	+	+	+	-
ALS-P 13	Possible-III	2012	Bosma	52	M	48/'08	4	Alive	Auyu	3/4	+	-/-	-/-	-	-	-	++/+	+	+	+	-

Abbreviations: ALS = amyotrophic lateral sclerosis; BK = bradykinesia; BS = Babinski sign; DTR = deep tendon reflex; Dys = dysphagia/dysarthria; F = female; Fasc = fasciculation; LMN = lower motor neuron signs; M = male; MA = muscle atrophy with weakness; NC = not confirmed by examination; P = parkinsonism; PI = postural impairment; R = rigidity; T = tremor; TA = tongue atrophy; U/L: upper limbs/lower limbs; UMN = upper motor neuron signs.

Age: at the time of final survey; Duration: between subjective onset of illness and last survey or death; Alive: alive in 2012; Onset: subjective onset of illness; Deep tendon reflexes, -: absent, 1: hypoactive, 2: normal, 3: hyperactive, 4: markedly hyperactive with ankle clonus.

Other signs are classified as: -: absent; ±: questionably present; +: present; ++: markedly present.

Family history 1: cousin (possible ALS); 2: grandfather (NC); 3: sister (ALS15); 4: cousin's daughter (P16); 5: cousin (NC); 6: father (NC); 7: sister (ALS 7); 8: sister (P 6) and cousin (NC); 9: father (NC); 10: son: (NC); 11: sister (ALS-P 1) and cousin (NC); 12: mother and father (NC); 13: grandmother (NC); 14: mother (NC); 15: ALS 10 (mother’s cousin).

For peer review only - http://bmjopen.bmj.com/site/about/guidelines.xhtml

Table 3. Relevant characteristics and neurological signs in cases with parkinsonism with/without cognitive impairment in Papua, 2001–2012.

Case number	ALS criteria Yahr stage	Survey year	Village	Age	Sex	Onset (age/year)	Duration (years.)	Death (year)	Ethnicity	UMN signs DTR U/L	UMN signs BS	LMN signs MA U/L	LMN signs Fasc U/L	Bulbar signs TA	Bulbar signs Dys	Parkinsonian sign T U/L	Parkinsonian sign R U/L	Parkinsonian sign BK	Parkinsonian sign PI	Cognitive impairment	Family history
Parkinsonism (n = 16)																					
P 1	Yahr IV	2001	Kepi	50	M	NC	NC	NC	Jakai	2/2	-	-/-	-/-	-	-	+/+	+/+	++	+	-	-
P 2	Yahr II	2001	Kepi	50	M	NC	NC	NC	Jakai	2/2	-	-/-	-/-	-	-	+	+	+	-	-	-
P 3	Yahr IV	2002	Emete	52	M	49/'99	3	NC	Jakai	2/2	-	-/-	-/-	-	-	+	+	+	+	-	-
P 4	Yahr II	2002	Senggo	41	M	36/'97	5	NC	Citak	2/2	-	-/-	-/-	-	-	+	+	-	-	-	-
P 5	Yahr I	2007	Mur	65	F	61/'03	4	NC	Jakai	2/3	-	-/-	-/-	-	-	+/-	+/-	-	-	-	10
P 6	Yahr IV	2008	Pies	60	F	55/'03	6	2009	Auyu	2/2	-	-/-	-/-	-	-	+/+	+/+	++	+	-	11
P 7	Yahr IV	2008/'10	Mopio	52	F	47/'05	5	Alive	Auyu	3/3	-	-/-	-/-	-	-	++/+	+/+	++	+	-	-
P 8	Yahr II	2011	Sien	57	F	49/03	9	2012	Auyu	3/3	-	-/-	-/-	-	-	+/+	+/+	+	-	-	12
P 9	Yahr IV	2007/'08/'10/'[illegible]	Bade	46	F	40/'06	6	Alive	Auyu	3/3	-	-/-	-/-	-	-	+/+	++/+	+	+	-	13
P 10	Yahr II	2011/'12	Asset	38	M	27/'01	11	Alive	Auyu	3/3	-	-/-	-/-	-	-	+/-	+/-	+	-	-	-
P 11	Yahr III	2011/'12	Sien	54	F	40/'98	14	Alive	Auyu	3/3	-	-/-	-/-	-	-	++/+	++/+	+	+	-	-
P 12	Yahr II	2011/'12	Memes	66	F	64/10	2	Alive	Auyu	3/3	-	-/-	-/-	-	-	+/+	+/+	+	-	-	14
P 13	Yahr II	2012	Harapan	60	M	57/09	3	Alive	Auyu	2/2	-	-/-	-/-	-	-	-	+/+	-	-	-	-
P 14	Yahr II	2007	Mur	50	M	38/'95	12	NC	Auyu	2/4	+	-/-	-/-	-	-	+	+	+	-	-	-
P 15	Yahr I	2007	Homilikia	50	M	NC	NC	Alive	Auyu	2/3	+	-/-	-/-	-	-	+/-	+/±	-	-	-	-
P 16	Yahr III	2011	Osso	35	F	33/'09	2	2011	Auyu	3/2	-	-/-	-/-	-	-	+/+	+/+	+	+	+	15

Abbreviations: ALS = amyotrophic lateral sclerosis; BK = bradykinesia; BS = Babinski sign; DTR = deep tendon reflex; Dys = dysphagia/dysarthria; F = female; Fasc = fasciculation; LMN = lower motor neuron signs; M = male; MA = muscle atrophy with weakness; NC = not confirmed by examination; P = parkinsonism; PI = postural impairment; R = rigidity; T = tremor; TA = tongue atrophy; U/L: upper limbs/lower limbs; UMN = upper motor neuron signs.

Age: at the time of final survey; Duration: between subjective onset of illness and last survey or death; Alive: alive in 2012; Onset: subjective onset of illness; Deep tendon reflexes, -: absent, 1: hypoactive, 2: normal, 3: hyperactive, 4: markedly hyperactive with ankle clonus.

Other signs are classified as: -: absent; ±: questionably present; +: present; ++: markedly present.

Family history 1: cousin (possible ALS); 2: grandfather (NC); 3: sister (ALS15); 4: cousin's daughter (P16); 5: cousin (NC); 6: father (NC); 7: sister (ALS 7); 8: sister (P 6) and cousin (NC); 9: father (NC); 10: son: (NC); 11: sister (ALS-P 1) and cousin (NC); 12: mother and father (NC); 13: grandmother (NC); 14: mother (NC); 15: ALS 10 (mother's cousin).

For peer review only

For peer review only - http://bmjopen.bmj.com/site/about/guidelines.xhtml

Table 4. Cases of ALS and parkinsonism in the field sites

	ALS criteria	Case No.	Crude point prevalence rate (95%CI) among ALS/ ALS-P/ Parkinsonism (2007* or 2010#)
Bade 2007–2012 (population 3800)			
ALS	Definite	ALS 5*	105 (2-208) /100,000 in 2007*
(n=4)	Definite	ALS 6*	
	Definite	ALS 7*	
	Definite	ALS 15*	
ALS-parkinsonism	Definite	ALS-P 4*	53 (0-126) /100,000 in 2007*
(n=2)	Possible	ALS-P 8*	
Parkinsonism (n=1)		P 9*	26 (0-78) /100,000 in 2007*
Ia river 2007–2012 (population 4500)			
ALS	Definite	ALS 4	133(27-240) /100,000 in 2010#
(n=7)	Probable	ALS 8#	
	Probable	ALS 10#	
	Probable	ALS 11#	
	Definite	ALS 12#	
	Definite	ALS 13#	
	Definite	ALS 17#	
ALS-parkinsonism	Possible	ALS-P 2#	89 (2-176) /100,000 in 2010#
(n=4)	Possible	ALS-P 3#	
	Possible	ALS-P 9#	
	Possible	ALS-P 13#	
Parkinsonism		P 10#	67 (0-142) /100,000 in 2010#
(n=3)		P 15#	
		P 16#	
Edera and Dumut rivers 2008–2012 (population: 4100)			
ALS	Definite	ALS 9#	73 (0-156) /100,000 in 2010#
(n=3)	Probable	ALS 14#	
	Definite	ALS 16#	
ALS-parkinsonism	Possible	ALS-P 1	98 (2-193) /100,000 in 2010#
(n=5)	Definite	ALS-P 5#	
	Definite	ALS-P 6#	
	Possible	ALS-P 10#	
	Possible	ALS-P 11#	
Parkinsonism		P 6	122 (15-229) /100,000 in 2010
(n=6)		P 7#	
		P 8#	
		P 11#	
		P 12#	
		P 13#	

*:Active cases in 2007 in Bade

#:Active cases in 2010 in the villages along Ia, Edera and Dumut rivers

95%CI: 95% confidence interval

For peer review only

Amyotrophic lateral sclerosis and parkinsonism in Papua, Indonesia: 2001–2012 survey results

Kiyohito Okumiya[1,2], Taizo Wada[2], Michiko Fujisawa[2], Masayuki Ishine[3], Eva Garcia del Saz[4], Yutaka Hirata[5], Shigeki Kuzuhara[6], Yasumasa Kokubo[7], Harumichi Seguchi[8], Ryota Sakamoto[9], Indrajaya Manuaba[10], Paulina Watofa[10], Andreas L. Rantetampang[11], Kozo Matsubayashi[2]

[1]Research Institute for Humanity and Nature, Kyoto, Japan
[2]Center for Southeast Asian Studies, Kyoto University, Kyoto, Japan
[3]Yasugi Clinic, Shimane, Japan
[4]Center for Regional & International Collaboration, Kochi University, Kochi, Japan
[5]Kitaakita Municipal Hospital, Akita, Japan
[6]Faculty of Health Science, Suzuka University of Medical Science, Mie, Japan
[7]Graduate School of Regional Innovation Studies, Mie University, Mie, Japan
[8]Faculty of Medicine, Kochi University, Kochi, Japan
[9]Hakubi Center for Advanced Research, Kyoto University, Kyoto, Japan
[10]Faculty of Medicine, Cenderawasih University, Papua, Indonesia
[11]Faculty of Public Health, Cenderawasih University, Papua, Indonesia

Correspondence to: Kiyohito Okumiya,
Research Institute for Humanity and Nature
Motoyama 457-4, Kamigamo, Kita-ku, Kyoto 603-8047, Japan
Tel: +81-75-707-2411, Fax: +81-75-707-2509
E-mail: okumiyak@chikyu.ac.jp
Center for Southeast Asian Studies
Kyoto University
46, Shimoadachi-cho, Yoshida , Sakyo-ku, 606-9501, Japan
Tel: +81-75-753-7320
E-mail: okumiya@cseas.kyoto-u.ac.jp

Abstract:296 words
Text:5315 words
4 tables and 3 figures

For peer review only

ABSTRACT

Objective: Only one prior follow-up study of amyotrophic lateral sclerosis (ALS) and parkinsonism in Papua, Indonesia has been carried out since a survey undertaken from 1962 to 1981 by Gajdusek and colleagues. Therefore, to clarify the clinical epidemiology of ALS and parkinsonism in the south coastal region of Papua, the clinical characteristics and prevalence of the diseases in this region were examined and assessed.

Methods: Cases of ALS and parkinsonism were clinically examined during a 2001–2012 survey in Bade and other villages along the Ia, Edera, Dumut, and Obaa rivers in Papua, Indonesia. Possible, probable, and definite ALS were diagnosed clinically by certified neurologists on the basis of El Escorial Criteria. The criteria for diagnosis of parkinsonism were the presence of at least 2 of the 4 following signs: tremor, rigidity, bradykinesia, and postural impairment with a progressive course.

Results: During the survey, 46 cases of ALS and/or parkinsonism were diagnosed within a population range of 7000 (2001–2002) to 13,900 (2007–2012). The 46 cases consisted of 17 probable-definite cases of ALS, including 3 of cognitive impairment (CI); 13 cases of overlap of possible, probable, or definite ALS and parkinsonism, including 5 cases of CI; and 16 cases of parkinsonism, including 1 case of CI. The crude point prevalence rate of pure ALS was estimated as at least 73 (0–156) to 133 (27–240)/100,000 people and that of the overlap of ALS and parkinsonism was at least 53 (0–126) to 98 (2–193)/100,000 in 2007 or 2010 in some regions.

Conclusions: While the prevalence of ALS has decreased over the past ~30-35 years, it remains higher than the global average. There was a high prevalence of overlap of ALS, parkinsonism, and CI, which has been previously reported in Guam and Kii.

For peer review only - http://bmjopen.bmj.com/site/about/guidelines.xhtml

Strengths and limitations of this study

・This study is unique in the epidemiological survey of neurodegenerative diseases during the period between 2001 and 2012 in Papua, Indonesia, one of the foci with the highest incidence of ALS in the world.

・This study recognized the clinical findings of significant overlap of ALS with parkinsonism and cognitive impairment.

・ This study was based only on clinical findings and there are the limitations of not having electromyogram data, DNA analysis, or any autopsy data from this population. Without such data, it is difficult to be certain how these patients fit into sporadic ALS or Parkinson's disease and how they compare with ALS/PDC on Guam and in the Kii peninsula.

For peer review only

INTRODUCTION

Between 1962 and 1980, Gajdusek et al. reported 97 cases of amyotrophic lateral sclerosis (ALS), 18 cases of parkinsonism, and 18 cases of poliomyeloradiculitis (PMR) in the south coastal area of Papua (former Irian Jaya, Indonesia), which had a population of 7,000. The Ia River is in the epicenter of the high-incidence focus of ALS and parkinsonism in Papua.[1,2] Along with Guam and Kii in Japan, Papua (Indonesia) has been considered one of the foci with the highest incidence of ALS in the world.[1-7] The high incidence of ALS and parkinsonism-dementia complex (PDC) in Guam and Kii reportedly no longer exists.[8,9] In 1987, Spencer et al. reported only 2 cases of ALS and three cases of parkinsonism in the villages along the Ia River. In 1990, no cases of ALS and three cases of parkinsonism were reported along the same river; thus, decline or disappearance of ALS was suspected to have also occurred in Papua.[10] No follow-up surveys on ALS and parkinsonism have been conducted in Papua since 1991.

To clarify the clinical epidemiological characteristics of ALS and parkinsonism in the south coastal region of Papua, we conducted a survey of neurodegenerative diseases documented between 2001 and 2012.

METHODS

The survey sites for between 2001 and 2002 (total population, 7000) were the villages of Kepi (population, 800), Emete (800), Ogoto (400), Senggo (2000), and Bade (3000).[11,12] For between 2007 and 2012, the survey sites were the villages along 4 rivers (total population, 13,900), namely Bade along the Digul River (population, 3800

For peer review only - http://bmjopen.bmj.com/site/about/guidelines.xhtml

For peer review only

people), Mur along the Mappi River (1500), the 8 villages along the Ia River (4500), and the 12 villages along the Edera and Dumut rivers (4100 people). The 8 villages along the Ia River were Gimikya (800), Geturki (500), Homlikya (700), Kobeta (600), Asset (600), Osso (300), Bosma (500), and Ogorito (500). The 12 villages along the Edera and Dumut rivers were Benggo (200), Kokoya (500), Sahapikya (400), Harapan (400), Yibin (300), Mopio (300), Pies (300), Memes (300), Habeske (300), Muya (300), Getio (200), and Sien (600; Figure 1). At least 2 of 6 Japanese-certified neurologists (KO, MF, YH, SK, YK, and KM) visited each village with a local neurologist (IM).

The village leaders and medical staff members of the primary healthcare centre in each village agreed to take part in the survey, which was conducted with the permission of the Department of Health of the Papua Provincial Government in cooperation with Cenderawasih University. The purpose of the neurological examinations was explained to all of the villagers. All the subjects volunteered to participate after the announcement of availability of neurological check-ups, which were performed in the community health centres or homes in each field site. While this was not a complete inventory survey, we asked each medical doctor or health staff, and village leader to call all patients with neurological signs or symptoms, including muscle weakness, gait disturbance, tremor, bradykinesia, or cognitive impairment (CI), in each village. The patients who were called also volunteered to participate. Indonesian collaborators and the co-authors (an English teacher in Bade Senior High School and Indonesian neurologist IM, and staff from Cenderawasih University) who spoke both English and Indonesian; local people who spoke both Indonesian and the local language in each

For peer review only - http://bmjopen.bmj.com/site/about/guidelines.xhtml

village; and co-author EG, who spoke English, Indonesian, and Japanese, cooperatively served as translators. Written informed consent was obtained from all the study participants. For the participants who could not read/write, verbal communication was carried out with the support of their family members and the translators. All the participants agreed to undergo neurological examinations, and none of the patients declined annual re-examination. The survey was approved by the ethics committees of the Research Institute for Humanity and Nature in Japan and Cenderawasih University in Papua, Indonesia.

Among the many types of neurological and non-neurological cases encountered, diagnoses of ALS, parkinsonism, and CI were based on clinical examinations and discussions among the participating neurologists. ALS was classified as ‘definite’, ‘probable’, ‘possible’, and ‘suspected’ on the basis of combinations of upper motor neuron (UMN) and lower motor neuron (LMN) signs according to the El Escorial Criteria of the World Federation of Neurology.[13] Probable and definite ALS are defined as pure ALS in this report. A diagnosis of parkinsonism required the presence of at least 2 of the following 4 signs: tremor, rigidity, bradykinesia, and postural impairment with a progressive course without evidence/history of vascular accidents or history of taking drugs known to induce parkinsonism. The degree of disability of patients with parkinsonism was classified according to the scale of Hoehn and Yahr.[14] Overlap of possible, probable, or definite ALS with parkinsonism was defined as ALS-parkinsonism. CI was diagnosed when loss of memory or impairments of language usage, praxis, or executive functions were identified during interview and clinical examination by neurologists. As cognitive functional test such as the mini–mental state

For peer review only

examination was not performed for all of the participants, patients with complications of functional disabilities attributable to CI in activities of daily living or in their livelihood were screened during the interview of both the patients and their family members. PMR was evaluated using the definition of Gajdusek et al. that PMR is a subacute paralytic condition reminiscent of the Landry-Guillain-Barre syndrome, which combines radicular, or perhaps neuritic elements with more acute onset and sometimes asymmetric paralysis.[1,2] Neurological cases with overlapping cerebellar signs (2 cases) were excluded from this study.

In Bade and the villages along the Ia, Edera, and Dumut rivers, not only were new cases were identified but also previously diagnosed cases were followed up and assessed neurologically and documented in 2007, 2008, 2010, 2011, and 2012. Death years were confirmed, and the durations between subjective onset of illness and the last survey or death were recorded as durations of subjective illness.

The size of the populations of Bade and the villages along the Ia, Edera, and Dumut rivers were determined from a report by the public office of Edera district in 2006.[15] We were informed by the village leaders of the populations of other villages in the survey for the period 2001–2002.

RESULTS

Clinical types of ALS and/or parkinsonism

We identified 46 cases with signs of ALS and/or parkinsonism, including 17 cases with pure ALS (Table 1), 13 with ALS-parkinsonism (Table 2), and 16 with parkinsonism

For peer review only - http://bmjopen.bmj.com/site/about/guidelines.xhtml

(Table 3). We found no case of pure or dominant dementia and PMR consistent with the definition by Gajdusek et al; similarly, no cases of PMR were found by Spencer et al. in 1987 or 1990. [10] Relevant information about the cases, including neurological signs, age at last survey, age and year of onset, disease duration, certified death year, and ethnicity, is shown in Tables 1–3. The male-to-female sex distribution was 10:7 (male, 59%) for ALS, 11:2 (85%) for ALS-parkinsonism, and 8:8 (50%) for parkinsonism. The mean age at last survey was 51.3 years for ALS, 52.9 years for ALS-parkinsonism, and 51.6 years for parkinsonism. The mean age at onset was 46.2 years (range, 11–68 years) for pure ALS, 49.5 years (11–70 years) for ALS-parkinsonism, and 45.8 years (27–64 years) for parkinsonism. The mean duration of subjective illness was 7.0 years (2–20 years) for pure ALS, 6.3 years (2–17 years) for ALS-parkinsonism, and 6.3 years (2–14 years) for parkinsonism. We observed overlapping CI in three (18%) of the 17 cases of pure ALS, 5 (38%) of the 13 cases of ALS-parkinsonism, and 1 (6%) of the 16 cases of parkinsonism.

During the follow-up surveys of the cases in Bade, Ia, and Edera rivers, we observed the following changes in diagnosis: 1 case, from possible ALS to probable ALS (case ALS10); 2 cases, from parkinsonism to ALS-parkinsonism (cases ALS-P 2 and ALS-P 3); and 1 case, from probable ALS to definite ALS (case ALS16) complicated with CI. We also identified an instance of a shared family history of parkinsonism (P 6) and ALS-parkinsonism (ALS-P 1). There were family histories of neurological disorders in 7 of 17 cases (41%) of pure ALS, 2 (15%) of 13 cases of ALS-parkinsonism, and 6 (38%) of 16 cases of parkinsonism (Tables 1–3).

For peer review only

Cases of ALS and parkinsonism in Bade and the villages along the Ia, Edera, and Dumut rivers between 2007 and 2012

Between 2007 and 2012, we identified 4 cases of pure ALS, 2 cases of ALS-parkinsonism, and 1 case of parkinsonism in Bade (population, 3800 people). In addition, we found 7 cases of pure ALS, 4 cases of ALS-parkinsonism, and three cases of parkinsonism in the villages (population, 4500 people) along the Ia river. Between 2008 and 2012, we identified three cases of pure ALS, 5 cases of ALS-parkinsonism, and 6 cases of parkinsonism in the villages (population, 4100 people) along the Edera and Dumut rivers (Table 4).

In the 2007 survey, there were 4 active cases of pure ALS (crude point prevalence rate, 105 [2–208]/100,000 people), 2 cases of ALS-parkinsonism (53 [0–126]/100,000 people), and 1 case of parkinsonism (26 [0–78]/100,000 people) in Bade. In 2010, there were 6 active cases of pure ALS (133 [27–240]/100,000 people), 4 cases of ALS-parkinsonism (89 [2–176]/100,000 people), and three cases of parkinsonism (67 [0–142]/100,000 people) in the villages along the Ia River. In 2010, there were three active cases of pure ALS (73 [0–156]/100,000 people), 4 cases of ALS-parkinsonism (98 [2–193]/100,000 people), and 5 cases of parkinsonism (122 [15-229]/100,000 people) in the villages along the Edera and Dumut rivers (Table 4).

In the survey during the period 2007–2012, the number of each clinical type (ALS, ALS-parkinsonism, and parkinsonism) was shown respectively for Bade (4, 4, and 1); the villages along the Ia River, namely Ogorito (1,2, and 0), Bosma (1, 1, and 0), Homlikya (1, 1, and 1), Gimikya (1, 1, and 0), Geturki (1, 0, and 0), Asset (1, 0, and 1),

and Osso (1, 0, and 1); and the villages along the Edera and Dumut rivers, namely Yibin (1, 1, and 0), Sien (0, 1, and 2), Pies (1, 0, and 1), Benggo (0, 1, and 0), Harapan (0, 0, and 1), Mopio (0, 0, and 1), Memes (0, 0, and 1), Muya (1, 0, and 0), and Getio (0, 1, and 0).

For peer review only

Case reports

Case ALS 12

A 64-year-old (in 2012) man of the Auyu tribe, a resident of Bosma along the Ia River, had a history of progressive gait disturbance and motor weakness for 9 years in 2010; these symptoms were found to have progressed based on the findings from the follow-up check-ups between 2011 and 2012. Neurological examination revealed UMN and LMN signs, including a positive Babinski sign, hyperreflexia with ankle clonus, muscle atrophy, and weakness in the tongue and upper and lower limbs bilaterally, and fasciculation in the upper limbs and tongue. He had no CI or sensory disturbance. We diagnosed him as having definite ALS (Figure 2-A–D). The subject had ALS, and his brother reported that their father showed tremor and gait disturbance, which were interpreted to be consistent with parkinsonism.

Case ALS-CI 17

A 70-year-old (in 2012) man of the Auyu tribe, a resident of Ogorito along the Ia River, had a history of progressive memory loss for 4 years and slow gait for 2 years. Neurological examination revealed UMN and LMN signs, including a positive Babinski sign, hyperreflexia in the jaw jerk, bilateral upper and lower limbs, and muscle atrophy,

For peer review only - http://bmjopen.bmj.com/site/about/guidelines.xhtml

weakness, and fasciculation in the distal upper and lower limbs bilaterally. He had fasciculation in the tongue and respiratory muscle weakness with a low oxygen saturation level (SpO_2, 86%). He also had memory disturbances accompanied by positive snout and palmomental reflexes and difficulty with mental activities of daily living. He had no sensory disturbance. We diagnosed him as having definite ALS with CI (Figure 3A).

Case ALS-P 1

A 65-year-old (in 2008) woman of the Auyu tribe, a resident of Pies along the Edera River, had a history of progressive motor weakness and gait disturbance for 5 years. She could not walk without support. Neurological examination revealed a resting tremor, cogwheel rigidity in the upper and lower limbs, bradykinesia throughout, postural impairment, UMN and LMN signs with a positive Babinski sign, hyperreflexia in the upper and lower limbs bilaterally, and thenar and hypothenar muscle atrophies. She had no CI or sensory disturbance. We diagnosed her as having ALS-parkinsonism and, more specifically, with severe parkinsonism (Hoen and Yahr IV) simultaneously complicated by motor neuron signs indicative of possible ALS. She died in 2009. Her younger sister was case P 6. The subject had ALS-parkinsonism, and her sister reported that their cousin showed tremor and gait disturbance, which were interpreted to be consistent with parkinsonism (Figure 3B).

Case ALS-P 2

A 76-year-old (in 2011) man of the Auyu tribe, a resident of Ogorito, had a history of tremor in both hands for 5 years in 2010. Neurological examination revealed cogwheel

For peer review only

For peer review only - http://bmjopen.bmj.com/site/about/guidelines.xhtml

rigidity in his upper and lower limbs, bradykinesia, and UMN signs with hyperreflexia in bilateral upper and lower limbs. We diagnosed him as having parkinsonism (Hoen and Yahr II) in 2010. He was examined again in 2011, by which time his parkinsonism was complicated by not only postural impairment but also muscle weakness and atrophy, and spasticity of the upper and lower limbs with Babinski signs. He had no CI or sensory disturbance. We diagnosed him as having parkinsonism (Hoen and Yahr III) with possible ALS; parkinsonism was accompanied by ALS later. He died in 2011 of unknown causes.

We also diagnosed another case of ALS-P3 parkinsonism (Hoen and Yahr III) with possible ALS; parkinsonism was accompanied by ALS later.

Case ALS-P-CI 6

A 50-year-old (in 2012) man of the Auyu tribe, a resident of Benggo along the Edera River, had a history of dysarthria for 2 years. Neurological examination revealed UMN and LMN signs with a positive Babinski sign, muscle atrophy, and weakness in the tongue, proximal upper limb, shoulder, and tibialis anterior muscles. He had cogwheel rigidity in the wrist and elbow, bradykinesia throughout, a positive Myerson sign, and impairment of coordination and postural reflex. He had memory loss, bradyphrenia, slow speech, and positive snout and palmomental reflexes with difficulty performing activities of daily living. We diagnosed him as having a definite ALS and parkinsonism (Hoen and Yahr III) with CI (Figure 3C).

Case P 7

For peer review only - http://bmjopen.bmj.com/site/about/guidelines.xhtml

A 52-year-old (in 2010) woman of the Auyu tribe who lived in Mopio along the Edera River had a history of progressive gait disturbance with parkinsonian signs, including resting tremor, cogwheel rigidity, anterior bending, and bradykinesia for 3 years in 2008. In 2008, we diagnosed her as having parkinsonism (Hoen and Yahr II). By the time of the follow-up survey in 2010, she could not stand by herself and had postural impairment with parkinsonian hands bilaterally. She had no UMN or LMN signs, no CI, and no sensory disturbance. We diagnosed her as having parkinsonism (Hoen and Yahr IV; Figure 3D).

DISCUSSION

Internal migration and migration from outside the province are the most significant causes of demographic, social, and cultural change in Papua. The populations in the survey sites were composed of former hunter-gatherers who lived in the forest and who were brought out of the forest and assembled in villages by Dutch colonialists. The villages along the Ia River were founded in 1937–1951.[10] The emigration program called transmigration (*transmigrasi*) started in Irian Jaya in 1964. Local migrants and transmigrants from outside Irian Jaya increased, resulting in various changes, including the use of food and medicine.[16,17] Considering that Gadjusek et al. found out the high prevalence of ALS at the first survey in 1962, the neurodegenerative disease existed before the introduction of manufactured items.[1]

In the present survey, we performed full neurological examinations in 46 subjects with ALS and/or parkinsonism between 2001 and 2012. Among the subjects, 17 were probable-definite cases of pure ALS, 13 were overlapping ALS (possible, probable, or

For peer review only - http://bmjopen.bmj.com/site/about/guidelines.xhtml

definite) and parkinsonism, and 16 were parkinsonism. Nine cases of ALS and/or parkinsonism had a complication of CI. During the study period, the diagnosis in some patients changed from pure parkinsonism to ALS-parkinsonism, and their disease progressed in severity. Some subjects had both ALS and parkinsonism on their first examination. It is thus evident that overlapping ALS and parkinsonism with or without CI still exists in the specified region of Papua.

Gajdusek et al. reported diagnosing 97 cases (57 cases clinically confirmed and 40 cases not clinically confirmed) of ALS during the 6 surveys they conducted from 1974 to 1981.[1] However, they reported neurological findings of only 32 of these cases, in only 16 of which the reported findings of UMN, LMN, and parkinsonian signs justify a diagnosis of probable or definite ALS. The 16 cases comprise 12 cases of pure ALS and 4 cases of overlapping ALS-parkinsonism. Among the 32 ALS patients, three had a positive family history (9%); that is, 2 had sisters with ALS, and 1 who had ALS-parkinsonism had a family history of ALS. None of the ALS patients had a family history of parkinsonism. The authors also reported their neurological findings in 13 subjects in whom parkinsonism was diagnosed. They reported their findings from full neurological examinations of 10 of these parkinsonism cases; according to their reported neurological signs, these comprised 8 patients of parkinsonism, including 5 with dementia and 2 with overlapping ALS-parkinsonism with dementia.[1] Among the 13 parkinsonism patients, three had a positive family history (23%). In summary, we believe that in the 26 cases reported by Gajdusek et al., the full neurological examination findings comprised 12 pure ALS cases (probable or definite), 6

For peer review only - http://bmjopen.bmj.com/site/about/guidelines.xhtml

overlapping ALS-parkinsonism, and 8 parkinsonism with/without dementia.[1] Spencer et al reported 2 cases of ALS and three cases of parkinsonism, including 1 with dementia, in the survey in 1987. He also reported 3 subjects with parkinsonism overlapping UMN signs, including 1 with dementia in 1990. We have here reported 17 cases of pure ALS (probable or definite), 13 cases of overlapping ALS-parkinsonism, and 16 cases of parkinsonism with or without CI in the 46 cases we neurologically examined fully. Thus, we identified many cases of overlapping ALS and parkinsonism (n = 13, 28%) like that observed in previous reports (n = 6, 23% and n = 3, 38%).[1, 10] We also identified many cases with CI (n = 9, 20%) like that observed in previous reports (n = 7, 27% and n = 2, 25%).[1, 10] Functional disabilities attributable to CI were recognized in activities of daily living or in their livelihood in all the cases with CI in this report.

Gajdusek et al. reported 13 cases of ALS and three cases of parkinsonism in 1975 in the villages along the Ia River (population, 2000).[1] The 13 ALS cases consisted of 10 clinically confirmed ones and 3 others that were not clinically confirmed. We compared the prevalence of probable-definite ALS and parkinsonism according to the report of Gajdusek et al. with our more recent findings. A crude point prevalence of ALS consistent with clinically probable to definite ALS (El Escorial criteria) may be estimated between 500 (95% confidence interval, 191–809/100,000 people) and 650 (298–1002) and that of parkinsonism was 150 (0–320/100,000 people) in 1975 along the Ia River. In our 2010 survey (population, 4500), we identified 6 probable-definite cases of ALS in this region (ALS 8, 10–13, and 17; 133 [27–-240]/100,000 people) and 7 of parkinsonism (possible ALS-P 2, 3, 9, and 13, and P 10, 15, and 16; 156 [40–

For peer review only - http://bmjopen.bmj.com/site/about/guidelines.xhtml

271]/100,000 people).

Gajdusek et al. reported 8 cases of ALS and 6 cases of parkinsonism in 1975 in the villages along the Edera and Dumut rivers (population, 1950). The 8 cases of ALS consisted of 7 clinically confirmed cases and 1 unconfirmed case. The crude point prevalence of ALS consistent with clinically probable to definite ALS (El Escorial criteria) may be estimated between 359 (94–624)/100,000 people and 410 (127–694)/100,000 people, and that of parkinsonism was 308 (62–554)/100,000 people in 1975 along the Edera and Dumut rivers. In our survey of this region in 2010 (population, 4100), we identified 5 probable-definite cases of ALS (ALS 9, 14, and 16, and ALS-P 5, 6; 122 [15–229]/100,000 people) and 7 cases of parkinsonism (possible ALS-P 10 and 11, and P 7, 8, and 11–13; 171 [44–297]/100,000 people; Table 4). As this was not a complete inventory survey, the actual prevalence may be a little higher than our estimation.

The population of Bade increased by a factor of 4 during 31 years (from 1000 in 1975 to 3800 in 2006), and those of villages along the Ia River (from 2000 to 4500) and the Edera and Dumut rivers (from 1950 to 4100) have increased 2-fold.[1,15] The subjects born outside Papua were only 2 (Maluku) among the 46 subjects with ALS and/or parkinsonism with/without CI in this survey, ie 44 were of Papuan genetic stock and 2 (from Maluku) were non-Papuan. The prevalence of the diseases among the local Papuan residents in this survey may be underestimated compared with the former report after taking into account the increase in the numbers of local migrants inside and transmigrants from outside Papua.[16,17,18]

For peer review only - http://bmjopen.bmj.com/site/about/guidelines.xhtml

As the median age and life expectancy in the field sites in Papua are estimated to be lower than the global median and fewer people may live beyond the age of 40 or 50 years,[18,19] the prevalence of the diseases in this survey may be underestimated compared with the global mean. As the median age and life expectancy in 1975 may be lower than those in 2010,[20] the prevalence of the diseases in the report by Gajdusek et al. might be underestimated compared with that in our survey.

The prevalence of ALS in this report was found to be three times lower than found by Gajdusek et al. Even after taking into account the change of population and age structure in the field sites, our findings show that the prevalence of ALS appears to have decreased over the past ~30-35 years since the report of Gajdusek et al.[10] This is consistent with the previous report by Spencer et al. However, it is still much higher than the global mean (incidence, approximately 2/100,000; prevalence, approximately 6/100,000) [21-26]; in particular, the prevalence of overlapping ALS and parkinsonism is especially high in Papua.

In the report by Gajdusek et al., who provided village-by-village data showing the number of cases of ALS and parkinsonism, there were many cases in the remote villages such as Bosma, Pies, Homlikya, Yibin, Sien, and Ogorite, which were less visited and least developed.[1, 2] In this survey, many cases (three ALS/ALS-parkinsonism) were found in the remotest village of Ogorito along the Ia River. Two cases of ALS/ALS-parkinsonism were also found in Bosma, Homlikya, and Yibin; 1 ALS and 2 cases of parkinsonism in Sien; 1 ALS and 1 parkinsonism in Pies, which consisted also of remote villages. While there were 8 cases of

For peer review only - http://bmjopen.bmj.com/site/about/guidelines.xhtml

ALS/ALS-parkinsonism in Bade and 2 in Gimikya, nearest to the port of the Digul River, none was reported by Gajdusek et al. in those sites. This increase may be brought about by local migrants from the affected villages.

Overlap of ALS-parkinsonism and dementia has also been reported in patients with ALS/PDC in Guam and Kii, Japan.[4, 27-33] Hirano et al. provided clinicopathological evidence of 47 cases of PDC in Guam in 1961, comprising the following 3 groups: (1) 30 cases of PDC; (2) 8 cases of parkinsonism with dementia with clinical evidence of UMN impairment; and (3) 9 cases of parkinsonism with dementia with clinical evidence of UMN and LMN impairment. At least 15 of these cases (31.9%) had a family history of ALS, parkinsonism, or both.[4] Kuzuhara and Kokubo et al. surveyed neurological diseases in Kii, Japan, in the 1990s and found not only a continuously high incidence of ALS but also neuropathologically verified cases of PDC. ALS and PDC frequently affected the same individuals simultaneously and the members of the same family. These authors reported 37 cases of ALS/PDC between 1996 and 2006, a high proportion of whom (78%) had a positive family history, as well as clinical examples of pure ALS, PDC, and overlapping ALS and PDC. They also reported neuropathological changes common to cases of ALS and PDC in 12 cases of ALS/PDC, resembling those found in patients with ALS/PDC in Guam [28-33] In our more recent study in Papua, such as the previous studies in Kii, Japan, and Guam, we identified many cases of pure ALS and ALS overlapping with parkinsonism with/without CI. The prevalence of CI was lower in our study sites than in Guam and Kii.[4, 27-33] CI might have been underestimated, and mild CIs or some mild dementia might have been overlooked by our neurological examinations in the field.

For peer review only - http://bmjopen.bmj.com/site/about/guidelines.xhtml

Our study was based only on clinical findings, and there are the limitations of not having electromyogram data, DNA analysis data, or any autopsy data from this population. Without such data, it is difficult to be certain how these patients fit into sporadic ALS or Parkinson disease and how they compare with ALS/PDC on Guam and in the Kii peninsula. However, we did identify many patients with overlapping ALS-parkinsonism in this study. The overlap of ALS and/or parkinsonism with CI suggests that it may be the same disease entity as the ALS/PDC diagnosed in Guam and Kii.

The proportion of males in the populations of Bade, the villages along the Ia River, and the villages along the Edera and Dumut rivers were 53%, 51%, and 50%. The proportion of males was high especially among the subjects with ALS-parkinsonism (85%) and ALS (59%) compared with among those with pure parkinsonism (50%) in our report. The proportion of males was higher among the subjects with ALS (63%) and parkinsonism (76%) in the report by Gadjusek et al., in which there were 3 male cases (75%) among the 4 ALS-parkinsonism cases. The high risks for ALS and PDC among the males were recognized in Guam and Kii.[1,7,27]

Indigenous people of Papua still retain aspects of their traditional lifestyles, such as hunting, gathering, fishing, and eating *sago* (trunk of *Metroxylon* sp.). They also drink water from shallow wells. However, with the spread of a market economy, recent increases in the numbers of rubber plantations and progress in transportation have been causing lifestyle changes in the villages in the south coastal area of Papua. As the diseases are declining as in Guam and Kii, the disease aetiology in Papua may be dominated by environmental factors. Genetic, culture-related and/or family-specific

For peer review only - http://bmjopen.bmj.com/site/about/guidelines.xhtml

factors may also play a role as evidenced by the family history of disease in 40% of the subjects in this survey in Papua and 80% in Kii.

Various possible causal genetic and environmental factors for these conditions have been reported. The environmental factors of low concentrations of calcium and magnesium in drinking water were hypothesized. [1,34] There is a recent report showing an association between the increase in ALS incidence and the change in drinking water source in the Kii Peninsula.[35] Spencer et al. pointed out that this explanation was inconsistent with a declining prevalence of ALS in a sessile population dependent on an unchanging supply of river water, which this report may support.[10]

Spencer et al. reported the discovery that cycad seed was used as a poultice in Papua, as in Guam. [36] Cycad-derived products were shown as the risk factor of dementia, mild CI, and PDC in Guam with the epidemiological evidence.[37] Although cycad had been suspected as the causal factor of Kii ALS,[38] people in Japan, including the Kii Peninsula, had never consumed cycad as daily food, although a small amount of cycad flour might have been taken by a few people as an ingredient of herb medicine.[39] We did not examine the association of prior skin wounds and exposure to cycad poultice with the diseases. Skin change and pigmentary retinopathy were not examined in this survey.[40, 41] Further studies are needed.

Genetic abnormalities that had been previously established to be associated with familial or sporadic ALS, dementia, familial Parkinson disease, and parkinsonism were looked for in the cases in Kii, but none of them were identified.[28,42] Hermousura et

For peer review only - http://bmjopen.bmj.com/site/about/guidelines.xhtml

al. have discovered a nuclear mutation in the calcium/magnesium membrane ion channel transient receptor potential melastatin 7 (*TRPM7*) gene in a subset of patients with ALS/PDC in Guam.[43] They reported that a TRPM7 variant that is associated with altered sensitivity to magnesium may have contributed to the pathogenesis of ALS and PDC in 2 Guamanians. *TRPM7* was not found to be associated with ALS/PDC in the Kii peninsula of Japan.[44] Ishiura et al. have recently discovered *C9ORF72* repeat expansion in some cases of ALS in the Kii peninsula of Japan; this repeat expansion partly accounts for the high prevalence of ALS in this region.[45] However, the responsible gene(s) remain unproved in almost all cases of ALS/PDC in both Guam and Kii.

Various environmental factors and multiple genetic factors are suggested to be underlying causes of these diseases, but these links are so far unproven.[33,46]

In Guam, although the incidence of pure ALS has declined markedly, the prevalence of PDC is still high.[47] The prevalence of dementia among elderly Chamorros (earliest known inhabitants of the Mariana Islands, which include Guam) is also reportedly relatively high.[48] In addition, in Kii the incidence of pure ALS has declined markedly, and the prevalence of PDC and ALS/PDC or ALS with dementia recently increased.[30,33] These changing patterns of ALS/PDC incidence in Guam and Kii might be caused by changes in environmental and socioeconomic factors as well as aging.[48,49]

The mean age of onset in our cases was 46 years for ALS, 53 years for ALS-parkinsonism, and 46 years for parkinsonism. Thus, they were older than the

For peer review only - http://bmjopen.bmj.com/site/about/guidelines.xhtml

previously reported cases of ALS (33 years) and parkinsonism (43 years).[1] In our cases, the mean duration of subjective illness was 7.0 years for ALS, 6.3 years for ALS-parkinsonism, and 6.3 years for parkinsonism. The duration of ALS in our data was longer than not only the world mean of 3 years[21-26] but also the previously reported 3.5 years for ALS in Papua.[1] The mean age of onset may recently have increased, and the mean disease duration became longer in subjects with ALS. The prevalence rate of cases with bulbar sign was 37% in 30 of pure ALS or ALS-parkinsonian in our report, and it was as the same as in the report of Gadjdusek (40%) and classical ALS (39%).[2] The change in onset and course of ALS in recent years might be attributable to the aging population and changing environmental and socioeconomic factors.

On-going follow-up surveys should be performed to evaluate changes in the clinical types of ALS and/or parkinsonism with or without CI and accompanying changes in environmental and socioeconomic factors, as have occurred in Guam and Kii.

In conclusion, the prevalence of ALS in the Papuan disease focus has decreased over the past ~30-35 years but, in 2010, it remained higher than the global average. During this period, there has been an influx of migrants from inside and transmigrants from outside Papua that has increased the size and changed the composition of the population in the villages studied. There may be the possible impact of the expanded population on prevalence estimates. There are still many cases of pure ALS and overlapping ALS-parkinsonism and CI in Papua, as has previously been reported in Guam and Kii. The disease continues to impact native Papuans but may also affect a small number of

For peer review only - http://bmjopen.bmj.com/site/about/guidelines.xhtml

non-Papuan immigrants (2 of 46 or 3-4%) who adopt the local lifestyle. Environmental factors may play important etiologic role. Further on-going follow-up surveys focusing on the clinical epidemiology and aetiology of these neurodegenerative diseases in association with ecological and environmental factors in Papua are needed. Future studies, including neuropathological examinations, should be performed when the local villagers in this region of Papua, Indonesia, are willing to consent to autopsies.

ACKNOWLEDGMENTS

The authors wish to thank all the participants in the villages who volunteered for the survey, the village leaders, and Mappi Regency officials. We are deeply indebted to doctors Imelda Mandagi, Reginald Nangoy, and Christha Z. Tamburian and their staff at Puskesmas Bade, and to Arifin Wasaraka, Lazarus Revasy, Yosefina Griapon, Arius Togodly, Kerry Yarangga, and Dolfinus Y. Bouway at Cenderawasih University for their invaluable contributions to the study. Andre Liem and Herry Wondiwoy at Papua Tour Guides Community and S.E. Amos at Bade High School provided logistic and language assistance. On the Japanese side, we acknowledge the technical support and advice of Katsuyuki Eguchi (Nagasaki University), Yasushi Osaki (Kochi University), Hidekazu Tomimoto (Mie University), Taro Yamauchi (The University of Tokyo), Hideyuki Onishi (Doshisha Women's College), Tetsuya Inamura (Aichi Prefectural University), Wenling Chen, Yumi Kimura, Yasuko Ishimoto, and Hissei Imai (Kyoto University), and Yasuyuki Kosaka, Yukiko Kita, and Chizu Wada (Research Institute for Humanity and Nature).

For peer review only - http://bmjopen.bmj.com/site/about/guidelines.xhtml

AUTHOR CONTRIBUTIONS

Okumiya had full access to all the data in the study and takes responsibility for the integrity of the data and the accuracy of the data analysis.

Study concept and design: Okumiya, Matsubayashi.

Acquisition of data: Okumiya, Wada, Fujisawa, Ishine, Garcia del Saz Hirata, Kuzuhara, Kokubo, Seguchi, Sakamoto, Manuaba, Matsubayashi.

Analysis and interpretation of data: Okumiya,Wada, Sakamoto, Fujisawa, Matusbayashi.

Drafting of the manuscript: Okumiya,Wada, Sakamoto, Fujisawa, Matusbayashi.

Critical revision of the manuscript for important intellectual content: All authors.

Statistical analysis: Okumiya.

Obtained funding: Okumiya, Wada, Kuzuhara, Kokubo, Matsubayashi

Administrative, technical, or material support: Okumiya, Watofa, Rantetampang, Matsubayashi.

Study supervision: Okumiya, Matsubayashi.

COMPETING INTERESTS : None declared

For peer review only

FUNDING

K. Okumiya receives research support from Grant-in-Aid for Scientific Research (A) from the Japan Society for the Promotion of Science (JSPS). K. Matsubayashi receives research support from Grant-in-Aid for Scientific Research (A) from the JSPS and is an editor of the journal *Geriatrics & Gerontology International*. T. Wada receives research

For peer review only - http://bmjopen.bmj.com/site/about/guidelines.xhtml

support from Grant-in-Aid for Scientific Research (C) from the JSPS. Y. Kokubo and S. Kuzuhara receive support from Grants-in-Aid from the Research Committee of CNS Degenerative Diseases and the Research Committee on the Muro disease (Kii amyotrophic lateral sclerosis/Parkinsonism-dementia), the Ministry of Health, Labour and Welfare of Japan.

For peer review only

REFERENCES

1. Gajdusek DC, Salazar AM. Amyotrophic lateral sclerosis and parkinsonian syndromes in high incidence among the Auyu and Jakai people of West New Guinea. Neurology 1982;**32**:107–26.
2. Gajdusek DC. Foci of motor neuron disease in high incidence in isolated populations of East Asia and the Western Pacific. In: Lewis P Rowland, ed. Human Motor Neuron Diseases. New York: Raven Press 1982:363–93.
3. Kurland LT, Mulder DW. Epidemiologic investigations of amyotrophic lateral sclerosis. 1. Preliminary report on geographic distribution and special reference to the Mariana Islands, including clinical and pathologic observations. Neurology 1954;**4**:438–48.
4. Hirano A, Kurland LT, Krooth RS, et al. Parkinsonism-dementia complex, an endemic disease on the island of Guam. I. Clinical features. Brain 1961;**84**:642–61.
5. Hirano A, Malamud N, Kurland LT. Parkinsonism-dementia complex, an endemic disease on the island of Guam. II. Pathological features. Brain 1961;**84**:662–79.
6. Kimura K, Yase Y, Higashi Y, et al. Epidemiological and geomedical studies on ALS and allied diseases in Kii peninsula (Japan): preliminary report. Proc Jpn Acad

For peer review only - http://bmjopen.bmj.com/site/about/guidelines.xhtml

1961;**37**:417–20.

7. Shiraki H, Yase Y. Amyotrophic lateral sclerosis in Japan. In: Vinken PJ, Bruyn GW, eds. Handbook of clinical neurology Vol. 22, System disorders and atrophies, Part II. Amsterdam: Elsevier Science 1975:353–419.
8. Garruto RM, Yanagihara R, Gajdusek DC. Disappearance of high incidence ALS and PDC on Guam. Neurology 1985;**35**:193–8.
9. Yoshida S. ALS tahatsuchi niokeru kankyoyoin to aluminium no neuron hensei eno kanyo nitsuite.[Environmental factors in western Pacific foci of ALS and a possible pathogenetic role of aluminum (Al) in motor neuron degeneration] (in Japanese with English abstract). Rinsho Shinkeigaku [Clin Neurol] 1991;**31**:1310–2.
10. Spencer P, Palmer VS, Ludolph AC. On the decline and etiology of high-incidence motor system disease in West Papua (Southwest New Guinea). Mov Disord 2005;**20(**Suppl 12):S119–126.
11. Wada T, Matsubayashi K, Okumiya K, et al. Health status and subjective economic satisfaction in West Papua. Lancet 2002;**360**:951.
12. Okumiya K, Fujisawa M, Ishine M, et al. Nishi New Guinea chiiki (Indonesia/ Papua shu) no shinkeihensei shikkan no jittai. 2001-2002 nen, 2006-2007 nen no fieldwork yori. [Fieldwork survey of neurodegenerative diseases in West New-Guinea in 2001-02 and 2006-07] (in Japanese). Rinsho Shinkeigaku [Clin Neurol] 2007;**47**:977–8.
13. Brooks BR. El Escorial World Federation of Neurology criteria for the diagnosis of amyotrophic lateral sclerosis: subcommittee on Motor Neuron Diseases/Amyotrophic Lateral Sclerosis of the World Federation of Neurology Research Group on Neuromuscular Diseases and the El Escorial “Clinical Limits of

For peer review only - http://bmjopen.bmj.com/site/about/guidelines.xhtml

ALS" Workshop Contributors. J Neurol Sci 1994;**124**:96–107.

14. Hoehn MM, Yahr MD. Parkinsonism: onset, progression and mortality. Neurology. 1967;**17**:427–42.
15. Kontor Statistik Distrik Edera. Laporan Kependudukan, Kabupaten Mappi Distrik Edera 2006 (December).
16. Manning C., Rumiak M. Irian Jaya: Economic change, migrants, and indinenous welfare. In Hill H ed. Unity and diversity: Regional Economic Development in Indonesia since 1970. Oxford University Press, Okford.p77-106, 1991.
17. Timmer J. A brief social and political history of Papua, 1962-2005. In Marshall AJ and Beehler BM ed. The Ecology of Indonesian Papua Part Two. Periplus Editions, Hong Kong. p1098-1124, 2007.
18. Badan Pusat Statistik, Sensus Penduduk 2010. http://sp2010.bps.go.id/index.php
19. United Nations , Department of Economic and Social Affairs, Population Division. World Population Prospects The 2012 Revision *Volume II: Demographic Profiles,* http://esa.un.org/unpd/wpp/index.htm
20. Kontor Statistik Kabupaten Merauke. Laporan Kependudukan, Kabupaten Merauke 1997
21. Armon C. Amyotrophic lateral sclerosis. In: Neuroepidemiology from principles to practice. USA: Oxford University Press 2004:162–87.
22. McGuire V, Longstreth WT Jr, Koepsell TD, et al. Incidence of amyotrophic lateral sclerosis in three counties in western Washington state. Neurology 1996 ;**47**:571–3.
23. Sorenson EJ, Stalker AP, Kurland LT, et al. Amyotrophic lateral sclerosis in Olmsted County, Minnesota, 1925 to 1998. Neurology 2002; **23**:280–2.
24. O'Toole O, Traynor BJ, Brennan P, et al. Epidemiology and clinical features of

For peer review only - http://bmjopen.bmj.com/site/about/guidelines.xhtml

amyotrophic lateral sclerosis in Ireland between 1995 and 2004. J Neurol Neurosurg Psychiatry 2008;**79**:30–2.

25. Chiò A, Mora G, Calvo A, et al. Epidemiology of ALS in Italy: a 10-year prospective population-based study. Neurology 2009;**72**:725–31.
26. Factor-Litvak P, Al-Chalabi A, Ascherio A, et al. Current pathways for epidemiological research in amyotrophic lateral sclerosis. Amyotroph Lateral Scler Frontotemporal Degener 2013;**14**(Suppl 1):33–43.
27. Rodgers-Johnson P, Garruto RM, Yanigahara R, et al. Amyotrophic lateral sclerosis and parkinsonism-dementia on Guam: a 30-year evaluation of clinical and neurological trends. *Neurology.* 1986;36:7-13.
28. Kuzuhara S, Kokubo Y, Sasaki R, et al. Familial amyotrophic lateral sclerosis and parkinsonism-dementia complex of the Kii peninsula of Japan: clinical and neuropathological study and tau analysis. Ann Neurol 2001;**49**:501–11.
29. Kuzuhara S, Kokubo Y. Atypical parkinsonism of Japan: amyotrophic lateral sclerosis-parkinsonism-dementia complex of the Kii peninsula of Japan (Muro disease): an update. Mov Disord 2005;**20**(Suppl 12):S108–13.
30. Kuzuhara S. Kii ALS/PDC (Murou byou) no rekishi, gainen, ekigaku. [ALS-parkinsonism-dementia complex of the Kii peninsula of Japan (Muro disease). Historical review, epidemiology and concept] (in Japanese with English abstract). Rinsho Shinkeigaku [Clin Neurol] 2007;**47**:962–5.
31. Kokubo Y. Kiihanto no kinishukuseisokusakukokasho/ parkinson chihou hukugo no rinshou, shinkei byourigakuteki kenkyu. [Clinical aspects, imaging and neuropathology of Kii ALS/PDC] (in Japanese with English abstract). Rinsho Shinkeigaku [Clin Neurol] 2007;**47**:966–9.

For peer review only - http://bmjopen.bmj.com/site/about/guidelines.xhtml

32. Kuzuhara S. Kii hanto no Murobyo (Kinishukuseisokusakukokasho/ parkinson/ ninchisho hukugo) [Muro disease or ALS–parkinsonism–dementia complex of the Kii peninsula of Japan] (in Japanese with English abstract). Rinsho Shinkeigaku [Clin Neurol] 2007;**47**:695–702.

33. Kuzuhara S, KokuboY. Amyotrophic lateral sclerosis-parkinsonism-dementia complex in the Kii peninsula of Japan (Muro disease): a review on recent research and new concept. In: Michael J. Strong, ed. Amyotrophic lateral sclerosis and the frontotemporal dementias. Oxford: Oxford University Press 2012:39–54.

34. Yase Y. The pathogenesis of amyotrophic lateral sclerosis. Lancet 1972;**2**(7772):292–6.

35. Kihara T, Yoshida S, Kondo T et al. An increase in ALS incidence on the Kii Peninsula, 1960-2009: A possible link to change in drinking water source. *Amyotrophic Lateral Sclerosis,* 2012; 13: 347–50.

36. Spencer PS, Nunn PB, Hugon J, et al. Guam amyotrophic lateral sclerosis-parkinsonism-dementia linked to a plant excitant neurotoxin. Science 1987;**237**(4814):517–22.

37. Borenstein AR, Mortimer JA, Schofield E. et al. Cycad exposure and risk of dementia, MCI, and PDC in the Chamorro population of Guam. Neurology. 2007;68:1764-71.

38. Spencer PS, Ohta M, Palmer VS. Cycad use and motor neuron disease in Kii peninsula of Japan. Lancet 1987;**2**(8573):1462–3.

39. Iwami O, Niki Y, Watanabe T, and Ikeda M. Motor neuron disease on the Kii Peninsula of Japan: Cycad exposure. Neuroepidemiology 1993;12:307-312.

40. Ono S, Waring SC, Kurland L et al. Guamanian neurodegenerative disease: ultrastructural studies of skin J. Neurol. Sci., 146 (1997), pp. 35–40

For peer review only

41. Kokubo Y, Ito K, Kuzuhara S. Ophthalmomyiasis-like pigmentary retinopathy in ALS/PDC in the Kii peninsula of Japan. Neurology. 2003 May 27;60(10):1725-6.

42. Tomiyama H, Kokubo Y, Sasaki R, et al. Mutation analyses in amyotrophic lateral sclerosis/parkinsonism-dementia complex of the Kii peninsula, Japan. Mov Disord 2008;**23**(16):2344–8.

43. Hermosura MC, Nayakanti H, Dorovkov MV, et al. A TRPM7 variant shows altered sensitivity to magnesium that may contribute to the pathogenesis of two Guamanian neurodegenerative disorders. Proc Natl Acad Sci 2005;**102**(32):11510–5.

44. Hara K, Kokubo Y, Ishiura H, et al. TRPM7 is not associated with amyotrophic lateral sclerosis-parkinsonism dementia complex in the Kii peninsula of Japan. Am J Med Genet B Neuropsychiatr Genet 2010;**153B**(1):310–3.

45. Ishiura H, Takahashi Y, Mitsui J, et al. *C9ORF72* Repeat expansion in amyotrophic lateral sclerosis in the Kii peninsula of Japan. Arch Neurol 2012;**69**(9):1154–8.

46. Garruto RM, Yanagihara R. Contributions of isolated Pacific populations to understanding neurodegenerative diseases. Folia Neuropathol 2009;**47**(2):149–70.

47. Plato CC, Garruto RM, Galasko D, et al. Amyotrophic lateral sclerosis and parkinsonism-dementia complex of Guam: changing incidence rates during the past 60 years. Am J Epidemiol 2003;**157**:149–57.

48. Galasko D, Salmon D, Gamst A, et al. Prevalence of dementia in Chamorros on Guam: relationship to age, gender, education, and APOE. Neurology 2007;**68**:1772–81.

49. Plato CC, Galasko D, Garruto RM, et al. ALS and PDC of Guam: forty-year follow-up. Neurology 2002;**58**:765–73.

For peer review only - http://bmjopen.bmj.com/site/about/guidelines.xhtml

Figure and table legends

Table 1. Relevant characteristics and neurological signs of cases with ALS with/without cognitive impairment in Papua, 2001–2012.

Abbreviations: ALS = amyotrophic lateral sclerosis; BK = bradykinesia; BS = Babinski sign; DTR = deep tendon reflex; Dys = dysphagia/dysarthria; F= female; Fasc = fasciculation; LMN = lower motor neuron signs; M = male; MA = muscle atrophy with weakness; NC = not confirmed by examination; P = parkinsonism; PI = postural impairment; R = rigidity; T = tremor; TA = tongue atrophy; U/L: upper limbs/lower limbs; UMN = upper motor neuron signs.

Age: at the time of final survey; Duration: between subjective onset of illness and last survey or death; Alive: alive in 2012; Onset: subjective onset of the illness; Deep tendon reflexes, -: absent, 1: hypoactive, 2: normal, 3: hyperactive, 4: markedly hyperactive with ankle clonus.

Other signs are classified as: -: absent; ±: questionably present; +: present; ++: markedly present.

Family history 1: cousin (possible ALS); 2: grandfather (NC); 3: sister (ALS15); 4: cousin's daughter (P16); 5: cousin (NC); 6: father (NC); 7: sister (ALS 7); 8: sister (P 6) and cousin (NC); 9: father (NC); 10: son: (NC); 11: sister (ALS-P 1) and cousin (NC); 12: mother and father (NC); 13: grandmother (NC); 14: mother (NC); 15: ALS 10 (mother's cousin).

For peer review only - http://bmjopen.bmj.com/site/about/guidelines.xhtml

Table 2. Relevant characteristics and neurological signs in cases with overlap of ALS and parkinsonism with/without cognitive impairment in Papua, 2001–2012.

Table 3. Relevant characteristics and neurological signs in cases with parkinsonism with/without cognitive impairment in Papua, 2001–2012.

Table 4. Cases of ALS and parkinsonism according to field site

*; Active cases in 2007 in Bade

#; Active cases in 2010 in the villages along the Ia, Edera, and Dumut rivers

Figure 1. Map of the field sites

A. Large-scale map of the southern coastal plain in Papua, Indonesia.

B. Smaller scale map of the southern coastal plain in Papua, Indonesia showing rivers.

C. Detailed map showing villages in the southern coastal area in Papua in which all confirmed cases of ALS and/or parkinsonism with/without cognitive impairment were seen from 2001 to 2012.

Figure 2. Photographs of patient with ALS (Case ALS 12**)**

A: The patient needs assistance from family members to stand.

B: Advanced atrophy of the tongue.

C: There is upper limb-girdle and truncal muscle atrophy with a positive Babinski sign.

D: Advanced thenar muscle atrophy.

For peer review only - http://bmjopen.bmj.com/site/about/guidelines.xhtml

Figure 3. Photographs of patients with ALS, ALS-parkinsonism, and parkinsonism

A: Patient with ALS (Case ALS-17) showing muscle atrophy in the distal upper and lower limbs.

B: Patient with ALS-parkinsonism (Case ALS-P 1) showing muscle atrophy in distal upper limbs.

C: Patient with ALS-parkinsonism (Case ALS-P 7) showing atrophy in muscles in the proximal upper limb, shoulder, and tibialis anterior.

D: Patient with parkinsonism (Case P 7) showing typical parkinsonian hands and anterior bending posture.

Parkinsonian hand: Striatal deformities of the hand with abnormal postures that are common in patients with advanced Parkinson's disease.

For peer review only

For peer review only - http://bmjopen.bmj.com/site/about/guidelines.xhtml

Okumiya

Table 1. Relevant characteristics and neurological signs in cases with pure ALS with/without cognitive impairment in Papua, 2001–2012.

Case number	ALS criteria Yahr stage	Survey year	Village	Age	Sex	Onset (age/year)	Duration (years)	Death (year)	Tribe (origin)	UMN signs DTR U/L	UMN signs BS	LMN signs MA U/L	LMN signs Fasc U/L	Bulbar signs TA	Bulbar signs Dys	Parkinsonian sign T U/L	Parkinsonian sign R U/L	Parkinsonian sign BK	Parkinsonian sign PI	Cognitive impairment	Family history
Pure ALS (n=17)																					
ALS 1	Definite	2001	Bade	35	M	NC	NC	NC	Auyu	3/3	+	++/++	+/+	++	-	-	-	-	-	-	-
ALS 2	Definite	2001	Ogoto	43	F	NC	NC	2002	Auyu	3/3	+	++/++	+/+	++	++	-	-	-	-	-	-
ALS 3	Definite	2002	Senggo	50	M	48/'00	2	NC	Citak	3/3	+	++/++	±/±	±	+	-	-	-	-	-	1
ALS 4	Definite	2007	Homilikia	53	M	51/'05	2	2007	Auyu	3/2	+	++/++	+/+	-	-	-	-	-	-	-	-
ALS 5	Definite	2007/'08	Bade	51	M	49/'06	3	2009	Auyu	3/4	+	+/-	-/-	-	+	-	-	-	-	-	-
ALS 6	Definite	2008	Bade	21	M	11/'98	11	2009	Auyu	4/4	+	++/++	-/-	-	-	-	-	-	-	-	2
ALS 7	Definite	2007/'08/'10	Bade	70	F	57/'97	13	2010	Maluku	3/3	+	++/++	+/-	-	+	-	-	-	-	-	3
ALS 8	Probable	2011	Geturki	53	M	48/'06	5	Alive	Auyu	3/3	-	+/+	-/+	-	-	-	-	-	-	-	-
ALS 9	Definite	2011	Muya	41	F	36/'06	6	2012	Auyu	3/3	+	+/+	-/-	-	-	-	-	-	-	-	-
ALS 10	Probable	2007/'10/'11/'12	Osso	65	F	59/'06	6	Alive	Auyu	3/4	+	+/-	-/-	+	-	-	-	-	-	-	4
ALS 11	Probable	2007/'08/'10/'12	Gimikya	32	M	12/'92	20	Alive	Auyu	4/4	+	++/+	++/-	±	-	-	+/-	-	-	-	5
ALS 12	Definite	2010/'11/'12	Bosma	64	M	53/'01	11	Alive	Auyu	3/4	+	++/++	+/-	++	-	-	-	-	-	-	6
ALS 13	Definite	2012	Asset	70	F	68/'10	2	Alive	Auyu	3/3	-	+/+	-/-	-	-	-	-	-	-	-	-
ALS 14	Probable	2012	Pies	65	F	60/'07	5	Alive	Auyu	3/4	-	+/+	-/-	-	-	-	-	-	-	-	-
ALS 15	Definite	2007	Bade	64	F	62/'05	2	2007	Maluku	3/3	+	++/++	-/-	-	+	-	-	-	-	+	7
ALS 16	Definite	2008/'10/'11	Yibin	25	M	13/'99	13	2012	Auyu	3/4	+	++/+	++/++	-	-	-	-	-	-	+	-
ALS 17	Definite	2012	Ogorito	70	M	66/'08	4	Alive	Auyu	3/3	+	+/+	+/+	-	-	-	-	-	-	+	-

Abbreviations: ALS = amyotrophic lateral sclerosis; BK = bradykinesia; BS = Babinski sign; DTR = deep tendon reflex; Dys = dysphagia/dysarthria; F = female; Fasc = fasciculation; LMN = lower motor neuron signs; M = male; MA = muscle atrophy with weakness; NC = not confirmed by examination; P = parkinsonism; PI = postural impairment; R = rigidity; T = tremor; TA = tongue atrophy; U/L: upper limbs/lower limbs; UMN = upper motor neuron signs.

Age: at the time of final survey; Duration: between subjective onset of illness and last survey or death; Alive: alive in 2012; Onset: subjective onset of illness; Deep tendon reflexes, -: absent, 1: hypoactive, 2: normal, 3: hyperactive, 4: markedly hyperactive with ankle clonus.

Other signs are classified as: -: absent; ±: questionably present; +: present; ++: markedly present.

Family history 1: cousin (possible ALS); 2: grandfather (NC); 3: sister (ALS15); 4: cousin's daughter (P16); 5: cousin (NC); 6: father (NC); 7: sister (ALS 7); 8: sister (P 6) and cousin (NC); 9: father (NC); 10: son: (NC); 11: sister (ALS-P 1) and cousin (NC); 12: mother and father (NC); 13: grandmother (NC); 14: mother (NC); 15: ALS 10 (mother's cousin).

For peer review only

For peer review only - http://bmjopen.bmj.com/site/about/guidelines.xhtml

Okumiya

Table 2. Relevant characteristics and neurological signs in cases with overlap of ALS and parkinsonism with/without cognitive impairment in Papua, 2001–2012.

Case number	ALS criteria Yahr stage	Survey year	Village	Age	Sex	Onset (age/year)	Duration (year)	Death (year)	Tribe (origin)	UMN signs DTR U/L	UMN signs BS	LMN signs MA U/L	LMN signs Fasc U/L	Bulbar signs TA	Bulbar signs Dys	Parkinsonian sign T U/L	Parkinsonian sign R U/L	Parkinsonian sign BK	Parkinsonian sign PI	Cognitive impairment	Family history
ALS-parkinsonism (n = 13)																					
ALS-P 1	Possible-IV	2008	Pies	65	F	60/'03	6	2009	Auyu	3/3	+	+/-	-/-	-	-	+/+	++/++	++	+	-	8
ALS-P 2	Possible-III	2010/'11	Ogorito	76	M	70/'05	6	2011	Auyu	3/3	+	+/±	-/-	-	-	+/+	+/+	+	+	-	-
ALS-P 3	Possible-III	2010/'11/'12	Homilikia	69	M	65/'08	4	Alive	Auyu	3/2	+	+/-	-/-	-	-	+/+	+/+	+	+	-	-
ALS-P 4	Definite-III	2008	Bade	34	M	18/'92	17	2009	Muyu	3/3	+	+/-	-/-	++	++	-	+/+	+	+	+	9
ALS-P 5	Definite-III	2012	Sien	60	M	58/'10	2	Alive	Auyu	3/3	+	+/+	-/-	-	-	-	+/+	+	+	+	-
ALS-P 6	Definite-III	2012	Benggo	50	M	48/'10	2	Alive	Auyu	2/2	+	+/+	-/-	+	+	-	+/-	+	+	+	-
ALS-P 7	Possible-II	2001	Bade	36	M	NC	NC	NC	Auyu	3/3	+	-/-	-/-	-	-	+	+	+	-	-	-
ALS-P 8	Possible-II	2008/'10	Bade	23	M	11/'98	12	Alive	Auyu	3/4	+	-/-	-/-	-	-	+/+	+/±	+	-	-	-
ALS-P 9	Possible-III	2010/'11	Ogorito	76	M	70/'05	6	2011	Auyu	3/3	+	-/-	-/-	-	-	+/-	+/+	+	+	-	-
ALS-P 10	Possible-III	2008/'10/'11	Yibin	52	M	44/'03	8	Alive	Auyu	3/3	+	-/-	-/-	-	-	+/-	+/+	+	+	-	-
ALS-P 11	Possible-III	2011	Getio	55	F	53/09	2	2011	Auyu	3/3	+	±/-	-/-	-	-	+/+	+/+	+	+	-	-
ALS-P 12	Possible-IV	2001	Bade	40	M	NC	NC	NC	Auyu	3/3	+	-/-	-/-	-	+	+	+	+	+	+	-
ALS-P 13	Possible-III	2012	Bosma	52	M	48/'08	4	Alive	Auyu	3/4	+	-/-	-/-	-	-	-	++/+	+	+	+	-

Abbreviations: ALS = amyotrophic lateral sclerosis; BK = bradykinesia; BS = Babinski sign; DTR = deep tendon reflex; Dys = dysphagia/dysarthria; F = female; Fasc = fasciculation; LMN = lower motor neuron signs; M = male; MA = muscle atrophy with weakness; NC = not confirmed by examination; P = parkinsonism; PI = postural impairment; R = rigidity; T = tremor; TA = tongue atrophy; U/L: upper limbs/lower limbs; UMN = upper motor neuron signs.
Age: at the time of final survey; Duration: between subjective onset of illness and last survey or death; Alive: alive in 2012; Onset: subjective onset of illness; Deep tendon reflexes, -: absent, 1: hypoactive, 2: normal, 3: hyperactive, 4: markedly hyperactive with ankle clonus.
Other signs are classified as: -: absent; ±: questionably present; +: present; ++: markedly present.
Family history 1: cousin (possible ALS); 2: grandfather (NC); 3: sister (ALS15); 4: cousin's daughter (P16); 5: cousin (NC); 6: father (NC); 7: sister (ALS 7); 8: sister (P 6) and cousin (NC); 9: father (NC); 10: son: (NC); 11: sister (ALS-P 1) and cousin (NC); 12: mother and father (NC); 13: grandmother (NC); 14: mother (NC); 15: ALS 10 (mother's cousin).

For peer review only - http://bmjopen.bmj.com/site/about/guidelines.xhtml

Table 3. Relevant characteristics and neurological signs in cases with parkinsonism with/without cognitive impairment in Papua, 2001–2012.

Case number	ALS criteria Yahr stage	Survey year	Village	Age	Sex	Onset (age/year)	Duration (years.)	Death (year)	Ethnicity	UMN signs		LMN signs		Bulbar signs		Parkinsonian sign				Cognitive impairment	Family history
										DTR U/L	BS	MA U/L	Fasc U/L	TA	Dys	T U/L	R U/L	BK	PI		
Parkinsonism (n = 16)																					
P 1	Yahr IV	2001	Kepi	50	M	NC	NC	NC	Jakai	2/2	-	-/-	-/-	-	-	+/+	+/+	++	+	-	-
P 2	Yahr II	2001	Kepi	50	M	NC	NC	NC	Jakai	2/2	-	-/-	-/-	-	-	+	+	+	-	-	-
P 3	Yahr IV	2002	Emete	52	M	49/'99	3	NC	Jakai	2/2	-	-/-	-/-	-	-	+	+	+	+	-	-
P 4	Yahr II	2002	Senggo	41	M	36/'97	5	NC	Citak	2/2	-	-/-	-/-	-	-	+	+	-	-	-	-
P 5	Yahr I	2007	Mur	65	F	61/'03	4	NC	Jakai	2/3	-	-/-	-/-	-	-	+/-	+/-	-	-	-	10
P 6	Yahr IV	2008	Pies	60	F	55/'03	6	2009	Auyu	2/2	-	-/-	-/-	-	-	+/+	+/+	++	+	-	11
P 7	Yahr IV	2008/'10	Mopio	52	F	47/'05	5	Alive	Auyu	3/3	-	-/-	-/-	-	-	++/+	+/+	++	+	-	-
P 8	Yahr II	2011	Sien	57	F	49/03	9	2012	Auyu	3/3	-	-/-	-/-	-	-	+/+	+/+	+	-	-	12
P 9	Yahr IV	2007/'08/'10/'[illegible]	Bade	46	F	40/'06	6	Alive	Auyu	3/3	-	-/-	-/-	-	-	+/+	++/+	+	+	-	13
P 10	Yahr II	2011/'12	Asset	38	M	27/'01	11	Alive	Auyu	3/3	-	-/-	-/-	-	-	+/-	+/-	+	-	-	-
P 11	Yahr III	2011/'12	Sien	54	F	40/'98	14	Alive	Auyu	3/3	-	-/-	-/-	-	-	++/+	++/+	+	+	-	-
P 12	Yahr II	2011/'12	Memes	66	F	64/10	2	Alive	Auyu	3/3	-	-/-	-/-	-	-	+/+	+/+	+	-	-	14
P 13	Yahr II	2012	Harapan	60	M	57/09	3	Alive	Auyu	2/2	-	-/-	-/-	-	-	-	+/+	-	-	-	-
P 14	Yahr II	2007	Mur	50	M	38/'95	12	NC	Auyu	2/4	+	-/-	-/-	-	-	+	+	+	-	-	-
P 15	Yahr I	2007	Homilikia	50	M	NC	NC	Alive	Auyu	2/3	+	-/-	-/-	-	-	+/-	+/±	-	-	-	-
P 16	Yahr III	2011	Osso	35	F	33/'09	2	2011	Auyu	3/2	-	-/-	-/-	-	-	+/+	+/+	+	+	+	15

Abbreviations: ALS = amyotrophic lateral sclerosis; BK = bradykinesia; BS = Babinski sign; DTR = deep tendon reflex; Dys = dysphagia/dysarthria; F = female; Fasc = fasciculation; LMN = lower motor neuron signs; M = male; MA = muscle atrophy with weakness; NC = not confirmed by examination; P = parkinsonism; PI = postural impairment; R = rigidity; T = tremor; TA = tongue atrophy; U/L: upper limbs/lower limbs; UMN = upper motor neuron signs.

Age: at the time of final survey; Duration: between subjective onset of illness and last survey or death; Alive: alive in 2012; Onset: subjective onset of illness; Deep tendon reflexes, -: absent, 1: hypoactive, 2: normal, 3: hyperactive, 4: markedly hyperactive with ankle clonus.

Other signs are classified as: -: absent; ±: questionably present; +: present; ++: markedly present.

Family history 1: cousin (possible ALS); 2: grandfather (NC); 3: sister (ALS15); 4: cousin's daughter (P16); 5: cousin (NC); 6: father (NC); 7: sister (ALS 7); 8: sister (P 6) and cousin (NC); 9: father (NC); 10: son: (NC); 11: sister (ALS-P 1) and cousin (NC); 12: mother and father (NC); 13: grandmother (NC); 14: mother (NC); 15: ALS 10 (mother’s cousin).

For peer review only

For peer review only - http://bmjopen.bmj.com/site/about/guidelines.xhtml

Table 4. Cases of ALS and parkinsonism in the field sites

	ALS criteria	Case No.	Crude point prevalence rate (95%CI) among ALS/ ALS-P/ Parkinsonism (2007* or 2010#)
Bade 2007–2012 (population 3800)			
ALS	Definite	ALS 5*	105 (2-208) /100,000 in 2007*
(n=4)	Definite	ALS 6*	
	Definite	ALS 7*	
	Definite	ALS 15*	
ALS-parkinsonism	Definite	ALS-P 4*	53 (0-126) /100,000 in 2007*
(n=2)	Possible	ALS-P 8*	
Parkinsonism (n=1)		P 9*	26 (0-78) /100,000 in 2007*
Ia river 2007–2012 (population 4500)			
ALS	Definite	ALS 4	133(27-240) /100,000 in 2010#
(n=7)	Probable	ALS 8#	
	Probable	ALS 10#	
	Probable	ALS 11#	
	Definite	ALS 12#	
	Definite	ALS 13#	
	Definite	ALS 17#	
ALS-parkinsonism	Possible	ALS-P 2#	89 (2-176) /100,000 in 2010#
(n=4)	Possible	ALS-P 3#	
	Possible	ALS-P 9#	
	Possible	ALS-P 13#	
Parkinsonism		P 10#	67 (0-142) /100,000 in 2010#
(n=3)		P 15#	
		P 16#	
Edera and Dumut rivers 2008–2012 (population: 4100)			
ALS	Definite	ALS 9#	73 (0-156) /100,000 in 2010#
(n=3)	Probable	ALS 14#	
	Definite	ALS 16#	
ALS-parkinsonism	Possible	ALS-P 1	98 (2-193) /100,000 in 2010#
(n=5)	Definite	ALS-P 5#	
	Definite	ALS-P 6#	
	Possible	ALS-P 10#	
	Possible	ALS-P 11#	
Parkinsonism		P 6	122 (15-229) /100,000 in 2010
(n=6)		P 7#	
		P 8#	
		P 11#	
		P 12#	
		P 13#	

*:Active cases in 2007 in Bade
#:Active cases in 2010 in the villages along Ia, Edera and Dumut rivers
95%CI: 95% confidence interval

For peer review only - http://bmjopen.bmj.com/site/about/guidelines.xhtml

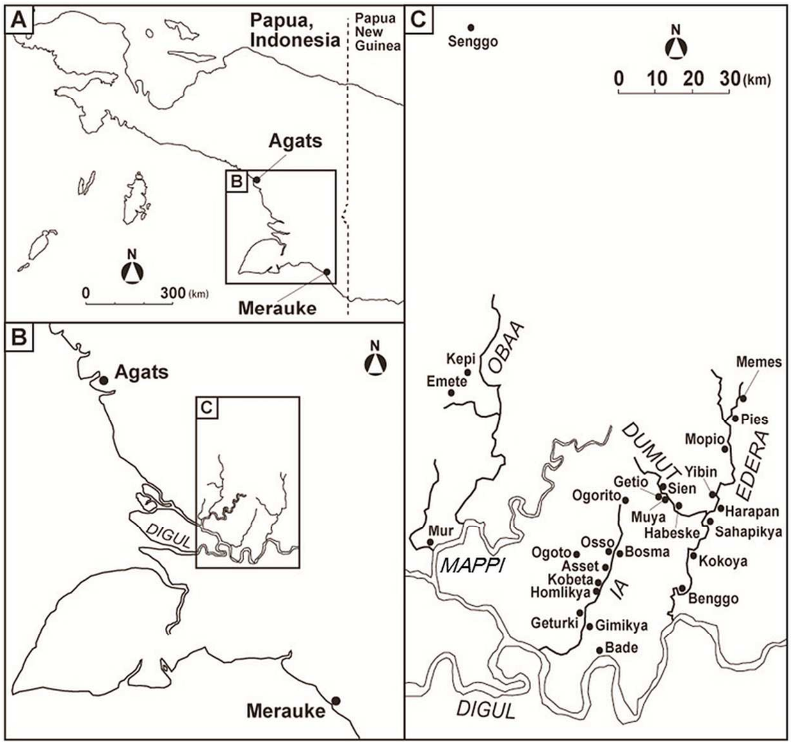

119x90mm (300 x 300 DPI)

For peer review only

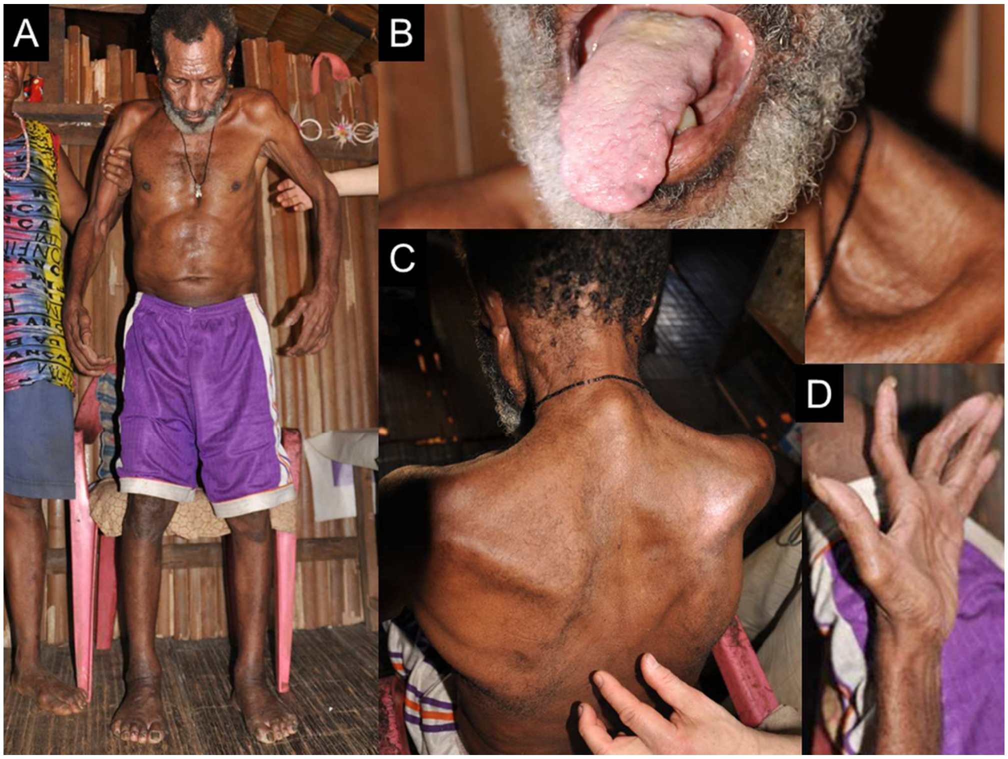

119x90mm (300 x 300 DPI)

For peer review only

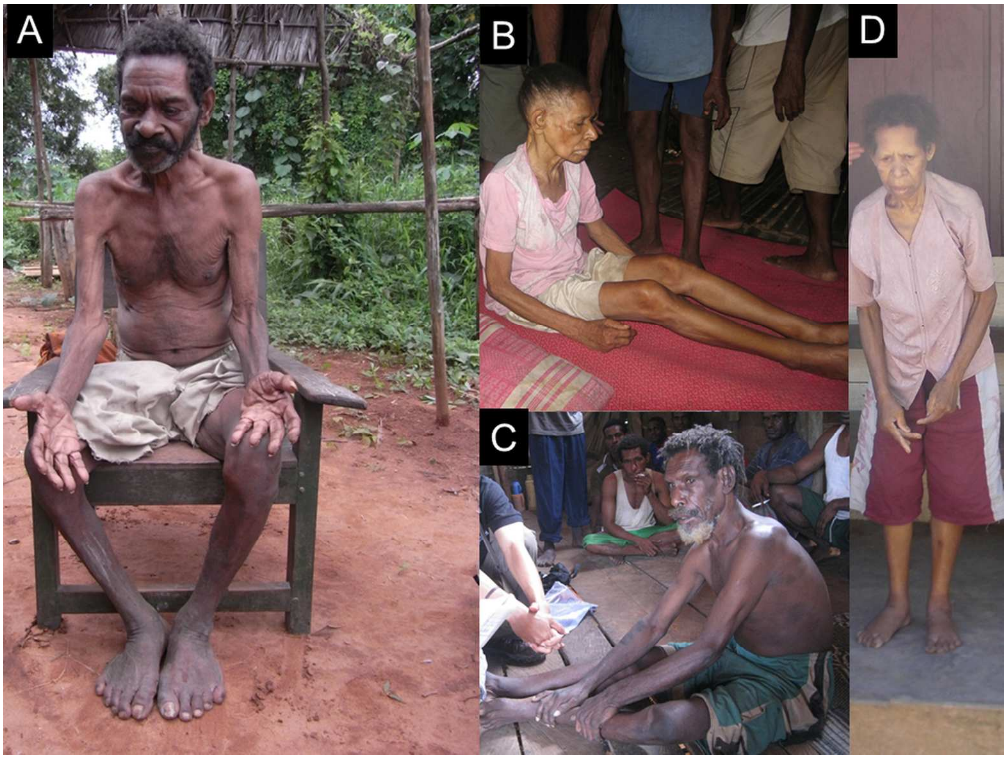

119x90mm (300 x 300 DPI)

For peer review only
